# Tracing the Transcriptomic Changes in Synthetic Trigenomic allohexaploids of *Brassica* Using an RNA-Seq Approach

**DOI:** 10.1371/journal.pone.0068883

**Published:** 2013-07-11

**Authors:** Qin Zhao, Jun Zou, Jinling Meng, Shiyong Mei, Jianbo Wang

**Affiliations:** 1 National Key Laboratory of Hybrid Rice, College of Life Sciences, Wuhan University, Wuhan, China; 2 National Key Laboratory of Crop Genetic Improvement, Huazhong Agricultural University, Wuhan, China; 3 Hubei Academy of Agricultural Science, Wuhan, China; Ben-Gurion University, Israel

## Abstract

Polyploidization has played an important role in plant evolution and speciation, and newly formed allopolyploids have experienced rapid transcriptomic changes. Here, we compared the transcriptomic differences between a synthetic *Brassica* allohexaploid and its parents using a high-throughput RNA-Seq method. A total of 35,644,409 sequence reads were generated, and 32,642 genes were aligned from the data. Totals of 29,260, 29,060, and 29,697 genes were identified in *Brassica* *rapa* , *Brassica* *carinata* , and *Brassica* allohexaploid, respectively. We compared 7,397 differentially expressed genes (DEGs) between *Brassica* hexaploid and its parents, as well as 2,545 nonadditive genes of *Brassica* hexaploid. We hypothesized that the higher ploidy level as well as secondary polyploidy might have influenced these changes. The majority of the 3,184 DEGs between *Brassica* hexaploid and its paternal parent, *B* *. rapa* , were involved in the biosynthesis of secondary metabolites, plant–pathogen interactions, photosynthesis, and circadian rhythm. Among the 2,233 DEGs between *Brassica* hexaploid and its maternal parent, *B* *. carinata* , several played roles in plant–pathogen interactions, plant hormone signal transduction, ribosomes, limonene and pinene degradation, photosynthesis, and biosynthesis of secondary metabolites. There were more significant differences in gene expression between the allohexaploid and its paternal parent than between it and its maternal parent, possibly partly because of cytoplasmic and maternal effects. Specific functional categories were enriched among the 2,545 nonadditive genes of *Brassica* hexaploid compared with the additive genes; the categories included response to stimulus, immune system process, cellular process, metabolic process, rhythmic process, and pigmentation. Many transcription factor genes, methyltransferases, and methylation genes showed differential expression between *Brassica* hexaploid and its parents. Our results demonstrate that the *Brassica* allohexaploid can generate extensive transcriptomic diversity compared with its parents. These changes may contribute to the normal growth and reproduction of allohexaploids.

## Introduction

Polyploidization is an important evolutionary process in eukaryotes, and it is believed that all angiosperms underwent a whole genome duplication event during their evolution [1]. Plant genome sequencing analysis has revealed that species once considered diploids, such as *Arabidopsis thaliana*, *Vinisvitifera* , *Oryza sativa*, and *Populus* *trichocarpa* , are possibly paleopolyploids [2–4] that experienced at least one whole genome duplication event followed by a process of genomic reorganization known as diploidization [5].

Allopolyploids originate through hybridization between different species followed by chromosome duplication. They have contributed to the origin of species and have driven the evolution of angiosperms [6]. The merging of genomes previously adapted to different environments allows allopolyploids to adapt to a wider range of environmental conditions [7]. Some important crops such as wheat, oat, cotton, coffee, and rapeseeds are allopolyploids [8]. The common occurrence of allopolyploids in nature suggests that they have a selective advantage, possibly due in part to heterozygosity and gene redundancy [9,10]. However, increasing gene and genome dosages in allopolyploids also leads to massive gene loss and genome rearrangement. Molecular studies have revealed dynamic and pervasive changes in the polyploid genome and transcriptome that vary among different allopolyploids; these include DNA sequence elimination [11,12], genome rearrangement [13,14], transposon activation [15,16], gene silencing [17], and alterations of gene expression [18,19].

Recent studies have indicated that the transcriptomic changes in allopolyploids may be an adaptive mechanism that facilitates the establishment of gene expression programs and the evolution of a stable species [20]. Changes in gene expression result in the subfunctionalization and neofunctionalization of genes. Subfunctionalization can occur when a homologous gene originating from one parental genome is silenced in specific organs, while the other is silenced in other organs. This is similar to homologs of the alcohol dehydrogenase gene *Adh* in *Gossypium hirsutum* [21]. The duplicated *BnPOX* in the allotetraploid *Brassica napus* retained its function by subfunctionalization of the homologs in different organs, as well as in response to biotic stress [22]. It has been suggested that subfunctionalization is a transition state to neofunctionalization [23]. Transcriptional subfunctionalization and neofunctionalization were both detected in allopolyploid cotton [24]. Changes in gene expression may vary in different allopolyploids, and thus further studies are required.

Molecular markers and microarray data have been applied to analyze genomic and transcriptomic changes in allopolyploids [13,25,26]. The sequencing-based method RNA-Seq allows the entire transcriptome to be surveyed in a high-throughput and quantitative manner. Compared with microarrays and other sequence-based approaches using DNA libraries, RNA-Seq has a low background signal, a large dynamic range of expression levels, more accurate quantification, and high levels of reproducibility [27]. Massively parallel sequencing of DNA using pyrosequencing technology has been applied to obtain an unbiased representation of *Arabidopsis* transcripts [28]. For *Medicago truncatula*, a single 454 Life Science GS20 sequencing approach using a normalized cDNA library has been used to identify novel genes and effectively detect transcript expression [29]. RNA-Seq has provided a powerful approach for determining the expression levels of transcripts and gene function annotation in rice and soybean [30–32]. RNA-Seq has also been applied to explore transcriptomic changes during berry development in grapes, to investigate the transcriptional response of sugar beets to vernalization and gibberellin treatment, and to identify genes involved in cucumber sex determination [16,33,34].

Many studies have used artificially synthesized polyploid materials to determine how duplicated genes and genomes maintain and lose functions during the early stages of polyploidization [35]. Synthesized polyploids can be compared with their direct parental lines, providing an advantage over natural polyploids. The genus *Brassica* is commonly used as a model system in which to perform molecular and phenotypic characterizations of polyploids. Also, the allotetraploid *B. napus* is often synthesized for polyploid studies [26,36]. The synthesized hexaploid *Brassica* is relatively rare, but has played an important role in allopolyploid studies [37]. However, little information is available for artificially synthesized polyploids after the first generation, possibly due to instability. Here, we used a relatively stable trigenomic *Brassica* allohexaploid material and its parents as an experimental system [38].

In this study, we applied RNA-Seq to identify differential gene expression and compared gene function between the trigenomic *Brassica* allohexaploid (BBCCAA, 2n = 54) and its parents. More differences in gene expression were observed between hexaploid *Brassica* and the paternal parent *B* *. rapa* (AA, 2n = 20) than between it and its maternal parent *B* *. carinata* (BBCC, 2n = 34). The differentially expressed genes were involved in a wide range of development and stress resistance processes.

## Materials and Methods

### Plant materials and RNA extraction

A trigenomic *Brassica* allohexaploid (BBCCAA genome) and its parents *B* *. carinata* (BBCC) and *B* *. rapa* (AA) were used in this study. The *B* *. carinata* (accession number CGN03953 in the Center for Genetic Resources, Wageningen, the Netherlands), and Chinese *B* *. rapa* (cultivar name, Dongkoutian) were grown and self-pollinated in the field of Huazhong Agricultural University. The trigenomic *Brassica* allohexaploid was generated by inter-specific hybridization followed with chromosome doubling, by using *B* *. carinata* as the maternal parent and *B* *. rapa* as the paternal parent in the year of 2004 [38]. The synthesized allohexaploid (code as C14) used in this study was in the fourth generation. After chromosome identification of the synthesized allohexaploid, it was confirmed to be euploid (2n=54). The plant materials were grown in a greenhouse at Hubei Academy of Agricultural Science under natural conditions of light and temperature (Wuhan, China). Young leaves from five replicate individuals of both parents and the allohexaploid were collected for RNA extraction when the materials have grown for three months.

For Illumina sequencing, total RNA was isolated using TRIzol reagent according to the manufacturer’s protocal (Invitrogen, Burlington, ON, Canada) and treated with RNase-free DNase I (Fermentas, Canada). RNA quality was confirmed using a 1% agarose gel with the RNA 6000 Nano Assay Kit and Agilent 2100 Bioanalyzer. The total RNA was stored at -80°C for later use.

### cDNA library construction and sequencing

Poly (A)-containing mRNA molecules were purified from total RNA using poly (T) oligo-attached magnetic beads. The mRNA was fragmented into small pieces (59±1bp) by treatment with divalent cations at elevated temperature. Then the double-stranded cDNA was synthesized using the SuperScript Double-Stranded cDNA Synthesized kit (Invitrogen, Camarillo, CA, USA) and random hexamer primers (Illumina, San Diego, CA, USA). The double-stranded cDNA was further subjected to end-repair and phosphorylation using T4 DNA polymerase, Klenow DNA polymerase and T4 PNK (Fermentas, canada). These repaired cDNA fragments were 3' adenylated using Klenow 3' to 5' exo-polymerase and ligated Illumina Paired-end adapters to the ends. The required fragments were separated by size on an agarose gel and excised from the gel. PCR was performed to enrich the purified cDNA fragments. After validation with a Bioanalyzer Chip DNA 1000 series II (Agilent, Santa Clara, CA, USA), the cDNA library was sequenced on an Illumina HiSeq2000 sequencing platform. Files containing reads sequence and quality scores were deposited in the Gene Expression Omnibus of National Center for Biotechnology Information with accession numbers GSE46299.

### Mapping of reads and expression analysis of genes

The raw Illumina sequencing files were base called into sequence data. We cleaned the raw reads by removing adapter sequences and low-quality sequences (reads has more than 50% of low-quality bases with a quality value ≤ 5). The *B* *. rapa* genome and gene information were downloaded from the *Brassica* database (BRAD, http://brassica.org/brad/) [39]. We used SOAPaligner/soap2 software to map the reads to the *B* *. rapa* reference genome with a permission of two base mismatches.

For gene expression analysis, the raw gene expression counts were measured by quantifying the number of reads that were mapped to the *B* *. rapa* genome sequences using ERANGE 4.0 software (http://woldlab.caltech.edu/gitweb/). ERANGE reports the number of mapped reads per kilobase of exon per million mapped reads, measuring the transcriptional activity for each gene [40].

### Gene annotation

To annotate the detected genes, a BLASTx search against the NCBI non-redundant protein (Nr) database (http://www.ncbi.nlm.nih.gov/) and the Swiss-Prot protein database was performed with an E-value threshold of less than 10^-5^. With Nr annotation, Blast2GO [41] was used to obtain GO annotation of genes according to molecular function, biological process and cellular component ontologies (http://www.geneontology.org/). WEGO [42] was used for GO functional classification of all genes and to plot the distribution of gene functions. Pathway assignments were carried out based on the KEGG database [43].

The putative TFs in these genes were got by querying *B* *. rapa* Transcription Factors in the Plant Transcription Factor Database (http://planttfdb.cbi.edu.cn/).

### Differential gene expression analysis

Significantly differential expressed genes were determined using R package DEGseq [44]. We defined genes with fold change ≥ 2 and FDR ≤ 0.001 as differentially expressed genes (DEGs). GO terms enrichment was performed to the DEGs. To group DEGs with similar expression patterns, a hierarchical clustering was generated using the expression values from each library. The analysis was conducted using MATLAB 7.8 software with Pearson correlation as the distance measure. The cluster tree concluded distinct clusters which contained genes with a unique expression profile by visual inspection.

## Results

### Illumina sequencing and the data analysis

We previously performed a *B* *. carinata* × *B* *. rapa* cross to double the genome, with *B* *. carinata* as the maternal parent and *B* *. rapa* as the paternal parent [38]. We used the RNA-Seq method to compare differences in gene expression between *Brassica* hexaploid and its parents. Using the Illumina HiSeq 2000 platform, a total of 35,644,409 sequence reads (59 bp in length) were generated from cDNA libraries derived from *B* *. rapa* , *B* *. carinata* , and *Brassica* hexaploid. Each library generated more than 10 million reads, reaching the saturation level of gene identification. SOAPaligner/soap2 software was used to map the reads to the *B* *. rapa* reference genome (http://brassica.org/brad/) with a tolerance of two base mismatches. Reads that mapped to a unique sequence were the most critical subset of the RNA-Seq libraries, as they can explicitly identify a gene. In total, 70.35%, 46.20%, and 54.18% of reads uniquely matched to a genomic location in the *B* *. rapa* , *B* *. carinata* , and *Brassica* hexaploid libraries, while unmapped reads accounted for 23.39%, 46.37%, and 39.26%, respectively (Table 1). It is possible that *B* *. carinata* had the smallest percentage of mapped reads because of genome differentiation between *B* *. carinata* (BBCC) and *B* *. rapa* (AA, the reference genome for gene annotation). Meanwhile, the hexaploid received a middle-parent number. Digital gene expression analysis was performed for the uniquely mapped reads. To compare transcript levels within and between samples, the raw read counts were normalized using a variation of the reads/Kb/million (RPKM) method. The RPKM method can avoid biases caused by different gene exon sizes and the different number of read sequences obtained from the three libraries.

**Table 1:** Number of reads matched to *B* *. rapa* genome.

	*B* *. rapa*	*B* *. carinata*	*Brassica* hexaploid
Total reads	12,216,743	11,859,888	11,567,778
Mapped reads	9,359,218 (76.61%)	6,360,133 (53.63%)	7,026,728 (60.74%)
Unique match	8,594,083 (70.35%)	5,479,383 (46.20%)	6,267,830 (54.18%)
Multi-position match	765,135 (6.26%)	880,750 (7.43%)	758,898 (6.56%)
Unmapped reads	2,857,525 (23.39%)	5,499,755 (46.37%)	4,541,050 (39.26%)

**Figure 1:**
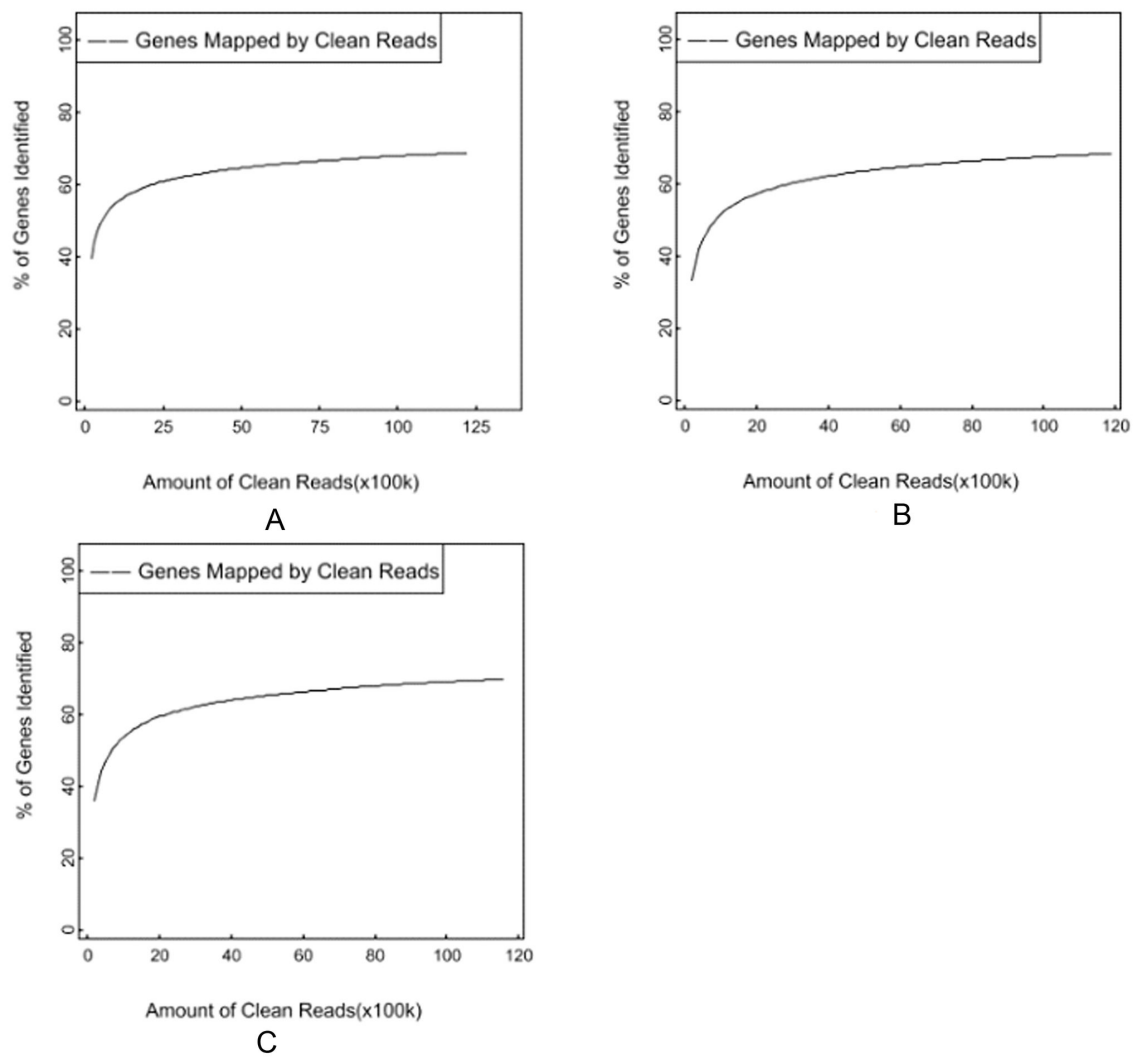
The saturation analysis of genes detected by sequencing reads. The gene saturation analysis are taken in the libraries of *B* *. rapa* (A), *B* *. carinata* (B), and *Brassica* hexaploid (C) respectively. The curve indicated that the number of detected genes almost ceases to increase when the number of reads reaches 2 million.

**Figure 2:**
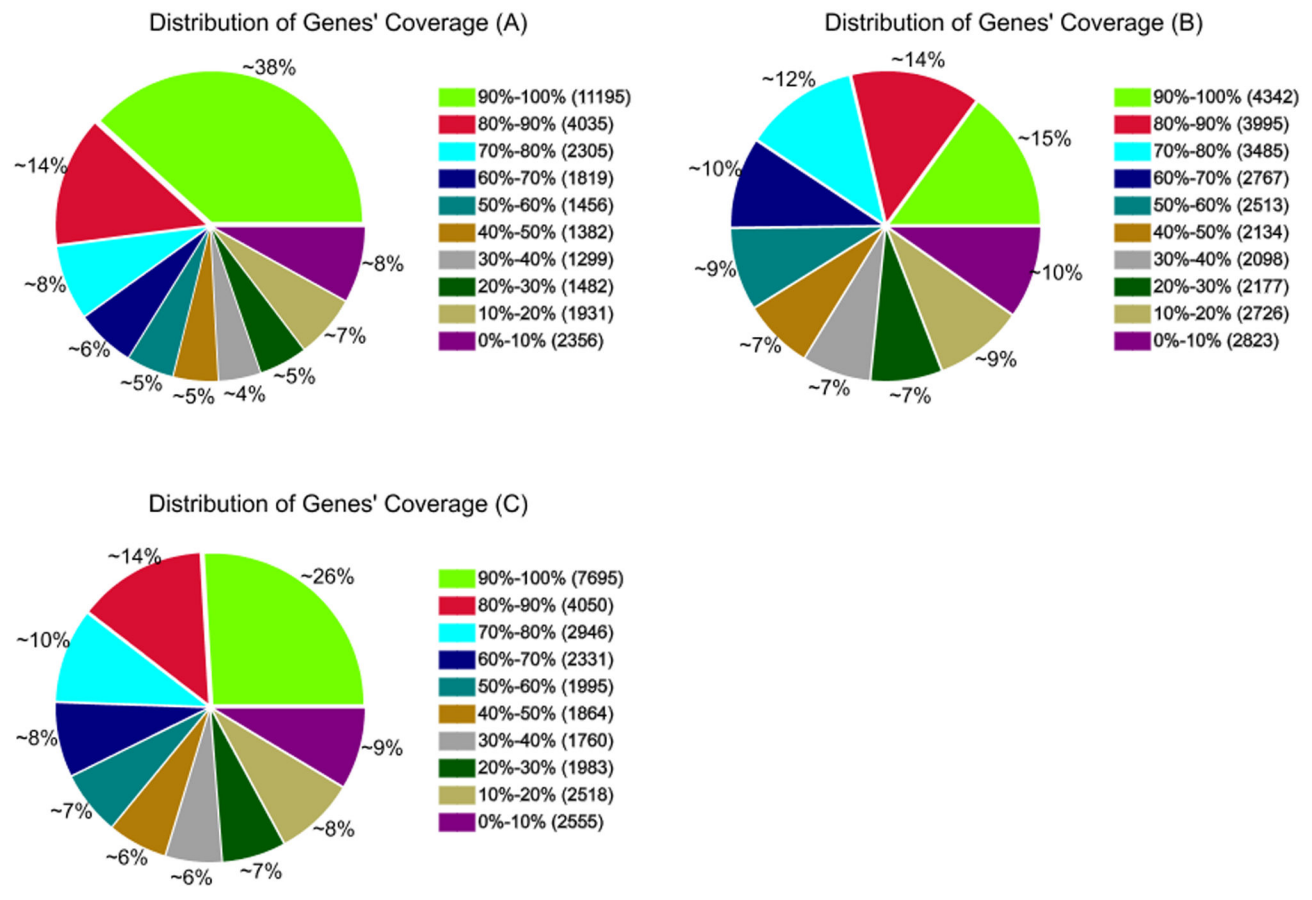
The different read coverage of genes in three libraries. The genes with different read coverage were indicated by different colours. The capital letters A, B, and C in brackets refer to libraries of *B* *. rapa* , *B* *. carinata* , and *Brassica* hexaploid respectively.

Our RNA-Seq data revealed 32,642 genes in *Brassica* hexaploid and its parents, accounting for 79.28% of the inferred genes in the *B* *. rapa* genome [39]. To confirm whether the number of detected genes increased proportionally with the sequencing amount (total tag number), a saturation analysis was performed. The saturation trend (Figure 1) showed that the number of detected genes stopped increasing when the number of reads reached 2 million. A total of 70.95% of the detected genes had at least 30 matching reads when including all three libraries. The genes matched to reads had different percentages of their sequences covered by the reads, possibly because of the heterogeneity and redundancy of mRNAs. Genes having more than 90% of their sequence covered by reads constituted the most abundant category, accounting for 38%, 15%, and 26% of the matched genes in *B* *. rapa* , *B* *. carinata* , and *Brassica* hexaploid, respectively. The second most abundant category was genes with 80% to 90% sequence coverage. The percentages of matched genes were similar among the other eight categories, established according to increments of 10% (Figure 2).

### Genes expressed in *Brassica* hexaploid and its parents, and their functional analysis

The 32,642 genes expressed in *Brassica* hexaploid and its parents had lengths of 100 to ≥ 2,000 bp. Genes of 500–1,000 bp were the most abundant, accounting for 30.71% of the total genes, and genes with lengths between 1,000 and 1,500 bp accounted for 25.41%. Genes with lengths of 100–500 bp, 1,500–2,000 bp, and ≥ 2,000 bp represented 15.46%, 13.62%, 14.80% of the total genes, respectively (Table 2).

**Table 2:** Gene sequence length distribution.

Gene length (bp)	Total number	Percentage
100-500	5048	15.46%
500-1000	10024	30.71%
1000-1500	8293	25.41%
1500-2000	4446	13.62%
≥2000	4831	14.80%
Total	32642	100%

**Figure 3:**
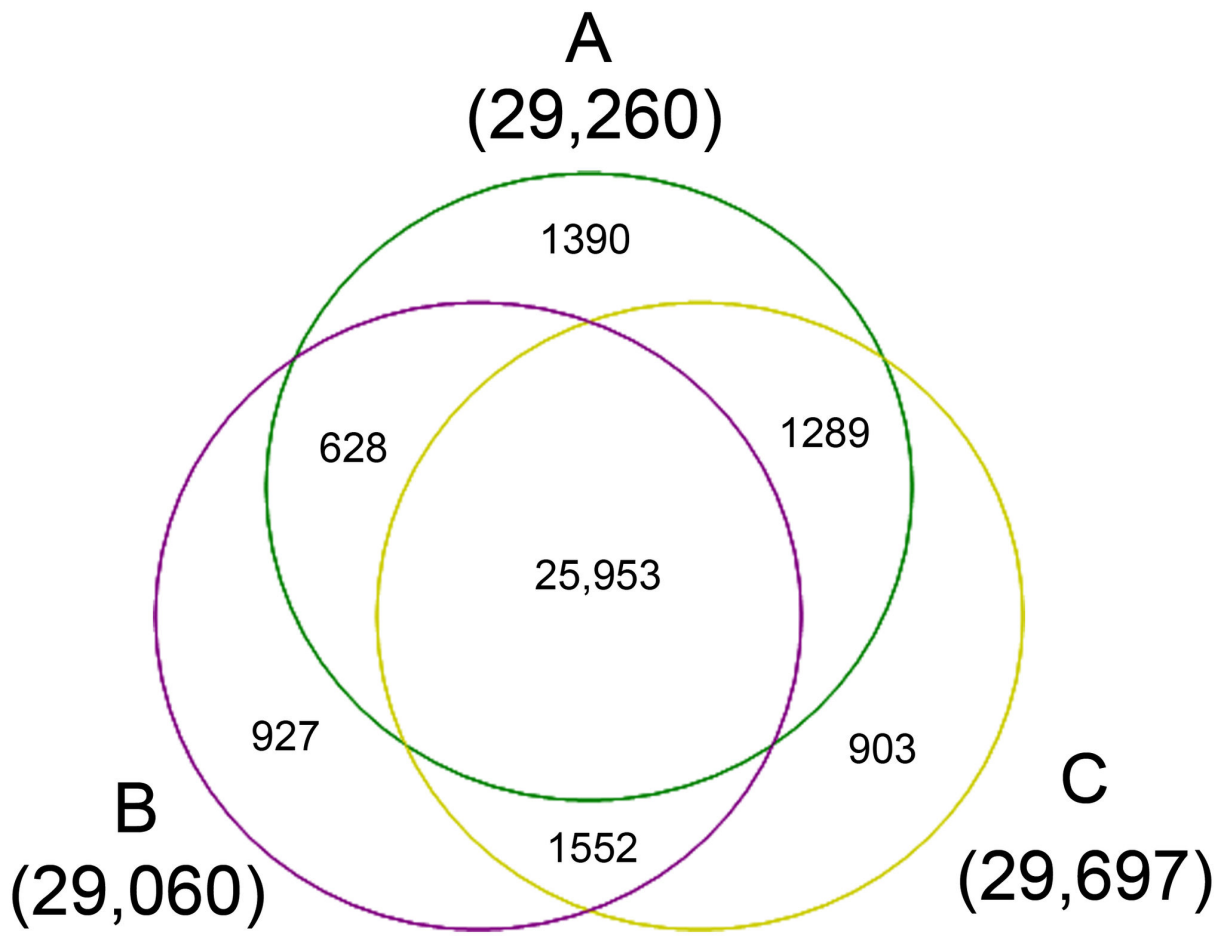
Venn diagram showing genes expressed in *Brassica* hexaploid and its parents. A, *B* *. rapa* ; B, *B* *. carinata* ; C, *Brassica* hexaploid.

Similar numbers of genes were detected in *B* *. rapa* , *B* *. carinata* , and the allohexaploid (Figure 3). Of the 25,953 genes shared by *Brassica* hexaploid and its parents, 628 were co-expressed in *B* *. rapa* and *B* *. carinata* , 1,552 were co-expressed in *B* *. carinata* and *Brassica* hexaploid, and 1,289 were co-expressed in *B* *. rapa* and *Brassica* hexaploid. However, 1,390 ( *B* *. rapa* ), 927 ( *B* *. carinata* ), and 903 ( *Brassica* hexaploid) genes were specifically expressed, respectively. Among the 25,953 genes expressed in all three *Brassica* and the 3,469 genes co-expressed in two, many showed variable gene expression levels between *Brassica* hexaploid and its parents.

**Figure 4:**
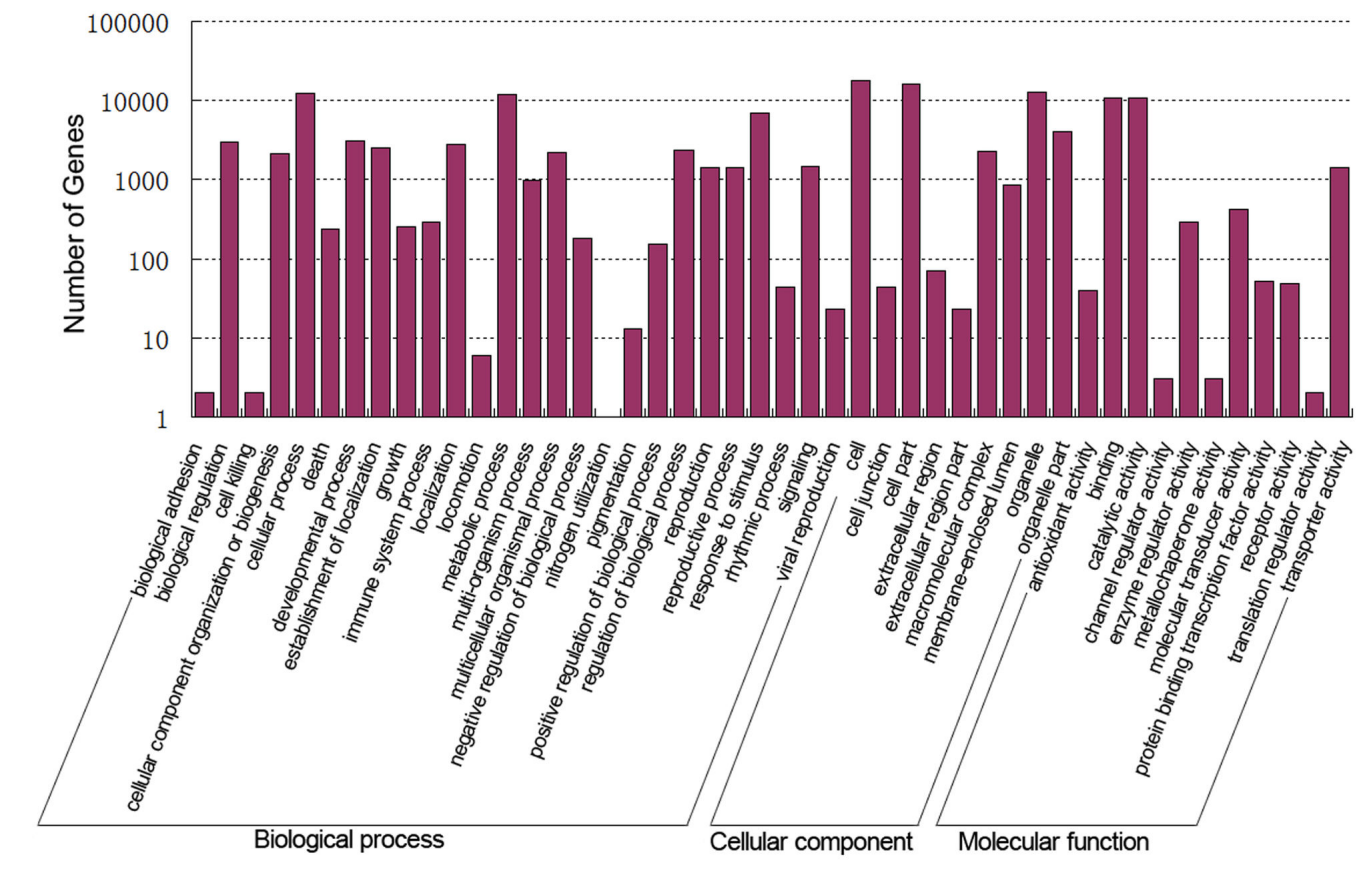
Gene ontology (GO) annotations of all detected genes. The histogram shows the result of classifying 24658 genes to the secondary classification of GO terms. The y-axis indicates the number of genes in a functional term.

To further explore the functional significance of all detected genes and the genes specifically expressed in each library, we assigned the gene sequences to gene ontology (GO) functional classes (http://www.geneontology.org/) using Blast2GO (version 2.3.5) (http://www.blast2go.org/). A total of 32,642 genes were detected, and 24,658 (75.54%) had at least one GO annotation based on sequence similarity. We categorized these 24,658 genes according to the secondary classification of GO terms, assigning genes to 46 functional groups of the three main GO classification categories (Figure 4). Genes were classified into 26 functional groups of the biological process GO category, 11 functional groups of the molecular function category, and nine functional groups of the cellular component category. The terms cellular process (GO:0009987) and metabolic process (GO:0008152), which included 12,537 and 12,007 genes, respectively, were dominant in biological process category. In the molecular function and cellular component categories, the terms binding (GO:0005488) and cell (GO:0005623) were most significantly enriched, with 18,115 and 10,922 genes, respectively. There was also an enrichment for the terms response to stimulus (GO:0050896), catalytic activity (GO:0003824), and cell part (GO:0044464) in the three main categories. Among the genes specifically expressed in *B* *. rapa* (1,390 genes), *B* *. carinata* (927 genes), and *Brassica* hexaploid (903 genes), we observed a similarly high distribution of genes encoding proteins involved in metal ion binding and antioxidation, with enrichment of the GO terms transition metal ion binding, metal ion binding, binding, and oxidoreductase activity.

Pathway enrichment analysis was performed on all expressed transcripts by mapping them to reference canonical pathways in the Kyoto Encyclopedia of Genes and Genomes (KEGG) (http://www.genome.ad.jp/kegg/). A total of 21,092 genes were mapped to 125 KEGG pathways. Pathways with the highest numbers of genes included metabolic pathways (4,529 genes), biosynthesis of secondary metabolites (2,547 genes), plant–pathogen interactions (1,867 genes), and plant hormone signal transduction (1,684 genes) (Table S1). These pathways are involved in major growth processes and stress reactions in plants.

### Differentially expressed genes and functional analysis

**Figure 5:**
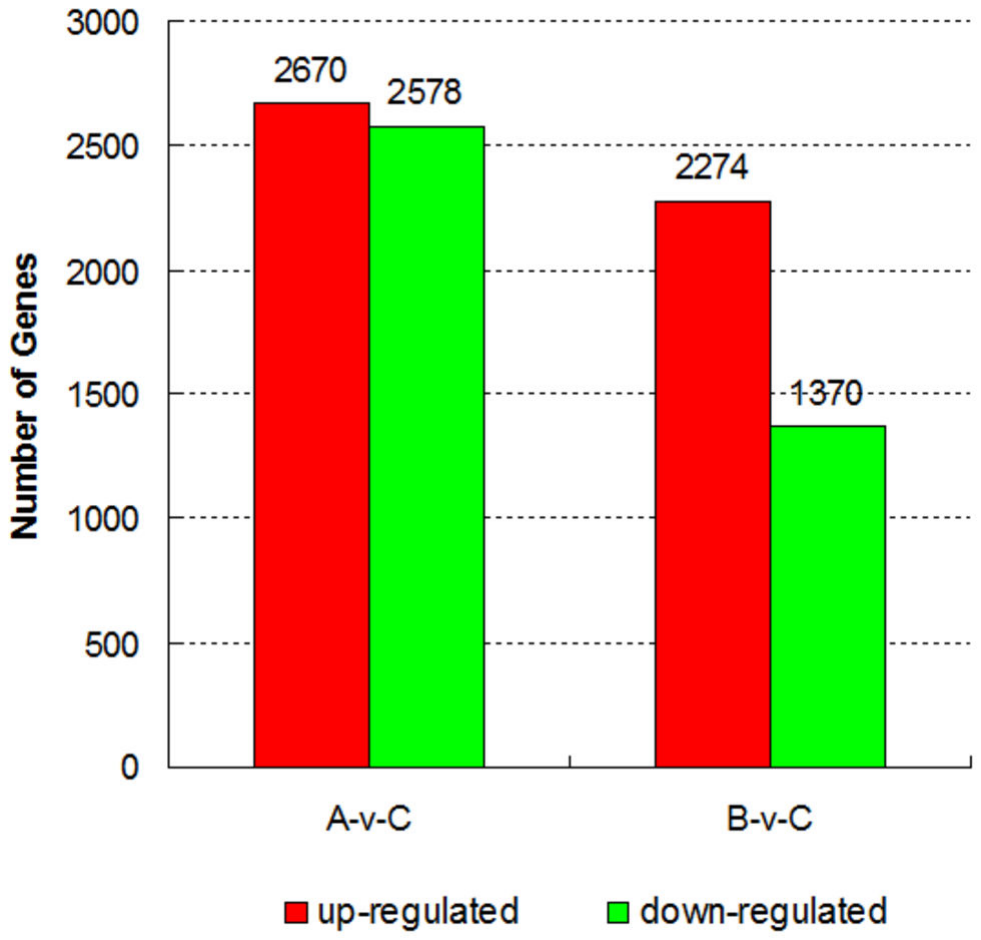
Differentially expressed genes detected between *Brassica* hexaploid and its parents. The number of up-regulated and down-regulated genes between *Brassica* hexaploid (C) and *B* *. rapa* (A), *Brassica* hexaploid (C) and *B* *. carinata* (B) are revealed.

Differentially expressed genes (DEGs) between the *Brassica* hexaploid and its parents were identified using a likelihood ratio test [45] to compare the gene RPKM values. Applying the algorithm test built by Audic et al. [46], we considered significantly altered genes to be those with expression changes of at least two-fold and false discovery rates (FDR) of no more than 0.001. As a result, 7,397 genes showed significant differential expression between *Brassica* hexaploid and its parents. Comparing *Brassica* hexaploid and *B* *. rapa* , 2,670 genes were upregulated and 2,578 genes were downregulated (Figure 5 and Table S2). Compared with *B* *. carinata* , *Brassica* hexaploid had 2,274 upregulated genes and 1,370 downregulated genes (Figure 5 and Table S3). The total value based on the fold change of DEGs between *Brassica* hexaploid and *B* *. rapa* was 15,640.92. Between *Brassica* hexaploid and *B* *. carinata* , the value was 10,371.10. These values suggest that there were more DEGs with larger differences in expression between *Brassica* hexaploid and *B* *. rapa* .

**Figure 6:**
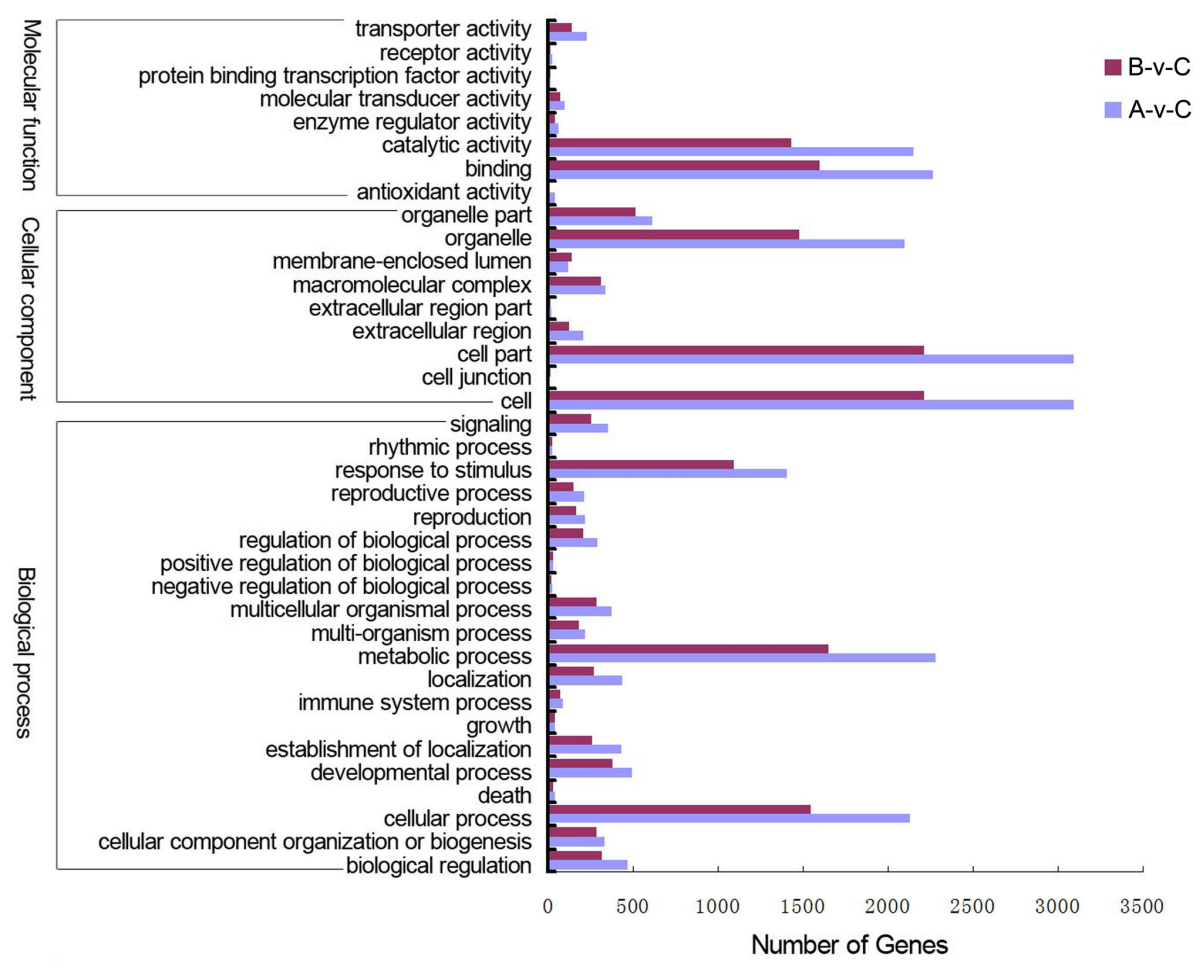
Functional annotation of DEGs between *Brassica* hexaploid and its parents based on GO terms. DEGs between *Brassica* hexaploid (C)- *B* *. rapa* (A) and *Brassica* hexaploid (C)- *B* *. carinata* (B) are grouped to the secondary classification of GO terms. There are 8, 9, and 20 functional groups in the three main categories (molecular function, cellular component, and biological process) of GO classification, respectively.

Using GO term enrichment analysis, we categorized the DEGs between *Brassica* hexaploid and *B* *. rapa* and between *Brassica* hexaploid and *B* *. carinata* according to the secondary classification of GO terms. There were genes belonging to eight, nine, and 20 functional groups in the three main GO classification categories: molecular function, cellular component, and biological process, respectively (Figure 6). The terms binding (GO:0005488) and metabolic process (GO:0008152) were dominant in the molecular function and biological process categories. In the cellular components category, the terms cell (GO:0005623) and cell part (GO:0044464) were equally common and occurred more frequently than the other functional groups in this category. The catalytic activity (GO:0003824), transporter activity (GO:0005215), organelle (GO:0043226), cellular process (GO:0009987), and response to stimulus (GO: 0050896) functional groups were also significantly enriched in DEGs between *Brassica* hexaploid and its parents.

Mapping of the DEGs to KEGG pathways revealed significant enrichment of specific pathways compared with the distribution of the whole transcriptome. Among the DEGs between *Brassica* hexaploid and its paternal parent, 3,184 had a KEGG pathway annotation. Pathways related to secondary metabolite biosynthesis, amino acids, nitrogen, sulfur, lipid, vitamin B6 metabolism, plant–pathogen interactions, photosynthesis, and circadian rhythm were overrepresented. Between *Brassica* hexaploid and its maternal parent, 2,233 DEGs mapped to KEGG pathways, with enrichment of pathways regarding plant–pathogen interactions, plant hormone signal transduction, ribosomes, limonene and pinene degradation, photosynthesis, and the biosynthesis of secondary metabolites such as stilbenoid, diarylheptanoid, gingerol, glucosinolate, flavone, and flavonol. Thus, the DEGs between *Brassica* hexaploid and its parents were involved in a wide range of plant physiological processes that may be essential for the differences in morphology and physiology between *Brassica* hexaploid and its parents.

### Clustering of differentially expressed genes

**Figure 7:**
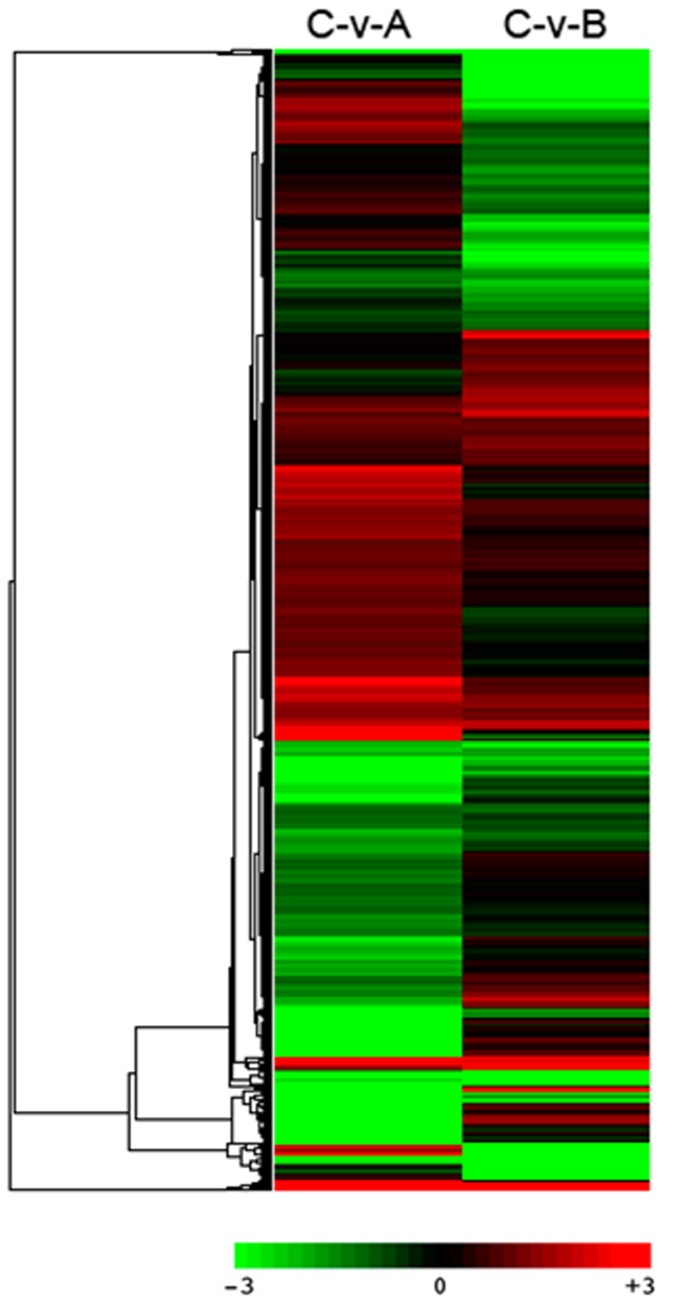
Hierarchical clustering analysis of 7397 DEGs based on log ratio RPKM data. A, *B* *. rapa* ; B, *B* *. carinata* ; C, *Brassica* hexaploid. The column represents individual experiment, and row represents individual gene. Genes up-regulated is represented by red, and genes down-regulated is represented by green.

We generated a hierarchical clustering of the 7,397 DEGs to examine the similarity and diversity of expression profiles. The RPKM value was transformed by dividing the value of a gene in one library by the control library value, and this was then expressed as the binary logarithm. Figure 7 shows the results as displayed by Java Treeview. The transformed values are represented by different colors, with red representing positive values and green representing negative values.

**Figure 8:**
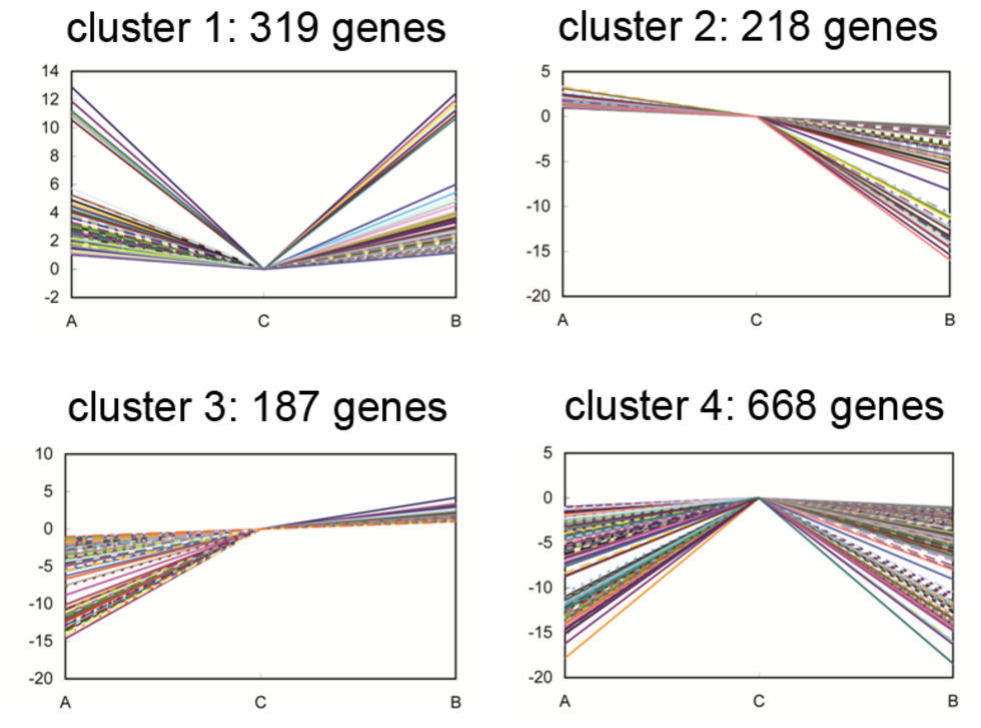
Four clusters of gene expression pattern. A, *B* *. rapa* ; B, *B* *. carinata* ; C, *Brassica* hexaploid. The four clusters of gene expression pattern were grouped by up or down regulation in *Brassica* hexaploid compared to its parents.

We analyzed the associations among DEGs based on the correlations and differences among their expression patterns as revealed by the hierarchical clustering. We classified 1,392 genes with significant differential expression between *Brassica* hexaploid and *B* *. rapa* and between *Brassica* hexaploid and *B* *. carinata* into four expression patterns using two-dimensional hierarchical clustering. Cluster 1 had 319 genes; cluster 2 had 218; cluster 3 had 187; and cluster 4 had 668 (Figure 8 and Table S4). Cluster 4, the most abundant group, was composed of genes with the highest expression in *Brassica* hexaploid. As the second most abundant group, cluster 1 contained genes negatively modulated in *Brassica* hexaploid compared with its parents. Genes in cluster 2 were downregulated in *Brassica* hexaploid compared with its paternal parent *B* *. rapa* , and were upregulated compared with its maternal parent *B* *. carinata* . Conversely, cluster 3 included genes upregulated in *Brassica* hexaploid compared with *B* *. rapa* and downregulated compared with *B* *. carinata* .

We also categorized 7,397 DEGs showing differential expression between *Brassica* hexaploid and *B* *. rapa* or *Brassica* hexaploid and *B* *. carinata* into four groups (Table S5). Genes in groups 1, 2, 3, and 4 had the same expression profiles as genes in clusters 1, 2, 3, and 4. There were 2,016, 1,701, 1,327, and 2,353 genes in groups 1, 2, 3, and 4, respectively. We calculated the functional category abundance in the four groups and identified differences in gene distribution. Excluding genes encoding proteins with functions in adenyl ribonucleotide binding, nucleic acid binding, transition metal ion binding, protein binding, structural molecule activity, and protein kinase activity (which were enriched in all four groups), there were more genes involved in molecular transducer activity, hydrolase activity, and protein phosphatase regulator activity in group 1, and more genes in the functional categories of metal ion binding, transcription regulator activity, carboxylesterase, and DNA binding in group 2. For group 3, cofactor binding, transferase activity, endopeptidase activity, RNA binding, and guanyl ribonucleotide binding functions were common. Genes categorized as carbohydrate binding, phosphatase activity, oxidoreductase activity, catalytic activity, and kinase activity were more enriched in group 4 than in the other three groups.

### Nonadditive genes expressed in *Brassica* hexaploid

We compared the expression levels of genes in *Brassica* hexaploid with the midparent value (MPV). Genes showing at least a two-fold change between *Brassica* hexaploid and MPV (with FDRs of no more than 0.001) were considered nonadditive genes; all others were considered additive genes. A total of 2,545 genes, accounting for 7.797% of the total detected genes, were nonadditively expressed in *Brassica* hexaploid. Of these nonadditive genes, 2,445 showed differential expression either between *Brassica* hexaploid and *B* *. rapa* or between *Brassica* hexaploid and *B* *. carinata* . Among the 2,445 differentially expressed nonadditive genes, 1,002 belonged to group 1, 163, to group 2, 43, to group 3, and 1,237, to group 4.

**Figure 9:**
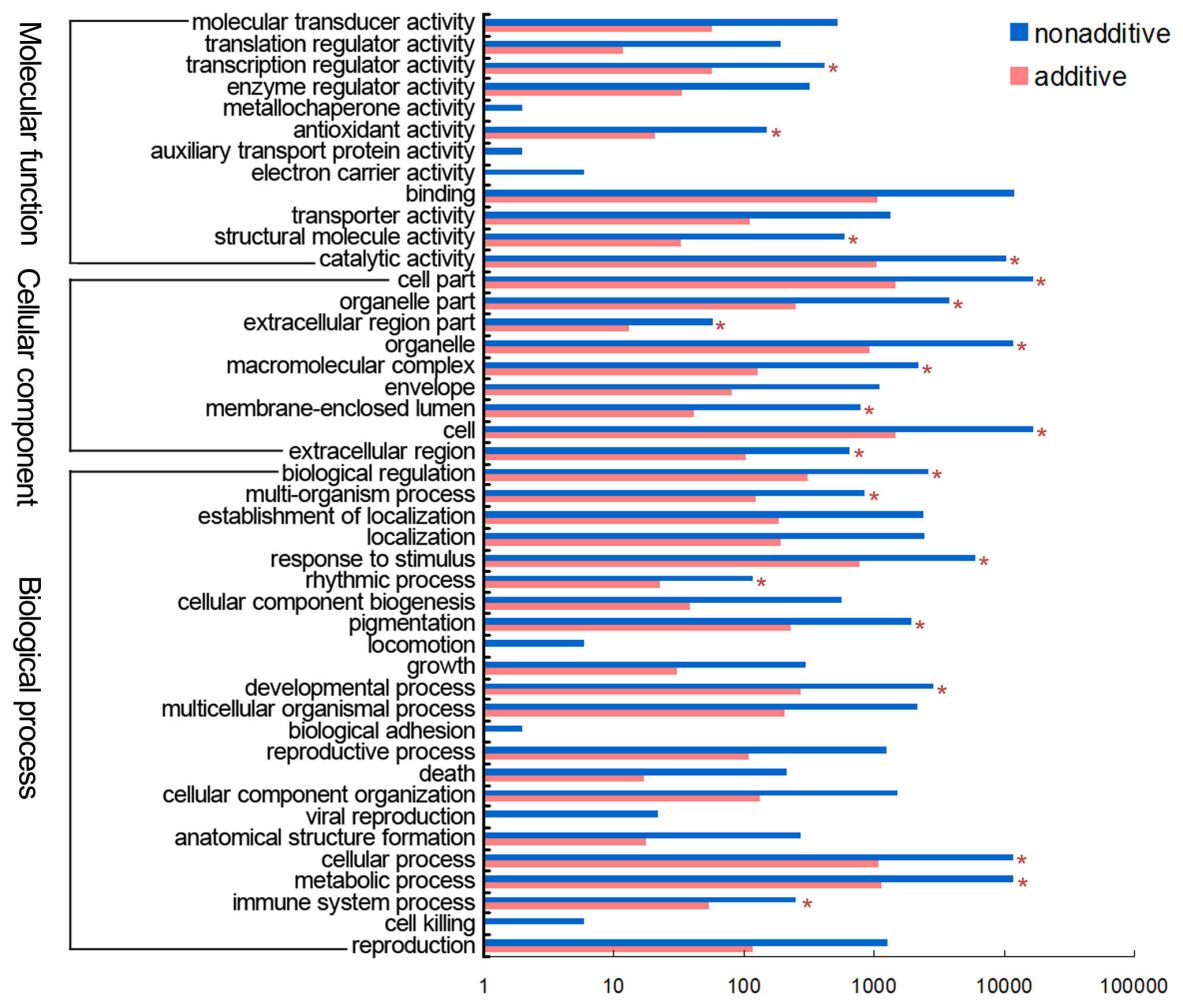
Functional annotation of nonadditive genes and additive genes in *Brassica* hexaploid based on GO terms. Nonadditive genes and additive genes are grouped to the secondary classification of GO terms. There are 12, 9, and 23 functional groups in the three main categories (molecular function, cellular component, and biological process) of GO classification, respectively.

To explore the differences in functional category distribution between additive and nonadditive genes, we assigned them to secondary classification GO terms. The genes belonged to 12, 9, and 23 functional groups in main GO categories of molecular function, cellular component, and biological process, respectively (Figure 9). In the molecular function category, the terms transcription regulator activity (GO:0030528), antioxidant activity (GO:0016209), structural molecular activity (GO:0005198), and catalytic activity (GO:0003824) showed significant differences in gene numbers between additive and nonadditive genes, with transcription regulator activity, antioxidant activity, and catalytic activity being significantly enriched in nonadditive genes. In the cellular component category, the numbers of additive and nonadditive genes differed significantly for the functional terms cell part (GO:0044464), organelle part (GO:0044422), extracellular region part (GO:0044421), organelle (GO:0043226), macromolecular complex (GO:0032991), membrane-enclosed lumen (GO:0031974), cell (GO:0005632), and extracellular region (GO:0005576). Four of these groups (cell part, extracellular region part, cell, and extracellular region) were significantly enriched in nonadditive genes. In the biological process category, nine functional groups, including biological regulation (GO:0065007), multi-organism process (GO:0051704), response to stimulus (GO:0050896), rhythmic process (0048511), pigmentation (GO:0043473), developmental process (GO:0032502), cellular process (GO:0009987), metabolic process (0008152), and immune system process (GO:0002376), had significantly more nonadditive genes than additive genes.

### Analysis of putative transcription factor and methylase genes

We queried *B* *. rapa* transcription factor (TF) genes in the Plant Transcription Factor Database (http://planttfdb.cbi.edu.cn/) and identified 589 putative TF genes, which could be classified into 49 families (Table S6). Of the 589 putative TF genes, 143 genes belonging to 36 families showed significant differential expression, accounting for 24.28% of the total TF genes (Table S6). There were 101 putative TF genes differentially expressed between *Brassica* hexaploid and *B* *. rapa* , with 49 genes upregulated and 52 genes downregulated in *Brassica* hexaploid. Seventy-six putative TF genes showed differential expression between *Brassica* hexaploid and *B* *. carinata* : 46 genes were upregulated and 30 were downregulated in *Brassica* hexaploid.

The TCP gene family is a family of transcription factors present in various plant species. We detected 15 TCP genes in our RNA-Seq data, and five showed significantly different expression between *Brassica* hexaploid and its parents (Table 3). Three TCP genes (Bra001992, Bra003959, and Bra006616) were expressed at higher levels in *Brassica* hexaploid compared with *B* *. rapa* , and two genes (Bra003959 and Bra006616) were upregulated in *Brassica* hexaploid compared with *B* *. carinata* . The other two differentially expressed TCP genes (Bra009679 and Bra011803) showed lower expression levels in *Brassica* hexaploid compared with *B* *. rapa* . Some putative target genes of the TCP gene family also showed differential expression. Ten of the 32 genes potentially regulated by TCP genes that we examined showed significant differential expression between *Brassica* hexaploid and its parents.

**Table 3:** TCP genes detected in RNA-Seq data.

GeneID	Length	A_RPKM	B_RPKM	C_RPKM
Bra001992	3102	0.364129	1.124772	2.335480
Bra003959	1122	1.901563	4.318980	29.517329
Bra006616	858	5.850964	3.162822	12.263409
Bra009679	486	26.340172	20.340797	10.647648
Bra011803	1242	21.927688	5.306295	10.277295
Bra001887	330	-	-	1.568108
Bra010597	1491	0.168348	-	-
Bra004415	525	0.239054	2.953689	2.628448
Bra004788	1287	0.975161	0.150611	0.938184
Bra003834	1521	4.373220	1.146958	2.494952
Bra008235	684	26.421721	26.638257	30.261735
Bra003890	288	20.917196	2.692164	14.374324
Bra011417	1017	18.140577	10.292168	10.515729
Bra003179	1671	16.373245	15.195986	17.651773
Bra004223	2622	1.292367	2.661361	1.513087

A, *B* *. rapa* ; B, B*. carinata*; C, *Brassica* hexaploid. The five genes marked by underline were significantly differently expressed genes between *Brassica* hexaploid and its parents.

To explore the underlying mechanism of changes in DNA methylation, we determined the expression levels of putative methyltransferase and methylase genes identified in *Brassica* hexaploid and its parents (Table S7). Among 157 putative methyltransferase genes, 47 (29.94%) exhibited significant differential expression between *Brassica* hexaploid and its parents. Between *Brassica* hexaploid and *B* *. rapa* , 39 putative methyltransferase genes were differentially expressed in the *Brassica* hexaploid; 32 were upregulated and seven were downregulated. Between *Brassica* hexaploid and *B* *. carinata* , 16 putative methyltransferase genes were upregulated and four were downregulated. Compared with the number of methyltransferase genes, fewer putative methylase genes were identified, with 11 genes being detected, one of which (Bra035280) was also annotated as a methyltransferase. Among these 11 genes, three (not including Bra035280) were significantly upregulated and one was significantly downregulated in *Brassica* hexaploid compared with *B* *. rapa* . No putative methylase gene showed significantly different expression between *Brassica* hexaploid and *B* *. carinata* .

## Discussion

RNA-Seq has been used for transcriptomic analysis in a number of plant species [16,30–34]. Here, we used RNA-Seq to trace the transcriptomic changes between *Brassica* hexaploid and its parents. Before bioinformatic analysis could be performed, we required a reference genome for aligning the reads. In previous studies, an *Arabidopsis* 70-mer oligo microarray was used to measure gene expression in resynthesized *B. napus* [26]. Considering the close relationship between *Arabidopsis* and *Brassica* , we mapped the *Brassica* hexaploid library reads to the *A. thaliana* reference genome (ftp://ftp.arabidopsis.org/Sequences/whole_chromosomes). The total mapped reads accounted for only 21.10% of the library reads, with 17.77% uniquely matched reads. The match scores of the *A. thaliana* genome were too low for transcriptome analysis (data unpublished). We then used the *B* *. rapa* genome as a reference, and the uniquely matched reads accounted for 70.35%, 46.20%, and 54.18% in the *B* *. rapa* , *B* *. carinata* , and *Brassica* hexaploid libraries, respectively. As the match scores fit the technological criterion, the *B* *. rapa* genome could be applied as a reference. A total of 6.26–7.43% of the multiply mapped reads in our sequencing data remains unanalyzed; however, most of these might have been generated from duplicated genes and chromosome segments. A total of 23.39–46.37% of the reads could not be matched to the *B* *. rapa* genome. This relatively low percentage may be attributable to the inclusion of species different from *B* *. rapa* , unavoidable errors in the reference genome, or the specific mapping criteria. Nevertheless, the numerous genes and DEGs detected in *Brassica* hexaploid and its parents provide valuable information for using synthesized *Brassica* hexaploid as a model to explore changes in gene expression.

### Parental-biased gene expression and non-additive gene expression in *Brassica* hexaploid

The parental-biased changes in gene expression and DNA methylation between synthesized *B. napus* and its parents, as well as between newly synthesized *Brassica* allohexaploid and its parents, were explored using cDNA-AFLP and MSAP [36,47]. To explore parental-biased gene expression in the allohexaploid, we compared the number and fold-change of DEGs between *Brassica* hexaploid and its maternal parent *B* *. carinata* and paternal parent *B* *. rapa* , respectively. Of the 7,397 DEGs identified between *Brassica* hexaploid and its parents, a total of 5,248 DEGs (71%) were differently expressed between *Brassica* hexaploid and *B* *. rapa* , and 3,644 DEGs (about 50%) showed differential expression between *Brassica* hexaploid and *B* *. carinata* .

To analyze the magnitudes of the differences in expression, we first divided the absolute difference between the RPKM value in *Brassica* hexaploid and the RPKM value in *B* *. rapa* by the RPKM value in *Brassica* hexaploid for each DEG. Similarly, we divided the absolute difference between the RPKM values in *Brassica* hexaploid and *B* *. carinata* by the RPKM value in *Brassica* hexaploid for each DEG. We then compared the normalized values for each gene by subtracting the later from the former, such that a positive final value indicated that the magnitude of the difference in the expression of the gene between *Brassica* hexaploid and *B* *. rapa* was larger than that between *Brassica* hexaploid and *B* *. carinata* . A negative value indicated the converse. Of the 7,397 DEGs, 4,425 DEGs (~60%) had a positive value and 2,854 DEGs (~39%) had a negative value. The remaining 52 DEGs (~1%) had a final value of zero. As there were more DEGs with a larger difference in expression between *Brassica* hexaploid and *B* *. rapa* than between *Brassica* hexaploid and *B* *. carinata* , it is possible that there were directional gene expression changes away from the paternal parent in *Brassica* hexaploid.

Our results are in accordance with the results of Xu et al. [36,47], which also showed larger gene expression differences between polyploids and their paternal parent. However, for more than 60% of the proteins in allotetraploid *B. napus*, the expression patterns were closer to those of its paternal parent, *B* *. rapa* [48]. Apart from differences generated by the divergence of experimental materials, it is also conceivable that changes at the transcriptomic level may not agree with changes at the proteomic level. The paternal-biased phenomenon has been shown in other polyploids. For example, Adams et al. [17,21] observed silencing and biased expression of homologous gene pairs in natural and synthetic tetraploid cotton, and noted that gene silencing in the newly synthesized allotetraploid cotton was mainly epigenetic. One possible explanation for paternal-biased changes in allopolyploids is cytoplasmic and maternal effects. Cytoplasm-nuclear interactions could have influenced the parental genomes, resulting in differences in the extent and direction of genomic and transcriptomic changes.

Nonadditive gene expression in polyploidy has been observed in *Arabidopsis* allotetraploids, hexaploid wheat, and citrus allotetraploid hybrids [18,49,50]. The percentage of nonadditive genes in *Brassica* hexaploid was lower than that in hexaploid wheat and higher than that in *Arabidopsis* allotetraploids [18,49]. Because RNA-Seq technology is much more precise than microarrays, we could use restrictive criteria to select nonadditive genes. The percentage of nonadditive genes in *Brassica* hexaploid would increase if less restrictive criteria were used. There were no significant relationships between the nonadditive genes in citrus allotetraploids and the functional categories of genes [50]. However, significant numbers of nonadditive genes in *Arabidopsis* allotetraploids belonged to the hormonal regulation, cell defense, and aging categories [18]. Compared with the additive genes in *Brassica* hexaploid, the nonadditive genes were significantly enriched in biological processes related to stimulus response, immune system, cellular metabolic, rhythmic process, developmental process, pigmentation, seed germination, and tropism, and in the molecular functions associated with catalytic activity, antioxidant activity, and transcription regulator activity.

The high frequency of changes in gene expression in the present study was presumably attributable in part to the higher ploidy level in *Brassica* hexaploid. The diversity of the genomes in allohexaploids may decrease the genomic stability compared with that in allotetraploids. Therefore, adjustments in gene expression are necessary for the adaptation of allohexaploids to the environment. The *Brassica* hexaploid can also be considered as a secondary polyploidy derivative of tetraploid *B* *. carinata* , and the conflict between tetraploid and diploid genomes may lead to more severe changes in the hexaploid transcriptome. We hypothesized that the natural tetraploid *B* *. carinata* used in our experiment had a lesser impact on the transcriptome when hybridized with the diploid *B* *. rapa* (for it has previously undergone allopolyploidization), and that the genes in *B* *. carinata* had entered a stabilized state.

### Putative transcription factor and methylase genes may be important in allohexaploid evolution

Transcription factors regulate the expression of many downstream genes and have critical roles in plant development and adaptation. In synthesized *Arabidopsis* allotetraploids, the regulation of genome-wide non-additive gene expression may be partly controlled by transcription factors [18]. Our results provide a comprehensive view of transcription factor gene families. We examined several multi-gene families to identify their expression profiles and functions in plants. As studies on the TCP gene family increase, their importance in the evolution and developmental control of plant forms is being elucidated [51]. One member of the TCP gene family, *teosinte branched 1*, determines the strong apical dominance in domesticated maize [52]. Another gene, *CYCLOIDEA* from snapdragon, controls floral bilateral symmetry [53]. *PROLIFERATING CELL* FACTORS *1* and *2* from rice are involved in DNA replication and repair, maintenance of chromatin structure, chromosome segregation, and cell-cycle progression [54]. We detected five TCP genes with significant differential expression between *Brassica* hexaploid and its parents; three were upregulated and two were downregulated. Meanwhile, one-third of the putative target genes of TCP genes were differentially expressed between the hexaploid and its parents. Thus, direct or indirect regulation of the interactions between TCP genes and their target genes may occur. The synergistic actions between members of the TCP gene family and their target genes may contribute to the morphological evolution of polyploids.

Auxin response factors (ARF) are transcription factors that control plant growth and development by regulating the expression of auxin response genes [55]. We detected two ARF genes (Bra007127 and Bra009079), and their expression levels did not differ significantly between *Brassica* hexaploid and its parents. This suggests that the morphological differences that develop between *Brassica* hexaploid and its parents may be determined by genes of multiple functional groups, excluding auxin-related genes.

DNA methylation is an important epigenetic factor for transcriptomic changes in allopolyploids, and changes in DNA methylation and their relationship with gene expression in allopolyploids have been widely studied. Alterations in cytosine methylation affected both repetitive DNA sequences and low-copy DNA in wheat allotetraploids [56]. Significant changes in methylation patterns were observed in *Spartina* allopolyploids and may explain the morphological plasticity and larger ecological amplitude of allopolyploids [25]. In a newly synthesized *Brassica* tetraploid line, *B. napus*, extensively altered DNA methylation was detected using restriction fragment length polymorphism probes [57]. Hegarty et al. [58] observed methylation state changes, which mirrored gene expression changes, in triploid *Senecio* hybrids and their allohexaploid derivatives. In our results, 47 of the 157 putative methyltransferase genes and 4 of the 11 putative methylase genes showed significant differential expression between *Brassica* hexaploid and its parents. Among the differentially expressed genes, 40 were upregulated and 11 were downregulated. Gene methylation leads to gene inactivation; thus, the upregulation of methylation-related genes may be associated with the lower expression of some DEGs in the hexaploid compared with its parents, and the downregulation of methyltransferase and methylation genes may influence the increased expression of some DEGs. Although the methylation states of genes were not examined in our study, altered expression of DNA methylation-related genes provides indirect evidence for the function of DNA methylation in transcriptome changes during allopolyploid evolution.

## Conclusions

This study identified genes expressed in *Brassica* hexaploid and its parents, detected genes differentially expressed between the hexaploid and its parents, and classified these genes according to different functional categories. The *Brassica* hexaploid showed more differences in gene expression compared with its paternal parent than compared with its maternal parent, which supports the paternal-biased changes suggested in many previous allopolyploid studies. The DEGs detected between the hexaploid and its parents revealed that the adaptation and evolution processes of polyploids involve genome-wide changes in gene expression. Many nonadditive genes associated with important biological processes were identified in *Brassica* hexaploid. We speculate that transcriptomic changes are more severe in allohexaploids because of their higher ploidy level and the processes of secondary polyploidization. Changes in the transcriptomes of newly formed allopolyploids provide more variations for selection and facilitate allopolyploid survival and evolution. Further studies on gene regulation are required to determine the molecular mechanisms behind the success of allopolyploids in nature.

## Supporting Information

Table S1 A list of KEGG pathways mapped by 21092 genes in our result. Pathways over-represented by genes were for metabolic pathways, biosynthesis of secondary metabolites, plant–pathogen interaction, and plant hormone signal transduction with 4529, 2547, 1867, and 1684 members respectively.
(XLS)

Table S2 A set of 5248 differentially expressed genes between *Brassica* hexaploid and *B* *. rapa* . FDR: false discovery rate. We screened genes with expression change no less than two-fold and FDR no more than 0.001 as differentially expressed genes. There were 2670 up-regulated genes and 2578 down-regulated genes compared *Brassica* hexaploid (C) to *B* *. rapa* (A).
(DOC)

Table S3 A set of 3644 differentially expressed genes between *Brassica* hexaploid and *B* *. carinata* . FDR: false discovery rate. We screened genes with expression change no less than two-fold and FDR no more than 0.001 as differentially expressed genes. There were 2274 up-regulated genes and 1370 down-regulated genes compared *Brassica* hexaploid (C) to *B* *. carinata* (B).
(DOC)

Table S4 A list of genes in cluster 1, 2, 3, and 4, respectively. For the 1392 DEGs significantly differential expressed in both comparisons of *Brassica* hexaploid (C)- *B* *. rapa* (A) and *Brassica* hexaploid (C)- *B* *. carinata* (B), we classified them into four expression patterns by two-dimensional hierarchical clustering. There were 319, 218, 187, and 668 genes in cluster 1, 2, 3, and 4, respectively.
(XLS)

Table S5 A list of genes in group 1, 2, 3, and 4, respectively. For the 7397 DEGs significantly differential expressed in either comparison of *Brassica* hexaploid (C)- *B* *. rapa* (A) and *Brassica* hexaploid (C)- *B* *. carinata* (B), we classified them into four groups. Genes in group 1, 2, 3, and 4 had the same expression profiles as genes in cluster 1, 2, 3, and 4. There were 2016, 1701, 1327, and 2353 genes in group 1, 2, 3, and 4 respectively.
(XLS)

Table S6 A list of putative transcription factors (TFs) in our result. A, *B* *. rapa* ; B, *B* *. carinata* ; C, *Brassica* hexaploid. We queried *B* *. rapa* transcription factors genes in the Plant Transcription Factor Database (http://planttfdb.cbi.edu.cn/) and identified 589 putative TF genes, which classified into 49 families. Of the 589 putative TFs, 143 genes belonged to 36 families which showed significantly differential expression between *Brassica* hexaploid and its parents are marked by *.
(XLS)

Table S7 **A list of the putative methyltransferase and methylase genes in our result.**
A, *B* *. rapa* ; B, *B* *. carinata* ; C, *Brassica* hexaploid. We screened 157 putative methyltransferase genes and 11 putative methylase genes with one gene (Bra035280) annotated by both methyltransferase gene and methylase gene. The differentially expressed genes between them are marked by *.
(XLS)
