# Supplementary material for: Tracing the Transcriptomic Changes in Synthetic Trigenomic allohexaploids of Brassica Using an RNA-Seq Approach
Source: PLoS One. 2013 Jul 11;8(7):e68883. doi: 10.1371/journal.pone.0068883 (PMC3708896; doi:10.1371/journal.pone.0068883)
Supplement: Table S2 — FDR: false discovery rate. We screened genes with expression change no less than two-fold and FDR no more than 0.001 as differentially expressed genes. There were 2670 up-regulated genes and 2578 down-regulated genes compared Brassica hexaploid (C) to B . rapa (A). (DOC) [file pone.0068883.s002.doc]

**A set of 5248 differentially expressed genes between *Brassica* hexaploid and *B. rapa***

FDR: false discovery rate. We screened genes with expression change no less than two-fold and FDR no more than 0.001 as differentially expressed genes. There were 2670 up-regulated genes and 2578 down-regulated genes compared *Brassica* hexaploid (C) to *B. rapa* (A).

| ***GeneID*** | ***Gene length (bp)*** | ***Up-Down-Regulation (C/A)*** | ***FDR*** |
| --- | --- | --- | --- |
| Bra039597 | 390 | Down | 3.64E-15 |
| Bra040764 | 288 | Down | 3.66E-15 |
| Bra008055 | 1605 | Down | 3.63E-15 |
| Bra021959 | 414 | Down | 3.65E-15 |
| Bra035069 | 354 | Down | 3.63E-15 |
| Bra026913 | 1140 | Down | 3.62E-15 |
| Bra008697 | 543 | Down | 3.62E-15 |
| Bra016247 | 921 | Down | 3.65E-15 |
| Bra006468 | 1344 | Down | 3.66E-15 |
| Bra023716 | 402 | Down | 3.64E-15 |
| Bra029310 | 234 | Down | 7.44E-11 |
| Bra008053 | 2601 | Down | 3.66E-15 |
| Bra010158 | 264 | Down | 9.50E-12 |
| Bra010724 | 2589 | Down | 3.65E-15 |
| Bra005333 | 267 | Down | 2.91E-09 |
| Bra033829 | 1473 | Down | 3.63E-15 |
| Bra027133 | 297 | Down | 1.02E-09 |
| Bra015648 | 360 | Down | 7.45E-11 |
| Bra006844 | 693 | Down | 3.62E-15 |
| Bra031678 | 483 | Down | 5.47E-14 |
| Bra038180 | 354 | Down | 6.06E-10 |
| Bra024033 | 1185 | Down | 3.65E-15 |
| Bra026629 | 483 | Down | 3.50E-12 |
| Bra005649 | 396 | Down | 6.06E-10 |
| Bra019515 | 297 | Down | 3.15E-07 |
| Bra038196 | 345 | Down | 1.88E-07 |
| Bra038480 | 501 | Down | 2.11E-10 |
| Bra037598 | 195 | Down | 0.000403313 |
| Bra000941 | 231 | Down | 8.94E-05 |
| Bra030686 | 327 | Down | 1.49E-06 |
| Bra035102 | 237 | Down | 8.95E-05 |
| Bra019262 | 348 | Down | 8.87E-07 |
| Bra002037 | 495 | Down | 4.91E-09 |
| Bra025871 | 1047 | Down | 3.64E-15 |
| Bra014327 | 477 | Down | 2.35E-08 |
| Bra019635 | 447 | Down | 1.12E-07 |
| Bra035007 | 846 | Down | 9.46E-14 |
| Bra029494 | 357 | Down | 6.96E-06 |
| Bra031312 | 240 | Down | 0.000666957 |
| Bra012687 | 753 | Down | 4.44E-11 |
| Bra027056 | 810 | Down | 9.50E-12 |
| Bra012702 | 1518 | Down | 3.64E-15 |
| Bra028797 | 1611 | Down | 3.63E-15 |
| Bra024962 | 282 | Down | 0.000666576 |
| Bra028286 | 1749 | Down | 3.64E-15 |
| Bra008056 | 1554 | Down | 3.64E-15 |
| Bra013396 | 504 | Down | 4.16E-06 |
| Bra030411 | 2157 | Down | 3.66E-15 |
| Bra031149 | 348 | Down | 0.000402928 |
| Bra015744 | 417 | Down | 8.94E-05 |
| Bra030542 | 1200 | Down | 4.75E-13 |
| Bra009677 | 642 | Down | 5.28E-07 |
| Bra031822 | 348 | Down | 0.000666424 |
| Bra034235 | 1503 | Down | 3.63E-15 |
| Bra018943 | 606 | Down | 2.49E-06 |
| Bra039933 | 714 | Down | 3.15E-07 |
| Bra020356 | 675 | Down | 8.87E-07 |
| Bra015085 | 840 | Down | 2.36E-08 |
| Bra023961 | 540 | Down | 3.23E-05 |
| Bra039963 | 1911 | Down | 3.65E-15 |
| Bra029897 | 1335 | Down | 3.50E-12 |
| Bra040560 | 1260 | Down | 2.65E-11 |
| Bra001383 | 1248 | Down | 4.44E-11 |
| Bra035061 | 948 | Down | 6.69E-08 |
| Bra023541 | 948 | Down | 6.69E-08 |
| Bra003955 | 474 | Down | 0.000666805 |
| Bra013459 | 594 | Down | 8.95E-05 |
| Bra039930 | 1497 | Down | 7.44E-11 |
| Bra024590 | 1053 | Down | 1.12E-07 |
| Bra026175 | 750 | Down | 1.94E-05 |
| Bra015629 | 963 | Down | 8.86E-07 |
| Bra020505 | 1002 | Down | 5.28E-07 |
| Bra016858 | 549 | Down | 0.0006665 |
| Bra034227 | 957 | Down | 2.49E-06 |
| Bra012295 | 678 | Down | 0.000148103 |
| Bra036242 | 1170 | Down | 1.88E-07 |
| Bra019719 | 1146 | Down | 3.15E-07 |
| Bra034236 | 2112 | Down | 7.84E-13 |
| Bra025824 | 699 | Down | 0.000245232 |
| Bra012297 | 1092 | Down | 4.16E-06 |
| Bra017994 | 2487 | Down | 1.65E-13 |
| Bra015591 | 2316 | Down | 2.16E-12 |
| Bra014620 | 1377 | Down | 1.88E-07 |
| Bra032046 | 921 | Down | 5.36E-05 |
| Bra001597 | 1053 | Down | 1.17E-05 |
| Bra030904 | 1713 | Down | 8.33E-09 |
| Bra015244 | 792 | Down | 0.000402976 |
| Bra037230 | 1326 | Down | 1.49E-06 |
| Bra006996 | 1116 | Down | 1.94E-05 |
| Bra001386 | 1155 | Down | 1.94E-05 |
| Bra007843 | 1119 | Down | 3.23E-05 |
| Bra021821 | 1530 | Down | 8.87E-07 |
| Bra013147 | 1137 | Down | 5.36E-05 |
| Bra019921 | 1722 | Down | 3.15E-07 |
| Bra012628 | 1338 | Down | 1.17E-05 |
| Bra026223 | 2190 | Down | 4.91E-09 |
| Bra039498 | 1500 | Down | 4.16E-06 |
| Bra031275 | 999 | Down | 0.000403217 |
| Bra029716 | 1062 | Down | 0.000245263 |
| Bra014193 | 2184 | Down | 1.40E-08 |
| Bra040463 | 1206 | Down | 8.95E-05 |
| Bra021523 | 1050 | Down | 0.000403265 |
| Bra030351 | 1368 | Down | 5.36E-05 |
| Bra029236 | 1716 | Down | 6.96E-06 |
| Bra031581 | 2007 | Down | 8.86E-07 |
| Bra000109 | 1476 | Down | 5.36E-05 |
| Bra012974 | 1626 | Down | 1.94E-05 |
| Bra013383 | 1365 | Down | 0.000148084 |
| Bra008323 | 1305 | Down | 0.000245325 |
| Bra019914 | 1254 | Down | 0.000403072 |
| Bra015204 | 3027 | Down | 1.73E-09 |
| Bra016021 | 1656 | Down | 3.23E-05 |
| Bra011055 | 1281 | Down | 0.000403024 |
| Bra015627 | 2253 | Down | 8.87E-07 |
| Bra009784 | 2424 | Down | 3.15E-07 |
| Bra023882 | 1338 | Down | 0.000403169 |
| Bra033932 | 2367 | Down | 8.87E-07 |
| Bra016211 | 1392 | Down | 0.000403121 |
| Bra022128 | 1494 | Down | 0.000245294 |
| Bra014676 | 1641 | Down | 0.000148065 |
| Bra027534 | 3675 | Down | 4.91E-09 |
| Bra018945 | 1584 | Down | 0.000666729 |
| Bra023494 | 1635 | Down | 0.000666652 |
| Bra021295 | 1674 | Down | 0.000666881 |
| Bra005521 | 2604 | Down | 1.17E-05 |
| Bra014035 | 6240 | Down | 8.95E-05 |
| Bra039592 | 261 | Down | 0.000319061 |
| Bra038094 | 1293 | Down | 3.34E-06 |
| Bra028706 | 1140 | Down | 0.000319334 |
| Bra007228 | 474 | Down | 0.000177089 |
| Bra003998 | 720 | Down | 5.17E-05 |
| Bra001820 | 1758 | Down | 0.000319491 |
| Bra033731 | 1137 | Down | 2.68E-05 |
| Bra010212 | 351 | Down | 0.000319178 |
| Bra015721 | 519 | Down | 1.36E-05 |
| Bra015997 | 615 | Down | 0.000319373 |
| Bra021082 | 1641 | Down | 7.85E-07 |
| Bra007927 | 447 | Down | 0.000319569 |
| Bra037613 | 1665 | Down | 0.000319256 |
| Bra022469 | 1149 | Down | 0.000176929 |
| Bra034221 | 765 | Down | 0.000176883 |
| Bra015767 | 777 | Down | 2.68E-05 |
| Bra008163 | 1551 | Down | 7.86E-07 |
| Bra039499 | 894 | Down | 6.79E-06 |
| Bra011332 | 696 | Down | 0.000319295 |
| Bra025954 | 555 | Down | 0.000319412 |
| Bra028951 | 468 | Down | 0.000319608 |
| Bra019485 | 1011 | Down | 0.0003191 |
| Bra035101 | 1128 | Down | 0.00017702 |
| Bra009276 | 2178 | Down | 0.00031953 |
| Bra015797 | 1467 | Down | 8.74E-09 |
| Bra005755 | 1074 | Down | 9.75E-05 |
| Bra014464 | 282 | Down | 0.000319451 |
| Bra001826 | 975 | Down | 0.000319217 |
| Bra022584 | 1719 | Down | 0.000176975 |
| Bra028865 | 1083 | Down | 8.45E-08 |
| Bra004483 | 1428 | Down | 0.000177066 |
| Bra024350 | 591 | Down | 0.000319139 |
| Bra020644 | 477 | Down | 5.17E-05 |
| Bra009773 | 924 | Down | 9.74E-05 |
| Bra015865 | 1041 | Down | 9.74E-05 |
| Bra007184 | 1350 | Down | 6.79E-06 |
| Bra020488 | 1491 | Down | 3.34E-06 |
| Bra018572 | 618 | Down | 2.68E-05 |
| Bra016893 | 1809 | Down | 0.000319023 |
| Bra033792 | 1782 | Down | 0.000318984 |
| Bra030376 | 351 | Down | 9.75E-05 |
| Bra038348 | 1233 | Down | 5.17E-05 |
| Bra011177 | 1569 | Down | 0.000177043 |
| Bra009406 | 1923 | Down | 0.000318951 |
| Bra026978 | 2673 | Down | 0.000176906 |
| Bra022907 | 4611 | Down | 0.000318917 |
| Bra029377 | 1281 | Down | 0.000318878 |
| Bra016220 | 1779 | Down | 9.75E-05 |
| Bra029212 | 1269 | Down | 1.63E-06 |
| Bra012184 | 1182 | Down | 5.17E-05 |
| Bra024939 | 1086 | Down | 1.79E-07 |
| Bra020640 | 774 | Down | 0.000177112 |
| Bra037668 | 249 | Down | 0.000176997 |
| Bra020465 | 1281 | Down | 1.36E-05 |
| Bra037509 | 2595 | Down | 0.000176952 |
| Bra012458 | 591 | Down | 5.17E-05 |
| Bra014527 | 1182 | Down | 0.000318992 |
| Bra007660 | 1269 | Down | 4.05E-09 |
| Bra004004 | 1041 | Down | 9.74E-05 |
| Bra021866 | 1683 | Down | 0.000319073 |
| Bra017698 | 888 | Down | 0.000319112 |
| Bra033315 | 705 | Down | 1.16E-11 |
| Bra034652 | 741 | Down | 3.34E-06 |
| Bra028978 | 1533 | Down | 0.000319295 |
| Bra005516 | 1593 | Down | 0.000319256 |
| Bra023019 | 1860 | Down | 0.000319217 |
| Bra039070 | 798 | Down | 5.98E-12 |
| Bra009569 | 1092 | Down | 1.79E-07 |
| Bra011273 | 936 | Down | 2.68E-05 |
| Bra018506 | 1509 | Down | 5.17E-05 |
| Bra026143 | 1047 | Down | 2.68E-05 |
| Bra026104 | 600 | Down | 0.000320231 |
| Bra001405 | 1152 | Down | 0.000176862 |
| Bra017641 | 1449 | Down | 3.98E-08 |
| Bra036946 | 882 | Down | 9.75E-05 |
| Bra022943 | 534 | Down | 2.68E-05 |
| Bra005436 | 384 | Down | 0.000321186 |
| Bra025261 | 846 | Down | 2.68E-05 |
| Bra019741 | 429 | Down | 1.31E-12 |
| Bra020120 | 456 | Down | 0.000176845 |
| Bra023877 | 675 | Down | 1.79E-07 |
| Bra039969 | 792 | Down | 0.000322912 |
| Bra011481 | 1089 | Down | 9.75E-05 |
| Bra003748 | 960 | Down | 8.45E-08 |
| Bra025485 | 4854 | Down | 8.78E-10 |
| Bra018164 | 3069 | Down | 0.000325709 |
| Bra013495 | 291 | Down | 0.000325749 |
| Bra032705 | 2139 | Down | 9.74E-05 |
| Bra000405 | 1158 | Down | 9.74E-05 |
| Bra013769 | 912 | Down | 0.000330241 |
| Bra013738 | 354 | Down | 6.79E-06 |
| Bra005922 | 1320 | Down | 1.79E-07 |
| Bra040047 | 1587 | Down | 0.00017687 |
| Bra019537 | 1551 | Down | 9.74E-05 |
| Bra040268 | 1089 | Down | 1.63E-06 |
| Bra000758 | 3756 | Down | 0.00035038 |
| Bra038748 | 1479 | Down | 0.000350337 |
| Bra023881 | 1737 | Down | 9.74E-05 |
| Bra006296 | 981 | Down | 1.63E-06 |
| Bra034525 | 1818 | Down | 0.000370297 |
| Bra002131 | 300 | Down | 0.00017718 |
| Bra019305 | 2700 | Down | 9.74E-05 |
| Bra002594 | 1239 | Down | 9.73E-05 |
| Bra023777 | 1245 | Down | 4.09E-10 |
| Bra026368 | 2934 | Down | 0 |
| Bra040489 | 885 | Down | 0.000401109 |
| Bra011885 | 2382 | Down | 1.63E-06 |
| Bra017218 | 873 | Down | 0.000401205 |
| Bra041153 | 639 | Down | 0.000401157 |
| Bra024204 | 1347 | Down | 0 |
| Bra016253 | 687 | Down | 2.68E-05 |
| Bra025117 | 915 | Down | 2.68E-05 |
| Bra017465 | 447 | Down | 7.85E-07 |
| Bra025829 | 1548 | Down | 5.17E-05 |
| Bra021809 | 405 | Down | 2.19E-11 |
| Bra000276 | 351 | Down | 9.73E-05 |
| Bra024395 | 1356 | Down | 1.36E-05 |
| Bra025545 | 834 | Down | 0.000177787 |
| Bra006910 | 2193 | Down | 3.34E-06 |
| Bra034137 | 1338 | Down | 0.000452161 |
| Bra027529 | 972 | Down | 0.000452322 |
| Bra041093 | 510 | Down | 0.000452214 |
| Bra011292 | 1122 | Down | 0.000452268 |
| Bra019232 | 384 | Down | 0.000452375 |
| Bra037350 | 1053 | Down | 5.17E-05 |
| Bra005558 | 309 | Down | 0.000178392 |
| Bra033577 | 1833 | Down | 8.46E-08 |
| Bra012220 | 2766 | Down | 8.45E-08 |
| Bra003483 | 924 | Down | 3.75E-07 |
| Bra025785 | 162 | Down | 2.68E-05 |
| Bra009620 | 519 | Down | 3.75E-07 |
| Bra004001 | 966 | Down | 9.73E-05 |
| Bra025061 | 2253 | Down | 0.000533404 |
| Bra016205 | 1842 | Down | 0.000533466 |
| Bra006799 | 1683 | Down | 7.85E-07 |
| Bra023115 | 867 | Down | 0.00066019 |
| Bra024637 | 1227 | Down | 9.74E-05 |
| Bra012118 | 795 | Down | 0.000660342 |
| Bra025268 | 1695 | Down | 0.000660266 |
| Bra032746 | 2589 | Down | 6.79E-06 |
| Bra020390 | 1872 | Down | 9.74E-05 |
| Bra009785 | 1518 | Down | 9.72E-13 |
| Bra009285 | 1140 | Down | 1.91E-10 |
| Bra015829 | 3417 | Down | 0.000183184 |
| Bra030132 | 360 | Down | 0.00018316 |
| Bra034700 | 318 | Down | 1.63E-06 |
| Bra013343 | 678 | Down | 1.87E-08 |
| Bra017242 | 1167 | Down | 9.74E-05 |
| Bra037020 | 1512 | Down | 6.79E-06 |
| Bra001043 | 687 | Down | 9.74E-05 |
| Bra006905 | 330 | Down | 9.06E-11 |
| Bra001882 | 486 | Down | 0.00018672 |
| Bra012065 | 879 | Down | 0.000860426 |
| Bra016048 | 399 | Down | 0.00086033 |
| Bra029138 | 330 | Down | 0.000186768 |
| Bra024161 | 1167 | Down | 0.000186744 |
| Bra030412 | 2673 | Down | 0.000860523 |
| Bra001349 | 1752 | Down | 1.36E-05 |
| Bra008288 | 2847 | Down | 0.000192877 |
| Bra029066 | 1800 | Down | 9.77E-05 |
| Bra030899 | 756 | Down | 9.77E-05 |
| Bra027796 | 1821 | Down | 2.68E-05 |
| Bra015904 | 540 | Down | 1.36E-05 |
| Bra023927 | 1332 | Down | 4.05E-09 |
| Bra036852 | 660 | Down | 9.80E-05 |
| Bra030715 | 324 | Down | 5.17E-05 |
| Bra032917 | 1170 | Down | 1.16E-11 |
| Bra015809 | 240 | Down | 6.51E-13 |
| Bra004432 | 1833 | Down | 0.000217993 |
| Bra021343 | 681 | Down | 6.79E-06 |
| Bra030656 | 1086 | Down | 2.68E-05 |
| Bra023914 | 426 | Down | 9.85E-05 |
| Bra003925 | 2418 | Down | 8.49E-13 |
| Bra040701 | 978 | Down | 1.36E-05 |
| Bra005747 | 1089 | Down | 2.68E-05 |
| Bra000932 | 876 | Down | 5.17E-05 |
| Bra013892 | 342 | Down | 5.17E-05 |
| Bra037371 | 1602 | Down | 9.92E-05 |
| Bra002340 | 2550 | Down | 6.79E-06 |
| Bra001782 | 1269 | Down | 9.91E-05 |
| Bra011052 | 339 | Down | 3.34E-06 |
| Bra017859 | 1899 | Down | 2.68E-05 |
| Bra040654 | 279 | Down | 2.68E-05 |
| Bra039649 | 759 | Down | 0.000242516 |
| Bra011456 | 591 | Down | 0.000242486 |
| Bra034887 | 840 | Down | 0.000242547 |
| Bra016178 | 477 | Down | 5.18E-05 |
| Bra021483 | 1014 | Down | 1.36E-05 |
| Bra008160 | 1020 | Down | 4.12E-13 |
| Bra039226 | 1731 | Down | 6.79E-06 |
| Bra040092 | 546 | Down | 0.000100336 |
| Bra024804 | 1380 | Down | 0 |
| Bra012880 | 1227 | Down | 4.05E-09 |
| Bra035205 | 939 | Down | 5.18E-05 |
| Bra024434 | 498 | Down | 0.000279181 |
| Bra025270 | 762 | Down | 2.68E-05 |
| Bra002696 | 222 | Down | 0.000279147 |
| Bra022655 | 825 | Down | 1.24E-12 |
| Bra021939 | 1872 | Down | 0.000101896 |
| Bra031854 | 1404 | Down | 5.19E-05 |
| Bra023573 | 1071 | Down | 4.73E-12 |
| Bra020971 | 1356 | Down | 3.75E-07 |
| Bra027612 | 558 | Down | 0.000104881 |
| Bra039324 | 642 | Down | 0 |
| Bra030873 | 594 | Down | 1.87E-08 |
| Bra030562 | 1452 | Down | 5.20E-05 |
| Bra030780 | 924 | Down | 0.000104867 |
| Bra016611 | 2049 | Down | 2.68E-05 |
| Bra003351 | 786 | Down | 0.000104839 |
| Bra003769 | 1833 | Down | 0.000104853 |
| Bra012066 | 603 | Down | 1.36E-05 |
| Bra025753 | 1179 | Down | 1.36E-05 |
| Bra026924 | 2544 | Down | 4.09E-10 |
| Bra037491 | 2424 | Down | 2.19E-11 |
| Bra027026 | 1572 | Down | 3.34E-06 |
| Bra010524 | 966 | Down | 1.79E-07 |
| Bra002404 | 1455 | Down | 5.22E-05 |
| Bra036708 | 432 | Down | 0.000109443 |
| Bra032823 | 987 | Down | 3.75E-07 |
| Bra039088 | 1956 | Down | 6.78E-06 |
| Bra035172 | 1680 | Down | 6.78E-06 |
| Bra028870 | 225 | Down | 6.78E-06 |
| Bra005612 | 414 | Down | 1.88E-09 |
| Bra029936 | 2319 | Down | 3.64E-12 |
| Bra019013 | 831 | Down | 2.68E-05 |
| Bra020790 | 1542 | Down | 0.000427563 |
| Bra017339 | 1173 | Down | 0.000427512 |
| Bra002072 | 2307 | Down | 6.52E-13 |
| Bra038267 | 414 | Down | 4.09E-10 |
| Bra003953 | 1356 | Down | 1.36E-05 |
| Bra017801 | 1137 | Down | 8.44E-08 |
| Bra029318 | 2379 | Down | 1.79E-07 |
| Bra034189 | 909 | Down | 0.000116262 |
| Bra031280 | 510 | Down | 3.75E-07 |
| Bra028591 | 351 | Down | 0.000116278 |
| Bra023549 | 1032 | Down | 0 |
| Bra006736 | 378 | Down | 3.34E-06 |
| Bra034084 | 213 | Down | 1.36E-05 |
| Bra033843 | 1908 | Down | 1.63E-06 |
| Bra038402 | 954 | Down | 5.31E-05 |
| Bra005852 | 987 | Down | 5.31E-05 |
| Bra034243 | 1818 | Down | 6.78E-06 |
| Bra020698 | 519 | Down | 0.000566229 |
| Bra022673 | 1575 | Down | 0.000566164 |
| Bra038537 | 1431 | Down | 4.08E-10 |
| Bra000060 | 1431 | Down | 0 |
| Bra013306 | 306 | Down | 0.000127387 |
| Bra004681 | 1701 | Down | 0.00012742 |
| Bra005201 | 810 | Down | 0.000127403 |
| Bra015785 | 1284 | Down | 8.74E-09 |
| Bra025951 | 5358 | Down | 1.14E-12 |
| Bra004222 | 1800 | Down | 5.39E-05 |
| Bra041030 | 900 | Down | 1.91E-10 |
| Bra012479 | 867 | Down | 2.19E-11 |
| Bra025835 | 1146 | Down | 2.19E-11 |
| Bra015842 | 1575 | Down | 1.87E-08 |
| Bra023879 | 522 | Down | 5.52E-05 |
| Bra021328 | 1065 | Down | 7.85E-07 |
| Bra005792 | 198 | Down | 0.000144666 |
| Bra000134 | 1374 | Down | 5.52E-05 |
| Bra000057 | 1467 | Down | 8.45E-08 |
| Bra025891 | 423 | Down | 0.000144685 |
| Bra015835 | 966 | Down | 1.79E-07 |
| Bra005961 | 2178 | Down | 1.16E-11 |
| Bra024929 | 540 | Down | 3.34E-06 |
| Bra015303 | 1851 | Down | 3.34E-06 |
| Bra001520 | 1821 | Down | 2.19E-11 |
| Bra032345 | 300 | Down | 9.85E-12 |
| Bra005702 | 630 | Down | 0 |
| Bra011648 | 789 | Down | 5.73E-05 |
| Bra009200 | 804 | Down | 8.75E-09 |
| Bra000225 | 1206 | Down | 0.000171418 |
| Bra022163 | 1164 | Down | 0.000171396 |
| Bra000251 | 807 | Down | 7.85E-07 |
| Bra004489 | 1203 | Down | 0 |
| Bra000479 | 423 | Down | 2.74E-05 |
| Bra003658 | 1056 | Down | 3.75E-07 |
| Bra009625 | 267 | Down | 6.79E-06 |
| Bra018532 | 384 | Down | 0 |
| Bra026915 | 900 | Down | 4.75E-13 |
| Bra003655 | 678 | Down | 1.37E-05 |
| Bra000021 | 873 | Down | 7.85E-07 |
| Bra019245 | 474 | Down | 4.46E-14 |
| Bra011747 | 2286 | Down | 4.08E-10 |
| Bra019545 | 1149 | Down | 0 |
| Bra017045 | 1824 | Down | 0.000210752 |
| Bra033746 | 1239 | Down | 0 |
| Bra035015 | 666 | Down | 8.77E-10 |
| Bra005151 | 1680 | Down | 1.63E-06 |
| Bra030617 | 3417 | Down | 2.78E-05 |
| Bra005470 | 429 | Down | 8.45E-08 |
| Bra010750 | 2016 | Down | 3.75E-07 |
| Bra016277 | 1263 | Down | 6.54E-05 |
| Bra027407 | 807 | Down | 7.84E-07 |
| Bra034538 | 492 | Down | 7.85E-07 |
| Bra032768 | 2250 | Down | 3.34E-06 |
| Bra023519 | 1608 | Down | 3.63E-12 |
| Bra037308 | 4110 | Down | 9.05E-11 |
| Bra014666 | 396 | Down | 8.45E-08 |
| Bra032273 | 945 | Down | 0 |
| Bra009862 | 675 | Down | 7.84E-07 |
| Bra034814 | 450 | Down | 0.000270903 |
| Bra005280 | 2667 | Down | 0.000270936 |
| Bra024807 | 2223 | Down | 9.73E-13 |
| Bra025403 | 1371 | Down | 3.34E-06 |
| Bra001086 | 501 | Down | 9.72E-13 |
| Bra023219 | 1026 | Down | 7.30E-05 |
| Bra024456 | 1296 | Down | 2.93E-05 |
| Bra006298 | 1407 | Down | 2.93E-05 |
| Bra005913 | 2202 | Down | 2.93E-05 |
| Bra001231 | 645 | Down | 1.39E-05 |
| Bra013153 | 747 | Down | 7.84E-07 |
| Bra040647 | 1362 | Down | 0.000361871 |
| Bra012895 | 927 | Down | 0.000361828 |
| Bra001144 | 969 | Down | 0.00036174 |
| Bra037027 | 1068 | Down | 0.000361784 |
| Bra008205 | 528 | Down | 8.45E-05 |
| Bra016318 | 1467 | Down | 6.84E-06 |
| Bra037519 | 504 | Down | 6.84E-06 |
| Bra030615 | 1089 | Down | 0.000361697 |
| Bra006664 | 831 | Down | 8.78E-10 |
| Bra014417 | 687 | Down | 5.97E-13 |
| Bra035424 | 2703 | Down | 8.44E-08 |
| Bra016266 | 867 | Down | 1.79E-07 |
| Bra033671 | 1089 | Down | 3.74E-07 |
| Bra007644 | 840 | Down | 8.77E-10 |
| Bra021868 | 2475 | Down | 4.05E-09 |
| Bra022510 | 2406 | Down | 6.87E-06 |
| Bra024126 | 366 | Down | 0 |
| Bra006441 | 789 | Down | 1.16E-11 |
| Bra013576 | 996 | Down | 1.08E-10 |
| Bra010605 | 3093 | Down | 4.42E-11 |
| Bra037414 | 936 | Down | 2.85E-12 |
| Bra005432 | 1416 | Down | 1.24E-12 |
| Bra031492 | 1935 | Down | 6.91E-06 |
| Bra007377 | 1233 | Down | 0.000498318 |
| Bra009793 | 876 | Down | 0.00049826 |
| Bra028437 | 1203 | Down | 0.000498201 |
| Bra026669 | 2394 | Down | 0 |
| Bra030948 | 1767 | Down | 8.74E-09 |
| Bra015866 | 1917 | Down | 0 |
| Bra025307 | 3576 | Down | 1.79E-07 |
| Bra004928 | 642 | Down | 0 |
| Bra026725 | 300 | Down | 3.61E-05 |
| Bra021994 | 1236 | Down | 3.61E-05 |
| Bra026142 | 471 | Down | 3.36E-06 |
| Bra031111 | 1101 | Down | 1.16E-11 |
| Bra012870 | 750 | Down | 6.98E-06 |
| Bra006650 | 858 | Down | 0.000127467 |
| Bra008058 | 831 | Down | 0.00012745 |
| Bra014955 | 915 | Down | 2.23E-12 |
| Bra007149 | 288 | Down | 9.08E-13 |
| Bra014743 | 765 | Down | 1.43E-13 |
| Bra035647 | 1599 | Down | 4.11E-05 |
| Bra030618 | 1839 | Down | 4.11E-05 |
| Bra022433 | 1440 | Down | 7.09E-06 |
| Bra036247 | 501 | Down | 8.77E-10 |
| Bra010060 | 1038 | Down | 4.08E-10 |
| Bra027478 | 1173 | Down | 1.88E-09 |
| Bra006070 | 1488 | Down | 1.62E-05 |
| Bra025152 | 1488 | Down | 1.62E-05 |
| Bra018683 | 1620 | Down | 1.64E-06 |
| Bra032740 | 3057 | Down | 8.78E-10 |
| Bra034389 | 1356 | Down | 0.00016733 |
| Bra040364 | 5736 | Down | 1.64E-06 |
| Bra019298 | 1062 | Down | 3.98E-08 |
| Bra036210 | 768 | Down | 1.79E-07 |
| Bra034086 | 1116 | Down | 9.05E-11 |
| Bra010666 | 888 | Down | 4.05E-09 |
| Bra032045 | 3363 | Down | 4.85E-05 |
| Bra040117 | 4503 | Down | 1.79E-07 |
| Bra012830 | 228 | Down | 8.44E-08 |
| Bra011017 | 414 | Down | 1.76E-05 |
| Bra009030 | 3429 | Down | 1.76E-05 |
| Bra004593 | 744 | Down | 7.53E-06 |
| Bra004396 | 1107 | Down | 1.79E-07 |
| Bra018707 | 1419 | Down | 1.91E-10 |
| Bra034224 | 975 | Down | 3.98E-08 |
| Bra016854 | 351 | Down | 3.98E-08 |
| Bra012181 | 3033 | Down | 0.000225441 |
| Bra015896 | 1641 | Down | 3.98E-08 |
| Bra011408 | 432 | Down | 4.00E-13 |
| Bra005339 | 489 | Down | 1.87E-08 |
| Bra012594 | 2283 | Down | 5.94E-05 |
| Bra028838 | 816 | Down | 4.46E-14 |
| Bra010304 | 1053 | Down | 8.63E-13 |
| Bra007613 | 660 | Down | 1.97E-05 |
| Bra003347 | 2283 | Down | 7.93E-06 |
| Bra026785 | 843 | Down | 7.93E-06 |
| Bra020192 | 519 | Down | 1.26E-12 |
| Bra004519 | 849 | Down | 5.52E-13 |
| Bra030182 | 804 | Down | 0 |
| Bra026893 | 807 | Down | 3.76E-07 |
| Bra001575 | 2904 | Down | 4.05E-09 |
| Bra027378 | 1533 | Down | 1.47E-12 |
| Bra001790 | 2511 | Down | 3.98E-08 |
| Bra018443 | 1050 | Down | 3.98E-08 |
| Bra020284 | 339 | Down | 3.66E-06 |
| Bra016030 | 1131 | Down | 1.69E-06 |
| Bra004373 | 735 | Down | 2.29E-05 |
| Bra032690 | 1032 | Down | 7.95E-07 |
| Bra000970 | 1965 | Down | 0.000310921 |
| Bra010704 | 2574 | Down | 2.29E-05 |
| Bra016991 | 558 | Down | 7.62E-05 |
| Bra032889 | 1020 | Down | 1.21E-12 |
| Bra027363 | 687 | Down | 1.87E-08 |
| Bra024012 | 1887 | Down | 3.64E-12 |
| Bra009836 | 2799 | Down | 6.21E-12 |
| Bra031672 | 3366 | Down | 2.19E-11 |
| Bra009022 | 912 | Down | 5.98E-13 |
| Bra016094 | 660 | Down | 3.78E-07 |
| Bra016617 | 1611 | Down | 1.31E-12 |
| Bra029101 | 699 | Down | 3.63E-12 |
| Bra016206 | 1962 | Down | 3.83E-06 |
| Bra023397 | 1290 | Down | 9.44E-06 |
| Bra014673 | 1728 | Down | 2.76E-05 |
| Bra009841 | 1104 | Down | 4.53E-12 |
| Bra019209 | 1251 | Down | 0.000100655 |
| Bra000128 | 2385 | Down | 0.000100669 |
| Bra037002 | 930 | Down | 0.000100642 |
| Bra040516 | 348 | Down | 0.000100628 |
| Bra005278 | 1563 | Down | 3.79E-07 |
| Bra016916 | 2667 | Down | 2.75E-12 |
| Bra009849 | 522 | Down | 4.08E-10 |
| Bra005577 | 489 | Down | 0.000435795 |
| Bra037397 | 1539 | Down | 1.77E-06 |
| Bra007035 | 1749 | Down | 1.77E-06 |
| Bra015297 | 561 | Down | 0.000435847 |
| Bra016034 | 1455 | Down | 0 |
| Bra003732 | 585 | Down | 0 |
| Bra034136 | 2907 | Down | 1.88E-09 |
| Bra012074 | 3429 | Down | 0 |
| Bra022613 | 510 | Down | 9.72E-13 |
| Bra031724 | 1548 | Down | 1.16E-11 |
| Bra019567 | 1173 | Down | 3.47E-05 |
| Bra032819 | 408 | Down | 8.74E-09 |
| Bra007233 | 1437 | Down | 1.87E-08 |
| Bra011579 | 1146 | Down | 0.000137091 |
| Bra009279 | 1044 | Down | 0.000137073 |
| Bra005362 | 1230 | Down | 0.000137109 |
| Bra014119 | 540 | Down | 4.75E-13 |
| Bra012358 | 1812 | Down | 6.21E-12 |
| Bra035799 | 1293 | Down | 1.27E-05 |
| Bra016141 | 4449 | Down | 1.27E-05 |
| Bra037008 | 1110 | Down | 4.08E-10 |
| Bra037473 | 630 | Down | 1.82E-07 |
| Bra015252 | 234 | Down | 1.82E-07 |
| Bra024196 | 699 | Down | 1.95E-06 |
| Bra008335 | 783 | Down | 0.000621358 |
| Bra024988 | 1002 | Down | 0.000621286 |
| Bra022407 | 1362 | Down | 3.99E-08 |
| Bra025820 | 321 | Down | 0.000621215 |
| Bra020008 | 2265 | Down | 3.91E-07 |
| Bra014665 | 702 | Down | 0 |
| Bra024771 | 1152 | Down | 1.88E-08 |
| Bra035649 | 3231 | Down | 0 |
| Bra012138 | 1314 | Down | 1.47E-12 |
| Bra033076 | 708 | Down | 0.000190301 |
| Bra039670 | 345 | Down | 1.57E-05 |
| Bra005351 | 786 | Down | 1.57E-05 |
| Bra019793 | 696 | Down | 1.57E-05 |
| Bra020011 | 891 | Down | 4.08E-10 |
| Bra025179 | 1425 | Down | 0 |
| Bra033441 | 3399 | Down | 5.53E-13 |
| Bra036405 | 1386 | Down | 9.16E-07 |
| Bra024047 | 1896 | Down | 8.77E-10 |
| Bra000436 | 1215 | Down | 5.98E-13 |
| Bra014419 | 1668 | Down | 1.14E-12 |
| Bra005565 | 681 | Down | 6.02E-05 |
| Bra026366 | 1281 | Down | 6.02E-05 |
| Bra035019 | 948 | Down | 1.41E-14 |
| Bra025914 | 1005 | Down | 0.000891239 |
| Bra026069 | 1386 | Down | 0.000891438 |
| Bra010565 | 1764 | Down | 0.00089114 |
| Bra019060 | 1839 | Down | 0.000891339 |
| Bra009083 | 2367 | Down | 9.82E-07 |
| Bra004679 | 909 | Down | 6.52E-13 |
| Bra005501 | 720 | Down | 3.49E-13 |
| Bra031663 | 1011 | Down | 0 |
| Bra037001 | 966 | Down | 4.06E-09 |
| Bra013088 | 222 | Down | 0.000267292 |
| Bra035279 | 2298 | Down | 6.20E-12 |
| Bra004658 | 1215 | Down | 0.000267259 |
| Bra034711 | 1818 | Down | 4.06E-09 |
| Bra026686 | 1020 | Down | 4.31E-07 |
| Bra004983 | 2700 | Down | 6.51E-13 |
| Bra012714 | 1746 | Down | 2.69E-06 |
| Bra001974 | 1524 | Down | 6.71E-13 |
| Bra008019 | 540 | Down | 8.27E-05 |
| Bra024436 | 246 | Down | 8.28E-05 |
| Bra020489 | 1491 | Down | 0 |
| Bra017753 | 1455 | Down | 8.91E-13 |
| Bra010404 | 753 | Down | 1.08E-06 |
| Bra012972 | 2013 | Down | 1.91E-10 |
| Bra019009 | 453 | Down | 2.65E-05 |
| Bra031170 | 819 | Down | 2.65E-05 |
| Bra038583 | 1260 | Down | 2.65E-05 |
| Bra035036 | 1224 | Down | 2.75E-12 |
| Bra021570 | 1527 | Down | 5.44E-13 |
| Bra003917 | 1179 | Down | 4.08E-09 |
| Bra024833 | 1542 | Down | 2.20E-13 |
| Bra015711 | 1398 | Down | 0 |
| Bra035164 | 1275 | Down | 3.22E-06 |
| Bra008309 | 783 | Down | 1.92E-08 |
| Bra004984 | 1767 | Down | 1.92E-08 |
| Bra023465 | 1356 | Down | 0 |
| Bra038807 | 1794 | Down | 1.23E-06 |
| Bra031704 | 2919 | Down | 0.000379295 |
| Bra036790 | 3669 | Down | 5.01E-07 |
| Bra017712 | 1638 | Down | 5.01E-07 |
| Bra015659 | 873 | Down | 0.000379341 |
| Bra028729 | 2259 | Down | 0 |
| Bra016076 | 3375 | Down | 2.14E-07 |
| Bra016441 | 732 | Down | 1.26E-12 |
| Bra033526 | 1287 | Down | 0 |
| Bra028794 | 972 | Down | 0.00011471 |
| Bra012359 | 1896 | Down | 3.15E-12 |
| Bra036482 | 1305 | Down | 4.00E-13 |
| Bra011045 | 732 | Down | 8.82E-10 |
| Bra015954 | 2103 | Down | 0 |
| Bra038288 | 1410 | Down | 1.92E-10 |
| Bra017737 | 1251 | Down | 2.19E-11 |
| Bra021558 | 1797 | Down | 5.47E-14 |
| Bra026151 | 549 | Down | 1.91E-09 |
| Bra028352 | 252 | Down | 4.43E-11 |
| Bra016507 | 1182 | Down | 4.19E-09 |
| Bra022654 | 837 | Down | 4.11E-10 |
| Bra029827 | 720 | Down | 1.56E-05 |
| Bra028939 | 1350 | Down | 0.000541439 |
| Bra037172 | 636 | Down | 0.000162554 |
| Bra008216 | 456 | Down | 2.57E-07 |
| Bra009369 | 1275 | Down | 1.77E-06 |
| Bra016249 | 537 | Down | 4.96E-05 |
| Bra012422 | 597 | Down | 0.000541502 |
| Bra012658 | 609 | Down | 0 |
| Bra034002 | 711 | Down | 6.97E-13 |
| Bra019145 | 903 | Down | 9.09E-13 |
| Bra019081 | 1161 | Down | 2.95E-07 |
| Bra008655 | 1065 | Down | 1.85E-12 |
| Bra033517 | 1704 | Down | 0 |
| Bra037327 | 1404 | Down | 2.24E-06 |
| Bra037746 | 1497 | Down | 2.24E-06 |
| Bra040933 | 1821 | Down | 8.49E-13 |
| Bra000505 | 498 | Down | 6.76E-06 |
| Bra014098 | 648 | Down | 2.13E-05 |
| Bra020159 | 750 | Down | 2.13E-05 |
| Bra002454 | 2178 | Down | 2.13E-05 |
| Bra005729 | 996 | Down | 9.85E-09 |
| Bra036212 | 471 | Down | 6.92E-05 |
| Bra035131 | 1464 | Down | 6.91E-05 |
| Bra012997 | 726 | Down | 6.91E-05 |
| Bra015886 | 492 | Down | 3.88E-12 |
| Bra035646 | 1083 | Down | 5.39E-08 |
| Bra013665 | 6501 | Down | 1.34E-07 |
| Bra027537 | 1134 | Down | 0 |
| Bra019786 | 882 | Down | 0.000776644 |
| Bra039449 | 675 | Down | 0.000776556 |
| Bra015989 | 1224 | Down | 0.000776732 |
| Bra031919 | 579 | Down | 9.83E-07 |
| Bra025831 | 1233 | Down | 5.98E-13 |
| Bra007978 | 2430 | Down | 2.29E-13 |
| Bra022948 | 735 | Down | 2.94E-05 |
| Bra016316 | 1821 | Down | 2.94E-05 |
| Bra016950 | 1479 | Down | 5.97E-13 |
| Bra024186 | 1530 | Down | 4.31E-10 |
| Bra024069 | 1911 | Down | 2.75E-12 |
| Bra020310 | 1377 | Down | 9.75E-05 |
| Bra001614 | 1038 | Down | 9.74E-05 |
| Bra034904 | 717 | Down | 0 |
| Bra026973 | 2244 | Down | 7.04E-08 |
| Bra005918 | 618 | Down | 6.18E-13 |
| Bra009933 | 1209 | Down | 0.000325782 |
| Bra015780 | 1119 | Down | 1.91E-07 |
| Bra018029 | 918 | Down | 1.24E-08 |
| Bra014346 | 795 | Down | 1.84E-12 |
| Bra020361 | 1587 | Down | 2.31E-09 |
| Bra016720 | 1026 | Down | 4.14E-05 |
| Bra007142 | 756 | Down | 8.44E-08 |
| Bra012967 | 975 | Down | 4.14E-05 |
| Bra016148 | 885 | Down | 8.44E-08 |
| Bra010588 | 5820 | Down | 1.18E-11 |
| Bra024958 | 657 | Down | 1.40E-08 |
| Bra019285 | 807 | Down | 0 |
| Bra000018 | 948 | Down | 2.40E-07 |
| Bra010157 | 1245 | Down | 0.00013807 |
| Bra008101 | 567 | Down | 0 |
| Bra004284 | 987 | Down | 9.71E-13 |
| Bra024035 | 1770 | Down | 0 |
| Bra009861 | 2988 | Down | 1.14E-09 |
| Bra012655 | 642 | Down | 6.51E-13 |
| Bra024157 | 1050 | Down | 1.75E-05 |
| Bra005079 | 615 | Down | 1.05E-07 |
| Bra030355 | 627 | Down | 0 |
| Bra039681 | 735 | Down | 8.49E-13 |
| Bra033598 | 2559 | Down | 0.000464582 |
| Bra016740 | 1509 | Down | 4.73E-12 |
| Bra015867 | 909 | Down | 5.81E-05 |
| Bra012979 | 2208 | Down | 1.25E-09 |
| Bra018157 | 558 | Down | 0 |
| Bra022607 | 405 | Down | 2.36E-10 |
| Bra012166 | 1692 | Down | 1.35E-07 |
| Bra001975 | 1251 | Down | 0 |
| Bra030558 | 2211 | Down | 0 |
| Bra034183 | 2649 | Down | 0 |
| Bra001900 | 714 | Down | 2.26E-12 |
| Bra027069 | 1284 | Down | 0.000195969 |
| Bra020707 | 228 | Down | 0.000195944 |
| Bra016140 | 2442 | Down | 3.15E-06 |
| Bra001873 | 795 | Down | 3.15E-06 |
| Bra025935 | 3303 | Down | 0 |
| Bra003132 | 267 | Down | 1.04E-05 |
| Bra014604 | 1752 | Down | 8.26E-05 |
| Bra024144 | 369 | Down | 8.26E-05 |
| Bra022657 | 1902 | Down | 0.000661088 |
| Bra000783 | 870 | Down | 3.87E-12 |
| Bra022069 | 1878 | Down | 0 |
| Bra019088 | 165 | Down | 1.36E-11 |
| Bra030040 | 1173 | Down | 4.00E-13 |
| Bra007003 | 402 | Down | 4.37E-06 |
| Bra012779 | 2055 | Down | 5.19E-12 |
| Bra016416 | 5160 | Down | 0 |
| Bra004484 | 3855 | Down | 3.49E-13 |
| Bra001810 | 1629 | Down | 3.49E-13 |
| Bra001274 | 639 | Down | 3.32E-10 |
| Bra037033 | 981 | Down | 1.41E-08 |
| Bra010890 | 1059 | Down | 1.85E-06 |
| Bra025733 | 996 | Down | 2.39E-12 |
| Bra008346 | 951 | Down | 0.000277695 |
| Bra015283 | 2265 | Down | 0.000277661 |
| Bra001552 | 1191 | Down | 0.00027773 |
| Bra030931 | 729 | Down | 6.07E-09 |
| Bra020500 | 1101 | Down | 1.17E-12 |
| Bra013876 | 1644 | Down | 1.46E-05 |
| Bra020017 | 1485 | Down | 3.20E-11 |
| Bra022897 | 753 | Down | 1.41E-14 |
| Bra004946 | 2025 | Down | 0 |
| Bra018896 | 1086 | Down | 0 |
| Bra035147 | 1059 | Down | 0.000116513 |
| Bra006726 | 897 | Down | 9.10E-13 |
| Bra005601 | 840 | Down | 1.22E-12 |
| Bra009986 | 1245 | Down | 4.38E-12 |
| Bra030098 | 2997 | Down | 3.05E-12 |
| Bra035122 | 1176 | Down | 4.92E-05 |
| Bra008694 | 774 | Down | 4.92E-05 |
| Bra001855 | 1032 | Down | 5.94E-08 |
| Bra036690 | 1548 | Down | 5.94E-08 |
| Bra010642 | 861 | Down | 0 |
| Bra017717 | 837 | Down | 1.09E-06 |
| Bra006482 | 291 | Down | 0.000941158 |
| Bra021163 | 438 | Down | 0 |
| Bra029679 | 1449 | Down | 0 |
| Bra009308 | 1386 | Down | 2.06E-05 |
| Bra025137 | 1203 | Down | 6.24E-10 |
| Bra022144 | 534 | Down | 4.57E-07 |
| Bra016967 | 1557 | Down | 0.000393258 |
| Bra037553 | 1068 | Down | 8.68E-06 |
| Bra028846 | 801 | Down | 4.50E-09 |
| Bra032174 | 960 | Down | 2.20E-13 |
| Bra030143 | 735 | Down | 8.15E-08 |
| Bra001148 | 2472 | Down | 3.63E-06 |
| Bra028141 | 420 | Down | 3.63E-06 |
| Bra002961 | 1686 | Down | 0.000166186 |
| Bra012606 | 1362 | Down | 3.44E-08 |
| Bra039384 | 474 | Down | 5.97E-13 |
| Bra032747 | 2481 | Down | 1.28E-12 |
| Bra003596 | 1464 | Down | 0 |
| Bra030191 | 4563 | Down | 1.45E-08 |
| Bra001659 | 711 | Down | 6.95E-05 |
| Bra014972 | 990 | Down | 5.97E-13 |
| Bra018952 | 1821 | Down | 0 |
| Bra037776 | 1227 | Down | 2.69E-07 |
| Bra031006 | 2307 | Down | 2.69E-07 |
| Bra003938 | 1641 | Down | 6.42E-12 |
| Bra035807 | 1395 | Down | 2.91E-05 |
| Bra021096 | 651 | Down | 4.64E-10 |
| Bra027838 | 1023 | Down | 1.08E-12 |
| Bra024890 | 651 | Down | 4.76E-08 |
| Bra028640 | 1200 | Down | 5.12E-06 |
| Bra032614 | 1509 | Down | 5.12E-06 |
| Bra037365 | 1020 | Down | 3.86E-11 |
| Bra038844 | 2448 | Down | 0 |
| Bra012800 | 2367 | Down | 3.54E-09 |
| Bra018533 | 567 | Down | 2.15E-06 |
| Bra005811 | 1512 | Down | 8.98E-07 |
| Bra030028 | 2301 | Down | 8.98E-07 |
| Bra027411 | 1434 | Down | 8.99E-07 |
| Bra028453 | 1041 | Down | 0.000556219 |
| Bra010731 | 957 | Down | 0.000556283 |
| Bra024875 | 1722 | Down | 0 |
| Bra016236 | 1449 | Down | 6.20E-13 |
| Bra008495 | 909 | Down | 0 |
| Bra012567 | 897 | Down | 0.000235072 |
| Bra010542 | 561 | Down | 0.000235042 |
| Bra011982 | 348 | Down | 1.03E-11 |
| Bra023012 | 1212 | Down | 9.83E-05 |
| Bra034488 | 969 | Down | 9.83E-05 |
| Bra029717 | 2157 | Down | 8.66E-10 |
| Bra036499 | 798 | Down | 7.23E-06 |
| Bra024038 | 1086 | Down | 7.23E-06 |
| Bra024940 | 3975 | Down | 2.13E-12 |
| Bra037863 | 2121 | Down | 3.04E-06 |
| Bra010640 | 1119 | Down | 5.29E-07 |
| Bra007725 | 1149 | Down | 2.22E-07 |
| Bra036255 | 1590 | Down | 2.26E-12 |
| Bra031434 | 2421 | Down | 0 |
| Bra037411 | 861 | Down | 5.52E-13 |
| Bra037968 | 768 | Down | 3.94E-11 |
| Bra025286 | 1071 | Down | 2.50E-12 |
| Bra028024 | 978 | Down | 1.75E-12 |
| Bra025015 | 5103 | Down | 3.27E-13 |
| Bra038729 | 1833 | Down | 4.30E-12 |
| Bra035203 | 2658 | Down | 7.50E-07 |
| Bra005681 | 2082 | Down | 1.02E-05 |
| Bra011219 | 4257 | Down | 2.95E-10 |
| Bra003843 | 1149 | Down | 4.75E-13 |
| Bra031687 | 450 | Down | 0.000138871 |
| Bra020959 | 618 | Down | 0.000790218 |
| Bra029695 | 609 | Down | 0.000790129 |
| Bra029287 | 1038 | Down | 5.80E-05 |
| Bra025371 | 1356 | Down | 6.53E-13 |
| Bra005277 | 1029 | Down | 1.68E-09 |
| Bra005217 | 873 | Down | 0.000138889 |
| Bra006269 | 1890 | Down | 0.000138853 |
| Bra031385 | 873 | Down | 1.02E-05 |
| Bra031737 | 711 | Down | 1.79E-06 |
| Bra003171 | 612 | Down | 5.81E-05 |
| Bra025484 | 1788 | Down | 1.68E-09 |
| Bra004090 | 699 | Down | 1.79E-06 |
| Bra026990 | 1080 | Down | 1.68E-09 |
| Bra012838 | 2769 | Down | 4.01E-09 |
| Bra025007 | 825 | Down | 0 |
| Bra025140 | 1758 | Down | 0 |
| Bra003504 | 1062 | Down | 1.13E-12 |
| Bra034042 | 3075 | Down | 1.73E-10 |
| Bra000943 | 1803 | Down | 0 |
| Bra008013 | 1491 | Down | 0 |
| Bra015851 | 1197 | Down | 1.35E-08 |
| Bra013335 | 1428 | Down | 3.24E-08 |
| Bra006237 | 1041 | Down | 4.41E-07 |
| Bra016189 | 588 | Down | 0 |
| Bra007197 | 2238 | Down | 2.53E-06 |
| Bra016927 | 1287 | Down | 6.04E-06 |
| Bra020388 | 2646 | Down | 6.20E-13 |
| Bra041096 | 2016 | Down | 0 |
| Bra008221 | 768 | Down | 3.45E-05 |
| Bra001863 | 672 | Down | 3.45E-05 |
| Bra020471 | 870 | Down | 7.78E-13 |
| Bra033749 | 2568 | Down | 4.30E-13 |
| Bra005257 | 1380 | Down | 4.47E-14 |
| Bra041145 | 471 | Down | 2.38E-11 |
| Bra005530 | 2406 | Down | 8.22E-05 |
| Bra021668 | 522 | Down | 8.22E-05 |
| Bra023962 | 2148 | Down | 1.91E-08 |
| Bra037923 | 996 | Down | 1.91E-08 |
| Bra026105 | 3162 | Down | 1.91E-08 |
| Bra001291 | 456 | Down | 0 |
| Bra001288 | 1173 | Down | 0.000195633 |
| Bra015976 | 2889 | Down | 0 |
| Bra038032 | 1140 | Down | 1.09E-07 |
| Bra025380 | 363 | Down | 0.000464768 |
| Bra000893 | 1620 | Down | 2.60E-07 |
| Bra018599 | 2877 | Down | 2.60E-07 |
| Bra032901 | 489 | Down | 0.000464877 |
| Bra004530 | 1755 | Down | 0.000464822 |
| Bra000435 | 771 | Down | 6.22E-07 |
| Bra039578 | 798 | Down | 1.42E-12 |
| Bra004568 | 477 | Down | 2.34E-12 |
| Bra032730 | 1356 | Down | 3.83E-12 |
| Bra007713 | 243 | Down | 1.49E-06 |
| Bra029254 | 912 | Down | 0 |
| Bra019074 | 2478 | Down | 4.66E-09 |
| Bra019179 | 606 | Down | 2.23E-12 |
| Bra011830 | 1533 | Down | 1.89E-12 |
| Bra040236 | 624 | Down | 0 |
| Bra023188 | 843 | Down | 2.68E-08 |
| Bra040939 | 1410 | Down | 0 |
| Bra035698 | 690 | Down | 0 |
| Bra010309 | 846 | Down | 6.45E-08 |
| Bra035429 | 1785 | Down | 1.14E-09 |
| Bra019794 | 1278 | Down | 2.75E-09 |
| Bra017961 | 2166 | Down | 0 |
| Bra012887 | 471 | Down | 3.67E-07 |
| Bra014388 | 912 | Down | 3.67E-07 |
| Bra006364 | 504 | Down | 0 |
| Bra034717 | 528 | Down | 0 |
| Bra024789 | 1131 | Down | 5.05E-11 |
| Bra030620 | 1362 | Down | 8.77E-13 |
| Bra027730 | 1602 | Down | 0.00011507 |
| Bra012541 | 3681 | Down | 1.59E-08 |
| Bra001115 | 792 | Down | 0 |
| Bra003677 | 1011 | Down | 6.75E-10 |
| Bra033005 | 840 | Down | 0.000274374 |
| Bra030742 | 3459 | Down | 7.03E-11 |
| Bra004573 | 585 | Down | 1.20E-05 |
| Bra037429 | 1335 | Down | 1.66E-10 |
| Bra025289 | 1488 | Down | 1.20E-05 |
| Bra010638 | 411 | Down | 0.000653123 |
| Bra004318 | 915 | Down | 5.16E-07 |
| Bra034762 | 840 | Down | 0.000652973 |
| Bra034452 | 564 | Down | 0.000653198 |
| Bra023032 | 2412 | Down | 0.000652898 |
| Bra014285 | 402 | Down | 0.000653048 |
| Bra037874 | 1005 | Down | 1.41E-14 |
| Bra018342 | 1854 | Down | 2.85E-05 |
| Bra008773 | 2100 | Down | 0 |
| Bra036498 | 645 | Down | 8.90E-13 |
| Bra018448 | 2913 | Down | 4.21E-11 |
| Bra000169 | 2781 | Down | 0 |
| Bra025374 | 930 | Down | 1.24E-06 |
| Bra024382 | 882 | Down | 1.24E-06 |
| Bra004295 | 597 | Down | 4.46E-14 |
| Bra026569 | 708 | Down | 0 |
| Bra015647 | 1350 | Down | 6.81E-05 |
| Bra006039 | 417 | Down | 6.81E-05 |
| Bra035161 | 687 | Down | 1.27E-07 |
| Bra035104 | 1332 | Down | 1.27E-07 |
| Bra035056 | 1032 | Down | 2.52E-11 |
| Bra037761 | 507 | Down | 0 |
| Bra022804 | 828 | Down | 3.50E-12 |
| Bra009990 | 1383 | Down | 7.05E-06 |
| Bra017179 | 1326 | Down | 7.04E-06 |
| Bra001742 | 1068 | Down | 7.05E-06 |
| Bra006006 | 2940 | Down | 1.34E-09 |
| Bra009951 | 2097 | Down | 0 |
| Bra024915 | 1686 | Down | 3.21E-09 |
| Bra025418 | 519 | Down | 7.51E-08 |
| Bra024308 | 1797 | Down | 1.21E-12 |
| Bra014499 | 1020 | Down | 4.53E-12 |
| Bra040861 | 1419 | Down | 1.74E-06 |
| Bra006273 | 771 | Down | 2.05E-12 |
| Bra025731 | 732 | Down | 9.55E-12 |
| Bra020690 | 504 | Down | 1.85E-08 |
| Bra033720 | 1848 | Down | 4.02E-05 |
| Bra009832 | 1500 | Down | 4.02E-05 |
| Bra020013 | 3633 | Down | 1.66E-12 |
| Bra031686 | 1437 | Down | 2.12E-11 |
| Bra017810 | 702 | Down | 4.15E-06 |
| Bra006598 | 723 | Down | 0 |
| Bra039229 | 1290 | Down | 4.27E-07 |
| Bra022147 | 561 | Down | 4.85E-11 |
| Bra002470 | 1443 | Down | 9.58E-13 |
| Bra037226 | 996 | Down | 3.32E-12 |
| Bra024835 | 1203 | Down | 9.94E-06 |
| Bra000267 | 2019 | Down | 0 |
| Bra020236 | 2541 | Down | 0.000914086 |
| Bra019763 | 213 | Down | 9.56E-05 |
| Bra015742 | 741 | Down | 3.28E-13 |
| Bra036316 | 840 | Down | 0 |
| Bra018695 | 483 | Down | 3.28E-13 |
| Bra018178 | 1572 | Down | 2.44E-06 |
| Bra039022 | 1074 | Down | 2.44E-06 |
| Bra025170 | 957 | Down | 0 |
| Bra020464 | 447 | Down | 0.000227066 |
| Bra021594 | 642 | Down | 0.000227094 |
| Bra000419 | 222 | Down | 5.82E-06 |
| Bra005536 | 1569 | Down | 0 |
| Bra012540 | 3831 | Down | 0 |
| Bra020330 | 711 | Down | 3.52E-13 |
| Bra029869 | 597 | Down | 4.00E-13 |
| Bra017778 | 2688 | Down | 0 |
| Bra029743 | 1065 | Down | 0.000536736 |
| Bra031054 | 1779 | Down | 0.000536799 |
| Bra030778 | 2598 | Down | 8.96E-09 |
| Bra005176 | 792 | Down | 5.47E-14 |
| Bra007203 | 879 | Down | 4.35E-13 |
| Bra033886 | 729 | Down | 0 |
| Bra019255 | 1872 | Down | 4.08E-12 |
| Bra014137 | 1725 | Down | 0.000133005 |
| Bra030804 | 1998 | Down | 8.43E-07 |
| Bra000154 | 1968 | Down | 0 |
| Bra019092 | 2154 | Down | 2.07E-07 |
| Bra037316 | 753 | Down | 5.12E-08 |
| Bra010842 | 1569 | Down | 0 |
| Bra024670 | 9486 | Down | 8.15E-06 |
| Bra020607 | 1827 | Down | 2.01E-12 |
| Bra003423 | 723 | Down | 0 |
| Bra002137 | 1032 | Down | 4.94E-07 |
| Bra019202 | 210 | Down | 4.94E-07 |
| Bra034429 | 594 | Down | 1.22E-07 |
| Bra023567 | 312 | Down | 3.00E-08 |
| Bra035151 | 2262 | Down | 0 |
| Bra007446 | 771 | Down | 0 |
| Bra015996 | 2154 | Down | 1.94E-05 |
| Bra029762 | 1506 | Down | 1.18E-06 |
| Bra017640 | 1476 | Down | 1.18E-06 |
| Bra022864 | 1368 | Down | 1.31E-12 |
| Bra010806 | 708 | Down | 3.70E-13 |
| Bra016891 | 1092 | Down | 7.17E-08 |
| Bra020135 | 666 | Down | 1.05E-09 |
| Bra026610 | 2832 | Down | 4.58E-12 |
| Bra006104 | 2988 | Down | 0 |
| Bra033604 | 1572 | Down | 2.22E-12 |
| Bra028770 | 1161 | Down | 6.91E-07 |
| Bra021527 | 927 | Down | 1.52E-10 |
| Bra018107 | 765 | Down | 0.000749127 |
| Bra028456 | 1575 | Down | 0.000749212 |
| Bra012894 | 639 | Down | 4.60E-05 |
| Bra022903 | 1533 | Down | 2.52E-09 |
| Bra030739 | 4662 | Down | 0 |
| Bra004251 | 957 | Down | 4.96E-12 |
| Bra013706 | 1170 | Down | 2.45E-08 |
| Bra012599 | 330 | Down | 1.65E-06 |
| Bra029183 | 1590 | Down | 1.65E-06 |
| Bra037009 | 1008 | Down | 1.65E-06 |
| Bra025361 | 1002 | Down | 1.65E-06 |
| Bra035543 | 1047 | Down | 6.68E-06 |
| Bra008484 | 1908 | Down | 0 |
| Bra008705 | 966 | Down | 0 |
| Bra024150 | 786 | Down | 3.53E-09 |
| Bra012636 | 1191 | Down | 0 |
| Bra011665 | 4326 | Down | 0 |
| Bra023102 | 852 | Down | 1.25E-10 |
| Bra037512 | 3195 | Down | 0.00043711 |
| Bra038442 | 870 | Down | 0.000437058 |
| Bra016896 | 1560 | Down | 2.37E-07 |
| Bra003044 | 453 | Down | 0 |
| Bra024990 | 861 | Down | 9.61E-07 |
| Bra015819 | 819 | Down | 3.91E-06 |
| Bra006179 | 660 | Down | 0 |
| Bra017450 | 1461 | Down | 5.42E-13 |
| Bra009772 | 213 | Down | 1.58E-05 |
| Bra014670 | 987 | Down | 1.58E-05 |
| Bra014952 | 2535 | Down | 1.12E-11 |
| Bra005696 | 1071 | Down | 1.73E-10 |
| Bra027091 | 1080 | Down | 0.000256599 |
| Bra032137 | 912 | Down | 0 |
| Bra016276 | 1506 | Down | 0 |
| Bra034078 | 1563 | Down | 6.96E-13 |
| Bra017022 | 1080 | Down | 1.39E-12 |
| Bra024971 | 4128 | Down | 0 |
| Bra031920 | 1638 | Down | 0 |
| Bra020784 | 600 | Down | 0 |
| Bra025614 | 777 | Down | 3.12E-12 |
| Bra002103 | 1536 | Down | 2.31E-12 |
| Bra040220 | 1308 | Down | 9.85E-10 |
| Bra021371 | 579 | Down | 0.000151077 |
| Bra025223 | 1134 | Down | 0.000151097 |
| Bra007348 | 1488 | Down | 0.000151058 |
| Bra004477 | 789 | Down | 0 |
| Bra013816 | 2814 | Down | 7.85E-07 |
| Bra002479 | 2277 | Down | 0 |
| Bra013320 | 1194 | Down | 0 |
| Bra012512 | 738 | Down | 2.20E-05 |
| Bra027460 | 966 | Down | 2.20E-05 |
| Bra004380 | 1794 | Down | 0 |
| Bra039408 | 1017 | Down | 3.19E-06 |
| Bra025783 | 1578 | Down | 3.19E-06 |
| Bra011231 | 969 | Down | 4.59E-07 |
| Bra006620 | 1356 | Down | 0.000606166 |
| Bra015395 | 1281 | Down | 0 |
| Bra030422 | 744 | Down | 6.67E-08 |
| Bra009053 | 1071 | Down | 6.67E-08 |
| Bra001307 | 1245 | Down | 8.88E-05 |
| Bra008162 | 306 | Down | 0 |
| Bra001021 | 2142 | Down | 2.92E-11 |
| Bra004331 | 2601 | Down | 2.69E-07 |
| Bra025819 | 816 | Down | 0 |
| Bra015225 | 585 | Down | 0 |
| Bra013022 | 3684 | Down | 0 |
| Bra008052 | 876 | Down | 0 |
| Bra033664 | 273 | Down | 5.18E-05 |
| Bra004286 | 840 | Down | 5.18E-05 |
| Bra018602 | 627 | Down | 5.18E-05 |
| Bra019271 | 990 | Down | 4.68E-10 |
| Bra036877 | 1065 | Down | 6.79E-11 |
| Bra004261 | 810 | Down | 0 |
| Bra011547 | 648 | Down | 6.68E-12 |
| Bra021926 | 2532 | Down | 0 |
| Bra012713 | 1368 | Down | 9.21E-08 |
| Bra025405 | 348 | Down | 0 |
| Bra023828 | 1218 | Down | 3.04E-05 |
| Bra031668 | 1479 | Down | 5.41E-08 |
| Bra020982 | 1239 | Down | 0.00020877 |
| Bra020750 | 1317 | Down | 7.14E-14 |
| Bra032784 | 1791 | Down | 4.73E-12 |
| Bra012397 | 345 | Down | 8.92E-12 |
| Bra001117 | 960 | Down | 2.58E-06 |
| Bra024389 | 441 | Down | 1.78E-05 |
| Bra036125 | 1851 | Down | 0 |
| Bra018365 | 438 | Down | 0.000122014 |
| Bra024901 | 1554 | Down | 0.000121998 |
| Bra033423 | 384 | Down | 1.51E-06 |
| Bra017443 | 1644 | Down | 2.40E-12 |
| Bra013456 | 1782 | Down | 0 |
| Bra014582 | 3528 | Down | 1.53E-13 |
| Bra036764 | 954 | Down | 6.53E-13 |
| Bra011457 | 288 | Down | 0 |
| Bra039142 | 957 | Down | 0.000837881 |
| Bra032894 | 357 | Down | 5.74E-12 |
| Bra011638 | 8130 | Down | 7.49E-08 |
| Bra021145 | 2112 | Down | 0 |
| Bra024413 | 2994 | Down | 0 |
| Bra027243 | 762 | Down | 0 |
| Bra019653 | 1887 | Down | 1.41E-14 |
| Bra018589 | 663 | Down | 3.68E-09 |
| Bra015940 | 702 | Down | 3.68E-09 |
| Bra002096 | 1350 | Down | 9.09E-13 |
| Bra010441 | 1269 | Down | 3.17E-12 |
| Bra034073 | 2574 | Down | 0 |
| Bra012612 | 705 | Down | 3.57E-06 |
| Bra029416 | 945 | Down | 4.20E-05 |
| Bra028312 | 1365 | Down | 3.02E-07 |
| Bra025258 | 606 | Down | 4.47E-14 |
| Bra022547 | 2490 | Down | 1.02E-11 |
| Bra026348 | 753 | Down | 1.50E-08 |
| Bra023126 | 1587 | Down | 1.77E-07 |
| Bra037076 | 3036 | Down | 2.09E-06 |
| Bra014437 | 861 | Down | 6.21E-11 |
| Bra025458 | 1377 | Down | 2.46E-05 |
| Bra028912 | 1110 | Down | 0 |
| Bra015956 | 1368 | Down | 5.09E-09 |
| Bra025067 | 2076 | Down | 5.09E-09 |
| Bra003793 | 1017 | Down | 2.21E-11 |
| Bra023898 | 1269 | Down | 2.23E-12 |
| Bra040862 | 1191 | Down | 1.44E-05 |
| Bra035052 | 2571 | Down | 3.28E-13 |
| Bra011398 | 1464 | Down | 6.11E-12 |
| Bra013020 | 708 | Down | 8.53E-11 |
| Bra032775 | 417 | Down | 1.41E-14 |
| Bra006797 | 1332 | Down | 0 |
| Bra011827 | 963 | Down | 3.28E-13 |
| Bra026702 | 483 | Down | 1.21E-08 |
| Bra017511 | 909 | Down | 9.87E-05 |
| Bra020266 | 1488 | Down | 4.92E-06 |
| Bra022071 | 2319 | Down | 0 |
| Bra039387 | 1203 | Down | 6.96E-13 |
| Bra024840 | 3405 | Down | 1.43E-07 |
| Bra012230 | 663 | Down | 1.48E-11 |
| Bra007530 | 936 | Down | 2.88E-06 |
| Bra024761 | 2091 | Down | 1.09E-11 |
| Bra017920 | 378 | Down | 0 |
| Bra009805 | 1056 | Down | 0 |
| Bra011357 | 1035 | Down | 8.17E-10 |
| Bra033732 | 1575 | Down | 0.000672359 |
| Bra001598 | 585 | Down | 2.32E-13 |
| Bra029960 | 429 | Down | 0.000672435 |
| Bra021754 | 2865 | Down | 1.67E-08 |
| Bra015373 | 717 | Down | 0.000672512 |
| Bra026931 | 1401 | Down | 0.000672589 |
| Bra037460 | 1863 | Down | 1.98E-05 |
| Bra022944 | 1482 | Down | 0 |
| Bra008057 | 1146 | Down | 6.97E-13 |
| Bra023679 | 1434 | Down | 2.78E-10 |
| Bra013436 | 660 | Down | 3.36E-07 |
| Bra034184 | 1275 | Down | 0.000393594 |
| Bra017424 | 963 | Down | 0 |
| Bra001707 | 1500 | Down | 4.96E-12 |
| Bra007201 | 1641 | Down | 0 |
| Bra014036 | 2139 | Down | 1.15E-07 |
| Bra012042 | 1719 | Down | 2.32E-06 |
| Bra009485 | 1539 | Down | 7.03E-12 |
| Bra009994 | 1833 | Down | 0 |
| Bra037635 | 801 | Down | 1.35E-08 |
| Bra036418 | 714 | Down | 7.95E-05 |
| Bra004456 | 1098 | Down | 7.95E-05 |
| Bra024020 | 1230 | Down | 3.11E-12 |
| Bra014599 | 909 | Down | 7.95E-05 |
| Bra018436 | 534 | Down | 4.56E-09 |
| Bra024805 | 894 | Down | 4.63E-07 |
| Bra012350 | 750 | Down | 1.63E-11 |
| Bra024716 | 1218 | Down | 9.51E-12 |
| Bra025408 | 3165 | Down | 9.06E-10 |
| Bra028847 | 1044 | Down | 2.72E-05 |
| Bra009890 | 948 | Down | 0 |
| Bra009835 | 1869 | Down | 0 |
| Bra012531 | 1611 | Down | 1.59E-05 |
| Bra035099 | 708 | Down | 0 |
| Bra004452 | 921 | Down | 3.17E-08 |
| Bra016487 | 1296 | Down | 0 |
| Bra020536 | 645 | Down | 0 |
| Bra018964 | 855 | Down | 1.87E-06 |
| Bra029495 | 1950 | Down | 1.98E-12 |
| Bra040621 | 285 | Down | 0.000922462 |
| Bra031193 | 1239 | Down | 0 |
| Bra003765 | 951 | Down | 6.36E-07 |
| Bra010221 | 1797 | Down | 0.000539838 |
| Bra023574 | 1842 | Down | 8.53E-11 |
| Bra005543 | 1437 | Down | 4.82E-12 |
| Bra000930 | 1578 | Down | 0.000316503 |
| Bra004622 | 1611 | Down | 0.000316464 |
| Bra031640 | 2079 | Down | 5.02E-11 |
| Bra038781 | 243 | Down | 0.000316541 |
| Bra038439 | 279 | Down | 0.00031658 |
| Bra025232 | 4671 | Down | 1.31E-12 |
| Bra005207 | 4272 | Down | 3.29E-13 |
| Bra029343 | 1350 | Down | 0.00018562 |
| Bra032412 | 207 | Down | 0.000185644 |
| Bra032167 | 2046 | Down | 0.000185596 |
| Bra015020 | 2175 | Down | 9.68E-12 |
| Bra009947 | 249 | Down | 0 |
| Bra019910 | 915 | Down | 6.36E-05 |
| Bra039927 | 951 | Down | 3.73E-05 |
| Bra015737 | 1020 | Down | 3.73E-05 |
| Bra032962 | 1122 | Down | 3.73E-05 |
| Bra028906 | 867 | Down | 0 |
| Bra017928 | 1170 | Down | 2.18E-05 |
| Bra020154 | 840 | Down | 2.18E-05 |
| Bra025239 | 1725 | Down | 1.28E-05 |
| Bra011966 | 1584 | Down | 1.28E-05 |
| Bra002685 | 585 | Down | 7.47E-06 |
| Bra012661 | 645 | Down | 4.36E-06 |
| Bra019522 | 1605 | Down | 2.29E-13 |
| Bra022692 | 1344 | Down | 1.29E-12 |
| Bra029329 | 1893 | Down | 0 |
| Bra022708 | 3636 | Down | 0 |
| Bra031532 | 1089 | Down | 0 |
| Bra013945 | 1278 | Down | 2.32E-13 |
| Bra033713 | 813 | Down | 6.96E-13 |
| Bra038218 | 1941 | Down | 3.48E-08 |
| Bra031521 | 2034 | Down | 0 |
| Bra030129 | 1461 | Down | 4.04E-09 |
| Bra031260 | 1269 | Down | 0 |
| Bra025512 | 741 | Down | 2.02E-12 |
| Bra025138 | 744 | Down | 7.80E-14 |
| Bra003257 | 717 | Down | 7.80E-14 |
| Bra022255 | 1137 | Down | 0 |
| Bra011466 | 1290 | Down | 3.28E-13 |
| Bra003635 | 1320 | Down | 2.38E-07 |
| Bra024862 | 2181 | Down | 1.40E-07 |
| Bra024828 | 1353 | Down | 0 |
| Bra005106 | 1644 | Down | 0.000252347 |
| Bra016788 | 1218 | Down | 4.42E-11 |
| Bra031615 | 624 | Down | 0.000738381 |
| Bra001945 | 1104 | Down | 5.09E-05 |
| Bra012492 | 1404 | Down | 2.04E-06 |
| Bra000568 | 1788 | Down | 2.38E-07 |
| Bra030616 | 969 | Down | 2.98E-05 |
| Bra033989 | 1068 | Down | 0.000252378 |
| Bra016314 | 4173 | Down | 1.88E-09 |
| Bra002708 | 1740 | Down | 0.00043015 |
| Bra014361 | 420 | Down | 1.63E-08 |
| Bra013917 | 417 | Down | 0.000738297 |
| Bra026871 | 2475 | Down | 2.04E-06 |
| Bra012784 | 1473 | Down | 0 |
| Bra017113 | 354 | Down | 1.88E-11 |
| Bra025365 | 507 | Down | 0 |
| Bra017966 | 1089 | Down | 7.55E-12 |
| Bra001124 | 1758 | Down | 1.02E-10 |
| Bra029249 | 3522 | Down | 3.15E-12 |
| Bra014303 | 813 | Down | 3.82E-08 |
| Bra024177 | 1185 | Down | 0 |
| Bra017953 | 615 | Down | 1.11E-07 |
| Bra006523 | 372 | Down | 5.56E-07 |
| Bra031201 | 675 | Down | 0 |
| Bra028031 | 981 | Down | 9.52E-07 |
| Bra011216 | 993 | Down | 4.31E-13 |
| Bra025597 | 1086 | Down | 1.64E-06 |
| Bra024211 | 1464 | Down | 0 |
| Bra023538 | 1014 | Down | 0 |
| Bra005847 | 1998 | Down | 0 |
| Bra033022 | 777 | Down | 0 |
| Bra013651 | 2373 | Down | 7.01E-10 |
| Bra005693 | 1260 | Down | 0 |
| Bra018576 | 582 | Down | 0 |
| Bra017661 | 669 | Down | 1.78E-08 |
| Bra024750 | 1245 | Down | 0 |
| Bra004449 | 1836 | Down | 1.31E-12 |
| Bra010607 | 510 | Down | 0.000201866 |
| Bra012735 | 669 | Down | 0.00020184 |
| Bra023306 | 594 | Down | 8.88E-08 |
| Bra014828 | 1260 | Down | 0 |
| Bra025423 | 1488 | Down | 6.47E-11 |
| Bra031064 | 1725 | Down | 6.48E-11 |
| Bra020497 | 2469 | Down | 0.000343442 |
| Bra025409 | 1398 | Down | 2.37E-12 |
| Bra028883 | 1755 | Down | 4.44E-07 |
| Bra036266 | 1230 | Down | 5.59E-10 |
| Bra031857 | 1518 | Down | 4.48E-13 |
| Bra018563 | 1257 | Down | 6.50E-13 |
| Bra017739 | 1083 | Down | 3.07E-12 |
| Bra023585 | 762 | Down | 3.07E-12 |
| Bra026349 | 1167 | Down | 0 |
| Bra019121 | 1242 | Down | 0 |
| Bra039436 | 2460 | Down | 1.84E-11 |
| Bra017292 | 744 | Down | 2.42E-08 |
| Bra029693 | 1098 | Down | 0 |
| Bra008242 | 585 | Down | 0 |
| Bra032810 | 675 | Down | 1.90E-05 |
| Bra026786 | 1599 | Down | 1.90E-05 |
| Bra027937 | 588 | Down | 1.90E-05 |
| Bra013717 | 831 | Down | 0 |
| Bra029311 | 1119 | Down | 2.86E-12 |
| Bra017743 | 1956 | Down | 2.85E-12 |
| Bra032648 | 543 | Down | 0 |
| Bra023529 | 972 | Down | 2.48E-11 |
| Bra007060 | 1278 | Down | 5.50E-05 |
| Bra019912 | 1602 | Down | 2.21E-12 |
| Bra036903 | 1707 | Down | 6.59E-09 |
| Bra022254 | 1008 | Down | 7.70E-13 |
| Bra037639 | 285 | Down | 7.06E-11 |
| Bra005306 | 1344 | Down | 2.02E-12 |
| Bra009853 | 2208 | Down | 0 |
| Bra010553 | 1404 | Down | 2.86E-12 |
| Bra036979 | 1281 | Down | 1.15E-12 |
| Bra010346 | 456 | Down | 9.43E-05 |
| Bra016008 | 945 | Down | 1.93E-08 |
| Bra012619 | 627 | Down | 3.30E-08 |
| Bra024820 | 642 | Down | 0 |
| Bra001953 | 594 | Down | 4.32E-11 |
| Bra016822 | 861 | Down | 0.000273199 |
| Bra012615 | 939 | Down | 5.16E-06 |
| Bra005424 | 486 | Down | 0 |
| Bra000391 | 1539 | Down | 0 |
| Bra000161 | 654 | Down | 3.05E-09 |
| Bra027735 | 1863 | Down | 3.41E-12 |
| Bra026246 | 1212 | Down | 2.32E-13 |
| Bra016815 | 780 | Down | 0.000465392 |
| Bra025515 | 810 | Down | 0.000465337 |
| Bra008710 | 603 | Down | 0.000465282 |
| Bra026602 | 993 | Down | 1.51E-05 |
| Bra025598 | 810 | Down | 1.51E-05 |
| Bra013627 | 807 | Down | 1.51E-05 |
| Bra007669 | 1125 | Down | 3.58E-13 |
| Bra033208 | 987 | Down | 0 |
| Bra006167 | 1521 | Down | 2.57E-05 |
| Bra025072 | 2340 | Down | 2.58E-05 |
| Bra038714 | 1734 | Down | 0.000796867 |
| Bra028095 | 1179 | Down | 0 |
| Bra017426 | 414 | Down | 4.39E-05 |
| Bra019767 | 891 | Down | 4.39E-05 |
| Bra038157 | 501 | Down | 2.31E-12 |
| Bra020343 | 1539 | Down | 0 |
| Bra017220 | 900 | Down | 1.17E-11 |
| Bra013154 | 1596 | Down | 7.15E-15 |
| Bra011646 | 1593 | Down | 0 |
| Bra020391 | 750 | Down | 4.10E-06 |
| Bra002115 | 4152 | Down | 3.28E-13 |
| Bra005677 | 831 | Down | 9.84E-12 |
| Bra001537 | 3474 | Down | 5.92E-13 |
| Bra036060 | 3078 | Down | 2.21E-12 |
| Bra005273 | 2508 | Down | 3.81E-07 |
| Bra023979 | 1128 | Down | 7.00E-06 |
| Bra023683 | 1302 | Down | 0.000127349 |
| Bra012788 | 1470 | Down | 6.97E-12 |
| Bra010253 | 588 | Down | 0 |
| Bra012721 | 1410 | Down | 1.92E-09 |
| Bra007632 | 3168 | Down | 4.30E-12 |
| Bra015669 | 1374 | Down | 0 |
| Bra040930 | 840 | Down | 6.11E-08 |
| Bra023526 | 2556 | Down | 6.11E-08 |
| Bra009680 | 1764 | Down | 1.46E-12 |
| Bra037283 | 840 | Down | 5.16E-12 |
| Bra011773 | 1359 | Down | 0 |
| Bra016721 | 1956 | Down | 2.04E-05 |
| Bra033167 | 1776 | Down | 1.91E-06 |
| Bra029492 | 1524 | Down | 0.000369265 |
| Bra019448 | 3900 | Down | 0 |
| Bra040437 | 1200 | Down | 1.66E-08 |
| Bra020827 | 2046 | Down | 0 |
| Bra004375 | 2505 | Down | 1.32E-11 |
| Bra036467 | 1758 | Down | 2.82E-13 |
| Bra023993 | 1998 | Down | 1.45E-12 |
| Bra008668 | 1014 | Down | 2.21E-11 |
| Bra004313 | 498 | Down | 2.08E-12 |
| Bra015815 | 1344 | Down | 1.08E-12 |
| Bra037437 | 1143 | Down | 1.61E-12 |
| Bra004121 | 1605 | Down | 1.59E-13 |
| Bra003015 | 726 | Down | 4.11E-10 |
| Bra016775 | 2256 | Down | 4.46E-09 |
| Bra026607 | 2697 | Down | 5.17E-07 |
| Bra026219 | 933 | Down | 0.000629017 |
| Bra009707 | 618 | Down | 4.84E-08 |
| Bra023436 | 1455 | Down | 5.56E-06 |
| Bra026099 | 777 | Down | 9.53E-12 |
| Bra016646 | 3150 | Down | 1.14E-12 |
| Bra019171 | 1557 | Down | 9.52E-06 |
| Bra015032 | 888 | Down | 0.000100875 |
| Bra016172 | 1446 | Down | 0.000100862 |
| Bra039158 | 930 | Down | 1.41E-07 |
| Bra012519 | 1584 | Down | 6.53E-13 |
| Bra025381 | 1590 | Down | 1.13E-12 |
| Bra009846 | 1488 | Down | 1.62E-05 |
| Bra019768 | 2673 | Down | 2.40E-07 |
| Bra000691 | 1791 | Down | 2.40E-07 |
| Bra005098 | 405 | Down | 1.31E-12 |
| Bra023256 | 792 | Down | 0.000172745 |
| Bra011020 | 1857 | Down | 0 |
| Bra020372 | 1683 | Down | 4.09E-07 |
| Bra002834 | 2919 | Down | 4.09E-07 |
| Bra020260 | 1602 | Down | 1.38E-11 |
| Bra013019 | 1173 | Down | 0 |
| Bra020125 | 3303 | Down | 6.04E-12 |
| Bra015448 | 1362 | Down | 2.55E-12 |
| Bra005704 | 630 | Down | 3.28E-13 |
| Bra001946 | 1272 | Down | 1.04E-08 |
| Bra012910 | 882 | Down | 0 |
| Bra002455 | 1770 | Down | 0.000292598 |
| Bra006351 | 558 | Down | 0.000292562 |
| Bra036395 | 2454 | Down | 2.80E-09 |
| Bra034026 | 660 | Down | 2.80E-09 |
| Bra024514 | 1503 | Down | 2.80E-09 |
| Bra027702 | 1497 | Down | 4.70E-05 |
| Bra009918 | 1008 | Down | 2.80E-09 |
| Bra024646 | 1917 | Down | 7.15E-15 |
| Bra013748 | 1809 | Down | 7.57E-10 |
| Bra025080 | 948 | Down | 7.57E-10 |
| Bra011236 | 1326 | Down | 5.38E-12 |
| Bra040611 | 2265 | Down | 2.04E-10 |
| Bra022898 | 405 | Down | 0.000497265 |
| Bra029689 | 915 | Down | 8.23E-09 |
| Bra038681 | 453 | Down | 8.01E-05 |
| Bra034079 | 2973 | Down | 8.23E-09 |
| Bra000990 | 2544 | Down | 5.19E-08 |
| Bra002942 | 1824 | Down | 0 |
| Bra017473 | 555 | Down | 0 |
| Bra022809 | 738 | Down | 0 |
| Bra016457 | 1134 | Down | 0 |
| Bra004996 | 1440 | Down | 0 |
| Bra031266 | 1659 | Down | 8.81E-08 |
| Bra020393 | 564 | Down | 2.31E-12 |
| Bra026616 | 1536 | Down | 3.48E-06 |
| Bra009543 | 531 | Down | 1.65E-12 |
| Bra016195 | 579 | Down | 3.79E-09 |
| Bra028068 | 786 | Down | 2.42E-12 |
| Bra024199 | 966 | Down | 1.39E-12 |
| Bra019286 | 2643 | Down | 0 |
| Bra014603 | 1041 | Down | 0 |
| Bra025178 | 1503 | Down | 1.51E-07 |
| Bra002965 | 2262 | Down | 0 |
| Bra015942 | 1437 | Down | 0.00084769 |
| Bra032646 | 1620 | Down | 2.77E-10 |
| Bra037461 | 417 | Down | 0 |
| Bra015187 | 816 | Down | 9.62E-13 |
| Bra039691 | 1632 | Down | 2.57E-07 |
| Bra017950 | 324 | Down | 3.72E-05 |
| Bra016093 | 1356 | Down | 0 |
| Bra014515 | 1380 | Down | 1.62E-06 |
| Bra015653 | 1221 | Down | 4.71E-10 |
| Bra004165 | 462 | Down | 0 |
| Bra019338 | 2190 | Down | 7.01E-08 |
| Bra000353 | 1596 | Down | 1.01E-05 |
| Bra017837 | 1737 | Down | 5.42E-13 |
| Bra026547 | 1173 | Down | 4.38E-07 |
| Bra039627 | 2049 | Down | 0 |
| Bra040973 | 819 | Down | 8.10E-10 |
| Bra008924 | 651 | Down | 0 |
| Bra013211 | 456 | Down | 6.32E-05 |
| Bra008413 | 2811 | Down | 6.32E-05 |
| Bra004125 | 2343 | Down | 4.96E-13 |
| Bra029699 | 1455 | Down | 1.73E-05 |
| Bra009774 | 1368 | Down | 2.84E-12 |
| Bra025375 | 906 | Down | 5.93E-13 |
| Bra025297 | 801 | Down | 4.60E-11 |
| Bra003439 | 2358 | Down | 0 |
| Bra001348 | 1626 | Down | 5.15E-12 |
| Bra012627 | 1608 | Down | 7.82E-12 |
| Bra012833 | 1278 | Down | 0.000107349 |
| Bra001996 | 1911 | Down | 4.05E-09 |
| Bra016482 | 993 | Down | 2.93E-05 |
| Bra023515 | 933 | Down | 1.09E-09 |
| Bra036728 | 2448 | Down | 1.31E-12 |
| Bra017912 | 1419 | Down | 6.53E-13 |
| Bra039623 | 531 | Down | 6.93E-09 |
| Bra031902 | 237 | Down | 2.29E-13 |
| Bra000263 | 429 | Down | 4.42E-11 |
| Bra007662 | 1383 | Down | 5.00E-05 |
| Bra021187 | 507 | Down | 5.05E-12 |
| Bra030565 | 498 | Down | 1.36E-05 |
| Bra018714 | 1110 | Down | 0 |
| Bra029902 | 1371 | Down | 2.32E-13 |
| Bra020305 | 168 | Down | 7.44E-08 |
| Bra022337 | 1797 | Down | 5.45E-09 |
| Bra002080 | 471 | Down | 8.29E-12 |
| Bra030946 | 2013 | Down | 0 |
| Bra024913 | 1569 | Down | 0.000310641 |
| Bra009864 | 990 | Down | 2.32E-05 |
| Bra037966 | 2091 | Down | 9.08E-13 |
| Bra003879 | 1800 | Down | 0.000310679 |
| Bra037530 | 744 | Down | 2.52E-09 |
| Bra023137 | 1803 | Down | 0 |
| Bra001588 | 636 | Down | 2.01E-12 |
| Bra028459 | 8049 | Down | 5.83E-12 |
| Bra016749 | 1302 | Down | 0 |
| Bra002328 | 612 | Down | 3.73E-12 |
| Bra005408 | 948 | Down | 3.95E-05 |
| Bra006428 | 4140 | Down | 9.08E-13 |
| Bra009877 | 1050 | Down | 9.98E-08 |
| Bra019378 | 1095 | Down | 6.70E-11 |
| Bra022347 | 1275 | Down | 6.18E-13 |
| Bra005352 | 927 | Down | 3.67E-07 |
| Bra007170 | 783 | Down | 0.000526473 |
| Bra026257 | 864 | Down | 1.61E-12 |
| Bra010381 | 261 | Down | 4.63E-08 |
| Bra004831 | 1872 | Down | 1.83E-05 |
| Bra027715 | 1218 | Down | 1.83E-05 |
| Bra031967 | 552 | Down | 1.83E-05 |
| Bra011700 | 2079 | Down | 0 |
| Bra004506 | 1134 | Down | 6.25E-07 |
| Bra017667 | 1164 | Down | 1.94E-10 |
| Bra011058 | 2514 | Down | 1.61E-12 |
| Bra018529 | 981 | Down | 0 |
| Bra008661 | 198 | Down | 5.10E-11 |
| Bra023432 | 1185 | Down | 2.50E-11 |
| Bra030229 | 7560 | Down | 1.58E-12 |
| Bra016582 | 2031 | Down | 0.000245255 |
| Bra029550 | 846 | Down | 8.48E-06 |
| Bra017417 | 717 | Down | 2.89E-07 |
| Bra009745 | 4800 | Down | 1.06E-06 |
| Bra039274 | 1710 | Down | 0.000895544 |
| Bra036329 | 774 | Down | 0.000895444 |
| Bra024163 | 630 | Down | 3.10E-05 |
| Bra010665 | 537 | Down | 3.31E-12 |
| Bra006215 | 1467 | Down | 0 |
| Bra023087 | 4020 | Down | 1.43E-12 |
| Bra000553 | 1389 | Down | 0.00011327 |
| Bra030345 | 1230 | Down | 0.000113285 |
| Bra009868 | 1512 | Down | 1.44E-05 |
| Bra028650 | 1911 | Down | 6.23E-08 |
| Bra013833 | 1221 | Down | 3.34E-11 |
| Bra002963 | 1608 | Down | 5.26E-05 |
| Bra027324 | 1653 | Down | 5.26E-05 |
| Bra026659 | 849 | Down | 0 |
| Bra013372 | 3459 | Down | 6.18E-13 |
| Bra025073 | 2424 | Down | 2.45E-05 |
| Bra009667 | 753 | Down | 0.000192841 |
| Bra036821 | 1923 | Down | 0.000192866 |
| Bra018966 | 1194 | Down | 0 |
| Bra022246 | 1299 | Down | 0 |
| Bra001825 | 1569 | Down | 3.07E-13 |
| Bra037471 | 612 | Down | 7.62E-10 |
| Bra015994 | 1389 | Down | 0 |
| Bra013591 | 2121 | Down | 1.80E-07 |
| Bra012617 | 4293 | Down | 1.43E-06 |
| Bra022250 | 1896 | Down | 1.98E-12 |
| Bra027232 | 585 | Down | 1.43E-06 |
| Bra037453 | 3261 | Down | 1.62E-10 |
| Bra036324 | 2343 | Down | 1.14E-05 |
| Bra019673 | 1020 | Down | 8.97E-05 |
| Bra012868 | 3855 | Down | 0 |
| Bra025233 | 1905 | Down | 4.04E-12 |
| Bra029537 | 1515 | Down | 4.30E-13 |
| Bra011082 | 873 | Down | 3.05E-07 |
| Bra030027 | 2130 | Down | 4.15E-05 |
| Bra020774 | 1452 | Down | 4.76E-12 |
| Bra024808 | 459 | Down | 1.78E-08 |
| Bra035855 | 576 | Down | 0.000325763 |
| Bra035237 | 990 | Down | 0.000325802 |
| Bra030843 | 486 | Down | 3.03E-11 |
| Bra017720 | 798 | Down | 1.92E-05 |
| Bra013667 | 1686 | Down | 1.92E-05 |
| Bra023408 | 1869 | Down | 0 |
| Bra013406 | 498 | Down | 2.86E-12 |
| Bra023172 | 3114 | Down | 0 |
| Bra024806 | 1758 | Down | 4.68E-11 |
| Bra034925 | 702 | Down | 0 |
| Bra009909 | 1254 | Down | 1.40E-08 |
| Bra021275 | 1275 | Down | 0 |
| Bra034193 | 1035 | Down | 5.05E-12 |
| Bra023096 | 1587 | Down | 4.12E-06 |
| Bra004953 | 735 | Down | 7.04E-05 |
| Bra034394 | 624 | Down | 5.95E-11 |
| Bra034102 | 1710 | Down | 2.38E-08 |
| Bra025834 | 2454 | Down | 1.31E-12 |
| Bra004676 | 1836 | Down | 4.07E-07 |
| Bra007308 | 2058 | Down | 1.51E-05 |
| Bra026745 | 780 | Down | 1.67E-11 |
| Bra016095 | 1986 | Down | 1.89E-07 |
| Bra025078 | 900 | Down | 6.25E-11 |
| Bra003046 | 1638 | Down | 0 |
| Bra006908 | 1062 | Down | 5.74E-11 |
| Bra011204 | 624 | Down | 8.72E-08 |
| Bra007460 | 2205 | Down | 0.000256301 |
| Bra037348 | 258 | Down | 8.72E-08 |
| Bra000308 | 3075 | Down | 3.25E-06 |
| Bra035556 | 1413 | Down | 3.25E-06 |
| Bra004929 | 975 | Down | 0.000118866 |
| Bra004133 | 1125 | Down | 1.72E-13 |
| Bra023868 | 1737 | Down | 0 |
| Bra021219 | 645 | Down | 0.000118882 |
| Bra032365 | 957 | Down | 0 |
| Bra026845 | 975 | Down | 5.40E-13 |
| Bra016624 | 651 | Down | 2.35E-11 |
| Bra019908 | 948 | Down | 1.02E-11 |
| Bra022498 | 1371 | Down | 6.19E-13 |
| Bra035527 | 1203 | Down | 0 |
| Bra010013 | 1947 | Down | 0 |
| Bra022371 | 846 | Down | 1.18E-06 |
| Bra013744 | 1047 | Down | 0.000937678 |
| Bra020106 | 402 | Down | 6.63E-10 |
| Bra035633 | 906 | Down | 1.41E-10 |
| Bra014164 | 651 | Down | 0.00043329 |
| Bra020425 | 1029 | Down | 0 |
| Bra002835 | 831 | Down | 0.000201908 |
| Bra012243 | 1641 | Down | 0.000201934 |
| Bra030253 | 1533 | Down | 2.48E-12 |
| Bra002598 | 2565 | Down | 1.15E-08 |
| Bra002797 | 1122 | Down | 9.39E-05 |
| Bra001884 | 3672 | Down | 1.15E-08 |
| Bra000358 | 285 | Down | 9.39E-05 |
| Bra014818 | 612 | Down | 2.45E-09 |
| Bra011284 | 2916 | Down | 2.85E-12 |
| Bra025321 | 1128 | Down | 0 |
| Bra019803 | 1218 | Down | 2.78E-12 |
| Bra003143 | 2676 | Down | 2.00E-06 |
| Bra009777 | 1209 | Down | 9.23E-07 |
| Bra001134 | 408 | Down | 2.26E-13 |
| Bra019509 | 705 | Down | 0 |
| Bra024851 | 1710 | Down | 0 |
| Bra012827 | 1506 | Down | 5.93E-13 |
| Bra003999 | 2946 | Down | 0 |
| Bra012337 | 831 | Down | 0.000159 |
| Bra016160 | 999 | Down | 3.20E-10 |
| Bra013247 | 2022 | Down | 0.00015898 |
| Bra007019 | 609 | Down | 1.51E-09 |
| Bra033808 | 1374 | Down | 0.000737065 |
| Bra002610 | 876 | Down | 7.36E-05 |
| Bra019307 | 864 | Down | 1.57E-06 |
| Bra029084 | 789 | Down | 1.57E-06 |
| Bra029104 | 2472 | Down | 3.15E-12 |
| Bra028274 | 2328 | Down | 0 |
| Bra004824 | 609 | Down | 2.17E-12 |
| Bra004355 | 1029 | Down | 2.61E-08 |
| Bra031642 | 459 | Down | 3.77E-12 |
| Bra029258 | 1032 | Down | 0 |
| Bra010738 | 462 | Down | 4.11E-11 |
| Bra025022 | 1530 | Down | 4.11E-11 |
| Bra025523 | 1098 | Down | 1.96E-10 |
| Bra023806 | 5136 | Down | 6.42E-12 |
| Bra017818 | 1479 | Down | 2.68E-05 |
| Bra004387 | 471 | Down | 2.22E-12 |
| Bra008224 | 966 | Down | 0 |
| Bra004408 | 561 | Down | 3.34E-10 |
| Bra016820 | 696 | Down | 0.000575219 |
| Bra040414 | 2037 | Down | 0 |
| Bra011690 | 2148 | Down | 0 |
| Bra003482 | 861 | Down | 9.64E-07 |
| Bra021551 | 2172 | Down | 0 |
| Bra030603 | 2046 | Down | 4.50E-06 |
| Bra038299 | 2142 | Down | 0 |
| Bra002999 | 798 | Down | 9.26E-11 |
| Bra006319 | 1527 | Down | 2.62E-10 |
| Bra000354 | 1431 | Down | 7.51E-08 |
| Bra037017 | 915 | Down | 2.10E-05 |
| Bra025226 | 1494 | Down | 0 |
| Bra023664 | 675 | Down | 2.07E-11 |
| Bra029732 | 867 | Down | 1.42E-11 |
| Bra023239 | 2184 | Down | 6.69E-13 |
| Bra040700 | 2238 | Down | 1.14E-12 |
| Bra024920 | 1005 | Down | 1.13E-12 |
| Bra023966 | 711 | Down | 1.47E-12 |
| Bra041078 | 528 | Down | 1.64E-06 |
| Bra025076 | 2202 | Down | 2.92E-12 |
| Bra019533 | 1038 | Down | 2.72E-08 |
| Bra015814 | 978 | Down | 7.12E-12 |
| Bra035635 | 2844 | Down | 3.53E-06 |
| Bra008201 | 1173 | Down | 0 |
| Bra030291 | 1563 | Down | 0 |
| Bra015329 | 1617 | Down | 7.63E-06 |
| Bra004782 | 531 | Down | 0.000451414 |
| Bra017744 | 486 | Down | 1.65E-05 |
| Bra009203 | 2268 | Down | 5.31E-12 |
| Bra024909 | 864 | Down | 2.14E-08 |
| Bra030906 | 1515 | Down | 3.40E-12 |
| Bra008757 | 1170 | Down | 0.000975785 |
| Bra010546 | 603 | Down | 1.28E-06 |
| Bra014497 | 2289 | Down | 1.60E-13 |
| Bra016751 | 333 | Down | 0 |
| Bra040095 | 1461 | Down | 2.77E-06 |
| Bra015739 | 1731 | Down | 4.96E-12 |
| Bra010706 | 1350 | Down | 3.34E-12 |
| Bra037340 | 2406 | Down | 0 |
| Bra022459 | 1686 | Down | 6.72E-12 |
| Bra027200 | 1659 | Down | 1.29E-05 |
| Bra028863 | 1386 | Down | 0 |
| Bra027889 | 768 | Down | 0.000353581 |
| Bra031203 | 576 | Down | 4.24E-13 |
| Bra022556 | 1431 | Down | 2.17E-06 |
| Bra026408 | 3693 | Down | 4.68E-12 |
| Bra035214 | 1320 | Down | 0 |
| Bra038538 | 1533 | Down | 0 |
| Bra009708 | 468 | Down | 2.62E-11 |
| Bra000592 | 2481 | Down | 1.16E-11 |
| Bra011928 | 1020 | Down | 5.98E-05 |
| Bra024153 | 1914 | Down | 2.57E-12 |
| Bra025792 | 819 | Down | 4.66E-12 |
| Bra007206 | 1431 | Down | 0.000764779 |
| Bra013873 | 1077 | Down | 9.77E-13 |
| Bra005000 | 1812 | Down | 0 |
| Bra034845 | 1200 | Down | 6.05E-12 |
| Bra022166 | 702 | Down | 0.000128766 |
| Bra039620 | 1137 | Down | 2.85E-12 |
| Bra039488 | 543 | Down | 7.56E-12 |
| Bra005624 | 5868 | Down | 1.65E-11 |
| Bra012082 | 2010 | Down | 1.02E-10 |
| Bra010126 | 879 | Down | 0 |
| Bra004652 | 2673 | Down | 8.03E-09 |
| Bra034418 | 1518 | Down | 0 |
| Bra040090 | 828 | Down | 0.000277322 |
| Bra011328 | 2262 | Down | 5.15E-12 |
| Bra009679 | 486 | Down | 1.71E-05 |
| Bra037409 | 342 | Down | 0 |
| Bra016517 | 3033 | Down | 0.000595853 |
| Bra033613 | 1083 | Down | 3.08E-12 |
| Bra019229 | 1398 | Down | 0 |
| Bra005969 | 1662 | Down | 1.77E-09 |
| Bra015298 | 675 | Down | 3.53E-12 |
| Bra013303 | 1134 | Down | 0 |
| Bra004931 | 1626 | Down | 0.000217631 |
| Bra022895 | 642 | Down | 0.000217658 |
| Bra009916 | 1212 | Down | 7.15E-12 |
| Bra001140 | 1569 | Down | 0 |
| Bra030220 | 2568 | Down | 1.54E-12 |
| Bra036058 | 999 | Down | 0 |
| Bra031968 | 777 | Down | 3.76E-11 |
| Bra018568 | 555 | Down | 4.97E-11 |
| Bra030162 | 357 | Down | 6.38E-13 |
| Bra036450 | 969 | Down | 7.93E-05 |
| Bra015428 | 1752 | Down | 7.93E-05 |
| Bra025556 | 1746 | Down | 0 |
| Bra005136 | 1482 | Down | 2.21E-12 |
| Bra003133 | 870 | Down | 0.000466532 |
| Bra041010 | 1437 | Down | 9.77E-12 |
| Bra014559 | 429 | Down | 0 |
| Bra012670 | 1020 | Down | 0 |
| Bra007260 | 2247 | Down | 3.88E-08 |
| Bra019225 | 2040 | Down | 0.00017098 |
| Bra002632 | 1719 | Down | 0 |
| Bra018203 | 1332 | Down | 1.83E-09 |
| Bra014034 | 696 | Down | 0 |
| Bra008689 | 2691 | Down | 4.44E-12 |
| Bra035630 | 1986 | Down | 1.81E-07 |
| Bra027279 | 1953 | Down | 6.58E-08 |
| Bra036787 | 2082 | Down | 0 |
| Bra031037 | 2508 | Down | 1.13E-12 |
| Bra016053 | 2271 | Down | 3.47E-12 |
| Bra012660 | 705 | Down | 1.07E-06 |
| Bra011649 | 1260 | Down | 6.62E-11 |
| Bra007089 | 669 | Down | 4.86E-05 |
| Bra037501 | 1278 | Down | 4.86E-05 |
| Bra005002 | 756 | Down | 3.89E-07 |
| Bra016080 | 978 | Down | 1.76E-05 |
| Bra003375 | 780 | Down | 6.04E-12 |
| Bra040738 | 357 | Down | 0 |
| Bra029559 | 1188 | Down | 1.46E-10 |
| Bra019362 | 963 | Down | 6.40E-06 |
| Bra003541 | 837 | Down | 0 |
| Bra019150 | 2823 | Down | 6.72E-09 |
| Bra004960 | 1050 | Down | 2.42E-09 |
| Bra006873 | 2628 | Down | 0 |
| Bra000366 | 834 | Down | 0.000285943 |
| Bra038181 | 3009 | Down | 0.000285978 |
| Bra011178 | 786 | Down | 0.000286013 |
| Bra019589 | 1428 | Down | 0 |
| Bra039323 | 1596 | Down | 0 |
| Bra037992 | 2166 | Down | 0 |
| Bra007684 | 1671 | Down | 0.000104007 |
| Bra018998 | 1152 | Down | 0 |
| Bra009559 | 1056 | Down | 6.83E-10 |
| Bra005826 | 1071 | Down | 1.38E-05 |
| Bra035505 | 240 | Down | 0 |
| Bra036871 | 2580 | Down | 5.00E-06 |
| Bra014265 | 1296 | Down | 1.04E-12 |
| Bra039453 | 1197 | Down | 6.57E-07 |
| Bra015969 | 1299 | Down | 9.70E-12 |
| Bra027866 | 2790 | Down | 0 |
| Bra009326 | 1101 | Down | 6.29E-12 |
| Bra000648 | 792 | Down | 5.13E-07 |
| Bra021756 | 1662 | Down | 0.00022437 |
| Bra024014 | 531 | Down | 8.17E-05 |
| Bra030591 | 1218 | Down | 5.13E-07 |
| Bra016768 | 951 | Down | 2.75E-12 |
| Bra011276 | 1224 | Down | 0 |
| Bra010117 | 1668 | Down | 3.24E-10 |
| Bra012985 | 1701 | Down | 9.00E-10 |
| Bra023296 | 1176 | Down | 1.11E-06 |
| Bra031621 | 1014 | Down | 0 |
| Bra027406 | 546 | Down | 8.44E-06 |
| Bra023938 | 1101 | Down | 1.95E-09 |
| Bra018922 | 195 | Down | 6.37E-05 |
| Bra039434 | 291 | Down | 0.000175669 |
| Bra019205 | 2559 | Down | 0 |
| Bra018580 | 546 | Down | 1.14E-07 |
| Bra022882 | 495 | Down | 0.000479361 |
| Bra040968 | 1074 | Down | 0.000479418 |
| Bra001905 | 312 | Down | 3.14E-07 |
| Bra009794 | 3099 | Down | 0 |
| Bra015758 | 2391 | Down | 4.20E-09 |
| Bra007279 | 687 | Down | 0 |
| Bra011923 | 1374 | Down | 4.94E-12 |
| Bra036528 | 1038 | Down | 0 |
| Bra004270 | 2502 | Down | 5.97E-12 |
| Bra023324 | 1023 | Down | 6.46E-12 |
| Bra014044 | 1440 | Down | 0 |
| Bra028003 | 798 | Down | 0.000136941 |
| Bra035992 | 1581 | Down | 4.30E-12 |
| Bra024112 | 2451 | Down | 1.36E-11 |
| Bra031769 | 381 | Down | 5.14E-06 |
| Bra011060 | 1086 | Down | 0 |
| Bra033566 | 1194 | Down | 1.98E-12 |
| Bra012918 | 555 | Down | 0 |
| Bra038797 | 3948 | Down | 9.25E-12 |
| Bra031255 | 666 | Down | 1.46E-06 |
| Bra038316 | 1548 | Down | 7.79E-12 |
| Bra001653 | 480 | Down | 6.28E-11 |
| Bra030795 | 429 | Down | 4.02E-06 |
| Bra006745 | 744 | Down | 0.00010679 |
| Bra017384 | 1770 | Down | 1.54E-08 |
| Bra030135 | 2211 | Down | 0.000106775 |
| Bra016055 | 1539 | Down | 1.56E-11 |
| Bra033428 | 627 | Down | 0 |
| Bra006119 | 903 | Down | 0.000293391 |
| Bra012908 | 1680 | Down | 0.000293319 |
| Bra030041 | 1125 | Down | 0.000293355 |
| Bra034343 | 924 | Down | 0 |
| Bra010775 | 2172 | Down | 0 |
| Bra024816 | 3642 | Down | 0 |
| Bra009696 | 1794 | Down | 0 |
| Bra023077 | 885 | Down | 0.000806122 |
| Bra011423 | 1227 | Down | 0.000806032 |
| Bra026434 | 1488 | Down | 8.66E-06 |
| Bra016609 | 1641 | Down | 8.66E-06 |
| Bra028566 | 1515 | Down | 8.38E-05 |
| Bra004623 | 534 | Down | 8.67E-06 |
| Bra002583 | 828 | Down | 2.58E-08 |
| Bra039038 | 930 | Down | 3.15E-12 |
| Bra013141 | 1899 | Down | 7.16E-08 |
| Bra004620 | 642 | Down | 0 |
| Bra009930 | 3240 | Down | 1.21E-12 |
| Bra004045 | 2163 | Down | 5.93E-11 |
| Bra028941 | 2262 | Down | 0 |
| Bra022521 | 1608 | Down | 1.96E-07 |
| Bra029642 | 1389 | Down | 0 |
| Bra020894 | 828 | Down | 0 |
| Bra004944 | 2019 | Down | 1.92E-06 |
| Bra034363 | 342 | Down | 0 |
| Bra011293 | 405 | Down | 0.000179704 |
| Bra016250 | 705 | Down | 1.23E-08 |
| Bra033582 | 1341 | Down | 0 |
| Bra032413 | 519 | Down | 4.69E-13 |
| Bra003382 | 2658 | Down | 4.22E-07 |
| Bra003733 | 1323 | Down | 4.34E-12 |
| Bra025495 | 1566 | Down | 7.75E-11 |
| Bra027164 | 1719 | Down | 4.12E-06 |
| Bra012724 | 729 | Down | 0 |
| Bra019900 | 777 | Down | 0.000490551 |
| Bra025284 | 1170 | Down | 3.30E-07 |
| Bra033107 | 1335 | Down | 3.30E-07 |
| Bra002366 | 858 | Down | 2.65E-08 |
| Bra012950 | 930 | Down | 4.00E-05 |
| Bra021758 | 1908 | Down | 2.40E-12 |
| Bra025295 | 5535 | Down | 3.07E-13 |
| Bra023647 | 2397 | Down | 9.09E-07 |
| Bra001897 | 1002 | Down | 9.08E-07 |
| Bra006090 | 3120 | Down | 9.09E-07 |
| Bra008156 | 543 | Down | 0 |
| Bra020975 | 1791 | Down | 0 |
| Bra026106 | 3663 | Down | 4.61E-10 |
| Bra012153 | 1323 | Down | 5.42E-13 |
| Bra028013 | 891 | Down | 8.87E-06 |
| Bra005842 | 1212 | Down | 3.11E-05 |
| Bra010911 | 1566 | Down | 2.51E-06 |
| Bra025115 | 1272 | Down | 0.000383037 |
| Bra037970 | 1095 | Down | 0.000383083 |
| Bra023217 | 855 | Down | 7.11E-07 |
| Bra014751 | 504 | Down | 0 |
| Bra027864 | 531 | Down | 0.000382991 |
| Bra029923 | 930 | Down | 4.54E-09 |
| Bra026371 | 2319 | Down | 1.01E-10 |
| Bra014427 | 207 | Down | 2.02E-12 |
| Bra018587 | 2814 | Down | 2.26E-12 |
| Bra023083 | 900 | Down | 0 |
| Bra006180 | 1074 | Down | 1.96E-06 |
| Bra001060 | 1434 | Down | 1.96E-06 |
| Bra007069 | 936 | Down | 2.44E-05 |
| Bra015725 | 1044 | Down | 0 |
| Bra005191 | 549 | Down | 9.84E-09 |
| Bra035086 | 1644 | Down | 0.000299852 |
| Bra039659 | 1383 | Down | 6.07E-10 |
| Bra012946 | 1131 | Down | 6.07E-10 |
| Bra040407 | 1605 | Down | 1.53E-06 |
| Bra024295 | 1305 | Down | 7.67E-09 |
| Bra032393 | 1074 | Down | 5.39E-06 |
| Bra034125 | 1470 | Down | 1.68E-09 |
| Bra017136 | 1368 | Down | 2.99E-11 |
| Bra010880 | 519 | Down | 0 |
| Bra003544 | 1845 | Down | 1.48E-05 |
| Bra012110 | 2106 | Down | 1.66E-08 |
| Bra033156 | 855 | Down | 3.88E-12 |
| Bra014359 | 987 | Down | 0.000822622 |
| Bra008245 | 1737 | Down | 9.28E-07 |
| Bra015913 | 672 | Down | 5.20E-05 |
| Bra030872 | 1308 | Down | 3.29E-06 |
| Bra023859 | 795 | Down | 2.05E-07 |
| Bra023972 | 1557 | Down | 7.95E-10 |
| Bra040178 | 831 | Down | 0 |
| Bra011485 | 1320 | Down | 0 |
| Bra026341 | 987 | Down | 2.31E-12 |
| Bra013809 | 1254 | Down | 5.52E-13 |
| Bra030249 | 825 | Down | 4.55E-08 |
| Bra032909 | 2985 | Down | 1.66E-12 |
| Bra006747 | 1494 | Down | 1.00E-08 |
| Bra033719 | 801 | Down | 4.08E-05 |
| Bra015779 | 588 | Down | 4.71E-11 |
| Bra018627 | 2331 | Down | 0.000641366 |
| Bra011769 | 1287 | Down | 0.000641293 |
| Bra031998 | 3603 | Down | 0 |
| Bra008012 | 1689 | Down | 0 |
| Bra001634 | 819 | Down | 1.25E-07 |
| Bra016173 | 1899 | Down | 0 |
| Bra033662 | 2316 | Down | 4.12E-13 |
| Bra006355 | 501 | Down | 4.41E-07 |
| Bra026367 | 399 | Down | 9.75E-08 |
| Bra036479 | 624 | Down | 7.03E-06 |
| Bra023291 | 1005 | Down | 1.44E-13 |
| Bra000231 | 978 | Down | 0.000111117 |
| Bra001706 | 321 | Down | 0.000111102 |
| Bra035526 | 2091 | Down | 8.12E-10 |
| Bra010473 | 2808 | Down | 8.95E-13 |
| Bra012749 | 906 | Down | 1.31E-08 |
| Bra033324 | 990 | Down | 1.31E-08 |
| Bra006082 | 798 | Down | 1.90E-12 |
| Bra011248 | 588 | Down | 4.28E-06 |
| Bra014820 | 2145 | Down | 2.39E-11 |
| Bra006794 | 282 | Down | 0.000389563 |
| Bra028745 | 630 | Down | 0.000389516 |
| Bra026727 | 702 | Down | 0.000389656 |
| Bra041026 | 1071 | Down | 8.73E-05 |
| Bra006123 | 945 | Down | 0.00038961 |
| Bra027897 | 1503 | Down | 4.46E-11 |
| Bra012989 | 1353 | Down | 6.61E-11 |
| Bra038663 | 753 | Down | 2.99E-10 |
| Bra000142 | 498 | Down | 1.51E-05 |
| Bra019239 | 1047 | Down | 1.51E-05 |
| Bra027519 | 294 | Down | 0 |
| Bra019149 | 1323 | Down | 6.80E-05 |
| Bra023497 | 1299 | Down | 6.80E-05 |
| Bra020886 | 438 | Down | 0.000305083 |
| Bra035800 | 2820 | Down | 0 |
| Bra031914 | 1698 | Down | 1.18E-05 |
| Bra005894 | 1332 | Down | 1.82E-10 |
| Bra020635 | 2181 | Down | 9.93E-08 |
| Bra026843 | 876 | Down | 0 |
| Bra012725 | 858 | Down | 2.03E-06 |
| Bra023148 | 2118 | Down | 0.000238839 |
| Bra025272 | 846 | Down | 0 |
| Bra038233 | 1686 | Down | 0 |
| Bra003679 | 339 | Down | 9.21E-06 |
| Bra037780 | 1683 | Down | 9.20E-06 |
| Bra034298 | 1578 | Down | 1.20E-12 |
| Bra027918 | 702 | Down | 4.14E-05 |
| Bra004683 | 429 | Down | 4.07E-12 |
| Bra018050 | 1269 | Down | 2.28E-09 |
| Bra025772 | 2847 | Down | 7.68E-12 |
| Bra040150 | 765 | Down | 2.73E-07 |
| Bra038523 | 936 | Down | 0 |
| Bra020130 | 2460 | Down | 0 |
| Bra016013 | 2235 | Down | 5.21E-11 |
| Bra038111 | 1758 | Down | 0 |
| Bra029975 | 1332 | Down | 3.23E-05 |
| Bra007320 | 1089 | Down | 1.67E-07 |
| Bra038796 | 750 | Down | 0.000145192 |
| Bra038680 | 603 | Down | 0.000145173 |
| Bra025325 | 1092 | Down | 0 |
| Bra000220 | 4761 | Down | 1.44E-10 |
| Bra000279 | 2364 | Down | 4.35E-06 |
| Bra013003 | 885 | Down | 4.35E-06 |
| Bra015771 | 1746 | Down | 3.83E-09 |
| Bra016717 | 2760 | Down | 3.83E-09 |
| Bra004378 | 666 | Down | 5.04E-13 |
| Bra019349 | 963 | Down | 0 |
| Bra007164 | 1155 | Down | 1.31E-12 |
| Bra029826 | 990 | Down | 5.85E-07 |
| Bra027791 | 2712 | Down | 0.000112846 |
| Bra012820 | 2106 | Down | 0.000650682 |
| Bra018723 | 234 | Down | 3.40E-06 |
| Bra011128 | 1503 | Down | 1.65E-12 |
| Bra023975 | 2940 | Down | 0 |
| Bra015682 | 1488 | Down | 1.53E-05 |
| Bra022196 | 630 | Down | 3.56E-07 |
| Bra011221 | 1710 | Down | 6.66E-13 |
| Bra021099 | 1290 | Down | 1.10E-09 |
| Bra033169 | 1980 | Down | 1.40E-12 |
| Bra032869 | 435 | Down | 6.90E-05 |
| Bra011578 | 597 | Down | 3.74E-08 |
| Bra013957 | 1494 | Down | 4.98E-09 |
| Bra022702 | 2088 | Down | 1.13E-10 |
| Bra006088 | 654 | Down | 0.000395162 |
| Bra041037 | 2670 | Down | 6.76E-12 |
| Bra001089 | 2391 | Down | 1.98E-12 |
| Bra039277 | 198 | Down | 5.36E-05 |
| Bra024838 | 1758 | Down | 3.88E-09 |
| Bra018492 | 1569 | Down | 1.14E-12 |
| Bra037334 | 2481 | Down | 5.17E-10 |
| Bra019137 | 2439 | Down | 7.21E-13 |
| Bra018834 | 2556 | Down | 4.27E-11 |
| Bra035232 | 1497 | Down | 5.67E-06 |
| Bra005187 | 1326 | Down | 2.44E-10 |
| Bra016167 | 4626 | Down | 1.11E-09 |
| Bra024643 | 1164 | Down | 1.11E-09 |
| Bra039500 | 903 | Down | 8.37E-09 |
| Bra011617 | 1374 | Down | 3.44E-06 |
| Bra013672 | 1491 | Down | 4.86E-08 |
| Bra033759 | 1599 | Down | 0.000147008 |
| Bra007448 | 2709 | Down | 2.30E-08 |
| Bra040061 | 2067 | Down | 1.43E-13 |
| Bra001547 | 1776 | Down | 0 |
| Bra021856 | 771 | Down | 2.47E-10 |
| Bra018864 | 612 | Down | 1.71E-07 |
| Bra009109 | 219 | Down | 1.21E-05 |
| Bra034103 | 960 | Down | 0 |
| Bra020875 | 1056 | Down | 1.27E-06 |
| Bra029272 | 684 | Down | 1.33E-07 |
| Bra023673 | 1113 | Down | 0 |
| Bra036844 | 693 | Down | 1.40E-08 |
| Bra012069 | 2220 | Down | 2.21E-12 |
| Bra024044 | 1707 | Down | 1.04E-07 |
| Bra031268 | 648 | Down | 0.000843841 |
| Bra013536 | 630 | Down | 8.11E-08 |
| Bra017076 | 954 | Down | 4.44E-12 |
| Bra015547 | 1086 | Down | 0 |
| Bra037903 | 1371 | Down | 1.22E-12 |
| Bra020461 | 1185 | Down | 0 |
| Bra017722 | 2862 | Down | 6.97E-05 |
| Bra009758 | 768 | Down | 6.11E-13 |
| Bra025413 | 2043 | Down | 5.20E-12 |
| Bra027370 | 3006 | Down | 5.74E-11 |
| Bra001642 | 1356 | Down | 6.00E-07 |
| Bra004259 | 1950 | Down | 0.000657449 |
| Bra038078 | 582 | Down | 0.000657524 |
| Bra016782 | 2748 | Down | 0.000657374 |
| Bra006367 | 2352 | Down | 4.68E-12 |
| Bra040682 | 1242 | Down | 5.42E-05 |
| Bra023035 | 1290 | Down | 4.46E-06 |
| Bra023909 | 150 | Down | 2.23E-12 |
| Bra000318 | 1788 | Down | 4.24E-05 |
| Bra015635 | 1335 | Down | 3.48E-06 |
| Bra033427 | 2391 | Down | 3.48E-06 |
| Bra019666 | 1308 | Down | 3.31E-05 |
| Bra024136 | 192 | Down | 3.31E-05 |
| Bra017407 | 1320 | Down | 0.000399123 |
| Bra000023 | 2169 | Down | 1.14E-09 |
| Bra038305 | 1770 | Down | 4.90E-12 |
| Bra012815 | 5715 | Down | 3.16E-12 |
| Bra038070 | 2733 | Down | 1.35E-07 |
| Bra018309 | 1776 | Down | 1.61E-12 |
| Bra038022 | 10965 | Down | 0 |
| Bra022436 | 804 | Down | 6.04E-12 |
| Bra019410 | 3471 | Down | 1.29E-06 |
| Bra002604 | 1653 | Down | 2.37E-12 |
| Bra029347 | 723 | Down | 0.000244196 |
| Bra004589 | 735 | Down | 0.000244166 |
| Bra013379 | 1875 | Down | 0.000244227 |
| Bra019761 | 2274 | Down | 0.000244135 |
| Bra006762 | 1719 | Down | 8.19E-08 |
| Bra023546 | 1065 | Down | 9.98E-07 |
| Bra033436 | 1029 | Down | 0 |
| Bra023045 | 1962 | Down | 7.17E-12 |
| Bra036507 | 831 | Down | 0.000190193 |
| Bra002896 | 1257 | Down | 1.22E-05 |
| Bra023981 | 516 | Down | 0.000190169 |
| Bra002528 | 483 | Down | 0 |
| Bra036015 | 1113 | Down | 6.06E-07 |
| Bra007083 | 1155 | Down | 0 |
| Bra034710 | 1785 | Down | 0 |
| Bra024863 | 1602 | Down | 2.97E-12 |
| Bra018623 | 1056 | Down | 0 |
| Bra037229 | 660 | Down | 4.82E-12 |
| Bra023501 | 4011 | Down | 4.32E-13 |
| Bra016536 | 2022 | Down | 0 |
| Bra025114 | 1881 | Down | 4.50E-06 |
| Bra039184 | 918 | Down | 4.50E-06 |
| Bra011088 | 1923 | Down | 2.24E-07 |
| Bra011184 | 1476 | Down | 1.31E-12 |
| Bra020322 | 339 | Down | 7.04E-05 |
| Bra025265 | 774 | Down | 7.04E-05 |
| Bra033569 | 423 | Down | 1.36E-07 |
| Bra022243 | 390 | Down | 2.74E-06 |
| Bra002878 | 1281 | Down | 0 |
| Bra019250 | 255 | Down | 4.28E-05 |
| Bra032135 | 243 | Down | 4.28E-05 |
| Bra038743 | 708 | Down | 4.68E-12 |
| Bra012106 | 2055 | Down | 0 |
| Bra012642 | 2574 | Down | 5.44E-13 |
| Bra010084 | 906 | Down | 2.49E-12 |
| Bra018684 | 1419 | Down | 1.86E-12 |
| Bra014091 | 846 | Down | 4.38E-11 |
| Bra022698 | 1296 | Down | 0.000850516 |
| Bra010732 | 1068 | Down | 0.000850611 |
| Bra009287 | 2811 | Down | 3.57E-11 |
| Bra023967 | 546 | Down | 1.68E-11 |
| Bra039179 | 1065 | Down | 9.05E-10 |
| Bra020491 | 1182 | Down | 4.30E-13 |
| Bra027956 | 2523 | Down | 0 |
| Bra016298 | 681 | Down | 0.000662062 |
| Bra034704 | 579 | Down | 3.07E-11 |
| Bra004438 | 3027 | Down | 3.31E-10 |
| Bra025683 | 1143 | Down | 2.34E-12 |
| Bra008836 | 1713 | Down | 4.01E-12 |
| Bra018596 | 1131 | Down | 6.23E-12 |
| Bra019352 | 1245 | Down | 9.64E-06 |
| Bra023033 | 1275 | Down | 0 |
| Bra040498 | 2187 | Down | 1.37E-07 |
| Bra012439 | 669 | Down | 0.000314673 |
| Bra034196 | 1434 | Down | 0.000314711 |
| Bra002686 | 903 | Down | 4.53E-06 |
| Bra040575 | 663 | Down | 3.54E-06 |
| Bra004907 | 939 | Down | 0.000191473 |
| Bra019263 | 1845 | Down | 7.10E-10 |
| Bra031975 | 2340 | Down | 4.30E-13 |
| Bra035103 | 2454 | Down | 3.16E-12 |
| Bra024906 | 693 | Down | 0.000116081 |
| Bra039746 | 1239 | Down | 5.68E-12 |
| Bra022264 | 1032 | Down | 1.56E-12 |
| Bra039018 | 396 | Down | 8.80E-09 |
| Bra012148 | 2220 | Down | 7.36E-11 |
| Bra023362 | 2187 | Down | 7.08E-05 |
| Bra000393 | 642 | Down | 4.73E-12 |
| Bra004491 | 1563 | Down | 2.27E-07 |
| Bra028871 | 1305 | Down | 1.24E-05 |
| Bra022598 | 1332 | Down | 2.02E-12 |
| Bra037055 | 747 | Down | 1.46E-08 |
| Bra016870 | 942 | Down | 3.56E-06 |
| Bra035841 | 1635 | Down | 2.42E-12 |
| Bra032857 | 570 | Down | 0 |
| Bra018746 | 1104 | Down | 1.69E-06 |
| Bra039451 | 504 | Down | 1.02E-06 |
| Bra026239 | 1266 | Down | 0.000854147 |
| Bra028655 | 2073 | Down | 1.02E-06 |
| Bra006240 | 780 | Down | 7.96E-07 |
| Bra033597 | 3660 | Down | 4.91E-12 |
| Bra008557 | 1590 | Down | 2.26E-12 |
| Bra040529 | 846 | Down | 0.000518516 |
| Bra022632 | 2220 | Down | 1.85E-12 |
| Bra003194 | 942 | Down | 0 |
| Bra011863 | 1008 | Down | 0.000403171 |
| Bra023220 | 2511 | Down | 5.81E-11 |
| Bra014800 | 1578 | Down | 0.000316065 |
| Bra026891 | 930 | Down | 0.000316026 |
| Bra029658 | 780 | Down | 0.000246677 |
| Bra017290 | 774 | Down | 1.86E-12 |
| Bra021958 | 1713 | Down | 0.0001165 |
| Bra020340 | 1434 | Down | 1.14E-08 |
| Bra040360 | 729 | Down | 9.13E-05 |
| Bra005742 | 2148 | Down | 5.52E-05 |
| Bra012651 | 510 | Down | 5.52E-05 |
| Bra012146 | 1065 | Down | 3.37E-05 |
| Bra037362 | 1401 | Down | 0 |
| Bra005775 | 1308 | Down | 3.67E-11 |
| Bra026471 | 1674 | Down | 3.57E-06 |
| Bra028956 | 1935 | Down | 3.57E-06 |
| Bra014378 | 1881 | Down | 1.02E-11 |
| Bra027654 | 1995 | Down | 2.79E-06 |
| Bra014683 | 1389 | Down | 8.27E-12 |
| Bra011249 | 1452 | Down | 2.17E-06 |
| Bra012130 | 390 | Down | 1.32E-06 |
| Bra034675 | 846 | Down | 4.03E-12 |
| Bra014283 | 504 | Down | 2.42E-12 |
| Bra013048 | 720 | Down | 2.29E-07 |
| Bra002206 | 1698 | Down | 2.42E-08 |
| Bra023847 | 2022 | Down | 8.93E-09 |
| Bra000181 | 1755 | Down | 1.98E-12 |
| Bra040398 | 696 | Down | 0 |
| Bra014192 | 642 | Down | 4.33E-05 |
| Bra033796 | 1350 | Down | 1.25E-10 |
| Bra025784 | 936 | Down | 0.000150245 |
| Bra024117 | 3186 | Down | 0 |
| Bra016458 | 2376 | Down | 0.000150264 |
| Bra009385 | 957 | Down | 6.96E-09 |
| Bra020269 | 1719 | Down | 1.40E-07 |
| Bra016714 | 1497 | Down | 6.23E-07 |
| Bra029133 | 552 | Down | 0.000247045 |
| Bra016558 | 1179 | Down | 5.42E-13 |
| Bra011862 | 3585 | Down | 5.41E-13 |
| Bra012201 | 1002 | Down | 0.000519137 |
| Bra027863 | 1632 | Down | 3.58E-06 |
| Bra015735 | 942 | Down | 1.32E-06 |
| Bra032915 | 1194 | Down | 0.000316441 |
| Bra029631 | 1131 | Down | 0.000403631 |
| Bra014627 | 1353 | Down | 0.000116627 |
| Bra020176 | 1104 | Down | 0.000855052 |
| Bra033776 | 1578 | Down | 5.53E-05 |
| Bra019766 | 2604 | Down | 0 |
| Bra016477 | 591 | Down | 0.00040368 |
| Bra037474 | 1344 | Down | 0.000403583 |
| Bra037032 | 789 | Down | 3.58E-06 |
| Bra033284 | 1581 | Down | 0.000855148 |
| Bra026307 | 1095 | Down | 4.33E-05 |
| Bra037994 | 609 | Down | 0.000519198 |
| Bra001393 | 753 | Down | 2.42E-08 |
| Bra007335 | 882 | Down | 0.000519259 |
| Bra034101 | 978 | Down | 2.05E-05 |
| Bra028622 | 627 | Down | 2.64E-05 |
| Bra038261 | 675 | Down | 0.000403728 |
| Bra019133 | 1197 | Down | 5.72E-13 |
| Bra032316 | 2379 | Down | 0 |
| Bra024562 | 1893 | Down | 0 |
| Bra037525 | 1296 | Down | 4.44E-12 |
| Bra012850 | 2616 | Down | 5.47E-13 |
| Bra022718 | 6567 | Down | 0 |
| Bra024887 | 2925 | Down | 9.79E-11 |
| Bra025979 | 3039 | Down | 3.34E-12 |
| Bra033714 | 3159 | Down | 4.40E-10 |
| Bra008947 | 1173 | Down | 1.90E-08 |
| Bra027094 | 1278 | Down | 1.90E-08 |
| Bra000064 | 1422 | Down | 0 |
| Bra017951 | 981 | Down | 1.70E-06 |
| Bra014309 | 2565 | Down | 2.18E-06 |
| Bra024858 | 2850 | Down | 2.79E-06 |
| Bra017741 | 1314 | Down | 3.91E-13 |
| Bra025085 | 1467 | Down | 3.58E-06 |
| Bra016801 | 2553 | Down | 4.59E-06 |
| Bra019069 | 918 | Down | 5.91E-06 |
| Bra012785 | 1887 | Down | 9.76E-06 |
| Bra013870 | 1014 | Down | 3.44E-10 |
| Bra025518 | 2739 | Down | 0 |
| Bra024625 | 1500 | Down | 2.05E-05 |
| Bra004367 | 1425 | Down | 2.64E-05 |
| Bra030532 | 1188 | Down | 3.38E-05 |
| Bra040485 | 981 | Down | 3.38E-05 |
| Bra004280 | 1023 | Down | 3.38E-05 |
| Bra025315 | 1995 | Down | 4.20E-09 |
| Bra020569 | 1128 | Down | 1.75E-12 |
| Bra018907 | 309 | Down | 1.08E-12 |
| Bra023384 | 2592 | Down | 0 |
| Bra004391 | 885 | Down | 9.15E-05 |
| Bra012158 | 1806 | Down | 1.71E-12 |
| Bra024882 | 1695 | Down | 0.000150237 |
| Bra010171 | 474 | Down | 0.000150217 |
| Bra002336 | 522 | Down | 0.000150198 |
| Bra022585 | 1746 | Down | 1.51E-10 |
| Bra030202 | 1710 | Down | 4.66E-12 |
| Bra024796 | 1773 | Down | 6.64E-08 |
| Bra028781 | 1056 | Down | 8.47E-08 |
| Bra025273 | 1776 | Down | 0.000246872 |
| Bra007759 | 2964 | Down | 2.73E-12 |
| Bra012620 | 1056 | Down | 2.07E-10 |
| Bra024638 | 1155 | Down | 2.07E-10 |
| Bra007845 | 2871 | Down | 4.86E-07 |
| Bra008378 | 879 | Down | 0.000665024 |
| Bra023820 | 789 | Down | 9.36E-10 |
| Bra018938 | 762 | Down | 0.000854051 |
| Bra021386 | 2106 | Down | 1.70E-06 |
| Bra036248 | 3204 | Down | 6.25E-12 |
| Bra033615 | 3585 | Down | 3.15E-12 |
| Bra033644 | 537 | Down | 1.48E-08 |
| Bra025220 | 1341 | Down | 5.90E-06 |
| Bra024032 | 1314 | Down | 0 |
| Bra005527 | 2397 | Down | 3.13E-08 |
| Bra035943 | 672 | Down | 2.50E-12 |
| Bra019658 | 1317 | Down | 0 |
| Bra023618 | 1233 | Down | 1.25E-05 |
| Bra022485 | 804 | Down | 1.25E-05 |
| Bra016263 | 2247 | Down | 6.63E-08 |
| Bra019012 | 825 | Down | 7.28E-10 |
| Bra034960 | 1581 | Down | 2.63E-05 |
| Bra029710 | 774 | Down | 2.30E-07 |
| Bra012958 | 1356 | Down | 4.20E-09 |
| Bra039661 | 1731 | Down | 0 |
| Bra009609 | 399 | Down | 6.71E-13 |
| Bra040781 | 921 | Down | 7.11E-05 |
| Bra022567 | 969 | Down | 1.03E-06 |
| Bra004792 | 681 | Down | 1.61E-10 |
| Bra016806 | 657 | Down | 1.90E-08 |
| Bra000650 | 1704 | Down | 0.000116391 |
| Bra004767 | 2481 | Down | 0.000116406 |
| Bra036798 | 1245 | Down | 2.42E-08 |
| Bra006243 | 2157 | Down | 2.17E-06 |
| Bra038899 | 1116 | Down | 2.79E-06 |
| Bra013324 | 1821 | Down | 5.15E-08 |
| Bra034170 | 771 | Down | 0.000246144 |
| Bra014778 | 1149 | Down | 8.44E-08 |
| Bra034033 | 777 | Down | 8.44E-08 |
| Bra036176 | 1563 | Down | 0 |
| Bra008761 | 1113 | Down | 1.98E-09 |
| Bra027635 | 2853 | Down | 5.01E-13 |
| Bra032214 | 1275 | Down | 0.000402439 |
| Bra024537 | 582 | Down | 0.000402391 |
| Bra026515 | 3609 | Down | 6.19E-13 |
| Bra025672 | 1554 | Down | 2.04E-05 |
| Bra012963 | 849 | Down | 2.02E-11 |
| Bra034376 | 2013 | Down | 2.17E-11 |
| Bra011803 | 1242 | Down | 2.42E-08 |
| Bra036597 | 2949 | Down | 4.96E-12 |
| Bra019275 | 333 | Down | 3.11E-08 |
| Bra000739 | 804 | Down | 4.57E-11 |
| Bra009751 | 2223 | Down | 2.54E-09 |
| Bra000789 | 2307 | Down | 3.09E-12 |
| Bra014390 | 1314 | Down | 4.33E-13 |
| Bra007248 | 1464 | Down | 2.93E-07 |
| Bra009393 | 1425 | Down | 3.34E-12 |
| Bra033413 | 918 | Down | 2.41E-08 |
| Bra015951 | 4176 | Down | 4.32E-13 |
| Bra007343 | 2385 | Down | 0.000149156 |
| Bra012956 | 3162 | Down | 6.23E-12 |
| Bra028110 | 2535 | Down | 1.96E-09 |
| Bra018759 | 1974 | Down | 8.03E-12 |
| Bra006335 | 2040 | Down | 6.18E-07 |
| Bra019094 | 1392 | Down | 7.95E-07 |
| Bra002001 | 1563 | Down | 1.58E-05 |
| Bra018513 | 1335 | Down | 4.30E-12 |
| Bra026072 | 3348 | Down | 4.13E-12 |
| Bra027920 | 1572 | Down | 0 |
| Bra022183 | 2046 | Down | 2.15E-06 |
| Bra015783 | 2424 | Down | 1.31E-12 |
| Bra019897 | 1989 | Down | 0.00040033 |
| Bra032170 | 2133 | Down | 3.54E-06 |
| Bra019814 | 2460 | Down | 1.64E-11 |
| Bra026113 | 1737 | Down | 0 |
| Bra016920 | 1278 | Down | 4.29E-05 |
| Bra037380 | 489 | Down | 0 |
| Bra024961 | 1722 | Down | 0.000513783 |
| Bra000596 | 2328 | Down | 3.29E-12 |
| Bra011650 | 954 | Down | 2.27E-12 |
| Bra022645 | 447 | Down | 8.90E-13 |
| Bra034085 | 789 | Down | 5.48E-05 |
| Bra020796 | 1278 | Down | 5.83E-06 |
| Bra027532 | 1086 | Down | 5.83E-06 |
| Bra037800 | 909 | Down | 0.000658606 |
| Bra001244 | 1344 | Down | 6.54E-08 |
| Bra010459 | 1047 | Down | 7.75E-12 |
| Bra014433 | 771 | Down | 0 |
| Bra024918 | 1665 | Down | 1.01E-06 |
| Bra026579 | 1089 | Down | 9.62E-06 |
| Bra033382 | 1662 | Down | 6.52E-13 |
| Bra024095 | 2007 | Down | 3.12E-12 |
| Bra024902 | 456 | Down | 0.000115157 |
| Bra038045 | 408 | Down | 1.58E-05 |
| Bra005979 | 1440 | Down | 1.17E-12 |
| Bra030957 | 570 | Down | 0.00014817 |
| Bra027185 | 1782 | Down | 7.49E-12 |
| Bra027042 | 594 | Down | 0 |
| Bra007871 | 363 | Down | 6.52E-13 |
| Bra000973 | 2373 | Down | 2.24E-12 |
| Bra006603 | 1098 | Down | 0 |
| Bra005974 | 1158 | Down | 3.72E-07 |
| Bra000197 | 1029 | Down | 0.000243539 |
| Bra024445 | 327 | Down | 0.000243508 |
| Bra040791 | 1584 | Down | 0.000243569 |
| Bra017293 | 1344 | Down | 0 |
| Bra021077 | 1029 | Down | 4.68E-12 |
| Bra012760 | 366 | Down | 5.79E-06 |
| Bra017826 | 819 | Down | 5.79E-06 |
| Bra018624 | 513 | Down | 1.47E-12 |
| Bra020876 | 3102 | Down | 6.69E-13 |
| Bra009944 | 291 | Down | 0.00039737 |
| Bra017769 | 1509 | Down | 9.55E-06 |
| Bra018955 | 438 | Down | 4.71E-11 |
| Bra013739 | 525 | Down | 6.78E-09 |
| Bra038825 | 1302 | Down | 1.60E-13 |
| Bra014468 | 672 | Down | 2.01E-10 |
| Bra024725 | 2460 | Down | 3.46E-11 |
| Bra036518 | 912 | Down | 3.64E-11 |
| Bra033443 | 1338 | Down | 0.000509732 |
| Bra036497 | 735 | Down | 2.15E-12 |
| Bra016041 | 1065 | Down | 6.45E-08 |
| Bra018688 | 1071 | Down | 8.97E-05 |
| Bra001292 | 543 | Down | 1.57E-05 |
| Bra038031 | 891 | Down | 1.57E-05 |
| Bra015661 | 3090 | Down | 4.73E-07 |
| Bra021002 | 1887 | Down | 1.06E-07 |
| Bra033725 | 4923 | Down | 1.29E-12 |
| Bra021665 | 3918 | Down | 0 |
| Bra009306 | 3714 | Down | 1.06E-07 |
| Bra034851 | 936 | Down | 1.21E-10 |
| Bra038163 | 1344 | Down | 2.35E-08 |
| Bra011654 | 903 | Down | 1.36E-07 |
| Bra017428 | 1737 | Down | 2.57E-05 |
| Bra029702 | 198 | Down | 0.000146996 |
| Bra039076 | 1200 | Down | 3.02E-08 |
| Bra027498 | 474 | Down | 0 |
| Bra028519 | 465 | Down | 0.000838011 |
| Bra015155 | 1467 | Down | 0.000837917 |
| Bra007547 | 717 | Down | 5.75E-06 |
| Bra013624 | 2184 | Down | 0 |
| Bra033082 | 1395 | Down | 4.98E-08 |
| Bra023560 | 873 | Down | 2.86E-07 |
| Bra004898 | 2211 | Down | 3.66E-07 |
| Bra022787 | 564 | Down | 0.00024147 |
| Bra006870 | 645 | Down | 1.83E-08 |
| Bra019854 | 2340 | Down | 1.79E-12 |
| Bra025203 | 1083 | Down | 1.21E-05 |
| Bra025323 | 2028 | Down | 9.37E-12 |
| Bra020823 | 1665 | Down | 0 |
| Bra014963 | 3924 | Down | 7.75E-07 |
| Bra030155 | 1095 | Down | 4.57E-11 |
| Bra012978 | 918 | Down | 3.91E-13 |
| Bra032963 | 1341 | Down | 3.11E-09 |
| Bra021766 | 1734 | Down | 2.55E-05 |
| Bra021478 | 432 | Down | 8.86E-10 |
| Bra019839 | 1467 | Down | 2.48E-12 |
| Bra013931 | 972 | Down | 0 |
| Bra014655 | 1146 | Down | 1.81E-08 |
| Bra038816 | 1449 | Down | 8.12E-08 |
| Bra035239 | 1068 | Down | 0 |
| Bra007293 | 1110 | Down | 0.000504612 |
| Bra019026 | 1176 | Down | 4.59E-12 |
| Bra010684 | 3525 | Down | 3.48E-12 |
| Bra024819 | 960 | Down | 2.32E-08 |
| Bra026490 | 1791 | Down | 2.78E-12 |
| Bra037378 | 3039 | Down | 3.23E-10 |
| Bra033737 | 1338 | Down | 1.37E-13 |
| Bra026923 | 2559 | Down | 0.000145541 |
| Bra021638 | 1557 | Down | 1.43E-13 |
| Bra011404 | 636 | Down | 0.000647348 |
| Bra006212 | 1158 | Down | 0.000647199 |
| Bra005763 | 1098 | Down | 0.000647274 |
| Bra017730 | 2520 | Down | 3.34E-12 |
| Bra025074 | 3249 | Down | 2.19E-07 |
| Bra017183 | 570 | Down | 3.43E-06 |
| Bra018446 | 2244 | Down | 9.79E-07 |
| Bra015290 | 2337 | Down | 0.000829396 |
| Bra031931 | 1110 | Down | 0 |
| Bra024928 | 1263 | Down | 0.000239079 |
| Bra036473 | 3618 | Down | 4.39E-06 |
| Bra036276 | 951 | Down | 4.67E-12 |
| Bra015845 | 1560 | Down | 3.59E-12 |
| Bra007110 | 525 | Down | 0.000305083 |
| Bra002823 | 1059 | Down | 0.000305121 |
| Bra025370 | 903 | Down | 0.000305046 |
| Bra021282 | 2454 | Down | 3.78E-08 |
| Bra023502 | 1299 | Down | 7.05E-11 |
| Bra023706 | 342 | Down | 1.69E-07 |
| Bra006035 | 1020 | Down | 4.60E-13 |
| Bra007358 | 1077 | Down | 2.16E-07 |
| Bra025257 | 564 | Down | 0.00038944 |
| Bra016674 | 1710 | Down | 7.59E-07 |
| Bra037586 | 2454 | Down | 0 |
| Bra018212 | 1956 | Down | 0 |
| Bra035418 | 3324 | Down | 1.02E-11 |
| Bra037482 | 1953 | Down | 2.27E-08 |
| Bra010551 | 5961 | Down | 2.27E-08 |
| Bra036491 | 1671 | Down | 2.78E-07 |
| Bra013015 | 2409 | Down | 3.40E-06 |
| Bra006782 | 2709 | Down | 3.77E-12 |
| Bra000139 | 1011 | Down | 0.000498938 |
| Bra008606 | 489 | Down | 2.68E-12 |
| Bra024205 | 864 | Down | 2.42E-12 |
| Bra014850 | 906 | Down | 1.52E-05 |
| Bra040637 | 1737 | Down | 1.52E-05 |
| Bra012508 | 1242 | Down | 1.52E-05 |
| Bra035000 | 2736 | Down | 3.42E-11 |
| Bra025858 | 1860 | Down | 0 |
| Bra022853 | 510 | Down | 1.82E-09 |
| Bra022758 | 1242 | Down | 6.40E-09 |
| Bra019939 | 921 | Down | 7.13E-06 |
| Bra012180 | 3600 | Down | 2.25E-08 |
| Bra010715 | 1122 | Down | 0.000236342 |
| Bra014370 | 1008 | Down | 0.000236312 |
| Bra000085 | 936 | Down | 2.62E-06 |
| Bra013607 | 1332 | Down | 0.000819782 |
| Bra034856 | 1680 | Down | 8.70E-05 |
| Bra019199 | 2091 | Down | 0.00081969 |
| Bra012279 | 1440 | Down | 4.67E-12 |
| Bra006391 | 1245 | Down | 3.81E-12 |
| Bra001935 | 2088 | Down | 4.50E-07 |
| Bra004260 | 1326 | Down | 4.50E-07 |
| Bra013258 | 1299 | Down | 0.000110902 |
| Bra005286 | 1092 | Down | 1.66E-07 |
| Bra037043 | 1950 | Down | 3.39E-12 |
| Bra025018 | 1359 | Down | 0.000142105 |
| Bra004003 | 657 | Down | 7.41E-07 |
| Bra037297 | 756 | Down | 5.21E-05 |
| Bra003105 | 852 | Down | 1.92E-05 |
| Bra033590 | 354 | Down | 3.16E-11 |
| Bra016488 | 498 | Down | 0.000182079 |
| Bra038679 | 2091 | Down | 0.000182103 |
| Bra023254 | 1101 | Down | 4.44E-07 |
| Bra007048 | 1896 | Down | 1.64E-07 |
| Bra023060 | 996 | Down | 2.19E-08 |
| Bra030841 | 1800 | Down | 5.37E-11 |
| Bra011766 | 1587 | Down | 1.50E-11 |
| Bra030702 | 1290 | Down | 4.19E-12 |
| Bra026582 | 459 | Down | 1.16E-05 |
| Bra003419 | 738 | Down | 1.29E-12 |
| Bra007007 | 1221 | Down | 3.15E-05 |
| Bra024871 | 1716 | Down | 1.36E-09 |
| Bra027444 | 687 | Down | 0.000233149 |
| Bra013814 | 3063 | Down | 3.60E-08 |
| Bra025675 | 1344 | Down | 2.94E-12 |
| Bra029274 | 369 | Down | 1.99E-06 |
| Bra035459 | 1860 | Down | 2.44E-12 |
| Bra006337 | 1131 | Down | 5.38E-12 |
| Bra037151 | 1479 | Down | 4.04E-05 |
| Bra027831 | 2241 | Down | 0.000297163 |
| Bra039061 | 2031 | Down | 1.59E-13 |
| Bra010932 | 525 | Down | 0.000808047 |
| Bra004689 | 921 | Down | 2.85E-12 |
| Bra023912 | 951 | Down | 1.90E-05 |
| Bra007694 | 1221 | Down | 1.61E-07 |
| Bra014891 | 1803 | Down | 1.20E-06 |
| Bra032494 | 1296 | Down | 0.000140053 |
| Bra037715 | 846 | Down | 2.77E-08 |
| Bra014523 | 1335 | Down | 1.13E-11 |
| Bra016627 | 858 | Down | 0 |
| Bra007423 | 1290 | Down | 2.64E-07 |
| Bra013286 | 3345 | Down | 5.68E-12 |
| Bra000245 | 747 | Down | 0.000179555 |
| Bra017245 | 2850 | Down | 1.14E-05 |
| Bra021406 | 3609 | Down | 3.41E-12 |
| Bra018518 | 1323 | Down | 8.45E-05 |
| Bra021649 | 402 | Down | 3.93E-11 |
| Bra019044 | 2142 | Down | 2.19E-11 |
| Bra015683 | 1635 | Down | 3.15E-12 |
| Bra011136 | 3558 | Down | 4.81E-11 |
| Bra014848 | 909 | Down | 5.60E-13 |
| Bra004059 | 2601 | Down | 3.73E-10 |
| Bra017767 | 2982 | Down | 1.02E-09 |
| Bra000670 | 381 | Down | 6.74E-12 |
| Bra030504 | 900 | Down | 4.73E-12 |
| Bra002527 | 651 | Down | 4.90E-12 |
| Bra018873 | 372 | Down | 5.52E-07 |
| Bra010297 | 1923 | Down | 8.25E-11 |
| Bra011729 | 1635 | Down | 7.69E-09 |
| Bra031541 | 975 | Down | 0.000292603 |
| Bra031195 | 1035 | Down | 5.54E-12 |
| Bra025069 | 786 | Down | 3.33E-07 |
| Bra030962 | 1980 | Down | 4.59E-09 |
| Bra019618 | 1254 | Down | 5.02E-13 |
| Bra027115 | 3480 | Down | 2.89E-11 |
| Bra038716 | 402 | Down | 5.27E-06 |
| Bra020754 | 1035 | Down | 5.27E-06 |
| Bra015802 | 1089 | Down | 1.77E-11 |
| Bra016084 | 1677 | Down | 7.15E-12 |
| Bra005177 | 1086 | Down | 0 |
| Bra036184 | 1737 | Down | 4.49E-12 |
| Bra001822 | 618 | Down | 0.000177004 |
| Bra003460 | 954 | Down | 0.000177027 |
| Bra029989 | 1890 | Down | 5.44E-07 |
| Bra036462 | 1326 | Down | 6.77E-06 |
| Bra001848 | 849 | Down | 3.92E-05 |
| Bra036267 | 939 | Down | 5.67E-08 |
| Bra039262 | 1314 | Down | 3.92E-05 |
| Bra025346 | 1470 | Down | 0.000477496 |
| Bra021752 | 1956 | Down | 2.18E-10 |
| Bra037846 | 1581 | Down | 8.66E-06 |
| Bra007782 | 915 | Down | 1.52E-12 |
| Bra019386 | 783 | Down | 0.000105969 |
| Bra033283 | 1590 | Down | 3.50E-12 |
| Bra036542 | 1851 | Down | 4.19E-07 |
| Bra006556 | 3708 | Down | 1.98E-12 |
| Bra020793 | 1890 | Down | 5.61E-13 |
| Bra006522 | 615 | Down | 0 |
| Bra022839 | 978 | Down | 5.36E-07 |
| Bra037430 | 582 | Down | 0.000287777 |
| Bra011283 | 3726 | Down | 0 |
| Bra035049 | 2853 | Down | 4.48E-12 |
| Bra022926 | 669 | Down | 1.41E-05 |
| Bra014602 | 483 | Down | 1.41E-05 |
| Bra014598 | 405 | Down | 9.90E-12 |
| Bra012975 | 981 | Down | 3.70E-13 |
| Bra019302 | 2643 | Down | 0 |
| Bra016689 | 1770 | Down | 9.78E-14 |
| Bra011774 | 1365 | Down | 0 |
| Bra031700 | 984 | Down | 0.00078387 |
| Bra028291 | 714 | Down | 2.01E-08 |
| Bra028308 | 1224 | Down | 2.01E-08 |
| Bra012551 | 1404 | Down | 9.77E-11 |
| Bra024097 | 1920 | Down | 4.48E-10 |
| Bra032536 | 1368 | Down | 0.000783959 |
| Bra035461 | 873 | Down | 0.000367517 |
| Bra003225 | 1437 | Down | 0.000784048 |
| Bra015846 | 1488 | Down | 8.79E-07 |
| Bra034519 | 414 | Up | 1.60E-207 |
| Bra012057 | 528 | Up | 2.98E-187 |
| Bra008531 | 453 | Up | 1.90E-75 |
| Bra014162 | 1746 | Up | 2.38E-271 |
| Bra019091 | 360 | Up | 1.22E-40 |
| Bra016075 | 627 | Up | 7.96E-70 |
| Bra008238 | 405 | Up | 1.54E-36 |
| Bra025766 | 1035 | Up | 3.44E-88 |
| Bra010320 | 1350 | Up | 1.93E-105 |
| Bra008316 | 1128 | Up | 8.06E-82 |
| Bra003118 | 540 | Up | 6.51E-37 |
| Bra019543 | 705 | Up | 5.42E-47 |
| Bra005479 | 600 | Up | 6.76E-40 |
| Bra005114 | 705 | Up | 1.24E-43 |
| Bra039971 | 789 | Up | 1.73E-48 |
| Bra030336 | 288 | Up | 7.95E-17 |
| Bra039120 | 1923 | Up | 1.92E-111 |
| Bra008532 | 780 | Up | 5.27E-44 |
| Bra023455 | 303 | Up | 1.85E-16 |
| Bra015518 | 357 | Up | 2.01E-19 |
| Bra003214 | 606 | Up | 8.18E-33 |
| Bra012379 | 522 | Up | 5.66E-28 |
| Bra005262 | 1149 | Up | 4.36E-60 |
| Bra021614 | 2274 | Up | 8.09E-115 |
| Bra028932 | 1032 | Up | 9.85E-51 |
| Bra013979 | 753 | Up | 3.61E-36 |
| Bra004316 | 966 | Up | 7.15E-46 |
| Bra041120 | 1515 | Up | 1.42E-70 |
| Bra028625 | 501 | Up | 2.17E-22 |
| Bra036570 | 666 | Up | 7.84E-30 |
| Bra024963 | 288 | Up | 8.12E-13 |
| Bra037764 | 354 | Up | 1.01E-15 |
| Bra000640 | 963 | Up | 1.59E-39 |
| Bra009213 | 1323 | Up | 7.44E-52 |
| Bra007314 | 654 | Up | 7.04E-24 |
| Bra018725 | 1038 | Up | 4.96E-38 |
| Bra037542 | 183 | Up | 8.64E-07 |
| Bra028302 | 1560 | Up | 2.40E-56 |
| Bra033504 | 291 | Up | 2.26E-10 |
| Bra000170 | 396 | Up | 6.92E-14 |
| Bra014534 | 258 | Up | 6.26E-09 |
| Bra038417 | 648 | Up | 1.20E-21 |
| Bra037901 | 303 | Up | 5.20E-10 |
| Bra019123 | 585 | Up | 4.73E-19 |
| Bra037695 | 747 | Up | 7.05E-24 |
| Bra003821 | 360 | Up | 1.93E-11 |
| Bra002983 | 342 | Up | 9.88E-11 |
| Bra021641 | 309 | Up | 1.19E-09 |
| Bra037934 | 627 | Up | 4.74E-19 |
| Bra005843 | 1527 | Up | 7.14E-46 |
| Bra026323 | 189 | Up | 9.95E-06 |
| Bra027477 | 582 | Up | 1.86E-16 |
| Bra010291 | 849 | Up | 7.05E-24 |
| Bra034486 | 342 | Up | 1.19E-09 |
| Bra004266 | 177 | Up | 5.00E-05 |
| Bra035618 | 687 | Up | 6.14E-18 |
| Bra007411 | 345 | Up | 6.27E-09 |
| Bra018109 | 465 | Up | 8.59E-12 |
| Bra013177 | 252 | Up | 1.96E-06 |
| Bra040174 | 1293 | Up | 3.32E-30 |
| Bra036114 | 1029 | Up | 7.06E-24 |
| Bra014981 | 1431 | Up | 8.18E-33 |
| Bra021238 | 276 | Up | 1.96E-06 |
| Bra016461 | 294 | Up | 8.64E-07 |
| Bra040831 | 615 | Up | 1.60E-13 |
| Bra031844 | 234 | Up | 2.23E-05 |
| Bra032090 | 1059 | Up | 3.93E-23 |
| Bra025646 | 237 | Up | 2.23E-05 |
| Bra023815 | 309 | Up | 8.64E-07 |
| Bra035435 | 639 | Up | 1.60E-13 |
| Bra016841 | 318 | Up | 8.66E-07 |
| Bra014760 | 693 | Up | 2.96E-14 |
| Bra002962 | 1413 | Up | 4.36E-29 |
| Bra003087 | 291 | Up | 4.40E-06 |
| Bra027681 | 549 | Up | 4.38E-11 |
| Bra038117 | 732 | Up | 1.27E-14 |
| Bra020551 | 387 | Up | 7.43E-08 |
| Bra007307 | 261 | Up | 2.23E-05 |
| Bra023330 | 2955 | Up | 1.37E-58 |
| Bra017082 | 1329 | Up | 4.14E-26 |
| Bra001612 | 1092 | Up | 1.55E-20 |
| Bra034440 | 414 | Up | 7.43E-08 |
| Bra025687 | 702 | Up | 8.13E-13 |
| Bra000423 | 846 | Up | 2.36E-15 |
| Bra008329 | 222 | Up | 0.000247227 |
| Bra029918 | 303 | Up | 9.98E-06 |
| Bra040638 | 405 | Up | 1.69E-07 |
| Bra015937 | 756 | Up | 1.60E-13 |
| Bra019358 | 1065 | Up | 4.74E-19 |
| Bra017975 | 294 | Up | 2.23E-05 |
| Bra020575 | 234 | Up | 0.000246702 |
| Bra038919 | 447 | Up | 7.42E-08 |
| Bra006711 | 1080 | Up | 2.62E-18 |
| Bra007313 | 951 | Up | 4.35E-16 |
| Bra007946 | 414 | Up | 3.80E-07 |
| Bra023472 | 1374 | Up | 3.93E-23 |
| Bra022783 | 465 | Up | 7.42E-08 |
| Bra036269 | 1692 | Up | 1.34E-27 |
| Bra011428 | 1518 | Up | 1.28E-24 |
| Bra039286 | 1044 | Up | 7.94E-17 |
| Bra014397 | 1932 | Up | 2.54E-31 |
| Bra010088 | 705 | Up | 1.93E-11 |
| Bra030842 | 1347 | Up | 1.20E-21 |
| Bra022932 | 231 | Up | 0.000545632 |
| Bra036942 | 1050 | Up | 1.86E-16 |
| Bra009211 | 351 | Up | 9.95E-06 |
| Bra020021 | 969 | Up | 5.47E-15 |
| Bra021093 | 315 | Up | 5.00E-05 |
| Bra030111 | 1170 | Up | 1.44E-17 |
| Bra039764 | 1500 | Up | 2.17E-22 |
| Bra000426 | 399 | Up | 4.41E-06 |
| Bra019417 | 384 | Up | 9.96E-06 |
| Bra028639 | 519 | Up | 1.69E-07 |
| Bra015220 | 1737 | Up | 3.00E-24 |
| Bra025445 | 924 | Up | 8.12E-13 |
| Bra017373 | 807 | Up | 4.38E-11 |
| Bra039977 | 582 | Up | 7.43E-08 |
| Bra003188 | 417 | Up | 9.95E-06 |
| Bra037977 | 1239 | Up | 4.34E-16 |
| Bra027854 | 423 | Up | 9.96E-06 |
| Bra017440 | 423 | Up | 9.96E-06 |
| Bra031452 | 3558 | Up | 3.03E-46 |
| Bra004434 | 603 | Up | 7.43E-08 |
| Bra001681 | 402 | Up | 2.23E-05 |
| Bra026986 | 1395 | Up | 1.44E-17 |
| Bra008313 | 1146 | Up | 2.96E-14 |
| Bra033318 | 588 | Up | 1.69E-07 |
| Bra002329 | 564 | Up | 3.81E-07 |
| Bra008300 | 834 | Up | 2.26E-10 |
| Bra037909 | 540 | Up | 8.65E-07 |
| Bra032563 | 309 | Up | 0.000545315 |
| Bra007980 | 372 | Up | 0.000111177 |
| Bra008357 | 345 | Up | 0.000247289 |
| Bra010087 | 819 | Up | 1.19E-09 |
| Bra038098 | 978 | Up | 1.93E-11 |
| Bra034934 | 546 | Up | 1.96E-06 |
| Bra018524 | 1494 | Up | 7.95E-17 |
| Bra016930 | 981 | Up | 4.38E-11 |
| Bra030453 | 627 | Up | 3.81E-07 |
| Bra034066 | 990 | Up | 4.38E-11 |
| Bra002410 | 861 | Up | 1.19E-09 |
| Bra041028 | 1029 | Up | 1.93E-11 |
| Bra034065 | 963 | Up | 9.88E-11 |
| Bra019592 | 1035 | Up | 1.93E-11 |
| Bra011124 | 906 | Up | 1.19E-09 |
| Bra030298 | 2766 | Up | 4.37E-29 |
| Bra027357 | 1122 | Up | 8.59E-12 |
| Bra024553 | 534 | Up | 9.95E-06 |
| Bra015572 | 393 | Up | 0.000246856 |
| Bra029092 | 465 | Up | 4.99E-05 |
| Bra009159 | 789 | Up | 3.26E-08 |
| Bra022797 | 435 | Up | 0.000111147 |
| Bra017790 | 960 | Up | 1.19E-09 |
| Bra005807 | 456 | Up | 0.000111028 |
| Bra007933 | 459 | Up | 0.000111058 |
| Bra039867 | 393 | Up | 0.000546586 |
| Bra038396 | 1104 | Up | 2.26E-10 |
| Bra021969 | 558 | Up | 2.23E-05 |
| Bra036322 | 681 | Up | 1.96E-06 |
| Bra037593 | 1140 | Up | 2.26E-10 |
| Bra023725 | 408 | Up | 0.000545252 |
| Bra023916 | 414 | Up | 0.000545886 |
| Bra039521 | 456 | Up | 0.000247444 |
| Bra020426 | 540 | Up | 4.99E-05 |
| Bra023100 | 420 | Up | 0.000546522 |
| Bra021124 | 768 | Up | 8.65E-07 |
| Bra033113 | 2058 | Up | 1.44E-17 |
| Bra004765 | 816 | Up | 3.81E-07 |
| Bra028540 | 516 | Up | 0.000111043 |
| Bra035808 | 1053 | Up | 6.27E-09 |
| Bra014905 | 837 | Up | 3.81E-07 |
| Bra016879 | 1455 | Up | 3.91E-12 |
| Bra004012 | 1617 | Up | 3.66E-13 |
| Bra005748 | 4050 | Up | 3.47E-33 |
| Bra034527 | 675 | Up | 9.97E-06 |
| Bra039686 | 678 | Up | 9.97E-06 |
| Bra032138 | 2403 | Up | 2.02E-19 |
| Bra005169 | 1860 | Up | 5.47E-15 |
| Bra005899 | 1005 | Up | 3.26E-08 |
| Bra014511 | 2799 | Up | 2.17E-22 |
| Bra031041 | 645 | Up | 2.23E-05 |
| Bra038622 | 1107 | Up | 6.27E-09 |
| Bra005937 | 555 | Up | 0.000111014 |
| Bra005301 | 747 | Up | 4.41E-06 |
| Bra017429 | 1482 | Up | 1.93E-11 |
| Bra031050 | 1197 | Up | 2.73E-09 |
| Bra018201 | 1395 | Up | 9.88E-11 |
| Bra008822 | 828 | Up | 1.96E-06 |
| Bra012335 | 489 | Up | 0.000545823 |
| Bra007697 | 936 | Up | 3.80E-07 |
| Bra006051 | 594 | Up | 0.000111296 |
| Bra029015 | 645 | Up | 5.00E-05 |
| Bra003792 | 501 | Up | 0.000546013 |
| Bra001561 | 1107 | Up | 3.26E-08 |
| Bra020675 | 906 | Up | 8.66E-07 |
| Bra032340 | 822 | Up | 4.41E-06 |
| Bra021438 | 774 | Up | 9.98E-06 |
| Bra019883 | 1398 | Up | 5.20E-10 |
| Bra007989 | 1554 | Up | 4.38E-11 |
| Bra031136 | 885 | Up | 1.96E-06 |
| Bra017878 | 573 | Up | 0.00024732 |
| Bra015645 | 630 | Up | 0.000111266 |
| Bra020207 | 840 | Up | 4.40E-06 |
| Bra023143 | 687 | Up | 5.00E-05 |
| Bra036158 | 900 | Up | 1.96E-06 |
| Bra031334 | 636 | Up | 0.000111117 |
| Bra002546 | 1275 | Up | 6.27E-09 |
| Bra017756 | 915 | Up | 1.96E-06 |
| Bra015461 | 1920 | Up | 8.13E-13 |
| Bra010958 | 1761 | Up | 8.60E-12 |
| Bra034068 | 555 | Up | 0.000546268 |
| Bra021304 | 678 | Up | 0.000110999 |
| Bra008084 | 624 | Up | 0.000247196 |
| Bra038068 | 1197 | Up | 7.43E-08 |
| Bra008850 | 630 | Up | 0.000247413 |
| Bra041140 | 1155 | Up | 1.69E-07 |
| Bra031809 | 579 | Up | 0.000544808 |
| Bra039646 | 1332 | Up | 1.43E-08 |
| Bra003631 | 642 | Up | 0.000247475 |
| Bra021947 | 1284 | Up | 3.26E-08 |
| Bra010416 | 888 | Up | 9.95E-06 |
| Bra031528 | 657 | Up | 0.000247165 |
| Bra000548 | 1320 | Up | 3.26E-08 |
| Bra009339 | 1020 | Up | 1.96E-06 |
| Bra035814 | 1509 | Up | 2.73E-09 |
| Bra014832 | 918 | Up | 9.97E-06 |
| Bra012707 | 741 | Up | 0.000111192 |
| Bra020858 | 930 | Up | 9.97E-06 |
| Bra034664 | 1119 | Up | 8.64E-07 |
| Bra038638 | 1623 | Up | 1.19E-09 |
| Bra014467 | 1947 | Up | 1.93E-11 |
| Bra019736 | 882 | Up | 2.23E-05 |
| Bra000582 | 822 | Up | 5.00E-05 |
| Bra036260 | 642 | Up | 0.000546077 |
| Bra012219 | 645 | Up | 0.000546841 |
| Bra030915 | 1161 | Up | 8.65E-07 |
| Bra009874 | 1161 | Up | 8.65E-07 |
| Bra010290 | 843 | Up | 5.00E-05 |
| Bra012532 | 921 | Up | 2.23E-05 |
| Bra008446 | 2973 | Up | 1.85E-16 |
| Bra009072 | 2025 | Up | 4.38E-11 |
| Bra037403 | 750 | Up | 0.000247382 |
| Bra033535 | 750 | Up | 0.000246733 |
| Bra039972 | 753 | Up | 0.000247351 |
| Bra026585 | 894 | Up | 4.99E-05 |
| Bra032367 | 693 | Up | 0.000545696 |
| Bra034443 | 972 | Up | 2.23E-05 |
| Bra028829 | 972 | Up | 2.23E-05 |
| Bra025163 | 1683 | Up | 6.27E-09 |
| Bra020078 | 918 | Up | 4.99E-05 |
| Bra028167 | 804 | Up | 0.000246887 |
| Bra006526 | 1320 | Up | 8.64E-07 |
| Bra015936 | 1041 | Up | 2.23E-05 |
| Bra034754 | 972 | Up | 5.00E-05 |
| Bra038604 | 978 | Up | 5.00E-05 |
| Bra009323 | 1140 | Up | 9.97E-06 |
| Bra000870 | 1143 | Up | 9.97E-06 |
| Bra022653 | 1470 | Up | 3.80E-07 |
| Bra017948 | 1497 | Up | 3.81E-07 |
| Bra021024 | 879 | Up | 0.000247506 |
| Bra035809 | 1758 | Up | 3.26E-08 |
| Bra005916 | 1044 | Up | 4.99E-05 |
| Bra008866 | 885 | Up | 0.000246918 |
| Bra021670 | 1059 | Up | 5.00E-05 |
| Bra006334 | 909 | Up | 0.000246825 |
| Bra008527 | 993 | Up | 0.000111088 |
| Bra023463 | 828 | Up | 0.000545505 |
| Bra007263 | 1671 | Up | 1.69E-07 |
| Bra020197 | 843 | Up | 0.000544872 |
| Bra028133 | 1191 | Up | 2.23E-05 |
| Bra028111 | 2568 | Up | 4.38E-11 |
| Bra017186 | 1113 | Up | 5.00E-05 |
| Bra029840 | 1326 | Up | 9.98E-06 |
| Bra024209 | 888 | Up | 0.000546141 |
| Bra029555 | 1257 | Up | 2.23E-05 |
| Bra018334 | 1089 | Up | 0.000111221 |
| Bra033873 | 1653 | Up | 8.65E-07 |
| Bra020429 | 1575 | Up | 1.96E-06 |
| Bra040421 | 1116 | Up | 0.000111206 |
| Bra034825 | 939 | Up | 0.000546777 |
| Bra015757 | 1221 | Up | 4.99E-05 |
| Bra031990 | 1410 | Up | 9.95E-06 |
| Bra010213 | 1881 | Up | 1.69E-07 |
| Bra021780 | 1788 | Up | 3.80E-07 |
| Bra036336 | 1512 | Up | 4.40E-06 |
| Bra004617 | 954 | Up | 0.000545062 |
| Bra025962 | 1161 | Up | 0.000111236 |
| Bra028422 | 984 | Up | 0.000545442 |
| Bra038573 | 3660 | Up | 1.60E-13 |
| Bra011255 | 3585 | Up | 3.66E-13 |
| Bra036282 | 2436 | Up | 6.27E-09 |
| Bra020104 | 1422 | Up | 2.23E-05 |
| Bra000284 | 1443 | Up | 2.23E-05 |
| Bra023377 | 1461 | Up | 2.23E-05 |
| Bra000058 | 1053 | Up | 0.000545759 |
| Bra017480 | 1161 | Up | 0.000246795 |
| Bra020871 | 1056 | Up | 0.000544935 |
| Bra026116 | 1077 | Up | 0.000546969 |
| Bra024691 | 2301 | Up | 7.42E-08 |
| Bra031107 | 3003 | Up | 5.20E-10 |
| Bra001609 | 1122 | Up | 0.00054595 |
| Bra020379 | 1251 | Up | 0.000247041 |
| Bra021798 | 1149 | Up | 0.000544998 |
| Bra016430 | 3108 | Up | 5.20E-10 |
| Bra029382 | 1503 | Up | 4.99E-05 |
| Bra022314 | 2094 | Up | 8.66E-07 |
| Bra009494 | 1182 | Up | 0.00054665 |
| Bra019189 | 2502 | Up | 7.43E-08 |
| Bra026160 | 1440 | Up | 0.000111073 |
| Bra005529 | 2166 | Up | 8.66E-07 |
| Bra025585 | 1458 | Up | 0.000111162 |
| Bra033451 | 768 | Up | 0 |
| Bra039163 | 1368 | Up | 0.000247072 |
| Bra035991 | 1755 | Up | 2.23E-05 |
| Bra035818 | 1548 | Up | 0.000111281 |
| Bra013664 | 1935 | Up | 9.96E-06 |
| Bra028808 | 1971 | Up | 9.96E-06 |
| Bra014718 | 1872 | Up | 2.23E-05 |
| Bra021728 | 1347 | Up | 0.000544745 |
| Bra003019 | 1491 | Up | 0.000247134 |
| Bra027431 | 1560 | Up | 0.000246764 |
| Bra016907 | 1584 | Up | 0.000247568 |
| Bra018888 | 1758 | Up | 0.000111102 |
| Bra025191 | 1470 | Up | 0.000545125 |
| Bra008681 | 1476 | Up | 0.000545379 |
| Bra030170 | 2301 | Up | 9.96E-06 |
| Bra006194 | 1722 | Up | 0.00024698 |
| Bra004199 | 1884 | Up | 0.000111132 |
| Bra011023 | 2070 | Up | 4.99E-05 |
| Bra032180 | 1755 | Up | 0.000246949 |
| Bra003081 | 2277 | Up | 2.23E-05 |
| Bra015352 | 1857 | Up | 0.000247258 |
| Bra022968 | 3063 | Up | 8.65E-07 |
| Bra009269 | 1944 | Up | 0.000247103 |
| Bra013662 | 2157 | Up | 0.000111251 |
| Bra030994 | 3225 | Up | 1.96E-06 |
| Bra020259 | 1938 | Up | 0.000546905 |
| Bra003860 | 1965 | Up | 0.000545569 |
| Bra035440 | 2169 | Up | 0.000247011 |
| Bra016311 | 3918 | Up | 3.80E-07 |
| Bra031186 | 3684 | Up | 4.40E-06 |
| Bra015587 | 2541 | Up | 0.000247537 |
| Bra007756 | 3768 | Up | 4.40E-06 |
| Bra039077 | 2367 | Up | 0.000545188 |
| Bra030416 | 2688 | Up | 0.000546714 |
| Bra033384 | 3384 | Up | 0.000546459 |
| Bra001239 | 5724 | Up | 9.98E-06 |
| Bra000333 | 3882 | Up | 0.000546331 |
| Bra020470 | 807 | Up | 0 |
| Bra032058 | 4215 | Up | 0.000546395 |
| Bra022603 | 867 | Up | 7.68E-106 |
| Bra027096 | 1065 | Up | 3.59E-87 |
| Bra008138 | 5616 | Up | 0.000546204 |
| Bra023065 | 4290 | Up | 9.61E-58 |
| Bra036938 | 774 | Up | 1.62E-55 |
| Bra017160 | 2373 | Up | 6.50E-53 |
| Bra036642 | 660 | Up | 2.85E-212 |
| Bra027359 | 1131 | Up | 0 |
| Bra022707 | 399 | Up | 1.01E-47 |
| Bra016073 | 672 | Up | 1.39E-186 |
| Bra026562 | 3990 | Up | 1.20E-43 |
| Bra024174 | 462 | Up | 2.81E-43 |
| Bra003970 | 705 | Up | 1.41E-39 |
| Bra013863 | 639 | Up | 0 |
| Bra003263 | 1113 | Up | 7.97E-195 |
| Bra007507 | 1542 | Up | 1.60E-35 |
| Bra038898 | 1194 | Up | 1.60E-35 |
| Bra034677 | 1107 | Up | 3.16E-239 |
| Bra039195 | 1881 | Up | 6.08E-33 |
| Bra023798 | 585 | Up | 0 |
| Bra000806 | 429 | Up | 7.77E-32 |
| Bra011711 | 1107 | Up | 1.82E-31 |
| Bra026525 | 1647 | Up | 1.85E-148 |
| Bra016895 | 912 | Up | 1.07E-26 |
| Bra007212 | 1731 | Up | 1.27E-236 |
| Bra015094 | 1416 | Up | 1.36E-25 |
| Bra039051 | 2205 | Up | 2.23E-102 |
| Bra011130 | 1350 | Up | 3.16E-25 |
| Bra028983 | 2031 | Up | 7.36E-25 |
| Bra031515 | 1671 | Up | 6.86E-94 |
| Bra015873 | 1395 | Up | 1.09E-66 |
| Bra003086 | 1191 | Up | 1.43E-21 |
| Bra036160 | 423 | Up | 1.43E-21 |
| Bra024088 | 849 | Up | 7.07E-123 |
| Bra004819 | 1197 | Up | 4.08E-20 |
| Bra029349 | 843 | Up | 9.48E-20 |
| Bra019187 | 765 | Up | 9.48E-20 |
| Bra040704 | 1104 | Up | 2.04E-215 |
| Bra030386 | 1581 | Up | 2.57E-113 |
| Bra007482 | 1062 | Up | 1.17E-18 |
| Bra008873 | 1461 | Up | 2.70E-18 |
| Bra017551 | 1815 | Up | 3.44E-72 |
| Bra016271 | 3144 | Up | 4.05E-234 |
| Bra016025 | 876 | Up | 6.22E-18 |
| Bra003426 | 1389 | Up | 0 |
| Bra029456 | 510 | Up | 1.82E-270 |
| Bra026262 | 900 | Up | 7.64E-17 |
| Bra022244 | 1014 | Up | 2.54E-33 |
| Bra026653 | 456 | Up | 1.75E-16 |
| Bra031321 | 768 | Up | 4.02E-16 |
| Bra017890 | 273 | Up | 4.02E-16 |
| Bra003996 | 600 | Up | 9.18E-16 |
| Bra005345 | 936 | Up | 1.25E-123 |
| Bra029306 | 1122 | Up | 2.10E-15 |
| Bra019689 | 2229 | Up | 2.07E-30 |
| Bra025449 | 237 | Up | 1.09E-14 |
| Bra001888 | 660 | Up | 1.09E-14 |
| Bra022318 | 1257 | Up | 2.47E-14 |
| Bra006511 | 1911 | Up | 7.14E-28 |
| Bra031662 | 450 | Up | 1.96E-41 |
| Bra000775 | 501 | Up | 0 |
| Bra002619 | 918 | Up | 1.65E-27 |
| Bra036573 | 1053 | Up | 2.99E-176 |
| Bra018573 | 972 | Up | 6.36E-54 |
| Bra038370 | 1086 | Up | 2.89E-13 |
| Bra028889 | 1896 | Up | 2.89E-13 |
| Bra013449 | 1518 | Up | 1.10E-65 |
| Bra003696 | 480 | Up | 6.79E-39 |
| Bra033830 | 582 | Up | 6.31E-13 |
| Bra015736 | 1170 | Up | 1.06E-25 |
| Bra017083 | 1098 | Up | 6.32E-13 |
| Bra009427 | 534 | Up | 1.36E-12 |
| Bra015551 | 597 | Up | 1.36E-12 |
| Bra040920 | 1638 | Up | 1.29E-24 |
| Bra010644 | 1557 | Up | 6.21E-12 |
| Bra013683 | 1986 | Up | 6.21E-12 |
| Bra022161 | 1353 | Up | 1.56E-23 |
| Bra021681 | 1053 | Up | 1.72E-126 |
| Bra014758 | 504 | Up | 1.35E-11 |
| Bra030641 | 1059 | Up | 1.35E-11 |
| Bra017086 | 1464 | Up | 1.35E-11 |
| Bra013426 | 810 | Up | 3.42E-34 |
| Bra022833 | 2232 | Up | 8.21E-23 |
| Bra001574 | 327 | Up | 2.99E-11 |
| Bra016432 | 1125 | Up | 4.30E-22 |
| Bra016669 | 4314 | Up | 3.94E-44 |
| Bra032139 | 660 | Up | 3.95E-44 |
| Bra005831 | 963 | Up | 9.84E-22 |
| Bra010116 | 690 | Up | 7.82E-108 |
| Bra036465 | 1440 | Up | 2.17E-64 |
| Bra021572 | 1539 | Up | 6.59E-11 |
| Bra007144 | 546 | Up | 6.59E-11 |
| Bra035574 | 348 | Up | 1.15E-31 |
| Bra024100 | 2847 | Up | 2.85E-52 |
| Bra001640 | 738 | Up | 1.46E-10 |
| Bra032871 | 1185 | Up | 3.04E-41 |
| Bra039555 | 1317 | Up | 1.46E-10 |
| Bra033407 | 582 | Up | 1.46E-10 |
| Bra033071 | 504 | Up | 1.46E-10 |
| Bra015307 | 621 | Up | 1.46E-10 |
| Bra030216 | 1509 | Up | 1.68E-61 |
| Bra010283 | 753 | Up | 1.88E-71 |
| Bra015240 | 699 | Up | 3.26E-10 |
| Bra020450 | 966 | Up | 3.26E-10 |
| Bra001651 | 747 | Up | 3.26E-10 |
| Bra017707 | 444 | Up | 3.26E-10 |
| Bra030285 | 795 | Up | 3.74E-29 |
| Bra017133 | 2064 | Up | 3.18E-144 |
| Bra031486 | 1011 | Up | 7.27E-10 |
| Bra000019 | 948 | Up | 8.51E-29 |
| Bra013199 | 2022 | Up | 7.27E-10 |
| Bra013788 | 1605 | Up | 3.15E-19 |
| Bra025934 | 2514 | Up | 1.94E-28 |
| Bra008333 | 2721 | Up | 9.84E-56 |
| Bra029163 | 786 | Up | 1.61E-09 |
| Bra026621 | 1482 | Up | 3.73E-18 |
| Bra039531 | 2904 | Up | 3.59E-09 |
| Bra034522 | 1365 | Up | 3.59E-09 |
| Bra025522 | 1128 | Up | 8.45E-18 |
| Bra037254 | 2949 | Up | 3.85E-35 |
| Bra009613 | 1323 | Up | 6.20E-26 |
| Bra027391 | 2313 | Up | 2.00E-34 |
| Bra000889 | 1185 | Up | 7.97E-09 |
| Bra000184 | 639 | Up | 7.97E-09 |
| Bra017699 | 1101 | Up | 7.97E-09 |
| Bra001921 | 1458 | Up | 1.65E-75 |
| Bra020564 | 1632 | Up | 3.22E-25 |
| Bra003699 | 816 | Up | 4.09E-175 |
| Bra002283 | 534 | Up | 8.95E-58 |
| Bra021153 | 1236 | Up | 1.76E-08 |
| Bra035466 | 1224 | Up | 3.21E-88 |
| Bra011776 | 1125 | Up | 3.87E-08 |
| Bra039702 | 792 | Up | 3.87E-08 |
| Bra015384 | 1632 | Up | 2.53E-15 |
| Bra006397 | 1509 | Up | 8.48E-08 |
| Bra031002 | 861 | Up | 8.48E-08 |
| Bra027059 | 798 | Up | 8.48E-08 |
| Bra018953 | 567 | Up | 1.27E-14 |
| Bra000753 | 2385 | Up | 1.26E-14 |
| Bra024500 | 957 | Up | 3.77E-100 |
| Bra035187 | 1185 | Up | 1.86E-07 |
| Bra002189 | 1458 | Up | 2.24E-28 |
| Bra024707 | 1344 | Up | 1.86E-07 |
| Bra005478 | 336 | Up | 1.86E-07 |
| Bra022302 | 525 | Up | 2.58E-21 |
| Bra035394 | 1353 | Up | 1.86E-07 |
| Bra013092 | 2586 | Up | 1.86E-07 |
| Bra021672 | 1086 | Up | 4.45E-35 |
| Bra033788 | 1320 | Up | 6.35E-14 |
| Bra038676 | 621 | Up | 6.35E-14 |
| Bra010209 | 1437 | Up | 1.99E-47 |
| Bra013584 | 1617 | Up | 4.06E-07 |
| Bra018131 | 1173 | Up | 1.42E-13 |
| Bra035033 | 1809 | Up | 4.05E-07 |
| Bra030197 | 258 | Up | 4.06E-07 |
| Bra010078 | 369 | Up | 4.05E-07 |
| Bra011742 | 333 | Up | 4.06E-07 |
| Bra038872 | 3330 | Up | 4.05E-07 |
| Bra018969 | 1587 | Up | 6.67E-20 |
| Bra014936 | 1842 | Up | 2.66E-39 |
| Bra030550 | 1509 | Up | 3.11E-13 |
| Bra014643 | 852 | Up | 3.11E-13 |
| Bra041038 | 621 | Up | 3.03E-91 |
| Bra000427 | 1479 | Up | 6.74E-26 |
| Bra014749 | 1782 | Up | 8.85E-07 |
| Bra022710 | 960 | Up | 8.85E-07 |
| Bra019920 | 1710 | Up | 7.79E-31 |
| Bra009395 | 1245 | Up | 1.93E-06 |
| Bra008651 | 999 | Up | 1.93E-06 |
| Bra027842 | 465 | Up | 1.93E-06 |
| Bra017927 | 426 | Up | 8.84E-30 |
| Bra033148 | 1116 | Up | 6.19E-12 |
| Bra022599 | 546 | Up | 4.16E-06 |
| Bra039603 | 1095 | Up | 1.32E-11 |
| Bra023863 | 1608 | Up | 1.32E-11 |
| Bra014107 | 1902 | Up | 4.16E-06 |
| Bra028682 | 1173 | Up | 1.32E-11 |
| Bra034062 | 3117 | Up | 4.16E-06 |
| Bra004571 | 1854 | Up | 9.85E-23 |
| Bra036090 | 825 | Up | 4.16E-06 |
| Bra016676 | 1617 | Up | 1.32E-11 |
| Bra033350 | 297 | Up | 6.73E-62 |
| Bra020088 | 798 | Up | 1.34E-50 |
| Bra004101 | 2232 | Up | 6.76E-56 |
| Bra026902 | 1143 | Up | 9.58E-17 |
| Bra015652 | 1539 | Up | 2.86E-11 |
| Bra001870 | 1674 | Up | 2.86E-11 |
| Bra013828 | 465 | Up | 2.86E-11 |
| Bra025715 | 699 | Up | 2.86E-11 |
| Bra021209 | 516 | Up | 2.86E-11 |
| Bra002347 | 4095 | Up | 4.68E-153 |
| Bra009894 | 768 | Up | 2.51E-27 |
| Bra017093 | 1605 | Up | 1.38E-97 |
| Bra023025 | 1995 | Up | 2.13E-16 |
| Bra006710 | 1050 | Up | 1.10E-21 |
| Bra025367 | 507 | Up | 6.17E-11 |
| Bra034816 | 609 | Up | 8.99E-06 |
| Bra028830 | 639 | Up | 8.99E-06 |
| Bra034392 | 951 | Up | 8.99E-06 |
| Bra013522 | 2541 | Up | 4.73E-16 |
| Bra037858 | 252 | Up | 6.17E-11 |
| Bra028313 | 1227 | Up | 8.59E-42 |
| Bra018307 | 309 | Up | 1.05E-15 |
| Bra004175 | 1062 | Up | 3.16E-25 |
| Bra034161 | 2568 | Up | 3.15E-25 |
| Bra016258 | 456 | Up | 1.93E-05 |
| Bra002595 | 570 | Up | 1.93E-05 |
| Bra021299 | 294 | Up | 1.93E-05 |
| Bra002210 | 1272 | Up | 1.93E-05 |
| Bra027775 | 3078 | Up | 1.93E-05 |
| Bra012270 | 1131 | Up | 2.91E-10 |
| Bra025434 | 855 | Up | 1.93E-05 |
| Bra011983 | 399 | Up | 2.91E-10 |
| Bra026122 | 1080 | Up | 1.93E-05 |
| Bra028710 | 675 | Up | 5.90E-91 |
| Bra003737 | 969 | Up | 6.34E-10 |
| Bra001562 | 1539 | Up | 2.30E-33 |
| Bra029172 | 333 | Up | 0 |
| Bra018065 | 1137 | Up | 4.04E-290 |
| Bra026695 | 1023 | Up | 4.11E-05 |
| Bra035662 | 2700 | Up | 4.11E-05 |
| Bra038223 | 1383 | Up | 4.11E-05 |
| Bra028553 | 441 | Up | 4.11E-05 |
| Bra024760 | 801 | Up | 4.11E-05 |
| Bra008131 | 1005 | Up | 4.11E-05 |
| Bra032770 | 1692 | Up | 1.73E-151 |
| Bra029017 | 387 | Up | 1.79E-81 |
| Bra002907 | 309 | Up | 4.13E-40 |
| Bra020622 | 1209 | Up | 2.98E-09 |
| Bra009895 | 1053 | Up | 2.98E-48 |
| Bra012433 | 933 | Up | 2.38E-26 |
| Bra014762 | 843 | Up | 3.27E-47 |
| Bra032369 | 1341 | Up | 8.73E-05 |
| Bra022952 | 531 | Up | 6.46E-09 |
| Bra003722 | 624 | Up | 8.73E-05 |
| Bra012389 | 912 | Up | 5.54E-13 |
| Bra022267 | 1056 | Up | 1.17E-25 |
| Bra013470 | 567 | Up | 8.73E-05 |
| Bra020820 | 2034 | Up | 1.16E-12 |
| Bra028399 | 1080 | Up | 1.16E-12 |
| Bra004390 | 933 | Up | 1.12E-82 |
| Bra025816 | 2196 | Up | 6.80E-41 |
| Bra007225 | 1305 | Up | 1.71E-16 |
| Bra040549 | 1347 | Up | 1.39E-08 |
| Bra013123 | 486 | Up | 0 |
| Bra013254 | 3666 | Up | 1.60E-28 |
| Bra001453 | 729 | Up | 1.70E-56 |
| Bra030217 | 1509 | Up | 2.41E-12 |
| Bra039368 | 4434 | Up | 2.41E-12 |
| Bra030773 | 1128 | Up | 3.76E-16 |
| Bra033146 | 1122 | Up | 4.80E-20 |
| Bra031770 | 618 | Up | 0.000183816 |
| Bra026027 | 2289 | Up | 0.000183745 |
| Bra029674 | 882 | Up | 0.000183697 |
| Bra001178 | 837 | Up | 0.000183768 |
| Bra005526 | 861 | Up | 2.25E-70 |
| Bra020354 | 921 | Up | 0.00018365 |
| Bra005490 | 330 | Up | 0.000183603 |
| Bra025433 | 1728 | Up | 0.000183721 |
| Bra030178 | 834 | Up | 0.000183627 |
| Bra025828 | 615 | Up | 0.000183674 |
| Bra030018 | 825 | Up | 0.000183792 |
| Bra002421 | 867 | Up | 0.000183579 |
| Bra007947 | 477 | Up | 0 |
| Bra024736 | 1119 | Up | 0 |
| Bra000261 | 2394 | Up | 1.80E-15 |
| Bra009633 | 1635 | Up | 5.34E-205 |
| Bra000132 | 921 | Up | 1.02E-11 |
| Bra039700 | 381 | Up | 1.02E-11 |
| Bra011893 | 744 | Up | 2.33E-52 |
| Bra031209 | 312 | Up | 5.17E-30 |
| Bra016591 | 837 | Up | 8.15E-60 |
| Bra036992 | 2070 | Up | 3.88E-15 |
| Bra018400 | 789 | Up | 1.85E-48 |
| Bra037614 | 879 | Up | 2.17E-11 |
| Bra024481 | 771 | Up | 3.22E-44 |
| Bra016002 | 645 | Up | 8.75E-59 |
| Bra009169 | 2559 | Up | 8.46E-15 |
| Bra012733 | 1107 | Up | 2.46E-18 |
| Bra032189 | 1281 | Up | 5.49E-29 |
| Bra024340 | 2418 | Up | 5.49E-29 |
| Bra006238 | 753 | Up | 0.000383008 |
| Bra013478 | 1569 | Up | 0.000382778 |
| Bra014953 | 1410 | Up | 0.00038287 |
| Bra025344 | 1494 | Up | 1.36E-07 |
| Bra008644 | 996 | Up | 4.58E-11 |
| Bra040618 | 591 | Up | 0.000382824 |
| Bra009432 | 624 | Up | 1.36E-07 |
| Bra005313 | 510 | Up | 0.000382962 |
| Bra012528 | 1986 | Up | 0.000382732 |
| Bra008591 | 1125 | Up | 0.000382916 |
| Bra028597 | 672 | Up | 1.52E-21 |
| Bra038667 | 678 | Up | 0 |
| Bra009221 | 1077 | Up | 7.58E-179 |
| Bra020140 | 4044 | Up | 6.30E-70 |
| Bra034862 | 402 | Up | 1.18E-17 |
| Bra015874 | 732 | Up | 1.74E-146 |
| Bra040524 | 273 | Up | 2.83E-38 |
| Bra008344 | 1134 | Up | 9.72E-11 |
| Bra014248 | 2145 | Up | 2.57E-17 |
| Bra003598 | 2376 | Up | 2.57E-17 |
| Bra026864 | 2433 | Up | 4.49E-24 |
| Bra034327 | 1794 | Up | 2.89E-07 |
| Bra011758 | 1500 | Up | 2.89E-07 |
| Bra024281 | 3738 | Up | 2.89E-07 |
| Bra030566 | 1080 | Up | 2.89E-07 |
| Bra017974 | 261 | Up | 2.89E-07 |
| Bra020493 | 1407 | Up | 2.89E-07 |
| Bra037095 | 1068 | Up | 2.89E-07 |
| Bra026803 | 765 | Up | 2.89E-07 |
| Bra030806 | 783 | Up | 4.80E-34 |
| Bra036601 | 1176 | Up | 0.000800903 |
| Bra010788 | 1518 | Up | 6.28E-26 |
| Bra033921 | 1137 | Up | 0.000799458 |
| Bra011710 | 996 | Up | 0.000799278 |
| Bra027837 | 387 | Up | 1.64E-19 |
| Bra027873 | 1524 | Up | 0.000799368 |
| Bra033674 | 1107 | Up | 0.000799548 |
| Bra004139 | 414 | Up | 4.43E-10 |
| Bra029545 | 888 | Up | 0.000799098 |
| Bra003941 | 762 | Up | 0.000800632 |
| Bra012364 | 480 | Up | 6.11E-07 |
| Bra006282 | 696 | Up | 0.00080027 |
| Bra028928 | 1059 | Up | 0.000799909 |
| Bra010560 | 240 | Up | 0.000799639 |
| Bra016580 | 588 | Up | 0.000800813 |
| Bra008121 | 357 | Up | 0.00080009 |
| Bra022422 | 3021 | Up | 0.0008 |
| Bra027144 | 606 | Up | 0.000799729 |
| Bra000713 | 1053 | Up | 3.96E-13 |
| Bra009258 | 831 | Up | 0.000799188 |
| Bra008921 | 2097 | Up | 0.000799008 |
| Bra038153 | 885 | Up | 0.000800722 |
| Bra014254 | 1500 | Up | 0.000800451 |
| Bra022033 | 954 | Up | 0.00080018 |
| Bra031028 | 2046 | Up | 0.000800541 |
| Bra000923 | 1080 | Up | 0.000800361 |
| Bra012524 | 1083 | Up | 0.000799819 |
| Bra007825 | 1728 | Up | 5.73E-16 |
| Bra001564 | 963 | Up | 5.73E-16 |
| Bra030337 | 1092 | Up | 6.81E-60 |
| Bra008038 | 1344 | Up | 5.90E-41 |
| Bra036546 | 1164 | Up | 1.62E-135 |
| Bra040469 | 525 | Up | 6.36E-208 |
| Bra034036 | 2571 | Up | 1.29E-06 |
| Bra039532 | 2880 | Up | 1.29E-06 |
| Bra025995 | 648 | Up | 1.29E-06 |
| Bra015133 | 1452 | Up | 1.29E-06 |
| Bra030410 | 243 | Up | 1.29E-06 |
| Bra000752 | 1581 | Up | 2.01E-09 |
| Bra010794 | 393 | Up | 4.62E-36 |
| Bra005688 | 861 | Up | 3.66E-18 |
| Bra000122 | 1653 | Up | 4.63E-36 |
| Bra017856 | 1998 | Up | 6.96E-48 |
| Bra003959 | 1122 | Up | 9.29E-51 |
| Bra014262 | 1272 | Up | 8.41E-42 |
| Bra024328 | 621 | Up | 5.70E-15 |
| Bra033911 | 591 | Up | 1.78E-88 |
| Bra003208 | 2124 | Up | 4.36E-197 |
| Bra019959 | 567 | Up | 7.65E-32 |
| Bra001454 | 1416 | Up | 5.66E-29 |
| Bra033904 | 1773 | Up | 2.71E-06 |
| Bra037954 | 1152 | Up | 4.07E-40 |
| Bra013714 | 1278 | Up | 1.21E-132 |
| Bra023635 | 1410 | Up | 1.43E-11 |
| Bra026860 | 939 | Up | 7.95E-17 |
| Bra017700 | 1104 | Up | 9.01E-09 |
| Bra027440 | 834 | Up | 9.01E-09 |
| Bra034691 | 480 | Up | 9.01E-09 |
| Bra017360 | 2319 | Up | 0 |
| Bra037112 | 2277 | Up | 9.22E-207 |
| Bra034180 | 1320 | Up | 5.62E-165 |
| Bra006714 | 621 | Up | 1.93E-38 |
| Bra005486 | 957 | Up | 9.17E-25 |
| Bra011833 | 831 | Up | 5.68E-06 |
| Bra036040 | 738 | Up | 5.68E-06 |
| Bra000282 | 819 | Up | 5.68E-06 |
| Bra004687 | 711 | Up | 5.68E-06 |
| Bra016103 | 1527 | Up | 4.98E-19 |
| Bra013214 | 1431 | Up | 1.20E-13 |
| Bra030414 | 2580 | Up | 1.20E-13 |
| Bra020007 | 3147 | Up | 2.80E-61 |
| Bra002895 | 1263 | Up | 1.90E-08 |
| Bra013213 | 2916 | Up | 1.90E-08 |
| Bra001988 | 1056 | Up | 1.07E-18 |
| Bra011890 | 1170 | Up | 6.22E-11 |
| Bra029445 | 1548 | Up | 5.43E-52 |
| Bra018579 | 837 | Up | 1.18E-05 |
| Bra003448 | 1275 | Up | 4.95E-18 |
| Bra012471 | 990 | Up | 3.97E-08 |
| Bra005021 | 1329 | Up | 3.97E-08 |
| Bra030281 | 1473 | Up | 1.18E-05 |
| Bra013569 | 822 | Up | 3.97E-08 |
| Bra017785 | 309 | Up | 1.18E-05 |
| Bra025430 | 1485 | Up | 1.18E-05 |
| Bra027339 | 1410 | Up | 2.52E-50 |
| Bra011305 | 945 | Up | 3.55E-15 |
| Bra005312 | 1485 | Up | 1.07E-12 |
| Bra008541 | 2064 | Up | 2.72E-10 |
| Bra008579 | 579 | Up | 8.29E-08 |
| Bra010578 | 453 | Up | 8.29E-08 |
| Bra027257 | 831 | Up | 7.49E-15 |
| Bra026461 | 1083 | Up | 8.29E-08 |
| Bra029023 | 1827 | Up | 2.44E-05 |
| Bra032371 | 294 | Up | 2.44E-05 |
| Bra035640 | 1557 | Up | 2.44E-05 |
| Bra025274 | 1239 | Up | 2.44E-05 |
| Bra038088 | 1497 | Up | 5.72E-10 |
| Bra025765 | 1464 | Up | 2.44E-05 |
| Bra038924 | 435 | Up | 2.44E-05 |
| Bra010583 | 558 | Up | 2.44E-05 |
| Bra024822 | 657 | Up | 2.44E-05 |
| Bra013922 | 864 | Up | 2.44E-05 |
| Bra037958 | 792 | Up | 0 |
| Bra028995 | 1131 | Up | 1.73E-07 |
| Bra038546 | 963 | Up | 2.04E-38 |
| Bra000474 | 675 | Up | 3.89E-224 |
| Bra018247 | 912 | Up | 5.00E-05 |
| Bra017572 | 2001 | Up | 5.00E-05 |
| Bra014675 | 486 | Up | 3.57E-07 |
| Bra032374 | 1404 | Up | 9.79E-16 |
| Bra009105 | 834 | Up | 2.49E-09 |
| Bra021824 | 2664 | Up | 3.57E-07 |
| Bra021645 | 1137 | Up | 3.74E-59 |
| Bra024717 | 1584 | Up | 4.32E-26 |
| Bra005019 | 1428 | Up | 0 |
| Bra040569 | 1302 | Up | 2.79E-17 |
| Bra031540 | 300 | Up | 2.32E-21 |
| Bra038589 | 1170 | Up | 1.21E-88 |
| Bra013043 | 1239 | Up | 1.31E-111 |
| Bra002747 | 933 | Up | 7.38E-07 |
| Bra003682 | 1065 | Up | 7.37E-07 |
| Bra024107 | 2652 | Up | 7.37E-07 |
| Bra032936 | 540 | Up | 7.47E-11 |
| Bra034610 | 1473 | Up | 8.60E-52 |
| Bra010285 | 1290 | Up | 0.00010186 |
| Bra013682 | 5703 | Up | 1.08E-08 |
| Bra003030 | 2415 | Up | 0.000101928 |
| Bra019413 | 2616 | Up | 1.08E-08 |
| Bra024009 | 528 | Up | 0.000101901 |
| Bra038467 | 1770 | Up | 0.000101887 |
| Bra009113 | 615 | Up | 0.000101874 |
| Bra027054 | 2748 | Up | 0.000101846 |
| Bra017016 | 3498 | Up | 0.000101833 |
| Bra038137 | 477 | Up | 0.000101915 |
| Bra010802 | 1002 | Up | 0.000101819 |
| Bra040633 | 1056 | Up | 1.47E-71 |
| Bra039546 | 1782 | Up | 1.54E-10 |
| Bra024675 | 525 | Up | 1.54E-10 |
| Bra030417 | 2556 | Up | 1.52E-06 |
| Bra009234 | 996 | Up | 1.52E-06 |
| Bra023849 | 591 | Up | 2.53E-12 |
| Bra038055 | 840 | Up | 3.99E-14 |
| Bra037242 | 474 | Up | 2.22E-08 |
| Bra000799 | 1971 | Up | 1.09E-25 |
| Bra038360 | 1107 | Up | 2.84E-37 |
| Bra040091 | 2376 | Up | 3.19E-10 |
| Bra021803 | 2229 | Up | 3.19E-10 |
| Bra020376 | 795 | Up | 1.43E-84 |
| Bra039053 | 1572 | Up | 4.84E-25 |
| Bra000796 | 1827 | Up | 1.13E-30 |
| Bra038569 | 2334 | Up | 3.10E-06 |
| Bra002531 | 1281 | Up | 4.55E-08 |
| Bra018538 | 1551 | Up | 2.39E-15 |
| Bra016060 | 531 | Up | 1.34E-26 |
| Bra018132 | 681 | Up | 4.55E-08 |
| Bra010436 | 354 | Up | 0.000206825 |
| Bra013652 | 1077 | Up | 9.91E-12 |
| Bra020156 | 1326 | Up | 0.000206746 |
| Bra016143 | 1455 | Up | 0.00020672 |
| Bra006231 | 1218 | Up | 0.000206851 |
| Bra027414 | 1731 | Up | 3.10E-06 |
| Bra017871 | 1626 | Up | 4.55E-08 |
| Bra006580 | 1029 | Up | 0.000206799 |
| Bra004388 | 2445 | Up | 0.000206772 |
| Bra005137 | 1506 | Up | 3.46E-58 |
| Bra040998 | 1887 | Up | 7.86E-28 |
| Bra039973 | 789 | Up | 2.01E-11 |
| Bra038671 | 636 | Up | 7.18E-42 |
| Bra023297 | 4524 | Up | 9.29E-08 |
| Bra025001 | 1092 | Up | 9.28E-08 |
| Bra023365 | 306 | Up | 7.04E-13 |
| Bra013227 | 1347 | Up | 7.04E-13 |
| Bra039223 | 1488 | Up | 6.28E-06 |
| Bra010995 | 1737 | Up | 6.29E-06 |
| Bra007828 | 1395 | Up | 4.09E-11 |
| Bra034855 | 432 | Up | 6.29E-06 |
| Bra010472 | 1077 | Up | 4.09E-11 |
| Bra015379 | 648 | Up | 9.97E-34 |
| Bra038089 | 771 | Up | 2.38E-24 |
| Bra032160 | 786 | Up | 6.37E-21 |
| Bra021485 | 1170 | Up | 0.000413132 |
| Bra018556 | 282 | Up | 0.000413181 |
| Bra006575 | 576 | Up | 0.000413083 |
| Bra032143 | 945 | Up | 1.89E-07 |
| Bra038492 | 2727 | Up | 0.000413034 |
| Bra014520 | 1347 | Up | 1.89E-07 |
| Bra037268 | 1182 | Up | 0.000412984 |
| Bra027272 | 1587 | Up | 0.000412935 |
| Bra016860 | 735 | Up | 8.92E-112 |
| Bra016789 | 1773 | Up | 7.53E-81 |
| Bra023978 | 699 | Up | 1.26E-39 |
| Bra023790 | 3054 | Up | 5.67E-09 |
| Bra031112 | 885 | Up | 3.58E-17 |
| Bra007588 | 879 | Up | 1.27E-05 |
| Bra038048 | 411 | Up | 1.27E-05 |
| Bra013783 | 1284 | Up | 1.68E-10 |
| Bra039755 | 801 | Up | 1.27E-05 |
| Bra036138 | 1062 | Up | 1.27E-05 |
| Bra001995 | 543 | Up | 2.63E-15 |
| Bra031889 | 300 | Up | 3.82E-07 |
| Bra015911 | 837 | Up | 1.88E-13 |
| Bra007931 | 909 | Up | 3.82E-07 |
| Bra007237 | 1017 | Up | 3.82E-07 |
| Bra029231 | 2148 | Up | 1.60E-21 |
| Bra015306 | 1041 | Up | 4.47E-23 |
| Bra019340 | 1041 | Up | 1.16E-08 |
| Bra032426 | 777 | Up | 1.16E-08 |
| Bra027006 | 888 | Up | 1.16E-08 |
| Bra004736 | 843 | Up | 1.06E-11 |
| Bra007163 | 579 | Up | 1.21E-52 |
| Bra009312 | 1536 | Up | 3.11E-63 |
| Bra016562 | 498 | Up | 2.34E-08 |
| Bra031900 | 594 | Up | 2.34E-08 |
| Bra000881 | 2028 | Up | 2.34E-08 |
| Bra020821 | 3555 | Up | 2.27E-14 |
| Bra016308 | 1518 | Up | 0.000826856 |
| Bra034639 | 429 | Up | 2.34E-08 |
| Bra031369 | 474 | Up | 7.77E-07 |
| Bra039000 | 1098 | Up | 2.34E-08 |
| Bra039511 | 549 | Up | 0.000826948 |
| Bra034506 | 1260 | Up | 2.56E-05 |
| Bra005936 | 792 | Up | 0.000826763 |
| Bra032788 | 1407 | Up | 6.98E-10 |
| Bra008366 | 2787 | Up | 6.53E-16 |
| Bra031833 | 726 | Up | 0.00082667 |
| Bra032141 | 675 | Up | 6.97E-10 |
| Bra007230 | 321 | Up | 1.01E-27 |
| Bra014524 | 1677 | Up | 1.38E-57 |
| Bra040531 | 3144 | Up | 7.21E-35 |
| Bra024735 | 1119 | Up | 8.49E-50 |
| Bra027906 | 1368 | Up | 3.34E-30 |
| Bra031043 | 606 | Up | 2.74E-15 |
| Bra024656 | 333 | Up | 2.86E-12 |
| Bra023676 | 1167 | Up | 1.49E-152 |
| Bra038610 | 924 | Up | 8.83E-27 |
| Bra030692 | 1293 | Up | 8.53E-11 |
| Bra010332 | 792 | Up | 0 |
| Bra027057 | 1659 | Up | 6.31E-34 |
| Bra015980 | 738 | Up | 5.08E-05 |
| Bra020628 | 1179 | Up | 2.85E-09 |
| Bra036174 | 2568 | Up | 5.08E-05 |
| Bra016844 | 2469 | Up | 5.08E-05 |
| Bra022535 | 2436 | Up | 2.09E-130 |
| Bra024472 | 1251 | Up | 9.47E-08 |
| Bra014637 | 960 | Up | 2.45E-37 |
| Bra015820 | 240 | Up | 1.71E-10 |
| Bra037113 | 2169 | Up | 6.24E-151 |
| Bra030917 | 1116 | Up | 4.84E-23 |
| Bra003778 | 669 | Up | 4.84E-23 |
| Bra025438 | 342 | Up | 4.38E-27 |
| Bra020984 | 1407 | Up | 3.12E-06 |
| Bra009055 | 621 | Up | 3.12E-06 |
| Bra015917 | 3255 | Up | 2.12E-36 |
| Bra039206 | 792 | Up | 1.36E-15 |
| Bra005644 | 390 | Up | 3.45E-10 |
| Bra011685 | 1080 | Up | 1.89E-07 |
| Bra012274 | 966 | Up | 1.25E-257 |
| Bra020915 | 1596 | Up | 2.14E-11 |
| Bra031387 | 1452 | Up | 2.76E-15 |
| Bra017368 | 972 | Up | 0.000100627 |
| Bra003501 | 1116 | Up | 1.15E-08 |
| Bra010475 | 477 | Up | 1.24E-28 |
| Bra038416 | 465 | Up | 0.000100641 |
| Bra012765 | 564 | Up | 0.000100614 |
| Bra017146 | 798 | Up | 1.15E-08 |
| Bra019321 | 864 | Up | 0.0001006 |
| Bra036596 | 2124 | Up | 4.06E-31 |
| Bra009108 | 303 | Up | 9.55E-14 |
| Bra022115 | 690 | Up | 5.28E-51 |
| Bra035561 | 1383 | Up | 6.94E-10 |
| Bra034666 | 1674 | Up | 5.25E-19 |
| Bra017529 | 1050 | Up | 6.94E-10 |
| Bra011590 | 1443 | Up | 1.32E-33 |
| Bra002248 | 456 | Up | 6.19E-06 |
| Bra004826 | 729 | Up | 6.19E-06 |
| Bra041122 | 1641 | Up | 2.85E-12 |
| Bra002937 | 1134 | Up | 2.85E-12 |
| Bra034237 | 1503 | Up | 0 |
| Bra031113 | 1365 | Up | 1.13E-14 |
| Bra007315 | 990 | Up | 2.65E-126 |
| Bra023471 | 894 | Up | 6.60E-16 |
| Bra025567 | 993 | Up | 2.30E-08 |
| Bra007991 | 1542 | Up | 1.97E-31 |
| Bra001341 | 1377 | Up | 8.37E-11 |
| Bra025547 | 1329 | Up | 4.15E-101 |
| Bra015415 | 984 | Up | 1.59E-62 |
| Bra031065 | 822 | Up | 1.80E-194 |
| Bra029392 | 1557 | Up | 3.10E-34 |
| Bra003816 | 2748 | Up | 0.000197801 |
| Bra026839 | 360 | Up | 7.49E-07 |
| Bra000352 | 729 | Up | 1.66E-10 |
| Bra001567 | 3048 | Up | 0.0001977 |
| Bra003335 | 906 | Up | 0.000197751 |
| Bra026536 | 567 | Up | 7.49E-07 |
| Bra011880 | 1617 | Up | 4.57E-08 |
| Bra037735 | 285 | Up | 2.76E-09 |
| Bra031636 | 1077 | Up | 1.66E-10 |
| Bra013409 | 930 | Up | 0.000197725 |
| Bra037857 | 756 | Up | 1.22E-05 |
| Bra014991 | 1113 | Up | 1.22E-05 |
| Bra004334 | 2313 | Up | 2.91E-20 |
| Bra040152 | 1074 | Up | 2.76E-09 |
| Bra040472 | 1128 | Up | 2.76E-09 |
| Bra002148 | 1011 | Up | 0.000197776 |
| Bra015515 | 1089 | Up | 0.000197675 |
| Bra030319 | 1071 | Up | 0 |
| Bra030360 | 1395 | Up | 4.18E-27 |
| Bra019587 | 1689 | Up | 9.21E-14 |
| Bra001610 | 1062 | Up | 9.01E-08 |
| Bra003636 | 1359 | Up | 6.01E-81 |
| Bra030515 | 633 | Up | 1.48E-06 |
| Bra001160 | 3135 | Up | 2.73E-12 |
| Bra034365 | 525 | Up | 2.40E-05 |
| Bra003373 | 1227 | Up | 2.40E-05 |
| Bra035271 | 1404 | Up | 2.15E-14 |
| Bra038763 | 1752 | Up | 2.40E-05 |
| Bra006543 | 771 | Up | 8.13E-55 |
| Bra033038 | 2790 | Up | 1.09E-08 |
| Bra026467 | 939 | Up | 5.13E-12 |
| Bra038962 | 348 | Up | 9.52E-80 |
| Bra020753 | 2382 | Up | 1.78E-07 |
| Bra002355 | 1722 | Up | 6.93E-13 |
| Bra024269 | 987 | Up | 1.02E-37 |
| Bra007581 | 564 | Up | 3.42E-17 |
| Bra012624 | 1872 | Up | 1.19E-48 |
| Bra034547 | 2616 | Up | 4.68E-05 |
| Bra016213 | 1404 | Up | 4.68E-05 |
| Bra017126 | 501 | Up | 4.67E-05 |
| Bra038966 | 1353 | Up | 2.56E-09 |
| Bra001517 | 1275 | Up | 3.48E-07 |
| Bra033464 | 612 | Up | 4.68E-05 |
| Bra032454 | 450 | Up | 4.68E-05 |
| Bra027397 | 1743 | Up | 4.67E-05 |
| Bra023742 | 846 | Up | 1.91E-11 |
| Bra033278 | 1608 | Up | 2.34E-15 |
| Bra034561 | 939 | Up | 7.12E-56 |
| Bra012151 | 1428 | Up | 3.96E-28 |
| Bra013292 | 1173 | Up | 2.72E-16 |
| Bra030295 | 852 | Up | 5.65E-06 |
| Bra037828 | 945 | Up | 5.65E-06 |
| Bra002806 | 1071 | Up | 2.37E-20 |
| Bra019301 | 747 | Up | 0.000744103 |
| Bra015382 | 1635 | Up | 0.000744019 |
| Bra017647 | 1578 | Up | 0.000743681 |
| Bra020583 | 999 | Up | 0.000743765 |
| Bra014235 | 1083 | Up | 0.000743934 |
| Bra000935 | 1731 | Up | 0.00074385 |
| Bra019406 | 1407 | Up | 3.03E-81 |
| Bra006610 | 924 | Up | 5.44E-21 |
| Bra030589 | 882 | Up | 5.44E-21 |
| Bra001402 | 1503 | Up | 8.97E-12 |
| Bra039787 | 501 | Up | 1.07E-15 |
| Bra024380 | 1422 | Up | 9.84E-09 |
| Bra037487 | 2436 | Up | 9.84E-09 |
| Bra031297 | 1500 | Up | 9.04E-05 |
| Bra002886 | 306 | Up | 9.84E-09 |
| Bra029535 | 1137 | Up | 9.04E-05 |
| Bra037782 | 2331 | Up | 2.87E-153 |
| Bra041014 | 930 | Up | 1.17E-09 |
| Bra036701 | 954 | Up | 1.17E-09 |
| Bra029235 | 1107 | Up | 1.66E-43 |
| Bra015935 | 561 | Up | 1.10E-05 |
| Bra040471 | 1227 | Up | 1.10E-05 |
| Bra001694 | 1062 | Up | 1.10E-05 |
| Bra010318 | 1656 | Up | 1.10E-05 |
| Bra008519 | 1668 | Up | 1.10E-05 |
| Bra017552 | 1917 | Up | 1.10E-05 |
| Bra038438 | 912 | Up | 1.32E-06 |
| Bra030601 | 2325 | Up | 1.32E-06 |
| Bra030821 | 969 | Up | 1.97E-40 |
| Bra033494 | 1434 | Up | 2.72E-10 |
| Bra002235 | 3345 | Up | 1.45E-18 |
| Bra030106 | 1014 | Up | 5.22E-48 |
| Bra002849 | 2919 | Up | 4.36E-26 |
| Bra035470 | 1791 | Up | 6.21E-114 |
| Bra035306 | 1314 | Up | 2.55E-06 |
| Bra001277 | 1155 | Up | 0.00017352 |
| Bra020494 | 1422 | Up | 3.70E-08 |
| Bra027453 | 834 | Up | 0.000173475 |
| Bra005309 | 1164 | Up | 0.000173655 |
| Bra003222 | 642 | Up | 0.00017361 |
| Bra040944 | 918 | Up | 0.000173565 |
| Bra014547 | 873 | Up | 0.000173543 |
| Bra030434 | 1362 | Up | 2.55E-06 |
| Bra035885 | 1695 | Up | 0.000173633 |
| Bra018521 | 1287 | Up | 0.000173498 |
| Bra023688 | 309 | Up | 2.54E-06 |
| Bra020119 | 432 | Up | 7.95E-12 |
| Bra039484 | 597 | Up | 2.11E-05 |
| Bra022650 | 330 | Up | 2.11E-05 |
| Bra035996 | 648 | Up | 2.54E-06 |
| Bra031765 | 2511 | Up | 0.000173588 |
| Bra030575 | 1725 | Up | 2.64E-13 |
| Bra023481 | 1104 | Up | 3.96E-37 |
| Bra033546 | 618 | Up | 1.51E-11 |
| Bra036575 | 1599 | Up | 1.46E-19 |
| Bra000793 | 1560 | Up | 1.11E-17 |
| Bra031423 | 1170 | Up | 1.04E-55 |
| Bra009783 | 282 | Up | 7.15E-08 |
| Bra023154 | 1044 | Up | 7.15E-08 |
| Bra013579 | 1407 | Up | 2.04E-55 |
| Bra038766 | 2295 | Up | 6.08E-14 |
| Bra000860 | 348 | Up | 5.88E-07 |
| Bra009460 | 1179 | Up | 5.89E-07 |
| Bra003562 | 1185 | Up | 5.89E-07 |
| Bra040666 | 1740 | Up | 4.88E-06 |
| Bra014771 | 1899 | Up | 4.03E-05 |
| Bra003444 | 1497 | Up | 1.18E-13 |
| Bra031335 | 1509 | Up | 4.03E-05 |
| Bra003164 | 771 | Up | 4.03E-05 |
| Bra033682 | 3060 | Up | 4.03E-05 |
| Bra004330 | 1080 | Up | 3.68E-16 |
| Bra003234 | 384 | Up | 4.02E-51 |
| Bra015165 | 3051 | Up | 1.04E-52 |
| Bra026992 | 420 | Up | 3.76E-22 |
| Bra025781 | 498 | Up | 0.000327315 |
| Bra027648 | 1857 | Up | 0.000327395 |
| Bra010839 | 786 | Up | 0.000327355 |
| Bra036475 | 195 | Up | 0.000327475 |
| Bra017012 | 618 | Up | 0.000327435 |
| Bra021583 | 2271 | Up | 4.54E-10 |
| Bra017523 | 1524 | Up | 4.54E-10 |
| Bra037739 | 1551 | Up | 7.15E-16 |
| Bra036883 | 576 | Up | 3.61E-107 |
| Bra017111 | 591 | Up | 3.80E-09 |
| Bra001122 | 423 | Up | 2.93E-39 |
| Bra027295 | 1812 | Up | 2.93E-39 |
| Bra001992 | 3102 | Up | 3.16E-08 |
| Bra019389 | 3294 | Up | 9.34E-06 |
| Bra001766 | 1545 | Up | 3.16E-08 |
| Bra020052 | 1866 | Up | 3.16E-08 |
| Bra003947 | 663 | Up | 9.34E-06 |
| Bra030916 | 1092 | Up | 8.74E-10 |
| Bra005023 | 720 | Up | 3.13E-16 |
| Bra035067 | 1365 | Up | 1.80E-133 |
| Bra017846 | 1164 | Up | 7.29E-09 |
| Bra004370 | 885 | Up | 7.62E-05 |
| Bra013750 | 2160 | Up | 7.29E-09 |
| Bra018781 | 645 | Up | 7.62E-05 |
| Bra029472 | 663 | Up | 7.62E-05 |
| Bra008142 | 795 | Up | 2.12E-32 |
| Bra027476 | 882 | Up | 6.20E-29 |
| Bra026570 | 690 | Up | 1.93E-13 |
| Bra017051 | 2154 | Up | 1.93E-13 |
| Bra010816 | 2322 | Up | 5.14E-15 |
| Bra027501 | 738 | Up | 6.05E-08 |
| Bra029480 | 582 | Up | 3.53E-18 |
| Bra010581 | 939 | Up | 6.09E-23 |
| Bra005208 | 4248 | Up | 0.000617807 |
| Bra036784 | 339 | Up | 1.39E-08 |
| Bra002459 | 483 | Up | 7.82E-26 |
| Bra037576 | 654 | Up | 0.000617878 |
| Bra024892 | 807 | Up | 0.000617949 |
| Bra016433 | 1119 | Up | 3.82E-10 |
| Bra001891 | 1389 | Up | 6.65E-221 |
| Bra006925 | 1308 | Up | 3.92E-20 |
| Bra010967 | 1269 | Up | 8.45E-14 |
| Bra017958 | 1899 | Up | 8.46E-14 |
| Bra009018 | 525 | Up | 8.45E-14 |
| Bra030494 | 456 | Up | 1.74E-40 |
| Bra007904 | 1128 | Up | 5.10E-23 |
| Bra008440 | 597 | Up | 1.14E-07 |
| Bra019776 | 966 | Up | 1.14E-07 |
| Bra012938 | 777 | Up | 1.50E-69 |
| Bra001907 | 861 | Up | 1.13E-30 |
| Bra007103 | 1185 | Up | 2.54E-24 |
| Bra023644 | 321 | Up | 1.61E-13 |
| Bra031042 | 651 | Up | 0.000143291 |
| Bra028628 | 2043 | Up | 2.42E-39 |
| Bra024101 | 2718 | Up | 3.66E-14 |
| Bra039844 | 1524 | Up | 1.77E-285 |
| Bra031012 | 1116 | Up | 1.67E-10 |
| Bra016400 | 633 | Up | 3.26E-20 |
| Bra025993 | 663 | Up | 1.67E-10 |
| Bra040756 | 1104 | Up | 1.67E-10 |
| Bra017180 | 966 | Up | 2.33E-40 |
| Bra007259 | 1482 | Up | 3.90E-11 |
| Bra017035 | 345 | Up | 1.87E-15 |
| Bra028658 | 903 | Up | 3.65E-22 |
| Bra001912 | 2223 | Up | 3.32E-05 |
| Bra036307 | 1182 | Up | 2.44E-18 |
| Bra014640 | 960 | Up | 2.15E-07 |
| Bra002892 | 414 | Up | 3.12E-21 |
| Bra024030 | 1326 | Up | 1.57E-14 |
| Bra021441 | 1473 | Up | 1.05E-144 |
| Bra003058 | 651 | Up | 2.10E-17 |
| Bra036724 | 1575 | Up | 3.76E-57 |
| Bra008575 | 573 | Up | 7.27E-11 |
| Bra005860 | 1119 | Up | 7.64E-06 |
| Bra005813 | 204 | Up | 7.64E-06 |
| Bra004835 | 1521 | Up | 1.75E-147 |
| Bra009716 | 1389 | Up | 1.35E-21 |
| Bra020549 | 789 | Up | 9.11E-43 |
| Bra004066 | 756 | Up | 1.29E-162 |
| Bra022219 | 1341 | Up | 1.80E-16 |
| Bra037651 | 1524 | Up | 4.55E-136 |
| Bra018682 | 312 | Up | 1.76E-06 |
| Bra026630 | 1002 | Up | 3.17E-32 |
| Bra017495 | 738 | Up | 8.96E-18 |
| Bra001474 | 1518 | Up | 9.45E-28 |
| Bra018007 | 1062 | Up | 2.51E-13 |
| Bra000800 | 1950 | Up | 3.66E-26 |
| Bra030284 | 507 | Up | 7.06E-233 |
| Bra031484 | 1479 | Up | 3.82E-18 |
| Bra008408 | 1560 | Up | 0.000266432 |
| Bra025561 | 1707 | Up | 7.57E-12 |
| Bra038445 | 1365 | Up | 2.15E-08 |
| Bra029601 | 429 | Up | 1.21E-23 |
| Bra004836 | 1518 | Up | 9.37E-21 |
| Bra021220 | 465 | Up | 3.85E-29 |
| Bra015856 | 267 | Up | 6.17E-05 |
| Bra035897 | 600 | Up | 4.22E-115 |
| Bra035175 | 1455 | Up | 1.09E-13 |
| Bra024572 | 393 | Up | 1.43E-05 |
| Bra022785 | 2415 | Up | 1.43E-05 |
| Bra031478 | 2859 | Up | 1.43E-05 |
| Bra026377 | 912 | Up | 1.43E-05 |
| Bra010500 | 2361 | Up | 7.66E-85 |
| Bra001701 | 291 | Up | 5.94E-11 |
| Bra021018 | 1197 | Up | 3.30E-06 |
| Bra034608 | 1452 | Up | 1.39E-11 |
| Bra013764 | 2004 | Up | 2.21E-46 |
| Bra017872 | 885 | Up | 3.43E-12 |
| Bra015964 | 1374 | Up | 3.43E-12 |
| Bra003220 | 2022 | Up | 1.55E-30 |
| Bra018223 | 759 | Up | 1.75E-07 |
| Bra004768 | 267 | Up | 1.75E-07 |
| Bra005917 | 1029 | Up | 4.17E-24 |
| Bra034439 | 1737 | Up | 2.11E-09 |
| Bra006971 | 1509 | Up | 5.88E-18 |
| Bra006713 | 1101 | Up | 8.32E-38 |
| Bra026789 | 297 | Up | 4.41E-15 |
| Bra027321 | 1539 | Up | 2.24E-16 |
| Bra005641 | 1308 | Up | 1.70E-43 |
| Bra026163 | 1191 | Up | 0.000491636 |
| Bra011147 | 795 | Up | 1.41E-06 |
| Bra000382 | 1533 | Up | 3.24E-07 |
| Bra038769 | 402 | Up | 3.24E-07 |
| Bra000046 | 885 | Up | 0.000113958 |
| Bra030166 | 2178 | Up | 2.80E-12 |
| Bra015818 | 771 | Up | 3.70E-14 |
| Bra009036 | 1059 | Up | 1.89E-15 |
| Bra024491 | 612 | Up | 2.65E-05 |
| Bra006942 | 921 | Up | 2.23E-80 |
| Bra028754 | 978 | Up | 1.13E-84 |
| Bra028604 | 1089 | Up | 4.98E-12 |
| Bra002992 | 1125 | Up | 3.84E-10 |
| Bra018740 | 1422 | Up | 3.27E-35 |
| Bra010425 | 306 | Up | 7.35E-09 |
| Bra021802 | 2283 | Up | 2.94E-14 |
| Bra000880 | 2040 | Up | 3.49E-19 |
| Bra029045 | 1278 | Up | 6.00E-07 |
| Bra006495 | 2880 | Up | 2.62E-63 |
| Bra029515 | 1443 | Up | 2.61E-06 |
| Bra031470 | 1218 | Up | 1.05E-32 |
| Bra027302 | 2817 | Up | 1.79E-55 |
| Bra031435 | 543 | Up | 1.13E-05 |
| Bra036332 | 369 | Up | 1.13E-05 |
| Bra016193 | 750 | Up | 3.66E-88 |
| Bra039194 | 2325 | Up | 1.68E-24 |
| Bra011304 | 2277 | Up | 5.78E-43 |
| Bra010658 | 1038 | Up | 3.11E-09 |
| Bra037743 | 834 | Up | 4.88E-05 |
| Bra019536 | 1818 | Up | 3.11E-09 |
| Bra010821 | 1095 | Up | 2.62E-52 |
| Bra002626 | 1743 | Up | 5.93E-08 |
| Bra020621 | 1140 | Up | 5.36E-22 |
| Bra031229 | 2382 | Up | 0.000900832 |
| Bra018745 | 897 | Up | 0.000900932 |
| Bra007863 | 1017 | Up | 1.11E-06 |
| Bra019538 | 1455 | Up | 0.000901032 |
| Bra014247 | 909 | Up | 1.32E-09 |
| Bra029842 | 1248 | Up | 4.33E-25 |
| Bra013596 | 1938 | Up | 1.08E-07 |
| Bra034981 | 438 | Up | 5.59E-10 |
| Bra014490 | 795 | Up | 2.07E-05 |
| Bra018325 | 1122 | Up | 2.07E-05 |
| Bra004083 | 1866 | Up | 1.08E-07 |
| Bra024917 | 1119 | Up | 4.69E-07 |
| Bra040817 | 372 | Up | 2.53E-63 |
| Bra011164 | 2619 | Up | 5.48E-11 |
| Bra017206 | 987 | Up | 1.06E-08 |
| Bra009370 | 837 | Up | 2.47E-50 |
| Bra004193 | 2070 | Up | 2.03E-06 |
| Bra037183 | 3456 | Up | 2.03E-06 |
| Bra018251 | 924 | Up | 2.35E-11 |
| Bra028595 | 2034 | Up | 8.73E-43 |
| Bra035288 | 1110 | Up | 8.78E-06 |
| Bra007969 | 2100 | Up | 0.000380372 |
| Bra037159 | 1551 | Up | 8.78E-06 |
| Bra026157 | 4389 | Up | 2.64E-82 |
| Bra023940 | 639 | Up | 8.40E-33 |
| Bra027307 | 3036 | Up | 3.03E-42 |
| Bra006343 | 1515 | Up | 1.01E-11 |
| Bra028959 | 717 | Up | 1.94E-08 |
| Bra000610 | 618 | Up | 8.59E-07 |
| Bra020862 | 1509 | Up | 8.59E-07 |
| Bra000498 | 1608 | Up | 0 |
| Bra031599 | 624 | Up | 3.77E-05 |
| Bra034731 | 321 | Up | 0 |
| Bra019678 | 912 | Up | 2.17E-17 |
| Bra036394 | 351 | Up | 4.94E-13 |
| Bra018285 | 1062 | Up | 1.03E-15 |
| Bra005070 | 1605 | Up | 3.62E-22 |
| Bra036413 | 3147 | Up | 3.62E-07 |
| Bra035181 | 1569 | Up | 3.54E-08 |
| Bra014842 | 552 | Up | 0.000161653 |
| Bra003014 | 1311 | Up | 0.000161589 |
| Bra037088 | 4188 | Up | 0.000161611 |
| Bra001202 | 915 | Up | 0.000161568 |
| Bra032165 | 1530 | Up | 0.000161632 |
| Bra037785 | 531 | Up | 3.54E-08 |
| Bra032343 | 1008 | Up | 1.31E-150 |
| Bra026188 | 1857 | Up | 1.38E-35 |
| Bra023588 | 3423 | Up | 3.48E-12 |
| Bra002914 | 309 | Up | 8.74E-217 |
| Bra027692 | 747 | Up | 3.68E-14 |
| Bra032581 | 996 | Up | 1.53E-07 |
| Bra031929 | 1134 | Up | 3.50E-15 |
| Bra028472 | 1995 | Up | 1.44E-09 |
| Bra040428 | 1164 | Up | 1.41E-11 |
| Bra014436 | 1107 | Up | 2.96E-181 |
| Bra030385 | 2112 | Up | 3.60E-35 |
| Bra007568 | 942 | Up | 6.80E-05 |
| Bra002334 | 1029 | Up | 0.000685403 |
| Bra005739 | 453 | Up | 0.000685325 |
| Bra033351 | 378 | Up | 0.000685168 |
| Bra028874 | 2613 | Up | 0.000685247 |
| Bra034589 | 732 | Up | 6.27E-09 |
| Bra016792 | 159 | Up | 1.74E-21 |
| Bra010970 | 261 | Up | 2.17E-18 |
| Bra039968 | 1200 | Up | 6.25E-52 |
| Bra009702 | 1614 | Up | 2.54E-10 |
| Bra040159 | 723 | Up | 9.96E-34 |
| Bra028853 | 408 | Up | 2.81E-06 |
| Bra018039 | 870 | Up | 6.10E-119 |
| Bra015347 | 1194 | Up | 5.25E-13 |
| Bra010484 | 2049 | Up | 5.25E-13 |
| Bra029191 | 2916 | Up | 1.14E-08 |
| Bra003630 | 2304 | Up | 2.51E-61 |
| Bra023861 | 624 | Up | 0.000288706 |
| Bra024379 | 1707 | Up | 2.24E-13 |
| Bra000985 | 426 | Up | 3.90E-31 |
| Bra002773 | 1524 | Up | 1.18E-06 |
| Bra005198 | 1080 | Up | 2.12E-12 |
| Bra031132 | 1272 | Up | 3.22E-62 |
| Bra032339 | 390 | Up | 8.82E-15 |
| Bra006827 | 873 | Up | 1.21E-05 |
| Bra015979 | 480 | Up | 1.21E-05 |
| Bra019830 | 3393 | Up | 4.53E-46 |
| Bra015656 | 2673 | Up | 0 |
| Bra017125 | 615 | Up | 1.60E-14 |
| Bra000875 | 642 | Up | 4.57E-90 |
| Bra000096 | 1485 | Up | 0.000121309 |
| Bra029956 | 2355 | Up | 0.000121325 |
| Bra039268 | 405 | Up | 5.05E-06 |
| Bra029768 | 1116 | Up | 5.05E-06 |
| Bra035670 | 753 | Up | 3.45E-11 |
| Bra015193 | 1446 | Up | 5.05E-06 |
| Bra006063 | 558 | Up | 5.05E-06 |
| Bra006629 | 822 | Up | 3.96E-44 |
| Bra006224 | 1191 | Up | 7.91E-63 |
| Bra005009 | 873 | Up | 1.61E-12 |
| Bra040902 | 1719 | Up | 7.06E-14 |
| Bra036881 | 1167 | Up | 8.59E-09 |
| Bra037630 | 708 | Up | 1.46E-11 |
| Bra019526 | 795 | Up | 1.46E-11 |
| Bra016428 | 2211 | Up | 7.00E-13 |
| Bra036315 | 1473 | Up | 5.71E-39 |
| Bra027457 | 1158 | Up | 2.12E-06 |
| Bra015404 | 981 | Up | 5.11E-05 |
| Bra023011 | 663 | Up | 3.58E-09 |
| Bra031912 | 2337 | Up | 4.08E-44 |
| Bra007213 | 1713 | Up | 5.99E-30 |
| Bra040999 | 759 | Up | 2.15E-05 |
| Bra039563 | 876 | Up | 1.37E-17 |
| Bra031585 | 1221 | Up | 3.72E-07 |
| Bra009487 | 198 | Up | 0.000512836 |
| Bra023099 | 993 | Up | 1.39E-88 |
| Bra003712 | 540 | Up | 2.11E-28 |
| Bra004138 | 489 | Up | 1.23E-36 |
| Bra007071 | 822 | Up | 6.43E-09 |
| Bra010143 | 1059 | Up | 9.04E-06 |
| Bra012605 | 402 | Up | 9.04E-06 |
| Bra027908 | 1758 | Up | 3.11E-126 |
| Bra027568 | 927 | Up | 1.56E-07 |
| Bra031409 | 474 | Up | 4.61E-11 |
| Bra021444 | 318 | Up | 1.63E-14 |
| Bra004770 | 306 | Up | 4.22E-18 |
| Bra005824 | 1821 | Up | 2.62E-25 |
| Bra000883 | 1902 | Up | 6.58E-08 |
| Bra038499 | 792 | Up | 6.58E-08 |
| Bra021090 | 1485 | Up | 2.93E-45 |
| Bra015227 | 1770 | Up | 5.62E-110 |
| Bra009969 | 1824 | Up | 2.94E-14 |
| Bra017015 | 759 | Up | 9.09E-05 |
| Bra000797 | 1827 | Up | 2.24E-36 |
| Bra038584 | 864 | Up | 2.66E-194 |
| Bra022468 | 1452 | Up | 1.32E-35 |
| Bra023403 | 1713 | Up | 1.60E-05 |
| Bra021508 | 1740 | Up | 1.60E-05 |
| Bra012504 | 1257 | Up | 3.47E-10 |
| Bra011869 | 489 | Up | 1.07E-149 |
| Bra035782 | 2691 | Up | 3.55E-108 |
| Bra011409 | 1419 | Up | 2.04E-08 |
| Bra015952 | 1404 | Up | 8.98E-50 |
| Bra002712 | 1215 | Up | 2.64E-16 |
| Bra000165 | 345 | Up | 2.00E-20 |
| Bra025874 | 1119 | Up | 4.78E-12 |
| Bra003221 | 951 | Up | 1.30E-297 |
| Bra028118 | 390 | Up | 0.000904061 |
| Bra003654 | 2796 | Up | 0.000904162 |
| Bra019591 | 1065 | Up | 0.00090396 |
| Bra030661 | 1134 | Up | 9.79E-175 |
| Bra003289 | 1623 | Up | 6.18E-10 |
| Bra040056 | 1224 | Up | 4.91E-07 |
| Bra038081 | 804 | Up | 2.24E-77 |
| Bra035121 | 1512 | Up | 1.69E-13 |
| Bra009149 | 1467 | Up | 5.64E-105 |
| Bra026124 | 4242 | Up | 8.03E-17 |
| Bra001816 | 1716 | Up | 9.15E-74 |
| Bra022684 | 618 | Up | 0.000379205 |
| Bra037699 | 783 | Up | 2.06E-07 |
| Bra025640 | 1467 | Up | 2.49E-21 |
| Bra001008 | 2454 | Up | 6.44E-33 |
| Bra016194 | 933 | Up | 1.93E-11 |
| Bra021901 | 1050 | Up | 0.000160092 |
| Bra005311 | 702 | Up | 0.000160071 |
| Bra029505 | 1374 | Up | 1.31E-26 |
| Bra014080 | 945 | Up | 1.51E-08 |
| Bra017041 | 1413 | Up | 6.90E-13 |
| Bra030868 | 177 | Up | 2.98E-13 |
| Bra024099 | 1686 | Up | 2.62E-09 |
| Bra012780 | 1746 | Up | 2.62E-09 |
| Bra023095 | 603 | Up | 2.81E-05 |
| Bra036774 | 1773 | Up | 4.67E-50 |
| Bra023669 | 1011 | Up | 2.81E-05 |
| Bra019560 | 858 | Up | 1.47E-94 |
| Bra008066 | 879 | Up | 1.44E-82 |
| Bra012116 | 2253 | Up | 1.18E-05 |
| Bra026503 | 792 | Up | 4.07E-35 |
| Bra027902 | 1389 | Up | 4.93E-06 |
| Bra027963 | 1011 | Up | 4.34E-17 |
| Bra037506 | 969 | Up | 2.07E-06 |
| Bra005092 | 588 | Up | 2.07E-06 |
| Bra022753 | 894 | Up | 2.65E-24 |
| Bra014929 | 858 | Up | 2.07E-36 |
| Bra028878 | 786 | Up | 8.65E-07 |
| Bra035919 | 612 | Up | 8.65E-07 |
| Bra021736 | 924 | Up | 2.15E-80 |
| Bra009294 | 1323 | Up | 5.88E-38 |
| Bra025911 | 2607 | Up | 3.62E-07 |
| Bra031956 | 828 | Up | 3.62E-07 |
| Bra022592 | 717 | Up | 3.57E-28 |
| Bra004913 | 279 | Up | 6.38E-08 |
| Bra024145 | 432 | Up | 2.65E-08 |
| Bra035707 | 2055 | Up | 2.00E-32 |
| Bra001886 | 588 | Up | 2.81E-68 |
| Bra019853 | 810 | Up | 4.55E-97 |
| Bra008627 | 1104 | Up | 4.28E-42 |
| Bra017493 | 885 | Up | 1.77E-21 |
| Bra008915 | 684 | Up | 1.06E-11 |
| Bra029319 | 672 | Up | 1.96E-15 |
| Bra028596 | 1533 | Up | 1.96E-15 |
| Bra037358 | 867 | Up | 1.38E-16 |
| Bra001788 | 540 | Up | 3.53E-36 |
| Bra031986 | 2637 | Up | 0.000277731 |
| Bra015024 | 681 | Up | 0.000277697 |
| Bra018377 | 792 | Up | 4.92E-05 |
| Bra009602 | 777 | Up | 0.000116531 |
| Bra014105 | 525 | Up | 1.52E-12 |
| Bra007236 | 1761 | Up | 0.000116516 |
| Bra004130 | 2055 | Up | 1.00E-16 |
| Bra022682 | 681 | Up | 1.11E-07 |
| Bra015811 | 243 | Up | 0.000277628 |
| Bra013766 | 1491 | Up | 1.11E-07 |
| Bra025803 | 2283 | Up | 0.000277662 |
| Bra002843 | 810 | Up | 0.000277594 |
| Bra014636 | 930 | Up | 1.48E-124 |
| Bra037955 | 1185 | Up | 6.88E-22 |
| Bra019747 | 1395 | Up | 6.34E-31 |
| Bra028836 | 1146 | Up | 5.53E-82 |
| Bra034038 | 2379 | Up | 1.43E-08 |
| Bra021682 | 1281 | Up | 1.26E-44 |
| Bra038785 | 1788 | Up | 8.14E-08 |
| Bra027916 | 423 | Up | 8.14E-08 |
| Bra014375 | 1119 | Up | 5.57E-36 |
| Bra027004 | 2094 | Up | 1.53E-13 |
| Bra038501 | 1338 | Up | 1.10E-06 |
| Bra009798 | 1641 | Up | 2.64E-06 |
| Bra025994 | 660 | Up | 6.30E-06 |
| Bra018410 | 507 | Up | 6.30E-06 |
| Bra018591 | 1500 | Up | 2.56E-30 |
| Bra003670 | 2733 | Up | 2.56E-30 |
| Bra019503 | 558 | Up | 1.50E-05 |
| Bra003095 | 1110 | Up | 3.71E-29 |
| Bra020452 | 585 | Up | 1.50E-05 |
| Bra039737 | 747 | Up | 7.52E-10 |
| Bra004129 | 2097 | Up | 3.58E-05 |
| Bra012272 | 2268 | Up | 3.58E-05 |
| Bra019818 | 3627 | Up | 3.58E-05 |
| Bra002154 | 4029 | Up | 1.15E-17 |
| Bra020145 | 1713 | Up | 8.53E-05 |
| Bra010435 | 1779 | Up | 8.53E-05 |
| Bra009250 | 636 | Up | 2.36E-19 |
| Bra003039 | 642 | Up | 5.70E-23 |
| Bra013445 | 1896 | Up | 8.05E-14 |
| Bra024169 | 423 | Up | 0.00047934 |
| Bra016724 | 519 | Up | 0.000479397 |
| Bra023619 | 450 | Up | 0.000479284 |
| Bra005071 | 1524 | Up | 2.02E-28 |
| Bra005720 | 243 | Up | 7.25E-74 |
| Bra006721 | 348 | Up | 6.91E-46 |
| Bra026340 | 711 | Up | 1.92E-06 |
| Bra031181 | 1203 | Up | 1.27E-177 |
| Bra037162 | 552 | Up | 7.53E-09 |
| Bra022172 | 507 | Up | 6.95E-11 |
| Bra000315 | 837 | Up | 1.55E-62 |
| Bra001488 | 4482 | Up | 2.65E-31 |
| Bra004232 | 1035 | Up | 1.02E-07 |
| Bra023614 | 552 | Up | 2.60E-05 |
| Bra024659 | 684 | Up | 4.76E-45 |
| Bra029146 | 1257 | Up | 7.73E-25 |
| Bra032939 | 1005 | Up | 6.25E-120 |
| Bra040385 | 2253 | Up | 2.44E-07 |
| Bra003575 | 1638 | Up | 2.27E-09 |
| Bra009653 | 1620 | Up | 5.43E-09 |
| Bra031598 | 762 | Up | 1.09E-87 |
| Bra015882 | 564 | Up | 2.85E-29 |
| Bra029107 | 396 | Up | 1.25E-42 |
| Bra019765 | 1326 | Up | 4.01E-25 |
| Bra032930 | 315 | Up | 0.000146494 |
| Bra025553 | 681 | Up | 0.000146475 |
| Bra016585 | 996 | Up | 3.31E-06 |
| Bra023117 | 2121 | Up | 5.76E-24 |
| Bra019669 | 1419 | Up | 7.43E-08 |
| Bra000876 | 642 | Up | 1.25E-31 |
| Bra028996 | 1134 | Up | 5.83E-128 |
| Bra016733 | 2244 | Up | 0.000346447 |
| Bra020397 | 1056 | Up | 0.000346489 |
| Bra019925 | 546 | Up | 3.28E-40 |
| Bra022534 | 546 | Up | 1.83E-33 |
| Bra016381 | 1554 | Up | 2.88E-21 |
| Bra000487 | 1254 | Up | 4.19E-07 |
| Bra018933 | 2622 | Up | 1.28E-30 |
| Bra038579 | 2463 | Up | 4.93E-10 |
| Bra037671 | 1023 | Up | 1.66E-17 |
| Bra032018 | 1515 | Up | 2.24E-08 |
| Bra003136 | 621 | Up | 2.92E-119 |
| Bra025077 | 1299 | Up | 9.98E-07 |
| Bra019548 | 1428 | Up | 0.000823292 |
| Bra001718 | 1596 | Up | 0.000823385 |
| Bra027020 | 615 | Up | 0.000823478 |
| Bra034912 | 726 | Up | 6.28E-11 |
| Bra008032 | 354 | Up | 5.36E-08 |
| Bra033474 | 2313 | Up | 3.64E-12 |
| Bra039397 | 189 | Up | 4.45E-05 |
| Bra026393 | 1746 | Up | 1.13E-14 |
| Bra014984 | 1458 | Up | 1.49E-10 |
| Bra005229 | 1440 | Up | 3.78E-27 |
| Bra027210 | 1371 | Up | 2.38E-06 |
| Bra012691 | 933 | Up | 2.38E-06 |
| Bra037277 | 1656 | Up | 8.28E-12 |
| Bra029694 | 1611 | Up | 2.38E-06 |
| Bra028461 | 495 | Up | 3.55E-10 |
| Bra005190 | 1401 | Up | 1.62E-08 |
| Bra036259 | 642 | Up | 0.000105153 |
| Bra039936 | 1143 | Up | 3.54E-52 |
| Bra018150 | 1584 | Up | 4.32E-34 |
| Bra031495 | 984 | Up | 3.85E-08 |
| Bra011554 | 1947 | Up | 1.40E-11 |
| Bra019742 | 414 | Up | 4.86E-242 |
| Bra037386 | 1446 | Up | 1.80E-18 |
| Bra032150 | 1074 | Up | 2.59E-92 |
| Bra002036 | 1383 | Up | 0.000248868 |
| Bra001903 | 2217 | Up | 3.29E-11 |
| Bra033328 | 1476 | Up | 0.000248806 |
| Bra019626 | 837 | Up | 0.000248837 |
| Bra021015 | 414 | Up | 6.11E-10 |
| Bra037675 | 681 | Up | 2.39E-39 |
| Bra000361 | 600 | Up | 3.19E-05 |
| Bra033901 | 1380 | Up | 1.91E-137 |
| Bra002577 | 666 | Up | 1.51E-16 |
| Bra004115 | 918 | Up | 4.07E-06 |
| Bra014697 | 462 | Up | 3.48E-09 |
| Bra007240 | 672 | Up | 3.26E-12 |
| Bra010383 | 831 | Up | 0.000588674 |
| Bra031772 | 1032 | Up | 7.57E-05 |
| Bra015925 | 1083 | Up | 1.04E-09 |
| Bra007712 | 1254 | Up | 1.26E-37 |
| Bra036936 | 1365 | Up | 1.56E-07 |
| Bra038611 | 1149 | Up | 7.57E-64 |
| Bra034795 | 5793 | Up | 1.01E-44 |
| Bra023427 | 3276 | Up | 0 |
| Bra027915 | 1116 | Up | 1.37E-30 |
| Bra021274 | 1002 | Up | 7.17E-23 |
| Bra012371 | 1371 | Up | 7.73E-79 |
| Bra003981 | 546 | Up | 2.91E-06 |
| Bra009352 | 1533 | Up | 2.66E-26 |
| Bra036333 | 1017 | Up | 1.11E-07 |
| Bra037115 | 2301 | Up | 2.50E-161 |
| Bra028657 | 1176 | Up | 8.91E-12 |
| Bra014635 | 1041 | Up | 5.57E-87 |
| Bra036517 | 1494 | Up | 9.18E-13 |
| Bra011671 | 1482 | Up | 2.74E-45 |
| Bra039545 | 1452 | Up | 2.89E-13 |
| Bra030189 | 822 | Up | 7.98E-08 |
| Bra027531 | 750 | Up | 3.81E-24 |
| Bra034611 | 726 | Up | 1.12E-24 |
| Bra021265 | 2232 | Up | 3.02E-09 |
| Bra003273 | 1020 | Up | 4.30E-85 |
| Bra024377 | 918 | Up | 2.40E-08 |
| Bra024795 | 690 | Up | 0.000127442 |
| Bra003393 | 282 | Up | 0.000127425 |
| Bra020998 | 2325 | Up | 9.05E-10 |
| Bra020523 | 921 | Up | 7.09E-58 |
| Bra037302 | 1407 | Up | 1.44E-46 |
| Bra034128 | 1917 | Up | 5.45E-167 |
| Bra024345 | 1698 | Up | 1.56E-107 |
| Bra028367 | 1830 | Up | 1.96E-43 |
| Bra011822 | 882 | Up | 0.000996144 |
| Bra011899 | 810 | Up | 2.15E-09 |
| Bra011939 | 1365 | Up | 0.000996033 |
| Bra013196 | 396 | Up | 4.45E-07 |
| Bra021584 | 1002 | Up | 7.72E-12 |
| Bra030512 | 792 | Up | 1.17E-05 |
| Bra031135 | 1428 | Up | 1.17E-05 |
| Bra014692 | 858 | Up | 3.79E-15 |
| Bra023867 | 1503 | Up | 1.34E-07 |
| Bra008132 | 1020 | Up | 3.92E-126 |
| Bra004710 | 2928 | Up | 2.54E-249 |
| Bra010455 | 876 | Up | 4.13E-26 |
| Bra034122 | 486 | Up | 1.62E-40 |
| Bra029043 | 1086 | Up | 9.10E-05 |
| Bra002171 | 309 | Up | 1.05E-06 |
| Bra006467 | 309 | Up | 9.11E-05 |
| Bra020592 | 1428 | Up | 2.31E-23 |
| Bra015238 | 627 | Up | 2.18E-37 |
| Bra039081 | 1239 | Up | 2.74E-05 |
| Bra037791 | 1065 | Up | 9.55E-08 |
| Bra002624 | 576 | Up | 8.30E-06 |
| Bra039438 | 660 | Up | 2.88E-08 |
| Bra002153 | 4101 | Up | 8.30E-06 |
| Bra007961 | 780 | Up | 2.88E-08 |
| Bra023704 | 1170 | Up | 9.78E-11 |
| Bra037216 | 1260 | Up | 2.50E-06 |
| Bra035518 | 1611 | Up | 2.58E-09 |
| Bra015548 | 612 | Up | 3.75E-75 |
| Bra002584 | 741 | Up | 0.000705836 |
| Bra015899 | 1002 | Up | 9.63E-16 |
| Bra000344 | 1323 | Up | 0.000705916 |
| Bra024954 | 657 | Up | 2.26E-07 |
| Bra000069 | 1107 | Up | 2.26E-07 |
| Bra038949 | 1350 | Up | 0.000213663 |
| Bra037060 | 3477 | Up | 6.83E-08 |
| Bra016732 | 546 | Up | 2.14E-11 |
| Bra003333 | 3015 | Up | 1.54E-20 |
| Bra005743 | 261 | Up | 6.46E-05 |
| Bra037541 | 5289 | Up | 6.46E-05 |
| Bra030747 | 1413 | Up | 1.83E-09 |
| Bra003472 | 3282 | Up | 3.41E-40 |
| Bra039617 | 249 | Up | 1.95E-05 |
| Bra040762 | 1449 | Up | 9.54E-39 |
| Bra009824 | 579 | Up | 5.88E-06 |
| Bra023983 | 1038 | Up | 4.80E-12 |
| Bra009865 | 528 | Up | 5.31E-07 |
| Bra011584 | 1575 | Up | 5.06E-34 |
| Bra011771 | 981 | Up | 9.28E-15 |
| Bra006851 | 1155 | Up | 3.77E-07 |
| Bra016683 | 1005 | Up | 1.26E-06 |
| Bra010907 | 2376 | Up | 0.000499502 |
| Bra028999 | 567 | Up | 7.53E-14 |
| Bra040357 | 1848 | Up | 3.42E-08 |
| Bra022528 | 1002 | Up | 0.000499443 |
| Bra027655 | 2304 | Up | 1.53E-101 |
| Bra039182 | 627 | Up | 1.89E-21 |
| Bra015853 | 933 | Up | 5.71E-57 |
| Bra008855 | 1797 | Up | 1.78E-13 |
| Bra024686 | 750 | Up | 9.40E-38 |
| Bra010256 | 1932 | Up | 1.84E-12 |
| Bra021101 | 831 | Up | 1.95E-10 |
| Bra022892 | 1470 | Up | 1.41E-48 |
| Bra002822 | 1497 | Up | 4.15E-17 |
| Bra036295 | 642 | Up | 9.48E-13 |
| Bra034205 | 1833 | Up | 2.41E-24 |
| Bra014494 | 687 | Up | 2.99E-11 |
| Bra016886 | 1047 | Up | 6.28E-07 |
| Bra032579 | 3444 | Up | 2.13E-11 |
| Bra038405 | 1512 | Up | 2.30E-05 |
| Bra000814 | 1473 | Up | 1.34E-07 |
| Bra034024 | 546 | Up | 2.57E-09 |
| Bra001302 | 1221 | Up | 2.41E-33 |
| Bra032642 | 1611 | Up | 7.60E-05 |
| Bra013630 | 483 | Up | 7.60E-05 |
| Bra010519 | 609 | Up | 2.18E-14 |
| Bra034781 | 1974 | Up | 1.41E-141 |
| Bra031109 | 1545 | Up | 2.14E-28 |
| Bra003011 | 1464 | Up | 4.54E-15 |
| Bra034281 | 1944 | Up | 0.000249896 |
| Bra011821 | 1554 | Up | 4.98E-18 |
| Bra006756 | 1518 | Up | 8.16E-81 |
| Bra008750 | 963 | Up | 2.50E-12 |
| Bra013058 | 432 | Up | 2.02E-08 |
| Bra014550 | 1095 | Up | 2.02E-08 |
| Bra026064 | 1512 | Up | 3.89E-49 |
| Bra037702 | 270 | Up | 4.52E-110 |
| Bra027555 | 1602 | Up | 2.70E-10 |
| Bra016766 | 1713 | Up | 3.46E-06 |
| Bra037538 | 1212 | Up | 3.46E-06 |
| Bra032985 | 2352 | Up | 3.46E-06 |
| Bra026161 | 819 | Up | 2.22E-07 |
| Bra015975 | 1896 | Up | 4.35E-21 |
| Bra004771 | 849 | Up | 2.08E-28 |
| Bra014401 | 492 | Up | 9.36E-246 |
| Bra031811 | 1068 | Up | 4.10E-11 |
| Bra015522 | 3483 | Up | 1.17E-54 |
| Bra008566 | 2691 | Up | 6.59E-13 |
| Bra039815 | 1260 | Up | 2.44E-06 |
| Bra029482 | 1707 | Up | 3.37E-100 |
| Bra019320 | 2022 | Up | 1.86E-15 |
| Bra033242 | 786 | Up | 0.000581732 |
| Bra027092 | 468 | Up | 0.000581799 |
| Bra040207 | 441 | Up | 5.18E-07 |
| Bra024848 | 972 | Up | 3.75E-85 |
| Bra011461 | 1380 | Up | 3.31E-26 |
| Bra029481 | 957 | Up | 3.02E-60 |
| Bra028285 | 969 | Up | 0.000124652 |
| Bra030196 | 900 | Up | 3.62E-22 |
| Bra039811 | 2268 | Up | 7.67E-66 |
| Bra028521 | 1998 | Up | 2.38E-13 |
| Bra036140 | 483 | Up | 2.67E-05 |
| Bra030333 | 471 | Up | 2.67E-05 |
| Bra008167 | 2052 | Up | 5.59E-18 |
| Bra030785 | 663 | Up | 9.28E-17 |
| Bra039370 | 618 | Up | 1.13E-37 |
| Bra020355 | 1644 | Up | 1.21E-06 |
| Bra014794 | 2100 | Up | 0.000409145 |
| Bra005004 | 1326 | Up | 2.57E-07 |
| Bra007094 | 1821 | Up | 8.83E-05 |
| Bra027320 | 1539 | Up | 9.95E-65 |
| Bra021623 | 1053 | Up | 3.91E-13 |
| Bra021276 | 1065 | Up | 2.38E-11 |
| Bra038465 | 381 | Up | 8.54E-07 |
| Bra038965 | 681 | Up | 2.77E-13 |
| Bra039316 | 618 | Up | 1.81E-07 |
| Bra021636 | 1518 | Up | 3.86E-08 |
| Bra018162 | 726 | Up | 1.92E-19 |
| Bra011180 | 849 | Up | 2.39E-22 |
| Bra023598 | 1035 | Up | 6.45E-184 |
| Bra016667 | 489 | Up | 1.33E-05 |
| Bra024558 | 1746 | Up | 1.08E-28 |
| Bra026774 | 195 | Up | 1.28E-07 |
| Bra034554 | 690 | Up | 1.37E-13 |
| Bra040533 | 780 | Up | 2.74E-21 |
| Bra000517 | 1827 | Up | 7.54E-18 |
| Bra016592 | 2265 | Up | 1.99E-14 |
| Bra016894 | 1110 | Up | 8.96E-08 |
| Bra040375 | 699 | Up | 4.23E-16 |
| Bra030490 | 1497 | Up | 4.37E-05 |
| Bra033482 | 321 | Up | 1.01E-12 |
| Bra015640 | 1407 | Up | 1.34E-08 |
| Bra006206 | 1719 | Up | 3.97E-24 |
| Bra035230 | 411 | Up | 6.34E-08 |
| Bra039742 | 1494 | Up | 3.20E-22 |
| Bra019952 | 2460 | Up | 0.000951865 |
| Bra013467 | 2172 | Up | 0.000952076 |
| Bra013679 | 2064 | Up | 0.00095197 |
| Bra003675 | 1212 | Up | 3.70E-68 |
| Bra032088 | 822 | Up | 1.39E-09 |
| Bra005193 | 1143 | Up | 2.07E-10 |
| Bra027967 | 2097 | Up | 7.06E-68 |
| Bra000159 | 960 | Up | 1.04E-76 |
| Bra023925 | 1131 | Up | 6.88E-10 |
| Bra028893 | 1440 | Up | 7.01E-19 |
| Bra011835 | 1038 | Up | 6.89E-07 |
| Bra018609 | 1170 | Up | 3.24E-06 |
| Bra001840 | 1374 | Up | 0.000100679 |
| Bra017104 | 741 | Up | 7.18E-11 |
| Bra020433 | 783 | Up | 5.17E-170 |
| Bra022868 | 1017 | Up | 7.49E-29 |
| Bra020861 | 5826 | Up | 1.08E-08 |
| Bra035116 | 1548 | Up | 0.000468805 |
| Bra036803 | 1674 | Up | 0.000468861 |
| Bra004057 | 2676 | Up | 7.08E-05 |
| Bra012948 | 1527 | Up | 1.07E-05 |
| Bra039703 | 408 | Up | 3.09E-15 |
| Bra032293 | 2136 | Up | 7.39E-35 |
| Bra004892 | 3780 | Up | 6.90E-26 |
| Bra018940 | 3540 | Up | 4.03E-205 |
| Bra007044 | 1137 | Up | 7.67E-51 |
| Bra000194 | 774 | Up | 4.98E-05 |
| Bra018375 | 480 | Up | 4.98E-05 |
| Bra000031 | 603 | Up | 7.46E-06 |
| Bra026932 | 2184 | Up | 2.49E-08 |
| Bra013956 | 2199 | Up | 1.11E-06 |
| Bra034164 | 912 | Up | 1.12E-06 |
| Bra025432 | 1587 | Up | 4.98E-05 |
| Bra003789 | 1704 | Up | 9.14E-23 |
| Bra008037 | 2760 | Up | 3.57E-57 |
| Bra014638 | 882 | Up | 1.76E-08 |
| Bra013423 | 891 | Up | 1.17E-07 |
| Bra010128 | 627 | Up | 7.84E-07 |
| Bra021417 | 549 | Up | 7.06E-105 |
| Bra035535 | 381 | Up | 4.04E-87 |
| Bra010943 | 612 | Up | 0.000232335 |
| Bra007985 | 1356 | Up | 7.28E-22 |
| Bra003077 | 555 | Up | 1.27E-09 |
| Bra035257 | 594 | Up | 1.27E-09 |
| Bra032202 | 1269 | Up | 5.79E-08 |
| Bra007940 | 1245 | Up | 8.93E-10 |
| Bra000589 | 921 | Up | 2.58E-06 |
| Bra003126 | 2124 | Up | 1.01E-11 |
| Bra003145 | 987 | Up | 3.39E-33 |
| Bra000925 | 1032 | Up | 1.72E-05 |
| Bra025340 | 1578 | Up | 9.31E-15 |
| Bra031627 | 1203 | Up | 3.65E-95 |
| Bra000485 | 723 | Up | 1.94E-23 |
| Bra034404 | 2739 | Up | 1.43E-122 |
| Bra008976 | 495 | Up | 1.27E-06 |
| Bra030704 | 312 | Up | 1.27E-06 |
| Bra038650 | 2232 | Up | 1.33E-07 |
| Bra034765 | 1782 | Up | 1.44E-09 |
| Bra035006 | 993 | Up | 2.01E-128 |
| Bra040122 | 678 | Up | 9.28E-08 |
| Bra026418 | 1926 | Up | 6.76E-13 |
| Bra004389 | 933 | Up | 5.26E-49 |
| Bra018177 | 1827 | Up | 5.82E-53 |
| Bra000450 | 195 | Up | 0.000529065 |
| Bra022786 | 1137 | Up | 4.15E-06 |
| Bra002286 | 2553 | Up | 2.13E-12 |
| Bra009809 | 849 | Up | 3.95E-05 |
| Bra006182 | 774 | Up | 4.49E-139 |
| Bra018942 | 1143 | Up | 1.34E-25 |
| Bra008981 | 1053 | Up | 8.46E-16 |
| Bra028191 | 1503 | Up | 1.18E-13 |
| Bra015514 | 756 | Up | 0.00037079 |
| Bra032875 | 1365 | Up | 0.000370701 |
| Bra018380 | 390 | Up | 0.000370745 |
| Bra025805 | 798 | Up | 0.000370611 |
| Bra010610 | 1662 | Up | 0.000370656 |
| Bra031567 | 1488 | Up | 5.78E-15 |
| Bra003032 | 684 | Up | 7.60E-13 |
| Bra029677 | 2181 | Up | 9.46E-21 |
| Bra012324 | 1473 | Up | 2.03E-06 |
| Bra016993 | 1749 | Up | 7.26E-29 |
| Bra026114 | 1347 | Up | 4.22E-46 |
| Bra007333 | 2295 | Up | 6.12E-44 |
| Bra025406 | 1311 | Up | 0.000259785 |
| Bra000539 | 846 | Up | 7.37E-25 |
| Bra017390 | 2046 | Up | 3.19E-19 |
| Bra003163 | 480 | Up | 8.59E-117 |
| Bra027784 | 759 | Up | 3.74E-09 |
| Bra029852 | 1551 | Up | 5.14E-08 |
| Bra017287 | 1269 | Up | 5.77E-24 |
| Bra013823 | 1338 | Up | 1.83E-09 |
| Bra026022 | 450 | Up | 0.00012768 |
| Bra028498 | 1950 | Up | 1.88E-12 |
| Bra001721 | 651 | Up | 4.63E-06 |
| Bra003691 | 2559 | Up | 3.47E-27 |
| Bra029950 | 954 | Up | 3.26E-06 |
| Bra000558 | 1596 | Up | 1.13E-11 |
| Bra003842 | 927 | Up | 1.13E-11 |
| Bra016150 | 723 | Up | 2.28E-06 |
| Bra023717 | 858 | Up | 2.28E-06 |
| Bra040695 | 810 | Up | 1.49E-10 |
| Bra002416 | 1482 | Up | 1.69E-24 |
| Bra003155 | 339 | Up | 8.20E-08 |
| Bra014308 | 2202 | Up | 8.19E-15 |
| Bra019409 | 4017 | Up | 1.60E-06 |
| Bra017318 | 1167 | Up | 6.07E-58 |
| Bra018196 | 3606 | Up | 4.00E-08 |
| Bra028840 | 1239 | Up | 3.08E-05 |
| Bra021215 | 2016 | Up | 0.000843634 |
| Bra018401 | 762 | Up | 3.08E-05 |
| Bra004439 | 621 | Up | 0.000843539 |
| Bra009734 | 942 | Up | 0.000843445 |
| Bra007297 | 4038 | Up | 1.11E-06 |
| Bra023022 | 1317 | Up | 9.97E-10 |
| Bra011573 | 1647 | Up | 6.98E-70 |
| Bra021550 | 1290 | Up | 7.81E-07 |
| Bra015946 | 2010 | Up | 1.83E-83 |
| Bra018553 | 2004 | Up | 1.89E-14 |
| Bra019836 | 1506 | Up | 5.43E-07 |
| Bra037700 | 1806 | Up | 0.000589432 |
| Bra023336 | 1767 | Up | 6.45E-63 |
| Bra029971 | 240 | Up | 9.14E-23 |
| Bra000461 | 2058 | Up | 1.88E-48 |
| Bra007234 | 531 | Up | 9.60E-09 |
| Bra020700 | 1788 | Up | 6.70E-09 |
| Bra034315 | 1323 | Up | 1.18E-44 |
| Bra017278 | 1239 | Up | 5.88E-36 |
| Bra020903 | 396 | Up | 0.000289395 |
| Bra040280 | 348 | Up | 0.000289467 |
| Bra009117 | 1362 | Up | 0.000289431 |
| Bra036884 | 588 | Up | 1.53E-55 |
| Bra016163 | 1620 | Up | 3.35E-64 |
| Bra007463 | 441 | Up | 4.02E-11 |
| Bra041148 | 600 | Up | 0 |
| Bra020167 | 1113 | Up | 1.59E-09 |
| Bra015570 | 1545 | Up | 1.04E-160 |
| Bra037480 | 786 | Up | 2.92E-27 |
| Bra021052 | 2379 | Up | 0.00020294 |
| Bra032083 | 1242 | Up | 3.61E-06 |
| Bra025691 | 1179 | Up | 0.000202965 |
| Bra003487 | 1503 | Up | 3.81E-24 |
| Bra008743 | 1107 | Up | 0.000142182 |
| Bra015613 | 1455 | Up | 1.70E-36 |
| Bra022336 | 1707 | Up | 1.77E-06 |
| Bra029145 | 1524 | Up | 1.77E-06 |
| Bra023524 | 1287 | Up | 1.77E-06 |
| Bra031666 | 951 | Up | 4.86E-14 |
| Bra008264 | 1197 | Up | 7.03E-20 |
| Bra039847 | 909 | Up | 9.94E-05 |
| Bra023844 | 1926 | Up | 3.63E-28 |
| Bra034466 | 1440 | Up | 2.20E-145 |
| Bra003559 | 2139 | Up | 6.51E-13 |
| Bra036754 | 861 | Up | 5.18E-09 |
| Bra007841 | 735 | Up | 1.21E-46 |
| Bra036698 | 1731 | Up | 4.89E-05 |
| Bra024475 | 579 | Up | 4.89E-05 |
| Bra032539 | 867 | Up | 3.62E-09 |
| Bra025209 | 546 | Up | 2.95E-07 |
| Bra004424 | 309 | Up | 2.06E-07 |
| Bra035773 | 3015 | Up | 1.51E-29 |
| Bra000560 | 1167 | Up | 4.71E-23 |
| Bra026230 | 1650 | Up | 5.06E-125 |
| Bra000150 | 2148 | Up | 2.09E-16 |
| Bra007051 | 933 | Up | 3.90E-31 |
| Bra010220 | 927 | Up | 2.34E-17 |
| Bra035544 | 1737 | Up | 1.17E-05 |
| Bra010728 | 519 | Up | 5.40E-18 |
| Bra034004 | 1134 | Up | 8.19E-06 |
| Bra014371 | 1170 | Up | 2.41E-08 |
| Bra037647 | 2412 | Up | 2.41E-08 |
| Bra014702 | 498 | Up | 2.41E-08 |
| Bra033473 | 1146 | Up | 7.16E-27 |
| Bra032368 | 2445 | Up | 4.00E-06 |
| Bra016426 | 2202 | Up | 7.49E-22 |
| Bra038776 | 1959 | Up | 0.000931795 |
| Bra001123 | 645 | Up | 0.000931898 |
| Bra037351 | 1533 | Up | 1.57E-12 |
| Bra038080 | 1431 | Up | 0.00065161 |
| Bra004499 | 975 | Up | 0.000651535 |
| Bra036623 | 2328 | Up | 4.62E-10 |
| Bra034729 | 1131 | Up | 1.20E-191 |
| Bra013103 | 330 | Up | 0.000455282 |
| Bra006843 | 666 | Up | 3.26E-07 |
| Bra020688 | 1185 | Up | 0.000455336 |
| Bra040711 | 1599 | Up | 3.26E-07 |
| Bra014622 | 2925 | Up | 1.48E-23 |
| Bra017182 | 1410 | Up | 4.13E-17 |
| Bra000963 | 2919 | Up | 1.11E-07 |
| Bra023339 | 195 | Up | 4.71E-12 |
| Bra005189 | 435 | Up | 0.000109497 |
| Bra015881 | 648 | Up | 1.30E-08 |
| Bra035382 | 360 | Up | 6.33E-09 |
| Bra022618 | 1821 | Up | 3.76E-05 |
| Bra008437 | 1599 | Up | 3.76E-05 |
| Bra016099 | 1101 | Up | 1.05E-13 |
| Bra021801 | 414 | Up | 1.05E-09 |
| Bra037731 | 2631 | Up | 0 |
| Bra008848 | 303 | Up | 1.84E-05 |
| Bra001729 | 1734 | Up | 2.27E-34 |
| Bra009771 | 1257 | Up | 3.32E-21 |
| Bra000620 | 1209 | Up | 1.45E-11 |
| Bra008473 | 495 | Up | 7.21E-12 |
| Bra030423 | 558 | Up | 1.51E-06 |
| Bra034383 | 1758 | Up | 5.27E-168 |
| Bra035869 | 1482 | Up | 1.05E-06 |
| Bra016230 | 1137 | Up | 7.36E-07 |
| Bra036740 | 1419 | Up | 9.49E-13 |
| Bra006516 | 900 | Up | 2.36E-13 |
| Bra029485 | 1758 | Up | 1.76E-07 |
| Bra027285 | 1209 | Up | 1.24E-83 |
| Bra028372 | 822 | Up | 9.46E-23 |
| Bra004127 | 2073 | Up | 2.04E-08 |
| Bra036782 | 1239 | Up | 2.72E-33 |
| Bra007699 | 1518 | Up | 1.37E-29 |
| Bra018197 | 2472 | Up | 1.21E-20 |
| Bra020090 | 585 | Up | 6.52E-11 |
| Bra027696 | 456 | Up | 6.91E-34 |
| Bra017100 | 3108 | Up | 3.70E-13 |
| Bra003349 | 1536 | Up | 6.74E-18 |
| Bra025905 | 876 | Up | 1.56E-18 |
| Bra015346 | 894 | Up | 3.06E-21 |
| Bra027073 | 1506 | Up | 1.65E-22 |
| Bra023577 | 1161 | Up | 8.78E-24 |
| Bra000317 | 1959 | Up | 1.94E-41 |
| Bra006943 | 417 | Up | 2.74E-07 |
| Bra024667 | 795 | Up | 4.12E-05 |
| Bra009080 | 279 | Up | 6.88E-06 |
| Bra036492 | 894 | Up | 0.000348795 |
| Bra010713 | 726 | Up | 4.81E-06 |
| Bra034316 | 750 | Up | 2.10E-27 |
| Bra027795 | 1593 | Up | 2.74E-19 |
| Bra008972 | 1791 | Up | 0.00011954 |
| Bra022106 | 744 | Up | 1.46E-10 |
| Bra033772 | 1188 | Up | 0.000498152 |
| Bra006306 | 1287 | Up | 2.87E-05 |
| Bra008979 | 1851 | Up | 2.87E-05 |
| Bra014539 | 870 | Up | 0.00071364 |
| Bra018433 | 954 | Up | 0.000245171 |
| Bra006911 | 1074 | Up | 0.000172006 |
| Bra011280 | 1539 | Up | 1.09E-08 |
| Bra027952 | 2505 | Up | 6.88E-06 |
| Bra028055 | 1062 | Up | 1.90E-19 |
| Bra017008 | 2232 | Up | 1.25E-29 |
| Bra030653 | 999 | Up | 8.56E-36 |
| Bra033958 | 2946 | Up | 4.32E-85 |
| Bra003402 | 4485 | Up | 1.86E-38 |
| Bra026364 | 879 | Up | 2.54E-34 |
| Bra016153 | 1110 | Up | 5.06E-17 |
| Bra036488 | 2502 | Up | 4.53E-16 |
| Bra035183 | 1080 | Up | 3.26E-10 |
| Bra023829 | 2712 | Up | 4.66E-10 |
| Bra014712 | 612 | Up | 6.70E-10 |
| Bra016866 | 2895 | Up | 5.78E-200 |
| Bra035734 | 840 | Up | 3.94E-33 |
| Bra028132 | 1071 | Up | 1.53E-73 |
| Bra025728 | 741 | Up | 1.57E-47 |
| Bra036703 | 1179 | Up | 4.36E-15 |
| Bra001774 | 1626 | Up | 3.66E-06 |
| Bra017301 | 1485 | Up | 7.49E-06 |
| Bra025668 | 1014 | Up | 3.55E-10 |
| Bra019578 | 312 | Up | 4.48E-05 |
| Bra008711 | 3690 | Up | 4.47E-05 |
| Bra033744 | 984 | Up | 6.39E-05 |
| Bra019366 | 1578 | Up | 7.57E-21 |
| Bra013475 | 1608 | Up | 5.55E-12 |
| Bra039010 | 1092 | Up | 0.000186258 |
| Bra018435 | 1797 | Up | 9.22E-22 |
| Bra034681 | 3267 | Up | 1.11E-07 |
| Bra009560 | 1221 | Up | 4.94E-31 |
| Bra004743 | 759 | Up | 2.27E-07 |
| Bra026815 | 828 | Up | 0.000541606 |
| Bra014616 | 1473 | Up | 3.09E-16 |
| Bra021385 | 561 | Up | 0.000541543 |
| Bra028975 | 708 | Up | 3.20E-19 |
| Bra007585 | 891 | Up | 4.96E-47 |
| Bra027961 | 912 | Up | 7.88E-13 |
| Bra008728 | 1689 | Up | 0.000776326 |
| Bra021531 | 387 | Up | 0.000776238 |
| Bra010227 | 753 | Up | 1.51E-59 |
| Bra029200 | 2163 | Up | 1.19E-11 |
| Bra009182 | 648 | Up | 6.86E-09 |
| Bra003881 | 357 | Up | 3.98E-06 |
| Bra037056 | 453 | Up | 5.69E-06 |
| Bra028125 | 1386 | Up | 5.69E-06 |
| Bra040520 | 1002 | Up | 0 |
| Bra037096 | 1194 | Up | 1.66E-05 |
| Bra019932 | 762 | Up | 6.00E-10 |
| Bra016185 | 498 | Up | 8.59E-10 |
| Bra024934 | 1632 | Up | 6.46E-12 |
| Bra013674 | 696 | Up | 4.95E-104 |
| Bra014696 | 681 | Up | 3.52E-07 |
| Bra018658 | 2064 | Up | 3.52E-07 |
| Bra008991 | 660 | Up | 3.52E-07 |
| Bra026787 | 1551 | Up | 4.85E-05 |
| Bra002407 | 1413 | Up | 7.42E-09 |
| Bra017524 | 903 | Up | 3.55E-12 |
| Bra021979 | 2580 | Up | 1.03E-36 |
| Bra008470 | 1368 | Up | 6.89E-161 |
| Bra016375 | 1695 | Up | 2.09E-26 |
| Bra028047 | 1293 | Up | 0.000201127 |
| Bra006972 | 1506 | Up | 0.000201153 |
| Bra035641 | 1134 | Up | 2.65E-32 |
| Bra035070 | 1326 | Up | 6.14E-06 |
| Bra018800 | 1107 | Up | 3.90E-09 |
| Bra031361 | 357 | Up | 0.000407547 |
| Bra027940 | 765 | Up | 3.02E-88 |
| Bra029832 | 1236 | Up | 1.50E-11 |
| Bra036166 | 597 | Up | 5.41E-07 |
| Bra001919 | 2157 | Up | 1.79E-05 |
| Bra011047 | 648 | Up | 5.16E-53 |
| Bra026494 | 1008 | Up | 0.000834106 |
| Bra006245 | 771 | Up | 0.000833919 |
| Bra034373 | 648 | Up | 0.000834012 |
| Bra039976 | 816 | Up | 4.66E-18 |
| Bra039130 | 861 | Up | 4.03E-15 |
| Bra032550 | 1161 | Up | 1.74E-32 |
| Bra025285 | 1596 | Up | 3.65E-05 |
| Bra038398 | 282 | Up | 2.05E-09 |
| Bra024237 | 1506 | Up | 1.22E-23 |
| Bra012803 | 1272 | Up | 6.87E-08 |
| Bra039356 | 1686 | Up | 8.34E-15 |
| Bra014277 | 3063 | Up | 1.25E-10 |
| Bra031807 | 2694 | Up | 1.35E-121 |
| Bra010564 | 3147 | Up | 1.79E-10 |
| Bra035413 | 1719 | Up | 8.64E-41 |
| Bra004024 | 480 | Up | 7.38E-14 |
| Bra013513 | 1200 | Up | 4.76E-24 |
| Bra029583 | 1959 | Up | 1.33E-27 |
| Bra000929 | 342 | Up | 1.29E-14 |
| Bra011148 | 1497 | Up | 1.29E-14 |
| Bra006860 | 360 | Up | 4.07E-07 |
| Bra029142 | 987 | Up | 6.11E-36 |
| Bra008840 | 1656 | Up | 3.76E-18 |
| Bra015481 | 759 | Up | 5.82E-07 |
| Bra013786 | 2136 | Up | 1.87E-63 |
| Bra024257 | 1086 | Up | 1.19E-06 |
| Bra026484 | 480 | Up | 9.51E-23 |
| Bra040576 | 2076 | Up | 7.20E-40 |
| Bra036169 | 1191 | Up | 2.71E-59 |
| Bra004633 | 1461 | Up | 3.61E-67 |
| Bra029585 | 6627 | Up | 8.07E-30 |
| Bra000292 | 2724 | Up | 4.18E-57 |
| Bra010115 | 1755 | Up | 2.14E-07 |
| Bra008443 | 792 | Up | 4.35E-18 |
| Bra020666 | 3264 | Up | 3.76E-60 |
| Bra016604 | 813 | Up | 1.74E-13 |
| Bra013275 | 1884 | Up | 5.25E-21 |
| Bra014801 | 1848 | Up | 6.35E-22 |
| Bra018128 | 3123 | Up | 1.55E-16 |
| Bra029486 | 537 | Up | 4.95E-06 |
| Bra008657 | 825 | Up | 3.25E-18 |
| Bra029849 | 1512 | Up | 5.57E-05 |
| Bra032831 | 2403 | Up | 5.53E-08 |
| Bra040663 | 1119 | Up | 5.53E-08 |
| Bra013203 | 1059 | Up | 1.40E-19 |
| Bra000869 | 10728 | Up | 1.08E-10 |
| Bra035696 | 771 | Up | 7.97E-05 |
| Bra020016 | 999 | Up | 7.98E-05 |
| Bra010043 | 630 | Up | 7.97E-05 |
| Bra026916 | 1107 | Up | 2.06E-64 |
| Bra015560 | 1530 | Up | 1.44E-05 |
| Bra035195 | 633 | Up | 1.44E-05 |
| Bra022367 | 828 | Up | 1.82E-06 |
| Bra034307 | 1410 | Up | 1.78E-16 |
| Bra011500 | 777 | Up | 6.09E-38 |
| Bra026540 | 957 | Up | 1.63E-87 |
| Bra039026 | 846 | Up | 8.12E-11 |
| Bra008343 | 936 | Up | 3.78E-39 |
| Bra037620 | 813 | Up | 2.46E-14 |
| Bra026917 | 1023 | Up | 6.19E-15 |
| Bra011201 | 753 | Up | 1.76E-44 |
| Bra033743 | 1614 | Up | 2.93E-05 |
| Bra009047 | 1893 | Up | 0.000231446 |
| Bra002745 | 1131 | Up | 0.000231475 |
| Bra024634 | 1404 | Up | 2.25E-15 |
| Bra009029 | 531 | Up | 5.68E-12 |
| Bra039818 | 822 | Up | 1.83E-14 |
| Bra033039 | 981 | Up | 5.29E-06 |
| Bra037520 | 2769 | Up | 6.75E-41 |
| Bra040406 | 831 | Up | 1.83E-83 |
| Bra013394 | 1176 | Up | 1.52E-80 |
| Bra034696 | 846 | Up | 2.17E-08 |
| Bra011586 | 1113 | Up | 0.000327344 |
| Bra014253 | 897 | Up | 0.000327384 |
| Bra016773 | 1326 | Up | 1.72E-07 |
| Bra013856 | 1134 | Up | 4.33E-13 |
| Bra029046 | 1344 | Up | 6.10E-13 |
| Bra024603 | 1668 | Up | 8.51E-05 |
| Bra003055 | 2001 | Up | 1.23E-22 |
| Bra032267 | 2112 | Up | 4.98E-07 |
| Bra007791 | 2439 | Up | 8.97E-08 |
| Bra019343 | 660 | Up | 2.88E-09 |
| Bra039756 | 1071 | Up | 6.70E-221 |
| Bra007309 | 1980 | Up | 1.91E-24 |
| Bra032396 | 1257 | Up | 1.70E-13 |
| Bra036902 | 1230 | Up | 1.28E-07 |
| Bra009959 | 1035 | Up | 0.00066599 |
| Bra003774 | 327 | Up | 0.000120619 |
| Bra000280 | 1017 | Up | 4.12E-09 |
| Bra039314 | 744 | Up | 3.95E-06 |
| Bra001009 | 861 | Up | 2.32E-16 |
| Bra013450 | 732 | Up | 6.79E-23 |
| Bra009642 | 1863 | Up | 3.29E-08 |
| Bra028708 | 789 | Up | 6.48E-58 |
| Bra034975 | 3735 | Up | 1.05E-24 |
| Bra031248 | 1572 | Up | 2.51E-12 |
| Bra002388 | 1377 | Up | 4.70E-08 |
| Bra011669 | 1611 | Up | 6.92E-11 |
| Bra021653 | 2400 | Up | 3.66E-44 |
| Bra030858 | 2070 | Up | 0 |
| Bra009383 | 2772 | Up | 8.05E-06 |
| Bra001416 | 2148 | Up | 1.99E-20 |
| Bra000310 | 849 | Up | 2.00E-32 |
| Bra019243 | 1203 | Up | 0.000245625 |
| Bra027685 | 1464 | Up | 1.99E-42 |
| Bra040582 | 1638 | Up | 3.19E-17 |
| Bra001098 | 441 | Up | 4.38E-09 |
| Bra015727 | 546 | Up | 1.66E-41 |
| Bra010122 | 858 | Up | 1.15E-05 |
| Bra040046 | 1098 | Up | 5.29E-07 |
| Bra004937 | 1107 | Up | 1.27E-36 |
| Bra005495 | 693 | Up | 1.64E-17 |
| Bra020877 | 1275 | Up | 3.50E-08 |
| Bra030339 | 1755 | Up | 1.63E-05 |
| Bra017161 | 2076 | Up | 8.98E-09 |
| Bra026135 | 786 | Up | 4.19E-06 |
| Bra016702 | 810 | Up | 1.60E-35 |
| Bra041147 | 630 | Up | 9.04E-05 |
| Bra037470 | 660 | Up | 2.52E-14 |
| Bra007250 | 774 | Up | 3.88E-11 |
| Bra008823 | 1845 | Up | 8.34E-10 |
| Bra012008 | 1386 | Up | 2.48E-50 |
| Bra028087 | 1440 | Up | 4.95E-60 |
| Bra036788 | 351 | Up | 2.68E-57 |
| Bra034377 | 1377 | Up | 1.65E-38 |
| Bra022180 | 1932 | Up | 2.05E-13 |
| Bra005241 | 1491 | Up | 2.78E-24 |
| Bra007671 | 2157 | Up | 8.29E-16 |
| Bra015499 | 2079 | Up | 1.05E-164 |
| Bra002440 | 600 | Up | 0.000495914 |
| Bra024425 | 237 | Up | 7.45E-14 |
| Bra025281 | 1566 | Up | 1.70E-09 |
| Bra032737 | 999 | Up | 8.55E-06 |
| Bra039337 | 1101 | Up | 4.61E-39 |
| Bra022980 | 852 | Up | 2.19E-06 |
| Bra022931 | 1674 | Up | 1.52E-24 |
| Bra033806 | 2439 | Up | 1.43E-35 |
| Bra020123 | 753 | Up | 2.93E-71 |
| Bra001578 | 1116 | Up | 5.69E-22 |
| Bra007434 | 4356 | Up | 4.82E-26 |
| Bra034813 | 453 | Up | 1.22E-05 |
| Bra038444 | 594 | Up | 3.13E-06 |
| Bra031935 | 540 | Up | 0.000182976 |
| Bra027731 | 1035 | Up | 8.02E-07 |
| Bra032572 | 2316 | Up | 0.000706827 |
| Bra037664 | 1788 | Up | 4.72E-05 |
| Bra031959 | 1635 | Up | 3.13E-06 |
| Bra008265 | 975 | Up | 4.98E-15 |
| Bra025480 | 1464 | Up | 1.84E-37 |
| Bra022425 | 1098 | Up | 2.25E-30 |
| Bra018268 | 441 | Up | 6.57E-10 |
| Bra036139 | 591 | Up | 9.36E-10 |
| Bra019637 | 690 | Up | 3.77E-52 |
| Bra033773 | 543 | Up | 1.44E-08 |
| Bra009542 | 693 | Up | 1.10E-73 |
| Bra038381 | 732 | Up | 3.41E-10 |
| Bra004893 | 1212 | Up | 9.09E-13 |
| Bra034160 | 2187 | Up | 3.51E-05 |
| Bra023277 | 540 | Up | 3.51E-05 |
| Bra003190 | 1146 | Up | 1.56E-44 |
| Bra021829 | 1389 | Up | 2.72E-09 |
| Bra030666 | 3042 | Up | 1.55E-70 |
| Bra005341 | 1038 | Up | 5.40E-28 |
| Bra039827 | 747 | Up | 0.000523874 |
| Bra009101 | 618 | Up | 7.07E-32 |
| Bra037891 | 1050 | Up | 1.31E-10 |
| Bra023579 | 552 | Up | 3.99E-16 |
| Bra022859 | 750 | Up | 5.17E-13 |
| Bra020061 | 1353 | Up | 1.72E-06 |
| Bra025264 | 1332 | Up | 0.000192842 |
| Bra026919 | 3621 | Up | 2.41E-24 |
| Bra005026 | 1293 | Up | 1.29E-54 |
| Bra035801 | 1047 | Up | 9.73E-11 |
| Bra037006 | 1440 | Up | 2.60E-05 |
| Bra028826 | 1050 | Up | 6.02E-16 |
| Bra039653 | 2322 | Up | 1.20E-07 |
| Bra002257 | 1149 | Up | 1.49E-09 |
| Bra037948 | 2160 | Up | 3.44E-15 |
| Bra025250 | 1029 | Up | 7.10E-12 |
| Bra018806 | 1539 | Up | 9.31E-19 |
| Bra034985 | 201 | Up | 1.36E-05 |
| Bra031349 | 1809 | Up | 1.05E-17 |
| Bra025730 | 699 | Up | 9.09E-16 |
| Bra000371 | 2217 | Up | 4.95E-06 |
| Bra002954 | 1398 | Up | 1.69E-52 |
| Bra020369 | 906 | Up | 4.58E-23 |
| Bra015510 | 1776 | Up | 0.000142965 |
| Bra002841 | 1572 | Up | 5.23E-05 |
| Bra016945 | 864 | Up | 1.93E-05 |
| Bra034424 | 474 | Up | 1.92E-05 |
| Bra010693 | 498 | Up | 2.27E-34 |
| Bra023104 | 501 | Up | 2.92E-14 |
| Bra020721 | 1344 | Up | 1.26E-07 |
| Bra013431 | 7110 | Up | 1.28E-57 |
| Bra013204 | 438 | Up | 1.65E-13 |
| Bra003588 | 894 | Up | 5.65E-11 |
| Bra023450 | 1221 | Up | 3.46E-20 |
| Bra038432 | 1671 | Up | 2.73E-05 |
| Bra002976 | 315 | Up | 1.00E-05 |
| Bra035249 | 3369 | Up | 4.90E-07 |
| Bra031834 | 708 | Up | 0.000549744 |
| Bra011002 | 1080 | Up | 2.35E-13 |
| Bra011919 | 1827 | Up | 0 |
| Bra030183 | 3459 | Up | 1.22E-13 |
| Bra004884 | 2268 | Up | 3.40E-39 |
| Bra005434 | 3801 | Up | 1.42E-05 |
| Bra006413 | 546 | Up | 1.78E-08 |
| Bra018636 | 963 | Up | 0.000287467 |
| Bra031291 | 1143 | Up | 0.000287431 |
| Bra039995 | 1461 | Up | 1.01E-16 |
| Bra036672 | 1470 | Up | 3.62E-07 |
| Bra012614 | 939 | Up | 9.88E-07 |
| Bra004927 | 729 | Up | 0.000785387 |
| Bra025082 | 462 | Up | 9.17E-33 |
| Bra016755 | 384 | Up | 2.71E-06 |
| Bra030986 | 900 | Up | 4.22E-46 |
| Bra032750 | 1458 | Up | 1.88E-175 |
| Bra003159 | 678 | Up | 1.31E-08 |
| Bra040507 | 831 | Up | 0.000150137 |
| Bra027049 | 963 | Up | 1.00E-245 |
| Bra026125 | 2595 | Up | 1.27E-09 |
| Bra016908 | 1509 | Up | 2.41E-10 |
| Bra001125 | 2037 | Up | 1.39E-07 |
| Bra010977 | 717 | Up | 9.66E-09 |
| Bra007656 | 684 | Up | 7.82E-05 |
| Bra020538 | 3456 | Up | 2.25E-56 |
| Bra025660 | 1617 | Up | 7.03E-59 |
| Bra003391 | 276 | Up | 0.00021269 |
| Bra038220 | 1023 | Up | 1.42E-13 |
| Bra013865 | 771 | Up | 3.07E-90 |
| Bra000564 | 3048 | Up | 3.74E-14 |
| Bra040793 | 1092 | Up | 0.000576816 |
| Bra010437 | 369 | Up | 5.38E-07 |
| Bra034941 | 1500 | Up | 1.62E-94 |
| Bra013533 | 1203 | Up | 2.12E-05 |
| Bra025806 | 1599 | Up | 4.03E-06 |
| Bra026384 | 897 | Up | 0.000301126 |
| Bra040173 | 459 | Up | 0.0003012 |
| Bra000551 | 768 | Up | 0.000301163 |
| Bra000272 | 429 | Up | 5.75E-05 |
| Bra030472 | 3507 | Up | 7.22E-55 |
| Bra002715 | 2520 | Up | 2.09E-06 |
| Bra008931 | 1461 | Up | 1.92E-20 |
| Bra025563 | 1050 | Up | 0.000157342 |
| Bra031555 | 687 | Up | 3.01E-37 |
| Bra005212 | 852 | Up | 0 |
| Bra028669 | 468 | Up | 2.04E-08 |
| Bra017596 | 624 | Up | 2.52E-106 |
| Bra028101 | 1722 | Up | 8.18E-05 |
| Bra026080 | 810 | Up | 2.01E-20 |
| Bra032726 | 3177 | Up | 0.000424813 |
| Bra021027 | 1233 | Up | 8.12E-06 |
| Bra025541 | 1119 | Up | 2.83E-09 |
| Bra025713 | 765 | Up | 2.30E-13 |
| Bra018439 | 756 | Up | 4.25E-05 |
| Bra005919 | 996 | Up | 5.57E-40 |
| Bra008187 | 939 | Up | 8.01E-07 |
| Bra002181 | 4101 | Up | 4.20E-06 |
| Bra013202 | 1389 | Up | 4.14E-07 |
| Bra022668 | 642 | Up | 4.10E-08 |
| Bra040743 | 2625 | Up | 7.89E-21 |
| Bra037777 | 435 | Up | 8.58E-13 |
| Bra026988 | 1380 | Up | 3.91E-11 |
| Bra028353 | 2382 | Up | 8.32E-15 |
| Bra000690 | 1863 | Up | 5.31E-48 |
| Bra007914 | 1833 | Up | 5.97E-06 |
| Bra008876 | 894 | Up | 5.14E-128 |
| Bra038521 | 399 | Up | 3.06E-07 |
| Bra031046 | 627 | Up | 3.11E-06 |
| Bra000942 | 966 | Up | 3.11E-06 |
| Bra034674 | 1470 | Up | 8.42E-94 |
| Bra001131 | 1203 | Up | 0.000164179 |
| Bra038252 | 2286 | Up | 6.58E-21 |
| Bra037029 | 1440 | Up | 4.45E-66 |
| Bra008582 | 1752 | Up | 4.43E-05 |
| Bra034778 | 1134 | Up | 0.000854392 |
| Bra020731 | 1497 | Up | 2.43E-12 |
| Bra015538 | 3012 | Up | 6.59E-98 |
| Bra034399 | 642 | Up | 2.10E-18 |
| Bra016791 | 414 | Up | 0 |
| Bra024699 | 1755 | Up | 2.13E-30 |
| Bra008602 | 2913 | Up | 3.85E-94 |
| Bra009327 | 471 | Up | 3.24E-06 |
| Bra016602 | 2760 | Up | 1.84E-15 |
| Bra026834 | 1293 | Up | 0.000120086 |
| Bra031390 | 1170 | Up | 0.00012007 |
| Bra033892 | 2556 | Up | 1.68E-06 |
| Bra016114 | 1839 | Up | 4.22E-25 |
| Bra015504 | 5766 | Up | 3.21E-09 |
| Bra000659 | 1026 | Up | 1.66E-09 |
| Bra002113 | 333 | Up | 9.32E-17 |
| Bra021548 | 1779 | Up | 1.06E-45 |
| Bra032120 | 2823 | Up | 9.15E-24 |
| Bra030799 | 1674 | Up | 2.59E-108 |
| Bra032470 | 978 | Up | 1.73E-07 |
| Bra010498 | 2154 | Up | 4.31E-40 |
| Bra002244 | 999 | Up | 0.000170983 |
| Bra008107 | 1896 | Up | 6.97E-24 |
| Bra026571 | 1824 | Up | 1.25E-08 |
| Bra018631 | 681 | Up | 1.25E-05 |
| Bra001709 | 2814 | Up | 1.25E-05 |
| Bra022978 | 636 | Up | 1.46E-15 |
| Bra011407 | 1434 | Up | 1.01E-50 |
| Bra040287 | 543 | Up | 2.43E-07 |
| Bra036228 | 1596 | Up | 8.21E-30 |
| Bra004231 | 351 | Up | 0.000461108 |
| Bra007091 | 2118 | Up | 0.000124937 |
| Bra007296 | 1212 | Up | 0.000888225 |
| Bra037309 | 618 | Up | 2.34E-36 |
| Bra003236 | 525 | Up | 3.45E-07 |
| Bra032332 | 2865 | Up | 3.65E-34 |
| Bra005386 | 561 | Up | 1.96E-18 |
| Bra020836 | 1488 | Up | 8.73E-102 |
| Bra028006 | 1440 | Up | 3.47E-28 |
| Bra037042 | 1473 | Up | 3.61E-20 |
| Bra011035 | 873 | Up | 9.65E-10 |
| Bra037117 | 2205 | Up | 4.79E-05 |
| Bra008691 | 1287 | Up | 3.58E-07 |
| Bra005175 | 969 | Up | 1.59E-67 |
| Bra021805 | 2490 | Up | 2.56E-06 |
| Bra023454 | 1518 | Up | 7.10E-08 |
| Bra002522 | 420 | Up | 2.50E-51 |
| Bra004082 | 1995 | Up | 1.36E-07 |
| Bra021673 | 648 | Up | 1.68E-79 |
| Bra004614 | 1110 | Up | 0.000129459 |
| Bra028478 | 2313 | Up | 1.08E-11 |
| Bra011993 | 2901 | Up | 2.10E-21 |
| Bra007152 | 1941 | Up | 1.92E-07 |
| Bra035469 | 1179 | Up | 1.44E-10 |
| Bra039340 | 705 | Up | 0.000477544 |
| Bra023786 | 471 | Up | 0.000477488 |
| Bra022034 | 3264 | Up | 7.15E-07 |
| Bra029260 | 579 | Up | 2.58E-05 |
| Bra030549 | 876 | Up | 2.00E-09 |
| Bra026519 | 1155 | Up | 9.54E-05 |
| Bra009007 | 1308 | Up | 1.62E-43 |
| Bra001232 | 1755 | Up | 6.99E-17 |
| Bra015554 | 663 | Up | 9.59E-13 |
| Bra028016 | 1032 | Up | 5.08E-17 |
| Bra004392 | 747 | Up | 2.46E-19 |
| Bra008906 | 663 | Up | 1.89E-05 |
| Bra013069 | 1401 | Up | 3.01E-25 |
| Bra005059 | 1701 | Up | 3.74E-06 |
| Bra025943 | 1251 | Up | 2.49E-12 |
| Bra001020 | 1638 | Up | 7.57E-64 |
| Bra016127 | 1089 | Up | 3.45E-57 |
| Bra032649 | 2283 | Up | 1.52E-09 |
| Bra010359 | 975 | Up | 1.46E-07 |
| Bra023108 | 876 | Up | 7.31E-102 |
| Bra019521 | 1836 | Up | 2.67E-05 |
| Bra022478 | 4830 | Up | 4.35E-11 |
| Bra007519 | 279 | Up | 7.54E-18 |
| Bra015362 | 1338 | Up | 1.04E-06 |
| Bra035194 | 921 | Up | 2.35E-11 |
| Bra028466 | 1071 | Up | 1.13E-08 |
| Bra007982 | 1455 | Up | 3.76E-05 |
| Bra040747 | 615 | Up | 9.40E-44 |
| Bra003323 | 2001 | Up | 0.000188934 |
| Bra023724 | 1194 | Up | 7.89E-20 |
| Bra030573 | 423 | Up | 2.73E-12 |
| Bra007002 | 972 | Up | 5.03E-224 |
| Bra016267 | 543 | Up | 1.43E-05 |
| Bra003270 | 1644 | Up | 1.85E-28 |
| Bra019686 | 2436 | Up | 4.46E-16 |
| Bra030675 | 675 | Up | 1.51E-12 |
| Bra019677 | 765 | Up | 3.08E-08 |
| Bra023130 | 1860 | Up | 5.94E-08 |
| Bra006099 | 942 | Up | 5.15E-12 |
| Bra036226 | 1194 | Up | 5.27E-05 |
| Bra011749 | 987 | Up | 2.69E-31 |
| Bra006922 | 1644 | Up | 2.50E-11 |
| Bra004882 | 2355 | Up | 2.25E-08 |
| Bra010282 | 768 | Up | 0.000265125 |
| Bra024721 | 408 | Up | 1.52E-06 |
| Bra002156 | 1152 | Up | 4.33E-08 |
| Bra022840 | 927 | Up | 1.11E-06 |
| Bra021815 | 1797 | Up | 5.88E-29 |
| Bra038453 | 2094 | Up | 4.00E-34 |
| Bra029928 | 480 | Up | 2.31E-54 |
| Bra006345 | 546 | Up | 0.000194669 |
| Bra003476 | 1458 | Up | 2.90E-39 |
| Bra036805 | 963 | Up | 9.57E-15 |
| Bra009644 | 882 | Up | 5.23E-50 |
| Bra036448 | 1182 | Up | 2.62E-17 |
| Bra026129 | 4710 | Up | 1.57E-06 |
| Bra024568 | 1752 | Up | 2.67E-135 |
| Bra000887 | 570 | Up | 1.32E-10 |
| Bra009045 | 1530 | Up | 7.11E-19 |
| Bra022003 | 1140 | Up | 1.68E-39 |
| Bra009347 | 1224 | Up | 5.74E-51 |
| Bra029381 | 480 | Up | 5.12E-14 |
| Bra038926 | 1899 | Up | 2.71E-15 |
| Bra037091 | 1173 | Up | 1.82E-31 |
| Bra013104 | 1851 | Up | 3.83E-17 |
| Bra016729 | 1020 | Up | 2.90E-243 |
| Bra020203 | 1299 | Up | 8.37E-07 |
| Bra025513 | 1722 | Up | 5.01E-13 |
| Bra017666 | 1587 | Up | 2.20E-06 |
| Bra037098 | 2184 | Up | 1.52E-05 |
| Bra020267 | 4113 | Up | 3.14E-12 |
| Bra017591 | 537 | Up | 5.20E-11 |
| Bra002683 | 2949 | Up | 2.78E-22 |
| Bra038327 | 2715 | Up | 1.06E-16 |
| Bra005347 | 501 | Up | 9.97E-35 |
| Bra013161 | 1170 | Up | 2.03E-20 |
| Bra012347 | 510 | Up | 1.11E-05 |
| Bra013819 | 1851 | Up | 1.07E-15 |
| Bra023072 | 1335 | Up | 3.97E-92 |
| Bra033461 | 327 | Up | 0.000523196 |
| Bra020935 | 1995 | Up | 4.05E-22 |
| Bra027514 | 3339 | Up | 2.57E-09 |
| Bra022965 | 1146 | Up | 2.13E-05 |
| Bra015567 | 1434 | Up | 3.26E-07 |
| Bra014414 | 777 | Up | 5.82E-25 |
| Bra014618 | 1035 | Up | 0.000382673 |
| Bra014079 | 723 | Up | 3.13E-23 |
| Bra009414 | 3468 | Up | 1.00E-09 |
| Bra021340 | 1623 | Up | 1.65E-06 |
| Bra004989 | 831 | Up | 0 |
| Bra007733 | 795 | Up | 6.08E-17 |
| Bra007063 | 723 | Up | 1.14E-05 |
| Bra029025 | 618 | Up | 2.53E-12 |
| Bra001665 | 1332 | Up | 2.46E-29 |
| Bra029108 | 1236 | Up | 1.08E-10 |
| Bra032352 | 2127 | Up | 7.86E-05 |
| Bra016977 | 1248 | Up | 8.82E-07 |
| Bra007479 | 2217 | Up | 7.88E-11 |
| Bra038496 | 2541 | Up | 6.44E-07 |
| Bra002224 | 1008 | Up | 6.42E-17 |
| Bra018543 | 642 | Up | 1.04E-41 |
| Bra003057 | 873 | Up | 5.72E-05 |
| Bra006879 | 1320 | Up | 6.87E-27 |
| Bra037942 | 417 | Up | 1.64E-14 |
| Bra019875 | 891 | Up | 3.08E-61 |
| Bra009188 | 783 | Up | 4.99E-08 |
| Bra026506 | 651 | Up | 4.44E-06 |
| Bra009356 | 1686 | Up | 4.20E-05 |
| Bra028963 | 633 | Up | 1.29E-17 |
| Bra023091 | 888 | Up | 1.17E-05 |
| Bra030519 | 1293 | Up | 3.26E-06 |
| Bra006909 | 1194 | Up | 2.03E-09 |
| Bra022095 | 1965 | Up | 3.07E-05 |
| Bra000809 | 2670 | Up | 1.84E-07 |
| Bra028238 | 753 | Up | 2.18E-10 |
| Bra007921 | 1923 | Up | 0.000287403 |
| Bra003809 | 4143 | Up | 1.35E-16 |
| Bra008489 | 378 | Up | 9.80E-08 |
| Bra027254 | 204 | Up | 1.02E-19 |
| Bra031706 | 2565 | Up | 4.20E-10 |
| Bra021882 | 555 | Up | 0.000210601 |
| Bra018333 | 1374 | Up | 9.25E-07 |
| Bra000013 | 2016 | Up | 1.20E-05 |
| Bra035853 | 1158 | Up | 1.42E-16 |
| Bra024433 | 1401 | Up | 2.13E-09 |
| Bra022212 | 5565 | Up | 0.0001122 |
| Bra011724 | 972 | Up | 7.36E-08 |
| Bra033982 | 603 | Up | 2.67E-174 |
| Bra007327 | 2631 | Up | 2.55E-13 |
| Bra025602 | 966 | Up | 1.36E-13 |
| Bra028142 | 555 | Up | 1.23E-05 |
| Bra039543 | 2835 | Up | 4.20E-09 |
| Bra035883 | 765 | Up | 0.000215437 |
| Bra034896 | 1374 | Up | 0.000215409 |
| Bra026325 | 1146 | Up | 9.43E-80 |
| Bra028214 | 267 | Up | 6.67E-11 |
| Bra037832 | 792 | Up | 2.08E-30 |
| Bra025827 | 1659 | Up | 1.89E-25 |
| Bra027867 | 2766 | Up | 0.000157852 |
| Bra024320 | 846 | Up | 1.97E-07 |
| Bra029899 | 2025 | Up | 1.84E-75 |
| Bra002487 | 537 | Up | 0.000772175 |
| Bra029966 | 1245 | Up | 1.19E-09 |
| Bra034663 | 957 | Up | 5.56E-12 |
| Bra008841 | 2256 | Up | 2.35E-05 |
| Bra038690 | 5847 | Up | 1.74E-20 |
| Bra009355 | 2289 | Up | 3.22E-83 |
| Bra037267 | 2805 | Up | 1.80E-21 |
| Bra002236 | 993 | Up | 5.46E-27 |
| Bra025526 | 537 | Up | 1.72E-05 |
| Bra003750 | 810 | Up | 7.68E-08 |
| Bra002473 | 759 | Up | 4.99E-22 |
| Bra007126 | 1011 | Up | 1.25E-05 |
| Bra008612 | 1071 | Up | 8.18E-29 |
| Bra036582 | 2232 | Up | 2.78E-37 |
| Bra016680 | 1035 | Up | 1.32E-20 |
| Bra027021 | 2328 | Up | 0 |
| Bra018357 | 2220 | Up | 6.96E-11 |
| Bra021037 | 714 | Up | 2.00E-36 |
| Bra031690 | 1584 | Up | 1.28E-18 |
| Bra017656 | 813 | Up | 3.29E-05 |
| Bra018849 | 1773 | Up | 2.34E-16 |
| Bra018987 | 1356 | Up | 5.27E-13 |
| Bra040689 | 1080 | Up | 5.27E-07 |
| Bra007622 | 1422 | Up | 5.27E-07 |
| Bra006029 | 891 | Up | 1.71E-68 |
| Bra024640 | 2235 | Up | 2.81E-07 |
| Bra000574 | 237 | Up | 3.27E-09 |
| Bra019664 | 1023 | Up | 0.000787614 |
| Bra015784 | 1242 | Up | 6.29E-09 |
| Bra010337 | 1164 | Up | 5.37E-07 |
| Bra001942 | 1596 | Up | 6.04E-35 |
| Bra002258 | 837 | Up | 1.86E-12 |
| Bra036256 | 687 | Up | 0.000306568 |
| Bra026724 | 1461 | Up | 0.000306531 |
| Bra015928 | 1182 | Up | 2.54E-12 |
| Bra002918 | 1236 | Up | 7.03E-27 |
| Bra002625 | 1770 | Up | 0.00016431 |
| Bra010508 | 2697 | Up | 1.31E-113 |
| Bra020673 | 1371 | Up | 2.91E-29 |
| Bra035802 | 567 | Up | 1.52E-29 |
| Bra024344 | 1953 | Up | 0.000119306 |
| Bra029375 | 750 | Up | 1.23E-08 |
| Bra008701 | 1080 | Up | 3.71E-10 |
| Bra026650 | 543 | Up | 2.70E-06 |
| Bra033282 | 1449 | Up | 5.12E-16 |
| Bra030460 | 402 | Up | 4.68E-05 |
| Bra030608 | 1068 | Up | 3.47E-09 |
| Bra017350 | 762 | Up | 1.44E-06 |
| Bra021797 | 2241 | Up | 4.09E-28 |
| Bra006586 | 354 | Up | 0.000800624 |
| Bra006616 | 858 | Up | 0.000800534 |
| Bra022569 | 1707 | Up | 0.000583296 |
| Bra002176 | 804 | Up | 1.62E-11 |
| Bra018544 | 372 | Up | 2.17E-07 |
| Bra029828 | 2997 | Up | 1.17E-45 |
| Bra007116 | 1473 | Up | 2.57E-22 |
| Bra003084 | 663 | Up | 0.000167337 |
| Bra024709 | 1527 | Up | 2.68E-15 |
| Bra001911 | 318 | Up | 1.47E-06 |
| Bra034190 | 537 | Up | 8.93E-05 |
| Bra035939 | 432 | Up | 3.59E-09 |
| Bra040580 | 537 | Up | 3.47E-05 |
| Bra031087 | 816 | Up | 2.21E-07 |
| Bra003452 | 2289 | Up | 0.000593352 |
| Bra015734 | 1854 | Up | 5.47E-10 |
| Bra006419 | 849 | Up | 0.000432943 |
| Bra030242 | 2382 | Up | 3.70E-14 |
| Bra029552 | 1167 | Up | 7.45E-15 |
| Bra018437 | 3075 | Up | 4.66E-08 |
| Bra020170 | 741 | Up | 2.58E-05 |
| Bra036113 | 633 | Up | 2.58E-05 |
| Bra025832 | 1479 | Up | 1.31E-203 |
| Bra016463 | 1662 | Up | 1.52E-29 |
| Bra031393 | 1179 | Up | 8.76E-13 |
| Bra036688 | 4338 | Up | 4.82E-33 |
| Bra008820 | 2085 | Up | 2.51E-08 |
| Bra022815 | 648 | Up | 4.92E-63 |
| Bra016354 | 444 | Up | 1.16E-10 |
| Bra021059 | 1689 | Up | 5.64E-35 |
| Bra022229 | 885 | Up | 0.000826913 |
| Bra041018 | 1539 | Up | 9.01E-08 |
| Bra025875 | 1122 | Up | 0.000125528 |
| Bra026740 | 1917 | Up | 9.21E-05 |
| Bra002769 | 1593 | Up | 0.000321186 |
| Bra030023 | 1338 | Up | 2.11E-06 |
| Bra039217 | 819 | Up | 7.78E-34 |
| Bra033630 | 4095 | Up | 8.63E-16 |
| Bra025013 | 2136 | Up | 2.57E-66 |
| Bra029770 | 1317 | Up | 3.44E-11 |
| Bra006809 | 1161 | Up | 4.98E-31 |
| Bra007345 | 4923 | Up | 4.62E-16 |
| Bra004267 | 597 | Up | 2.38E-22 |
| Bra001059 | 1566 | Up | 6.05E-07 |
| Bra013398 | 1716 | Up | 2.93E-06 |
| Bra009384 | 894 | Up | 4.02E-06 |
| Bra029355 | 1443 | Up | 4.30E-10 |
| Bra037390 | 1974 | Up | 1.57E-14 |
| Bra023694 | 1737 | Up | 3.64E-05 |
| Bra036734 | 2058 | Up | 5.17E-13 |
| Bra003322 | 1281 | Up | 2.07E-25 |
| Bra039777 | 1533 | Up | 6.81E-05 |
| Bra011558 | 1668 | Up | 8.45E-21 |
| Bra022936 | 720 | Up | 7.55E-12 |
| Bra031940 | 1050 | Up | 3.61E-08 |
| Bra016555 | 705 | Up | 2.17E-32 |
| Bra000863 | 1113 | Up | 3.04E-45 |
| Bra035240 | 963 | Up | 0.000446115 |
| Bra031391 | 489 | Up | 3.18E-10 |
| Bra027394 | 1518 | Up | 6.14E-07 |
| Bra008453 | 1347 | Up | 1.29E-12 |
| Bra010702 | 1242 | Up | 0.000837906 |
| Bra007771 | 1041 | Up | 4.00E-09 |
| Bra037188 | 582 | Up | 1.15E-19 |
| Bra009435 | 1584 | Up | 3.90E-13 |
| Bra005759 | 942 | Up | 1.12E-13 |
| Bra022663 | 387 | Up | 4.53E-07 |
| Bra007440 | 2538 | Up | 7.18E-13 |
| Bra039495 | 1506 | Up | 6.90E-05 |
| Bra014328 | 804 | Up | 1.91E-16 |
| Bra027543 | 1758 | Up | 1.66E-31 |
| Bra037306 | 903 | Up | 1.16E-09 |
| Bra034541 | 438 | Up | 7.27E-13 |
| Bra014689 | 390 | Up | 9.35E-11 |
| Bra027670 | 1452 | Up | 1.80E-62 |
| Bra031267 | 4395 | Up | 9.40E-21 |
| Bra006181 | 1791 | Up | 1.99E-05 |
| Bra025431 | 546 | Up | 0 |
| Bra034705 | 1182 | Up | 2.50E-20 |
| Bra004540 | 858 | Up | 1.26E-14 |
| Bra002385 | 1269 | Up | 7.19E-16 |
| Bra019144 | 2457 | Up | 2.26E-18 |
| Bra006572 | 762 | Up | 3.68E-86 |
| Bra004630 | 915 | Up | 3.34E-10 |
| Bra010489 | 1362 | Up | 1.08E-08 |
| Bra026465 | 1554 | Up | 1.19E-64 |
| Bra004763 | 861 | Up | 5.72E-06 |
| Bra001207 | 990 | Up | 2.41E-14 |
| Bra023343 | 1035 | Up | 4.65E-07 |
| Bra002537 | 1887 | Up | 2.76E-08 |
| Bra006086 | 390 | Up | 2.24E-09 |
| Bra013955 | 855 | Up | 2.46E-10 |
| Bra009237 | 1011 | Up | 1.77E-14 |
| Bra020809 | 1188 | Up | 6.88E-64 |
| Bra004459 | 384 | Up | 4.14E-55 |
| Bra029712 | 1359 | Up | 1.13E-95 |
| Bra024480 | 360 | Up | 6.44E-07 |
| Bra006857 | 930 | Up | 7.42E-21 |
| Bra001090 | 2370 | Up | 9.10E-19 |
| Bra037199 | 1410 | Up | 7.06E-05 |
| Bra010986 | 1005 | Up | 7.93E-06 |
| Bra015697 | 1023 | Up | 1.53E-16 |
| Bra026131 | 840 | Up | 0.00085749 |
| Bra008975 | 897 | Up | 5.81E-22 |
| Bra035531 | 603 | Up | 9.68E-05 |
| Bra013425 | 2772 | Up | 2.18E-77 |
| Bra018202 | 1323 | Up | 0.000247514 |
| Bra027679 | 1953 | Up | 5.85E-06 |
| Bra035062 | 1635 | Up | 0.000337388 |
| Bra002982 | 615 | Up | 2.56E-07 |
| Bra033415 | 1203 | Up | 1.22E-97 |
| Bra024294 | 3921 | Up | 3.55E-14 |
| Bra004178 | 1014 | Up | 6.66E-10 |
| Bra020544 | 1470 | Up | 0.000632297 |
| Bra030404 | 423 | Up | 2.67E-12 |
| Bra013255 | 1296 | Up | 3.85E-05 |
