# Supplementary material for: Tracing the Transcriptomic Changes in Synthetic Trigenomic allohexaploids of Brassica Using an RNA-Seq Approach
Source: PLoS One. 2013 Jul 11;8(7):e68883. doi: 10.1371/journal.pone.0068883 (PMC3708896; doi:10.1371/journal.pone.0068883)
Supplement: Table S3 — FDR: false discovery rate. We screened genes with expression change no less than two-fold and FDR no more than 0.001 as differentially expressed genes. There were 2274 up-regulated genes and 1370 down-regulated genes compared Brassica hexaploid (C) to B . carinata (B). (DOC) [file pone.0068883.s003.doc]

**A set of 3644 differentially expressed genes between *Brassica* hexaploid and *B. carinata***

FDR: false discovery rate. We screened genes with expression change no less than two-fold and FDR no more than 0.001 as differentially expressed genes. There were 2274 up-regulated genes and 1370 down-regulated genes compared *Brassica* hexaploid (C) to *B. carinata* (B).

| ***GeneID*** | ***Gene length (bp)*** | ***Up-Down-Regulation (C/B)*** | ***FDR*** |
| --- | --- | --- | --- |
| Bra033985 | 312 | Down | 4.51E-15 |
| Bra004262 | 1185 | Down | 4.51E-15 |
| Bra005100 | 630 | Down | 4.50E-15 |
| Bra021182 | 1794 | Down | 4.51E-15 |
| Bra033824 | 447 | Down | 6.99E-09 |
| Bra019190 | 282 | Down | 1.79E-05 |
| Bra008254 | 258 | Down | 0.00014363 |
| Bra039255 | 426 | Down | 1.06E-06 |
| Bra007517 | 267 | Down | 0.0002876 |
| Bra039016 | 354 | Down | 1.79E-05 |
| Bra015125 | 582 | Down | 1.24E-07 |
| Bra021566 | 1527 | Down | 4.51E-15 |
| Bra019230 | 1098 | Down | 4.51E-11 |
| Bra004890 | 957 | Down | 3.40E-09 |
| Bra012702 | 1518 | Down | 7.26E-14 |
| Bra019605 | 630 | Down | 1.79E-05 |
| Bra029211 | 933 | Down | 1.24E-07 |
| Bra033119 | 702 | Down | 1.79E-05 |
| Bra014821 | 1686 | Down | 2.77E-12 |
| Bra028779 | 519 | Down | 0.00056848 |
| Bra031275 | 999 | Down | 1.06E-06 |
| Bra032329 | 621 | Down | 0.00028765 |
| Bra028045 | 1260 | Down | 2.97E-08 |
| Bra007920 | 984 | Down | 8.88E-06 |
| Bra008232 | 1422 | Down | 2.97E-08 |
| Bra031573 | 771 | Down | 0.00014373 |
| Bra014878 | 1269 | Down | 2.53E-07 |
| Bra028099 | 663 | Down | 0.0005683 |
| Bra008387 | 2283 | Down | 1.37E-12 |
| Bra038401 | 1218 | Down | 1.06E-06 |
| Bra012628 | 1338 | Down | 2.53E-07 |
| Bra024348 | 1017 | Down | 1.79E-05 |
| Bra033250 | 747 | Down | 0.00056821 |
| Bra020175 | 1812 | Down | 3.40E-09 |
| Bra031829 | 825 | Down | 0.00028755 |
| Bra036806 | 1014 | Down | 7.21E-05 |
| Bra015255 | 909 | Down | 0.0002875 |
| Bra021314 | 1017 | Down | 0.00014365 |
| Bra028149 | 1116 | Down | 7.20E-05 |
| Bra029236 | 1716 | Down | 2.53E-07 |
| Bra015629 | 963 | Down | 0.00056839 |
| Bra023495 | 1290 | Down | 7.20E-05 |
| Bra018530 | 1326 | Down | 0.0001436 |
| Bra022128 | 1494 | Down | 7.20E-05 |
| Bra025216 | 1413 | Down | 0.00014371 |
| Bra039630 | 1797 | Down | 1.79E-05 |
| Bra032815 | 1599 | Down | 0.00014368 |
| Bra032100 | 2289 | Down | 2.16E-06 |
| Bra021821 | 1530 | Down | 0.0002877 |
| Bra019705 | 1863 | Down | 0.00056858 |
| Bra030161 | 2145 | Down | 0.0004522 |
| Bra020698 | 519 | Down | 0.0004519 |
| Bra032841 | 1371 | Down | 0.00045197 |
| Bra037027 | 1068 | Down | 0.00030995 |
| Bra007228 | 474 | Down | 0.00045212 |
| Bra029212 | 1269 | Down | 1.95E-05 |
| Bra012118 | 795 | Down | 0.00064206 |
| Bra020644 | 477 | Down | 0.00020514 |
| Bra023115 | 867 | Down | 0.00064185 |
| Bra033304 | 738 | Down | 0.00064196 |
| Bra026612 | 1248 | Down | 0.00045205 |
| Bra039763 | 750 | Down | 5.21E-05 |
| Bra018429 | 1596 | Down | 4.80E-07 |
| Bra031937 | 495 | Down | 0 |
| Bra010000 | 1608 | Down | 3.20E-05 |
| Bra040821 | 1509 | Down | 0.00020507 |
| Bra038955 | 1101 | Down | 9.92E-09 |
| Bra015652 | 1539 | Down | 9.77E-12 |
| Bra009973 | 2610 | Down | 0.00064178 |
| Bra033913 | 1224 | Down | 0.00064171 |
| Bra004204 | 2358 | Down | 0.00064166 |
| Bra011332 | 696 | Down | 0.00064156 |
| Bra038998 | 1587 | Down | 0.00045182 |
| Bra038348 | 1233 | Down | 0.0002051 |
| Bra035339 | 1332 | Down | 3.20E-05 |
| Bra037493 | 942 | Down | 1.60E-07 |
| Bra004089 | 444 | Down | 0.00064179 |
| Bra025061 | 2253 | Down | 0.00064203 |
| Bra014767 | 288 | Down | 0.00064214 |
| Bra007951 | 354 | Down | 0.00031006 |
| Bra031724 | 1548 | Down | 3.22E-09 |
| Bra016178 | 477 | Down | 0.00020503 |
| Bra009837 | 513 | Down | 0.00064282 |
| Bra020595 | 861 | Down | 0.0003099 |
| Bra015035 | 3129 | Down | 8.37E-05 |
| Bra005755 | 1074 | Down | 0.00031011 |
| Bra011889 | 318 | Down | 0.00045168 |
| Bra017679 | 2190 | Down | 0.00045176 |
| Bra021497 | 558 | Down | 0.00013224 |
| Bra037350 | 1053 | Down | 0.00020518 |
| Bra012622 | 396 | Down | 0.0006536 |
| Bra007149 | 288 | Down | 3.56E-12 |
| Bra038442 | 870 | Down | 1.17E-05 |
| Bra040676 | 1284 | Down | 0.00045165 |
| Bra022673 | 1575 | Down | 0.00045157 |
| Bra009406 | 1923 | Down | 0.00066576 |
| Bra033554 | 3333 | Down | 0.00045154 |
| Bra008127 | 987 | Down | 0.00045147 |
| Bra038325 | 966 | Down | 0.00066565 |
| Bra025797 | 1017 | Down | 0.00031 |
| Bra027814 | 501 | Down | 0.00030984 |
| Bra005010 | 1290 | Down | 0.00013229 |
| Bra015973 | 1422 | Down | 0.00073499 |
| Bra016893 | 1809 | Down | 0.00073522 |
| Bra004432 | 1833 | Down | 0.0004518 |
| Bra026592 | 2127 | Down | 0.0007351 |
| Bra009785 | 1518 | Down | 3.99E-11 |
| Bra006168 | 1359 | Down | 3.20E-05 |
| Bra033731 | 1137 | Down | 0.00013231 |
| Bra028062 | 522 | Down | 0.0003098 |
| Bra039017 | 294 | Down | 0.00013226 |
| Bra013495 | 291 | Down | 0.00082074 |
| Bra011130 | 1350 | Down | 0 |
| Bra038101 | 552 | Down | 0.00030978 |
| Bra015163 | 1608 | Down | 0.00030972 |
| Bra018539 | 765 | Down | 5.20E-05 |
| Bra017664 | 462 | Down | 3.20E-05 |
| Bra012882 | 798 | Down | 0.00098444 |
| Bra017438 | 495 | Down | 0.00098459 |
| Bra028865 | 1083 | Down | 2.44E-06 |
| Bra033923 | 951 | Down | 0.00030977 |
| Bra001515 | 1812 | Down | 0.00046061 |
| Bra033682 | 3060 | Down | 9.91E-09 |
| Bra008357 | 345 | Down | 1.17E-05 |
| Bra036249 | 1887 | Down | 0.00046907 |
| Bra023100 | 420 | Down | 1.95E-05 |
| Bra024521 | 489 | Down | 0.00031047 |
| Bra005302 | 813 | Down | 0.00031052 |
| Bra012147 | 1101 | Down | 0.00013219 |
| Bra001121 | 426 | Down | 4.14E-06 |
| Bra032578 | 732 | Down | 0.00013221 |
| Bra008163 | 1551 | Down | 1.17E-05 |
| Bra005922 | 1320 | Down | 4.14E-06 |
| Bra031852 | 1077 | Down | 9.27E-08 |
| Bra004489 | 1203 | Down | 0 |
| Bra027758 | 351 | Down | 0.00057126 |
| Bra019080 | 456 | Down | 0.00013215 |
| Bra024992 | 816 | Down | 0.00013217 |
| Bra018690 | 750 | Down | 0.00031583 |
| Bra030096 | 735 | Down | 5.20E-05 |
| Bra007978 | 2430 | Down | 0 |
| Bra020695 | 495 | Down | 0.00013213 |
| Bra003483 | 924 | Down | 6.97E-06 |
| Bra031919 | 579 | Down | 6.97E-06 |
| Bra025785 | 162 | Down | 0.00013211 |
| Bra021880 | 426 | Down | 5.96E-10 |
| Bra004083 | 1866 | Down | 6.56E-13 |
| Bra015829 | 3417 | Down | 0.00067579 |
| Bra020456 | 180 | Down | 0.00067568 |
| Bra026978 | 2673 | Down | 0.00067558 |
| Bra038344 | 426 | Down | 0.0006759 |
| Bra034257 | 2427 | Down | 0.00067601 |
| Bra003232 | 474 | Down | 4.14E-06 |
| Bra007957 | 576 | Down | 8.37E-05 |
| Bra012433 | 933 | Down | 0 |
| Bra032801 | 912 | Down | 0.00020612 |
| Bra010479 | 1212 | Down | 8.37E-05 |
| Bra010078 | 369 | Down | 3.05E-08 |
| Bra012555 | 864 | Down | 0.00086608 |
| Bra024161 | 1167 | Down | 0.00086635 |
| Bra001100 | 1101 | Down | 2.77E-07 |
| Bra033970 | 582 | Down | 0.00086622 |
| Bra012233 | 1629 | Down | 0.0002069 |
| Bra029388 | 1431 | Down | 0.00020686 |
| Bra035164 | 1275 | Down | 1.94E-05 |
| Bra012982 | 1617 | Down | 0.00035001 |
| Bra000225 | 1206 | Down | 0.00035007 |
| Bra018497 | 579 | Down | 0.00035013 |
| Bra007644 | 840 | Down | 9.27E-08 |
| Bra039970 | 1521 | Down | 1.94E-05 |
| Bra004530 | 1755 | Down | 3.20E-05 |
| Bra003262 | 2127 | Down | 0.00013246 |
| Bra010946 | 630 | Down | 0.00038501 |
| Bra022135 | 1089 | Down | 4.14E-06 |
| Bra010913 | 267 | Down | 1.94E-05 |
| Bra016891 | 1092 | Down | 1.74E-08 |
| Bra015402 | 966 | Down | 8.38E-05 |
| Bra029089 | 156 | Down | 8.38E-05 |
| Bra008308 | 576 | Down | 3.20E-05 |
| Bra024180 | 627 | Down | 8.38E-05 |
| Bra017431 | 1434 | Down | 2.44E-06 |
| Bra013827 | 1473 | Down | 0.00013344 |
| Bra032458 | 969 | Down | 5.20E-05 |
| Bra017790 | 960 | Down | 3.22E-09 |
| Bra013088 | 222 | Down | 0.00013454 |
| Bra011158 | 327 | Down | 0.00022965 |
| Bra024057 | 840 | Down | 3.20E-05 |
| Bra024939 | 1086 | Down | 4.14E-06 |
| Bra016220 | 1779 | Down | 0.00056055 |
| Bra021800 | 1701 | Down | 0.00056027 |
| Bra033902 | 462 | Down | 0.00056046 |
| Bra004087 | 2622 | Down | 0.00056036 |
| Bra033581 | 315 | Down | 0.00056018 |
| Bra020551 | 387 | Down | 5.33E-08 |
| Bra019209 | 1251 | Down | 0.00013648 |
| Bra016142 | 2331 | Down | 0.00013645 |
| Bra001650 | 462 | Down | 0.0002501 |
| Bra003411 | 804 | Down | 1.94E-05 |
| Bra012969 | 192 | Down | 0.00025015 |
| Bra015981 | 1149 | Down | 5.65E-09 |
| Bra017816 | 486 | Down | 5.22E-05 |
| Bra035787 | 1215 | Down | 0.00075558 |
| Bra020661 | 4281 | Down | 1.05E-09 |
| Bra009569 | 1092 | Down | 4.14E-06 |
| Bra027480 | 987 | Down | 1.94E-05 |
| Bra011070 | 1044 | Down | 5.23E-05 |
| Bra018637 | 2085 | Down | 5.23E-05 |
| Bra025351 | 1362 | Down | 1.17E-05 |
| Bra012446 | 669 | Down | 0.00014632 |
| Bra026552 | 525 | Down | 1.05E-09 |
| Bra008471 | 1692 | Down | 2.77E-07 |
| Bra012717 | 378 | Down | 6.97E-06 |
| Bra005557 | 309 | Down | 0.00034987 |
| Bra008286 | 1554 | Down | 0 |
| Bra033671 | 1089 | Down | 6.97E-06 |
| Bra002046 | 1182 | Down | 8.83E-05 |
| Bra033172 | 1863 | Down | 5.28E-05 |
| Bra031860 | 702 | Down | 5.29E-05 |
| Bra015842 | 1575 | Down | 8.31E-07 |
| Bra016030 | 1131 | Down | 1.95E-05 |
| Bra020685 | 588 | Down | 1.95E-05 |
| Bra012594 | 2283 | Down | 0.00017879 |
| Bra034009 | 1569 | Down | 9.21E-05 |
| Bra036407 | 765 | Down | 1.95E-05 |
| Bra035407 | 2784 | Down | 0 |
| Bra037414 | 936 | Down | 2.07E-12 |
| Bra015742 | 741 | Down | 0 |
| Bra015018 | 1203 | Down | 1.17E-05 |
| Bra017797 | 639 | Down | 3.24E-05 |
| Bra007660 | 1269 | Down | 2.77E-07 |
| Bra034652 | 741 | Down | 3.24E-05 |
| Bra014339 | 456 | Down | 5.47E-05 |
| Bra030495 | 366 | Down | 3.43E-10 |
| Bra027756 | 2496 | Down | 3.40E-12 |
| Bra037433 | 534 | Down | 0.00021461 |
| Bra003687 | 918 | Down | 0.00065316 |
| Bra024929 | 540 | Down | 3.28E-05 |
| Bra024382 | 882 | Down | 4.80E-07 |
| Bra028167 | 804 | Down | 1.18E-05 |
| Bra031838 | 1323 | Down | 0.00011044 |
| Bra012066 | 603 | Down | 0.00011047 |
| Bra000378 | 678 | Down | 1.97E-05 |
| Bra027796 | 1821 | Down | 0.00027743 |
| Bra015220 | 1737 | Down | 0 |
| Bra012567 | 897 | Down | 3.34E-05 |
| Bra037119 | 2331 | Down | 1.05E-09 |
| Bra006851 | 1155 | Down | 0 |
| Bra038324 | 981 | Down | 6.04E-05 |
| Bra007096 | 780 | Down | 0.00097496 |
| Bra018856 | 1554 | Down | 0.00097481 |
| Bra038481 | 249 | Down | 0.00097511 |
| Bra033904 | 1773 | Down | 3.05E-08 |
| Bra016828 | 1533 | Down | 0.00012987 |
| Bra013060 | 813 | Down | 0.0001299 |
| Bra026992 | 420 | Down | 0 |
| Bra039103 | 1056 | Down | 3.46E-05 |
| Bra021082 | 1641 | Down | 1.18E-05 |
| Bra020690 | 504 | Down | 1.74E-08 |
| Bra038652 | 1074 | Down | 7.01E-06 |
| Bra026354 | 315 | Down | 6.68E-05 |
| Bra013264 | 894 | Down | 0.00038336 |
| Bra010175 | 282 | Down | 0.0003833 |
| Bra012745 | 1950 | Down | 7.02E-06 |
| Bra005677 | 831 | Down | 1.10E-11 |
| Bra001626 | 750 | Down | 5.33E-08 |
| Bra021970 | 2412 | Down | 4.79E-07 |
| Bra018532 | 384 | Down | 0 |
| Bra021282 | 2454 | Down | 0 |
| Bra007929 | 441 | Down | 3.41E-12 |
| Bra028794 | 972 | Down | 0.0001639 |
| Bra021108 | 1431 | Down | 0.00016387 |
| Bra036482 | 1305 | Down | 1.78E-12 |
| Bra033095 | 534 | Down | 1.44E-06 |
| Bra000436 | 1215 | Down | 1.50E-11 |
| Bra004836 | 1518 | Down | 0 |
| Bra014758 | 504 | Down | 2.41E-11 |
| Bra032823 | 987 | Down | 7.11E-06 |
| Bra025684 | 546 | Down | 0.00056234 |
| Bra015216 | 321 | Down | 4.01E-05 |
| Bra031309 | 981 | Down | 4.18E-06 |
| Bra017756 | 915 | Down | 4.80E-07 |
| Bra015904 | 540 | Down | 0.00022234 |
| Bra027851 | 2481 | Down | 9.75E-13 |
| Bra019009 | 453 | Down | 9.65E-05 |
| Bra001973 | 2103 | Down | 4.22E-06 |
| Bra014897 | 1242 | Down | 4.61E-05 |
| Bra008296 | 1041 | Down | 4.61E-05 |
| Bra012397 | 345 | Down | 3.56E-12 |
| Bra018103 | 1989 | Down | 0.00085591 |
| Bra040811 | 2409 | Down | 0.00085604 |
| Bra014952 | 2535 | Down | 9.77E-12 |
| Bra031170 | 819 | Down | 0.00012823 |
| Bra000469 | 4215 | Down | 0.0001282 |
| Bra024009 | 528 | Down | 4.81E-07 |
| Bra006695 | 969 | Down | 5.64E-05 |
| Bra038858 | 1365 | Down | 2.72E-05 |
| Bra027144 | 606 | Down | 7.77E-06 |
| Bra025408 | 3165 | Down | 1.50E-11 |
| Bra004000 | 1653 | Down | 1.61E-07 |
| Bra012294 | 612 | Down | 3.05E-08 |
| Bra000199 | 486 | Down | 7.34E-05 |
| Bra011900 | 3294 | Down | 0.0004799 |
| Bra033989 | 1068 | Down | 1.48E-06 |
| Bra019835 | 1176 | Down | 7.35E-05 |
| Bra015102 | 1035 | Down | 1.48E-06 |
| Bra021286 | 639 | Down | 3.28E-05 |
| Bra001609 | 1122 | Down | 3.28E-05 |
| Bra015094 | 1416 | Down | 0 |
| Bra007446 | 771 | Down | 0 |
| Bra018487 | 387 | Down | 3.56E-12 |
| Bra024145 | 432 | Down | 2.21E-13 |
| Bra027478 | 1173 | Down | 1.61E-07 |
| Bra022707 | 399 | Down | 0 |
| Bra036270 | 1689 | Down | 9.31E-06 |
| Bra005488 | 1389 | Down | 0 |
| Bra014936 | 1842 | Down | 0 |
| Bra008446 | 2973 | Down | 6.55E-13 |
| Bra012199 | 234 | Down | 4.92E-07 |
| Bra009339 | 1020 | Down | 4.92E-07 |
| Bra030421 | 1254 | Down | 0.00026833 |
| Bra040650 | 603 | Down | 3.05E-08 |
| Bra014969 | 3999 | Down | 1.50E-11 |
| Bra029874 | 3141 | Down | 0.00074138 |
| Bra005528 | 2205 | Down | 0 |
| Bra009948 | 2766 | Down | 1.10E-05 |
| Bra038807 | 1794 | Down | 2.39E-05 |
| Bra003348 | 2412 | Down | 5.75E-05 |
| Bra004674 | 1110 | Down | 5.75E-05 |
| Bra040652 | 384 | Down | 5.96E-10 |
| Bra024553 | 534 | Down | 1.80E-06 |
| Bra039142 | 957 | Down | 3.23E-05 |
| Bra024216 | 270 | Down | 3.23E-05 |
| Bra009947 | 249 | Down | 0 |
| Bra041111 | 777 | Down | 2.75E-12 |
| Bra024862 | 2181 | Down | 5.96E-10 |
| Bra005161 | 942 | Down | 4.85E-12 |
| Bra004983 | 2700 | Down | 2.42E-11 |
| Bra017765 | 807 | Down | 5.43E-07 |
| Bra003986 | 738 | Down | 8.28E-05 |
| Bra030873 | 594 | Down | 1.03E-06 |
| Bra039354 | 978 | Down | 1.64E-12 |
| Bra036803 | 1674 | Down | 3.98E-11 |
| Bra015650 | 963 | Down | 0.00022493 |
| Bra023455 | 303 | Down | 6.55E-13 |
| Bra004712 | 351 | Down | 1.81E-05 |
| Bra028814 | 195 | Down | 6.40E-13 |
| Bra009836 | 2799 | Down | 1.84E-09 |
| Bra020142 | 1248 | Down | 0.0006375 |
| Bra033113 | 2058 | Down | 1.78E-12 |
| Bra001141 | 504 | Down | 0.0006374 |
| Bra011971 | 612 | Down | 0 |
| Bra021155 | 1116 | Down | 0.0001239 |
| Bra004133 | 1125 | Down | 0 |
| Bra007238 | 405 | Down | 3.40E-12 |
| Bra040649 | 3090 | Down | 0 |
| Bra022412 | 1380 | Down | 1.04E-08 |
| Bra014905 | 837 | Down | 2.11E-07 |
| Bra027444 | 687 | Down | 3.57E-12 |
| Bra037491 | 2424 | Down | 5.90E-09 |
| Bra005846 | 1206 | Down | 6.03E-10 |
| Bra024215 | 798 | Down | 2.42E-11 |
| Bra024833 | 1542 | Down | 5.19E-13 |
| Bra006106 | 504 | Down | 0.00099624 |
| Bra039435 | 1326 | Down | 0.00099608 |
| Bra036993 | 561 | Down | 0.00099639 |
| Bra036281 | 1557 | Down | 3.35E-09 |
| Bra023692 | 177 | Down | 3.72E-05 |
| Bra021856 | 771 | Down | 0 |
| Bra039041 | 363 | Down | 0 |
| Bra021384 | 834 | Down | 2.04E-05 |
| Bra032273 | 945 | Down | 8.61E-13 |
| Bra038262 | 273 | Down | 0.00054348 |
| Bra019290 | 1101 | Down | 5.47E-13 |
| Bra018218 | 789 | Down | 3.59E-09 |
| Bra017948 | 1497 | Down | 2.99E-07 |
| Bra016140 | 2442 | Down | 5.61E-05 |
| Bra002410 | 861 | Down | 3.83E-09 |
| Bra012205 | 2607 | Down | 1.36E-08 |
| Bra026092 | 363 | Down | 0 |
| Bra035336 | 1194 | Down | 3.41E-12 |
| Bra015033 | 3369 | Down | 8.94E-13 |
| Bra000930 | 1578 | Down | 1.66E-05 |
| Bra038781 | 243 | Down | 1.66E-05 |
| Bra023573 | 1071 | Down | 7.83E-12 |
| Bra036821 | 1923 | Down | 2.14E-07 |
| Bra031202 | 327 | Down | 6.80E-12 |
| Bra029253 | 504 | Down | 0.00084789 |
| Bra016585 | 996 | Down | 6.55E-13 |
| Bra003946 | 1467 | Down | 7.16E-11 |
| Bra016900 | 765 | Down | 4.89E-12 |
| Bra024094 | 273 | Down | 0.00084776 |
| Bra027715 | 1218 | Down | 8.84E-09 |
| Bra008061 | 1785 | Down | 8.84E-09 |
| Bra025135 | 939 | Down | 7.48E-10 |
| Bra020643 | 2484 | Down | 7.17E-12 |
| Bra005176 | 792 | Down | 0 |
| Bra011349 | 1098 | Down | 0 |
| Bra014264 | 1359 | Down | 0.00024804 |
| Bra014708 | 2364 | Down | 1.36E-05 |
| Bra000760 | 1227 | Down | 1.36E-05 |
| Bra025239 | 1725 | Down | 4.13E-07 |
| Bra036488 | 2502 | Down | 0 |
| Bra032939 | 1005 | Down | 1.93E-11 |
| Bra015252 | 234 | Down | 7.20E-05 |
| Bra013833 | 1221 | Down | 1.19E-12 |
| Bra032889 | 1020 | Down | 0 |
| Bra019148 | 924 | Down | 3.87E-05 |
| Bra000679 | 1347 | Down | 3.87E-05 |
| Bra009815 | 570 | Down | 0.00071551 |
| Bra020161 | 939 | Down | 0.00038626 |
| Bra005137 | 1506 | Down | 0 |
| Bra023330 | 2955 | Down | 0 |
| Bra003779 | 669 | Down | 0.00020738 |
| Bra019666 | 1308 | Down | 2.29E-11 |
| Bra015394 | 1473 | Down | 0.00020735 |
| Bra001446 | 3084 | Down | 3.25E-06 |
| Bra034327 | 1794 | Down | 2.78E-08 |
| Bra026623 | 1443 | Down | 1.66E-12 |
| Bra028906 | 867 | Down | 2.54E-11 |
| Bra025112 | 309 | Down | 1.74E-06 |
| Bra020320 | 828 | Down | 1.75E-06 |
| Bra026569 | 708 | Down | 9.75E-13 |
| Bra029318 | 2379 | Down | 0.00011217 |
| Bra009043 | 777 | Down | 1.49E-08 |
| Bra017801 | 1137 | Down | 6.01E-05 |
| Bra029936 | 2319 | Down | 4.32E-09 |
| Bra029555 | 1257 | Down | 6.01E-05 |
| Bra031770 | 618 | Down | 6.02E-05 |
| Bra031920 | 1638 | Down | 0 |
| Bra004380 | 1794 | Down | 0 |
| Bra030931 | 729 | Down | 2.67E-06 |
| Bra000256 | 1089 | Down | 2.67E-06 |
| Bra033556 | 1302 | Down | 3.37E-09 |
| Bra009841 | 1104 | Down | 0 |
| Bra028746 | 2604 | Down | 4.02E-07 |
| Bra039521 | 456 | Down | 0.00060069 |
| Bra004901 | 456 | Down | 0 |
| Bra028443 | 279 | Down | 0.00032297 |
| Bra012309 | 477 | Down | 0.00032303 |
| Bra024915 | 1686 | Down | 6.20E-08 |
| Bra035937 | 912 | Down | 6.20E-08 |
| Bra038604 | 978 | Down | 0.00017339 |
| Bra028860 | 4332 | Down | 9.64E-12 |
| Bra036499 | 798 | Down | 0.00017342 |
| Bra025007 | 825 | Down | 8.60E-13 |
| Bra013534 | 2322 | Down | 2.68E-05 |
| Bra041082 | 1584 | Down | 2.68E-05 |
| Bra007384 | 816 | Down | 1.44E-05 |
| Bra013591 | 2121 | Down | 3.73E-11 |
| Bra014343 | 696 | Down | 3.52E-12 |
| Bra026618 | 789 | Down | 6.22E-07 |
| Bra014948 | 2355 | Down | 6.23E-07 |
| Bra012691 | 933 | Down | 0 |
| Bra003102 | 2469 | Down | 0 |
| Bra040471 | 1227 | Down | 5.07E-08 |
| Bra036710 | 432 | Down | 0 |
| Bra014697 | 462 | Down | 0 |
| Bra031094 | 360 | Down | 0 |
| Bra031093 | 432 | Down | 6.86E-13 |
| Bra026756 | 1230 | Down | 3.43E-06 |
| Bra039501 | 3180 | Down | 4.16E-05 |
| Bra006194 | 1722 | Down | 0.00092975 |
| Bra005702 | 630 | Down | 0 |
| Bra021670 | 1059 | Down | 0.0002696 |
| Bra028032 | 1062 | Down | 0.00026955 |
| Bra012342 | 1530 | Down | 0.00014409 |
| Bra039546 | 1782 | Down | 5.33E-12 |
| Bra026377 | 912 | Down | 1.82E-08 |
| Bra017758 | 1644 | Down | 1.21E-07 |
| Bra034486 | 342 | Down | 1.21E-07 |
| Bra040689 | 1080 | Down | 0 |
| Bra018008 | 312 | Down | 2.25E-07 |
| Bra020597 | 2217 | Down | 4.22E-07 |
| Bra019095 | 2265 | Down | 4.22E-07 |
| Bra022978 | 636 | Down | 0 |
| Bra017873 | 1029 | Down | 5.31E-06 |
| Bra025368 | 492 | Down | 9.92E-06 |
| Bra000627 | 210 | Down | 0.00012016 |
| Bra015996 | 2154 | Down | 0.00012018 |
| Bra004928 | 642 | Down | 0 |
| Bra019143 | 546 | Down | 0 |
| Bra035136 | 2325 | Down | 9.97E-08 |
| Bra026615 | 234 | Down | 0 |
| Bra037500 | 942 | Down | 1.86E-07 |
| Bra024885 | 1122 | Down | 0.00041519 |
| Bra036210 | 768 | Down | 0.00041526 |
| Bra003350 | 987 | Down | 0.00041512 |
| Bra015373 | 717 | Down | 0.00077053 |
| Bra025313 | 1275 | Down | 0.00077077 |
| Bra031943 | 2061 | Down | 0.00077065 |
| Bra021947 | 1284 | Down | 2.34E-06 |
| Bra009745 | 4800 | Down | 6.54E-09 |
| Bra012243 | 1641 | Down | 4.38E-06 |
| Bra037445 | 1713 | Down | 0 |
| Bra025265 | 774 | Down | 2.87E-09 |
| Bra008589 | 789 | Down | 5.32E-05 |
| Bra018436 | 534 | Down | 5.40E-09 |
| Bra007142 | 756 | Down | 0.00018474 |
| Bra001239 | 5724 | Down | 0.0001848 |
| Bra014783 | 1464 | Down | 0.00018477 |
| Bra026839 | 360 | Down | 3.61E-08 |
| Bra039030 | 2868 | Down | 2.36E-09 |
| Bra039762 | 1467 | Down | 0 |
| Bra014975 | 876 | Down | 5.48E-13 |
| Bra012460 | 1236 | Down | 1.26E-05 |
| Bra018271 | 723 | Down | 6.94E-12 |
| Bra009752 | 1038 | Down | 0.00063767 |
| Bra008210 | 309 | Down | 2.35E-05 |
| Bra015165 | 3051 | Down | 0 |
| Bra009966 | 981 | Down | 1.60E-06 |
| Bra010475 | 477 | Down | 5.54E-13 |
| Bra034855 | 432 | Down | 2.98E-06 |
| Bra025458 | 1377 | Down | 8.19E-05 |
| Bra002595 | 570 | Down | 8.19E-05 |
| Bra007662 | 1383 | Down | 5.59E-06 |
| Bra010943 | 612 | Down | 8.49E-10 |
| Bra012831 | 1977 | Down | 0.00015179 |
| Bra025072 | 2340 | Down | 1.04E-05 |
| Bra034038 | 2379 | Down | 0 |
| Bra025043 | 381 | Down | 0 |
| Bra018681 | 435 | Down | 0.00028337 |
| Bra015980 | 738 | Down | 1.94E-05 |
| Bra025304 | 576 | Down | 0 |
| Bra004568 | 477 | Down | 2.01E-08 |
| Bra024750 | 1245 | Down | 0 |
| Bra014127 | 468 | Down | 0.00052512 |
| Bra024892 | 807 | Down | 6.73E-05 |
| Bra021111 | 966 | Down | 8.56E-06 |
| Bra040117 | 4503 | Down | 0.00096859 |
| Bra037473 | 630 | Down | 0.0009683 |
| Bra013214 | 1431 | Down | 2.14E-12 |
| Bra037458 | 1521 | Down | 0.00096844 |
| Bra018896 | 1086 | Down | 2.47E-12 |
| Bra040570 | 345 | Down | 0.00012499 |
| Bra000347 | 1251 | Down | 0 |
| Bra012439 | 669 | Down | 2.48E-07 |
| Bra012832 | 480 | Down | 3.80E-09 |
| Bra002599 | 1476 | Down | 0 |
| Bra023052 | 453 | Down | 1.61E-12 |
| Bra017810 | 702 | Down | 0.00023225 |
| Bra020583 | 999 | Down | 0.00023229 |
| Bra009643 | 1086 | Down | 2.97E-05 |
| Bra030281 | 1473 | Down | 2.97E-05 |
| Bra033942 | 1233 | Down | 1.09E-07 |
| Bra039394 | 792 | Down | 0 |
| Bra036475 | 195 | Down | 5.52E-05 |
| Bra037009 | 1008 | Down | 5.52E-05 |
| Bra012667 | 615 | Down | 0 |
| Bra009994 | 1833 | Down | 2.28E-12 |
| Bra003299 | 1110 | Down | 1.31E-05 |
| Bra000231 | 978 | Down | 3.79E-07 |
| Bra025106 | 3315 | Down | 0.00010269 |
| Bra030949 | 1041 | Down | 0.00010271 |
| Bra038059 | 375 | Down | 0.00010267 |
| Bra017170 | 339 | Down | 7.45E-11 |
| Bra019094 | 1392 | Down | 0 |
| Bra031956 | 828 | Down | 1.79E-11 |
| Bra016404 | 606 | Down | 0.0007958 |
| Bra012085 | 1821 | Down | 7.15E-07 |
| Bra012860 | 960 | Down | 7.15E-07 |
| Bra020873 | 3915 | Down | 0.00079567 |
| Bra024895 | 729 | Down | 0 |
| Bra035139 | 645 | Down | 9.00E-09 |
| Bra005960 | 789 | Down | 4.51E-05 |
| Bra006136 | 1791 | Down | 7.28E-12 |
| Bra008264 | 1197 | Down | 4.94E-12 |
| Bra010435 | 1779 | Down | 1.36E-07 |
| Bra004898 | 2211 | Down | 0 |
| Bra011357 | 1035 | Down | 3.17E-08 |
| Bra024917 | 1119 | Down | 1.71E-09 |
| Bra008245 | 1737 | Down | 3.97E-10 |
| Bra025309 | 747 | Down | 2.23E-11 |
| Bra015667 | 2508 | Down | 1.10E-06 |
| Bra006337 | 1131 | Down | 8.32E-12 |
| Bra035980 | 585 | Down | 1.46E-12 |
| Bra001256 | 1146 | Down | 3.20E-09 |
| Bra037409 | 342 | Down | 0 |
| Bra040469 | 525 | Down | 9.60E-13 |
| Bra010857 | 1173 | Down | 0.00015508 |
| Bra017723 | 639 | Down | 2.60E-08 |
| Bra009874 | 1161 | Down | 0.00015511 |
| Bra002072 | 2307 | Down | 4.86E-08 |
| Bra010171 | 474 | Down | 4.86E-08 |
| Bra012658 | 609 | Down | 0 |
| Bra011638 | 8130 | Down | 3.83E-06 |
| Bra003630 | 2304 | Down | 0 |
| Bra035015 | 666 | Down | 6.86E-05 |
| Bra013263 | 816 | Down | 6.86E-05 |
| Bra025872 | 1302 | Down | 0.00028815 |
| Bra036055 | 1239 | Down | 0.0002881 |
| Bra029342 | 1359 | Down | 0.0002882 |
| Bra000178 | 1011 | Down | 3.33E-12 |
| Bra033756 | 2478 | Down | 0 |
| Bra015590 | 651 | Down | 0.00012677 |
| Bra025409 | 1398 | Down | 6.75E-13 |
| Bra015290 | 2337 | Down | 3.14E-07 |
| Bra008285 | 1668 | Down | 3.21E-08 |
| Bra028521 | 1998 | Down | 0 |
| Bra012925 | 2982 | Down | 0 |
| Bra013409 | 930 | Down | 0.00023426 |
| Bra018836 | 2871 | Down | 8.80E-12 |
| Bra012074 | 3429 | Down | 0 |
| Bra025162 | 2313 | Down | 0 |
| Bra040277 | 1491 | Down | 0.00097511 |
| Bra014490 | 795 | Down | 1.11E-06 |
| Bra025015 | 5103 | Down | 0 |
| Bra033491 | 318 | Down | 3.89E-12 |
| Bra008218 | 1341 | Down | 4.72E-06 |
| Bra023549 | 1032 | Down | 0 |
| Bra034326 | 693 | Down | 4.53E-05 |
| Bra007170 | 783 | Down | 0.00043309 |
| Bra025192 | 1503 | Down | 2.08E-07 |
| Bra030098 | 2997 | Down | 2.05E-06 |
| Bra025243 | 1101 | Down | 3.86E-07 |
| Bra030067 | 618 | Down | 8.40E-05 |
| Bra037718 | 522 | Down | 8.39E-05 |
| Bra035628 | 189 | Down | 0 |
| Bra023981 | 516 | Down | 1.68E-06 |
| Bra022231 | 903 | Down | 0.00079491 |
| Bra022496 | 1611 | Down | 0.00079478 |
| Bra019083 | 306 | Down | 0.00079503 |
| Bra012099 | 2316 | Down | 1.39E-08 |
| Bra024165 | 972 | Down | 2.65E-10 |
| Bra001673 | 1191 | Down | 7.08E-06 |
| Bra000675 | 933 | Down | 0.00035092 |
| Bra030515 | 633 | Down | 3.11E-06 |
| Bra023384 | 2592 | Down | 2.27E-12 |
| Bra041140 | 1155 | Down | 0.00015441 |
| Bra020500 | 1101 | Down | 0 |
| Bra037712 | 2064 | Down | 6.82E-05 |
| Bra023993 | 1998 | Down | 1.89E-11 |
| Bra037016 | 4521 | Down | 2.99E-05 |
| Bra007411 | 345 | Down | 2.99E-05 |
| Bra010760 | 318 | Down | 3.39E-13 |
| Bra015598 | 1260 | Down | 0 |
| Bra022678 | 195 | Down | 0 |
| Bra031496 | 1131 | Down | 1.37E-12 |
| Bra000990 | 2544 | Down | 2.06E-07 |
| Bra040700 | 2238 | Down | 0 |
| Bra034365 | 525 | Down | 5.52E-05 |
| Bra034447 | 459 | Down | 1.52E-12 |
| Bra024125 | 3306 | Down | 1.07E-05 |
| Bra018596 | 1131 | Down | 6.69E-12 |
| Bra005604 | 2010 | Down | 1.67E-07 |
| Bra019389 | 3294 | Down | 8.61E-06 |
| Bra025194 | 1503 | Down | 0 |
| Bra024802 | 1785 | Down | 7.13E-07 |
| Bra025080 | 948 | Down | 8.93E-09 |
| Bra006976 | 1374 | Down | 4.48E-12 |
| Bra001870 | 1674 | Down | 2.49E-07 |
| Bra025848 | 2166 | Down | 1.33E-06 |
| Bra015786 | 1329 | Down | 3.05E-06 |
| Bra016548 | 1113 | Down | 0 |
| Bra021809 | 405 | Down | 3.62E-05 |
| Bra038226 | 402 | Down | 8.23E-05 |
| Bra003862 | 1785 | Down | 1.67E-08 |
| Bra013580 | 393 | Down | 1.67E-08 |
| Bra034506 | 1260 | Down | 0.00018685 |
| Bra003158 | 249 | Down | 1.10E-12 |
| Bra017669 | 786 | Down | 0 |
| Bra005448 | 1584 | Down | 0.00095512 |
| Bra021023 | 402 | Down | 1.74E-12 |
| Bra039563 | 876 | Down | 0 |
| Bra009864 | 990 | Down | 6.65E-05 |
| Bra011060 | 1086 | Down | 0 |
| Bra020251 | 1239 | Down | 0 |
| Bra039810 | 417 | Down | 1.98E-06 |
| Bra031260 | 1269 | Down | 0 |
| Bra014802 | 2352 | Down | 3.00E-07 |
| Bra039603 | 1095 | Down | 3.00E-07 |
| Bra001575 | 2904 | Down | 0.00077276 |
| Bra016833 | 216 | Down | 2.35E-05 |
| Bra007270 | 990 | Down | 0.00077252 |
| Bra012257 | 1176 | Down | 0.00077288 |
| Bra016461 | 294 | Down | 0.00077264 |
| Bra011071 | 4239 | Down | 1.05E-11 |
| Bra033092 | 924 | Down | 1.61E-06 |
| Bra033149 | 1644 | Down | 0 |
| Bra006320 | 549 | Down | 5.36E-05 |
| Bra019089 | 918 | Down | 4.59E-10 |
| Bra032033 | 159 | Down | 0 |
| Bra033656 | 888 | Down | 5.55E-07 |
| Bra013007 | 5832 | Down | 0 |
| Bra008736 | 1038 | Down | 0.00027663 |
| Bra003662 | 339 | Down | 0 |
| Bra024955 | 1002 | Down | 0 |
| Bra012779 | 2055 | Down | 8.17E-12 |
| Bra040115 | 402 | Down | 0 |
| Bra038466 | 387 | Down | 4.32E-05 |
| Bra036125 | 1851 | Down | 2.99E-08 |
| Bra025237 | 1320 | Down | 6.73E-06 |
| Bra006899 | 1041 | Down | 6.87E-08 |
| Bra025912 | 2943 | Down | 1.04E-08 |
| Bra034728 | 885 | Down | 3.15E-12 |
| Bra025297 | 801 | Down | 1.11E-11 |
| Bra002983 | 342 | Down | 1.53E-05 |
| Bra015153 | 438 | Down | 9.77E-14 |
| Bra004438 | 3027 | Down | 0 |
| Bra023898 | 1269 | Down | 1.19E-12 |
| Bra005501 | 720 | Down | 5.53E-08 |
| Bra018707 | 1419 | Down | 0.00022268 |
| Bra024423 | 981 | Down | 1.27E-07 |
| Bra002460 | 501 | Down | 2.92E-09 |
| Bra019245 | 474 | Down | 4.43E-10 |
| Bra005460 | 1011 | Down | 7.92E-05 |
| Bra013059 | 1650 | Down | 1.23E-05 |
| Bra002436 | 426 | Down | 0 |
| Bra016010 | 501 | Down | 1.46E-12 |
| Bra006492 | 1080 | Down | 4.36E-06 |
| Bra040529 | 846 | Down | 2.80E-05 |
| Bra024577 | 354 | Down | 2.86E-10 |
| Bra008425 | 705 | Down | 5.33E-07 |
| Bra037398 | 972 | Down | 0 |
| Bra017782 | 942 | Down | 1.51E-09 |
| Bra039681 | 735 | Down | 1.86E-07 |
| Bra001579 | 1224 | Down | 2.75E-12 |
| Bra030182 | 804 | Down | 0 |
| Bra008241 | 1647 | Down | 4.76E-12 |
| Bra032646 | 1620 | Down | 2.31E-08 |
| Bra028590 | 750 | Down | 1.59E-12 |
| Bra020536 | 645 | Down | 2.07E-11 |
| Bra030842 | 1347 | Down | 4.23E-10 |
| Bra013576 | 996 | Down | 2.92E-10 |
| Bra012986 | 918 | Down | 0.00091614 |
| Bra039229 | 1290 | Down | 0.00091599 |
| Bra025274 | 1239 | Down | 0.00091585 |
| Bra019071 | 1437 | Down | 0 |
| Bra035092 | 951 | Down | 1.75E-11 |
| Bra021586 | 1053 | Down | 6.44E-06 |
| Bra013607 | 1332 | Down | 6.44E-06 |
| Bra004588 | 1491 | Down | 1.10E-12 |
| Bra012995 | 3828 | Down | 0 |
| Bra037501 | 1278 | Down | 4.12E-05 |
| Bra031812 | 669 | Down | 4.12E-05 |
| Bra027148 | 1611 | Down | 1.09E-12 |
| Bra017661 | 669 | Down | 5.18E-06 |
| Bra004121 | 1605 | Down | 0 |
| Bra036618 | 249 | Down | 5.00E-10 |
| Bra017426 | 414 | Down | 0.00073702 |
| Bra004786 | 2574 | Down | 1.75E-10 |
| Bra029784 | 222 | Down | 0.00026339 |
| Bra024707 | 1344 | Down | 0.00026335 |
| Bra009846 | 1488 | Down | 0.0002633 |
| Bra025391 | 1236 | Down | 9.36E-05 |
| Bra020577 | 1062 | Down | 9.36E-05 |
| Bra019109 | 969 | Down | 9.35E-05 |
| Bra038642 | 876 | Down | 3.30E-09 |
| Bra037765 | 195 | Down | 3.31E-05 |
| Bra025147 | 1158 | Down | 4.03E-10 |
| Bra012141 | 2055 | Down | 1.62E-11 |
| Bra013783 | 1284 | Down | 1.77E-07 |
| Bra024871 | 1716 | Down | 0 |
| Bra015088 | 621 | Down | 4.12E-12 |
| Bra024481 | 771 | Down | 0 |
| Bra004138 | 489 | Down | 0 |
| Bra038737 | 1122 | Down | 1.17E-06 |
| Bra019092 | 2154 | Down | 0.00059366 |
| Bra038827 | 1263 | Down | 0.00059357 |
| Bra009022 | 912 | Down | 3.33E-06 |
| Bra032627 | 786 | Down | 0.00059376 |
| Bra020607 | 1827 | Down | 1.62E-12 |
| Bra012258 | 951 | Down | 7.57E-06 |
| Bra001980 | 1674 | Down | 6.04E-05 |
| Bra018559 | 1545 | Down | 0 |
| Bra035429 | 1785 | Down | 0.00016956 |
| Bra008013 | 1491 | Down | 3.80E-10 |
| Bra004209 | 327 | Down | 7.46E-07 |
| Bra025138 | 744 | Down | 3.12E-09 |
| Bra012355 | 1767 | Down | 2.13E-06 |
| Bra022820 | 2280 | Down | 2.13E-06 |
| Bra030129 | 1461 | Down | 6.09E-06 |
| Bra038911 | 2418 | Down | 5.54E-13 |
| Bra023970 | 168 | Down | 1.72E-06 |
| Bra003916 | 1989 | Down | 0 |
| Bra031902 | 237 | Down | 0 |
| Bra012496 | 642 | Down | 1.38E-05 |
| Bra001028 | 432 | Down | 7.84E-12 |
| Bra040793 | 1092 | Down | 4.72E-08 |
| Bra000674 | 2250 | Down | 1.53E-11 |
| Bra033422 | 441 | Down | 0 |
| Bra008655 | 1065 | Down | 1.22E-12 |
| Bra038594 | 1416 | Down | 0.00030801 |
| Bra018986 | 987 | Down | 4.54E-12 |
| Bra004106 | 216 | Down | 1.80E-12 |
| Bra032539 | 867 | Down | 0 |
| Bra038537 | 1431 | Down | 0.00086031 |
| Bra008045 | 1284 | Down | 2.24E-11 |
| Bra009858 | 1572 | Down | 7.92E-12 |
| Bra019741 | 429 | Down | 2.50E-05 |
| Bra035348 | 552 | Down | 2.50E-05 |
| Bra011893 | 744 | Down | 2.89E-12 |
| Bra000509 | 690 | Down | 0.00024724 |
| Bra013694 | 1584 | Down | 0.00024733 |
| Bra017737 | 1251 | Down | 0.00024728 |
| Bra040415 | 402 | Down | 1.44E-11 |
| Bra024326 | 243 | Down | 1.95E-07 |
| Bra028778 | 540 | Down | 0 |
| Bra024069 | 1911 | Down | 7.05E-05 |
| Bra012204 | 663 | Down | 7.05E-05 |
| Bra009003 | 1305 | Down | 2.01E-05 |
| Bra009417 | 2385 | Down | 1.61E-06 |
| Bra034070 | 372 | Down | 0 |
| Bra032506 | 423 | Down | 0 |
| Bra005923 | 840 | Down | 0 |
| Bra001734 | 1962 | Down | 0 |
| Bra027735 | 1863 | Down | 2.10E-12 |
| Bra016676 | 1617 | Down | 4.56E-06 |
| Bra024117 | 3186 | Down | 0 |
| Bra037735 | 285 | Down | 1.28E-06 |
| Bra038063 | 879 | Down | 0.00055635 |
| Bra008644 | 996 | Down | 3.65E-06 |
| Bra025233 | 1905 | Down | 1.75E-09 |
| Bra031602 | 1350 | Down | 0 |
| Bra008070 | 921 | Down | 4.85E-10 |
| Bra040508 | 1458 | Down | 0.00012728 |
| Bra021273 | 390 | Down | 3.89E-10 |
| Bra014972 | 990 | Down | 1.03E-05 |
| Bra030792 | 495 | Down | 8.75E-13 |
| Bra012871 | 3045 | Down | 2.93E-12 |
| Bra013485 | 3108 | Down | 8.52E-11 |
| Bra034394 | 624 | Down | 5.40E-11 |
| Bra000194 | 774 | Down | 6.50E-07 |
| Bra007151 | 1347 | Down | 8.51E-12 |
| Bra023926 | 939 | Down | 8.25E-06 |
| Bra009710 | 1350 | Down | 3.19E-09 |
| Bra038461 | 762 | Down | 0.00010218 |
| Bra000011 | 1044 | Down | 1.43E-11 |
| Bra007007 | 1221 | Down | 1.46E-07 |
| Bra009706 | 480 | Down | 0 |
| Bra012616 | 402 | Down | 0 |
| Bra031807 | 2694 | Down | 5.28E-11 |
| Bra028498 | 1950 | Down | 2.24E-12 |
| Bra008711 | 3690 | Down | 3.27E-08 |
| Bra015403 | 963 | Down | 5.33E-12 |
| Bra021175 | 780 | Down | 8.18E-05 |
| Bra038766 | 2295 | Down | 1.25E-10 |
| Bra039438 | 660 | Down | 4.51E-10 |
| Bra017443 | 1644 | Down | 3.01E-12 |
| Bra014094 | 1257 | Down | 1.62E-09 |
| Bra000711 | 627 | Down | 0 |
| Bra034552 | 2559 | Down | 3.30E-07 |
| Bra020113 | 636 | Down | 8.49E-11 |
| Bra030722 | 351 | Down | 0 |
| Bra027986 | 1068 | Down | 0 |
| Bra039182 | 627 | Down | 0 |
| Bra006171 | 2574 | Down | 0 |
| Bra002762 | 3222 | Down | 1.49E-05 |
| Bra029852 | 1551 | Down | 1.09E-11 |
| Bra017051 | 2154 | Down | 1.82E-10 |
| Bra009656 | 450 | Down | 0 |
| Bra011769 | 1287 | Down | 0.00079745 |
| Bra029829 | 2988 | Down | 0.00079733 |
| Bra034612 | 1107 | Down | 1.19E-05 |
| Bra017644 | 426 | Down | 0 |
| Bra024404 | 1080 | Down | 0 |
| Bra003518 | 1785 | Down | 2.10E-11 |
| Bra034712 | 1662 | Down | 0.00064058 |
| Bra017410 | 459 | Down | 0.0001465 |
| Bra010696 | 327 | Down | 4.94E-12 |
| Bra014044 | 1440 | Down | 0 |
| Bra015243 | 1527 | Down | 0 |
| Bra008449 | 1269 | Down | 3.67E-11 |
| Bra007989 | 1554 | Down | 0.00011804 |
| Bra007493 | 1338 | Down | 1.54E-08 |
| Bra009203 | 2268 | Down | 7.15E-12 |
| Bra031034 | 705 | Down | 6.94E-13 |
| Bra008524 | 975 | Down | 1.10E-06 |
| Bra019155 | 603 | Down | 2.14E-05 |
| Bra025226 | 1494 | Down | 8.44E-11 |
| Bra018614 | 816 | Down | 3.08E-10 |
| Bra012956 | 3162 | Down | 0 |
| Bra032730 | 1356 | Down | 7.53E-05 |
| Bra035650 | 1134 | Down | 0 |
| Bra016055 | 1539 | Down | 2.44E-10 |
| Bra007845 | 2871 | Down | 2.79E-08 |
| Bra001474 | 1518 | Down | 0 |
| Bra040203 | 1101 | Down | 0 |
| Bra011816 | 2145 | Down | 1.37E-05 |
| Bra019226 | 630 | Down | 0 |
| Bra005423 | 1491 | Down | 0 |
| Bra021184 | 810 | Down | 6.03E-05 |
| Bra005209 | 624 | Down | 0 |
| Bra033698 | 2925 | Down | 2.34E-12 |
| Bra011548 | 1197 | Down | 1.98E-06 |
| Bra030116 | 1137 | Down | 0 |
| Bra011325 | 1098 | Down | 1.18E-11 |
| Bra028546 | 534 | Down | 0 |
| Bra036170 | 1959 | Down | 0 |
| Bra013200 | 1059 | Down | 2.08E-12 |
| Bra040902 | 1719 | Down | 0 |
| Bra011280 | 1539 | Down | 0 |
| Bra009169 | 2559 | Down | 2.24E-07 |
| Bra002316 | 636 | Down | 2.70E-11 |
| Bra008310 | 1077 | Down | 0.00016826 |
| Bra004031 | 2775 | Down | 8.48E-12 |
| Bra011649 | 1260 | Down | 4.51E-09 |
| Bra040831 | 615 | Down | 2.46E-05 |
| Bra026487 | 834 | Down | 3.59E-09 |
| Bra012174 | 1881 | Down | 6.35E-07 |
| Bra024281 | 3738 | Down | 0.00073326 |
| Bra009433 | 888 | Down | 0 |
| Bra039484 | 597 | Down | 0.00073337 |
| Bra025056 | 1848 | Down | 0.00073314 |
| Bra040094 | 858 | Down | 4.76E-12 |
| Bra005979 | 1440 | Down | 1.59E-12 |
| Bra000009 | 657 | Down | 0 |
| Bra015020 | 2175 | Down | 2.85E-06 |
| Bra014133 | 2157 | Down | 0.000587 |
| Bra038795 | 1338 | Down | 1.45E-09 |
| Bra028882 | 954 | Down | 6.90E-05 |
| Bra020681 | 621 | Down | 0 |
| Bra018007 | 1062 | Down | 5.82E-10 |
| Bra008030 | 891 | Down | 1.24E-11 |
| Bra000708 | 843 | Down | 7.66E-11 |
| Bra016525 | 2106 | Down | 0.00037559 |
| Bra037785 | 531 | Down | 9.17E-07 |
| Bra003498 | 195 | Down | 6.41E-06 |
| Bra038166 | 1275 | Down | 1.44E-11 |
| Bra012734 | 2463 | Down | 0 |
| Bra021440 | 489 | Down | 0 |
| Bra009909 | 1254 | Down | 5.12E-06 |
| Bra040561 | 714 | Down | 4.07E-06 |
| Bra033420 | 216 | Down | 1.44E-11 |
| Bra025895 | 705 | Down | 9.79E-12 |
| Bra003821 | 360 | Down | 0.00019212 |
| Bra030072 | 867 | Down | 4.61E-12 |
| Bra033830 | 582 | Down | 2.24E-05 |
| Bra032542 | 2094 | Down | 2.92E-07 |
| Bra000393 | 642 | Down | 3.89E-12 |
| Bra007033 | 249 | Down | 0 |
| Bra023574 | 1842 | Down | 1.43E-05 |
| Bra001664 | 1731 | Down | 1.89E-09 |
| Bra022675 | 726 | Down | 1.89E-09 |
| Bra039503 | 1002 | Down | 0.00012302 |
| Bra020549 | 789 | Down | 0 |
| Bra027391 | 2313 | Down | 0 |
| Bra040533 | 780 | Down | 0 |
| Bra020007 | 3147 | Down | 8.51E-12 |
| Bra018017 | 621 | Down | 2.76E-12 |
| Bra004806 | 729 | Down | 8.15E-12 |
| Bra019088 | 165 | Down | 0.00083179 |
| Bra013152 | 1401 | Down | 8.63E-13 |
| Bra036992 | 2070 | Down | 5.24E-07 |
| Bra010816 | 2322 | Down | 3.06E-10 |
| Bra014911 | 2124 | Down | 4.53E-12 |
| Bra040539 | 375 | Down | 2.28E-12 |
| Bra009549 | 1551 | Down | 0.00053386 |
| Bra028047 | 1293 | Down | 2.95E-06 |
| Bra007864 | 750 | Down | 0 |
| Bra036919 | 330 | Down | 1.09E-09 |
| Bra040632 | 591 | Down | 0.00034063 |
| Bra009085 | 390 | Down | 0.00034057 |
| Bra009978 | 1614 | Down | 0 |
| Bra041005 | 1917 | Down | 1.19E-11 |
| Bra027227 | 348 | Down | 1.50E-06 |
| Bra038644 | 1473 | Down | 1.50E-06 |
| Bra039396 | 3126 | Down | 1.50E-06 |
| Bra016488 | 498 | Down | 2.03E-05 |
| Bra028401 | 1035 | Down | 4.35E-10 |
| Bra029931 | 621 | Down | 8.70E-12 |
| Bra029445 | 1548 | Down | 1.71E-12 |
| Bra012753 | 1446 | Down | 5.49E-12 |
| Bra037714 | 210 | Down | 1.38E-10 |
| Bra013899 | 603 | Down | 1.04E-05 |
| Bra014858 | 249 | Down | 0 |
| Bra040682 | 1242 | Down | 0.0001386 |
| Bra016570 | 984 | Down | 2.93E-12 |
| Bra038844 | 2448 | Down | 0 |
| Bra009777 | 1209 | Down | 0.0001114 |
| Bra020471 | 870 | Down | 0.00011137 |
| Bra025379 | 1539 | Down | 1.10E-08 |
| Bra000640 | 963 | Down | 0 |
| Bra028357 | 1203 | Down | 6.91E-09 |
| Bra025104 | 1626 | Down | 4.38E-09 |
| Bra012293 | 1491 | Down | 1.24E-11 |
| Bra026282 | 909 | Down | 1.32E-12 |
| Bra004177 | 942 | Down | 2.79E-11 |
| Bra009695 | 1821 | Down | 0 |
| Bra004946 | 2025 | Down | 1.40E-09 |
| Bra029655 | 912 | Down | 0.00093899 |
| Bra003439 | 2358 | Down | 3.09E-08 |
| Bra019082 | 1188 | Down | 0 |
| Bra036761 | 1656 | Down | 0.00060146 |
| Bra001592 | 492 | Down | 4.25E-07 |
| Bra016916 | 2667 | Down | 0.00060155 |
| Bra029732 | 867 | Down | 4.76E-11 |
| Bra018518 | 1323 | Down | 9.31E-06 |
| Bra020450 | 966 | Down | 0.00038438 |
| Bra041032 | 471 | Down | 0.00038444 |
| Bra027700 | 2481 | Down | 1.88E-13 |
| Bra028134 | 3087 | Down | 1.37E-07 |
| Bra022146 | 384 | Down | 0.00030738 |
| Bra028373 | 3330 | Down | 0.00024567 |
| Bra020928 | 2535 | Down | 6.95E-08 |
| Bra010028 | 2169 | Down | 1.07E-11 |
| Bra025025 | 1413 | Down | 4.51E-13 |
| Bra039262 | 1314 | Down | 3.00E-06 |
| Bra024991 | 1047 | Down | 4.40E-08 |
| Bra028061 | 1482 | Down | 4.47E-12 |
| Bra019358 | 1065 | Down | 2.39E-06 |
| Bra019864 | 2523 | Down | 0 |
| Bra011000 | 1254 | Down | 0.00015598 |
| Bra002346 | 1833 | Down | 7.69E-13 |
| Bra040322 | 702 | Down | 3.04E-07 |
| Bra011890 | 1170 | Down | 4.05E-05 |
| Bra025114 | 1881 | Down | 2.06E-05 |
| Bra040119 | 786 | Down | 0 |
| Bra039018 | 396 | Down | 4.96E-08 |
| Bra017730 | 2520 | Down | 2.76E-12 |
| Bra007215 | 765 | Down | 7.12E-12 |
| Bra001127 | 789 | Down | 0 |
| Bra021909 | 486 | Down | 1.69E-10 |
| Bra019299 | 1482 | Down | 1.12E-12 |
| Bra010253 | 588 | Down | 7.92E-09 |
| Bra001093 | 2442 | Down | 0 |
| Bra040530 | 1047 | Down | 2.69E-06 |
| Bra036630 | 1149 | Down | 1.36E-06 |
| Bra017912 | 1419 | Down | 0 |
| Bra014520 | 1347 | Down | 0.00067365 |
| Bra009327 | 471 | Down | 3.20E-10 |
| Bra022255 | 1137 | Down | 3.42E-07 |
| Bra028900 | 4548 | Down | 2.76E-12 |
| Bra017424 | 963 | Down | 6.44E-11 |
| Bra018960 | 1281 | Down | 5.17E-11 |
| Bra008555 | 1530 | Down | 0.00021972 |
| Bra019133 | 1197 | Down | 5.70E-12 |
| Bra008004 | 1194 | Down | 0.00017521 |
| Bra006343 | 1515 | Down | 2.81E-08 |
| Bra018926 | 1446 | Down | 0 |
| Bra003843 | 1149 | Down | 0.00011215 |
| Bra025873 | 732 | Down | 5.70E-05 |
| Bra030937 | 921 | Down | 5.70E-05 |
| Bra008052 | 876 | Down | 2.30E-05 |
| Bra004883 | 738 | Down | 1.85E-05 |
| Bra032101 | 879 | Down | 1.45E-12 |
| Bra036938 | 774 | Down | 4.61E-12 |
| Bra023110 | 1929 | Down | 0 |
| Bra034613 | 1305 | Down | 1.91E-06 |
| Bra008376 | 1035 | Down | 1.92E-06 |
| Bra009680 | 1764 | Down | 1.92E-06 |
| Bra006782 | 2709 | Down | 5.69E-12 |
| Bra033517 | 1704 | Down | 0 |
| Bra039164 | 654 | Down | 1.55E-07 |
| Bra029752 | 972 | Down | 1.08E-11 |
| Bra020369 | 906 | Down | 8.49E-13 |
| Bra010009 | 435 | Down | 3.14E-08 |
| Bra006553 | 870 | Down | 0 |
| Bra016751 | 333 | Down | 4.91E-11 |
| Bra003808 | 339 | Down | 8.33E-12 |
| Bra023838 | 1548 | Down | 0 |
| Bra005038 | 465 | Down | 3.27E-12 |
| Bra032828 | 531 | Down | 0 |
| Bra015132 | 594 | Down | 0 |
| Bra017279 | 2637 | Down | 0 |
| Bra035633 | 906 | Down | 2.13E-06 |
| Bra012082 | 2010 | Down | 5.40E-07 |
| Bra026499 | 1488 | Down | 3.24E-05 |
| Bra031027 | 2850 | Down | 2.57E-05 |
| Bra012508 | 1242 | Down | 6.65E-06 |
| Bra039488 | 543 | Down | 1.10E-07 |
| Bra001120 | 1371 | Down | 0.00093949 |
| Bra012580 | 549 | Down | 0 |
| Bra008332 | 465 | Down | 3.24E-05 |
| Bra038465 | 381 | Down | 8.58E-07 |
| Bra025273 | 1776 | Down | 0.00047996 |
| Bra010982 | 690 | Down | 4.28E-07 |
| Bra036473 | 3618 | Down | 2.13E-06 |
| Bra019897 | 1989 | Down | 0.00048004 |
| Bra038330 | 1362 | Down | 1.31E-05 |
| Bra000361 | 600 | Down | 0.00015592 |
| Bra019137 | 2439 | Down | 1.14E-10 |
| Bra016870 | 942 | Down | 2.06E-05 |
| Bra020489 | 1491 | Down | 2.17E-07 |
| Bra023634 | 915 | Down | 4.77E-12 |
| Bra017204 | 501 | Down | 1.72E-12 |
| Bra013254 | 3666 | Down | 1.27E-10 |
| Bra014967 | 948 | Down | 3.12E-08 |
| Bra008162 | 306 | Down | 0 |
| Bra035162 | 1047 | Down | 3.81E-07 |
| Bra024843 | 1827 | Down | 4.94E-12 |
| Bra007296 | 1212 | Down | 1.46E-05 |
| Bra019912 | 1602 | Down | 1.40E-12 |
| Bra019410 | 3471 | Down | 2.29E-05 |
| Bra040575 | 663 | Down | 3.60E-05 |
| Bra003512 | 1218 | Down | 0 |
| Bra015971 | 1539 | Down | 0 |
| Bra024788 | 1008 | Down | 1.75E-08 |
| Bra025277 | 1035 | Down | 0.00013859 |
| Bra040785 | 1566 | Down | 0 |
| Bra009109 | 219 | Down | 0.00017358 |
| Bra027699 | 1866 | Down | 1.95E-11 |
| Bra021935 | 882 | Down | 1.95E-11 |
| Bra010546 | 603 | Down | 0.0003408 |
| Bra034943 | 1629 | Down | 5.43E-12 |
| Bra034639 | 429 | Down | 0.00042701 |
| Bra029785 | 807 | Down | 2.77E-12 |
| Bra023526 | 2556 | Down | 0.00053465 |
| Bra004404 | 786 | Down | 0.00053474 |
| Bra015225 | 585 | Down | 4.23E-07 |
| Bra023843 | 408 | Down | 8.05E-13 |
| Bra026065 | 1521 | Down | 0.00083327 |
| Bra030743 | 798 | Down | 8.48E-07 |
| Bra010243 | 702 | Down | 1.56E-09 |
| Bra024023 | 1929 | Down | 1.69E-06 |
| Bra003899 | 1140 | Down | 4.17E-06 |
| Bra008464 | 1254 | Down | 5.24E-06 |
| Bra024864 | 549 | Down | 2.81E-12 |
| Bra007730 | 1608 | Down | 8.23E-06 |
| Bra031959 | 1635 | Down | 2.45E-08 |
| Bra023029 | 735 | Down | 8.23E-06 |
| Bra013766 | 1491 | Down | 8.23E-06 |
| Bra024015 | 702 | Down | 3.86E-08 |
| Bra025940 | 501 | Down | 0 |
| Bra026579 | 1089 | Down | 2.03E-05 |
| Bra006786 | 3144 | Down | 2.03E-05 |
| Bra040452 | 942 | Down | 3.55E-12 |
| Bra035079 | 2298 | Down | 3.19E-05 |
| Bra034799 | 624 | Down | 0 |
| Bra037977 | 1239 | Down | 7.84E-05 |
| Bra038667 | 678 | Down | 1.80E-11 |
| Bra007015 | 3024 | Down | 9.41E-07 |
| Bra012918 | 555 | Down | 1.09E-08 |
| Bra029943 | 1815 | Down | 4.48E-12 |
| Bra000355 | 627 | Down | 1.94E-10 |
| Bra039735 | 1413 | Down | 0 |
| Bra026794 | 444 | Down | 0.00015347 |
| Bra036285 | 600 | Down | 0 |
| Bra021570 | 1527 | Down | 5.62E-13 |
| Bra027555 | 1602 | Down | 2.42E-09 |
| Bra001719 | 339 | Down | 6.04E-09 |
| Bra036979 | 1281 | Down | 1.15E-05 |
| Bra018120 | 873 | Down | 0.00047168 |
| Bra022440 | 2316 | Down | 0 |
| Bra014309 | 2565 | Down | 1.79E-05 |
| Bra019589 | 1428 | Down | 8.64E-13 |
| Bra030025 | 960 | Down | 5.62E-13 |
| Bra013945 | 1278 | Down | 2.40E-08 |
| Bra038584 | 864 | Down | 1.56E-11 |
| Bra032349 | 1230 | Down | 5.72E-12 |
| Bra030918 | 936 | Down | 0.00092257 |
| Bra027705 | 804 | Down | 1.04E-06 |
| Bra001817 | 1140 | Down | 1.31E-06 |
| Bra019658 | 1317 | Down | 0 |
| Bra014808 | 411 | Down | 4.41E-05 |
| Bra035637 | 411 | Down | 5.52E-05 |
| Bra039286 | 1044 | Down | 6.93E-05 |
| Bra030831 | 978 | Down | 5.33E-09 |
| Bra012620 | 1056 | Down | 6.68E-09 |
| Bra010500 | 2361 | Down | 0 |
| Bra023820 | 789 | Down | 2.65E-08 |
| Bra004648 | 2265 | Down | 0.00016951 |
| Bra014984 | 1458 | Down | 4.18E-08 |
| Bra033597 | 3660 | Down | 7.30E-12 |
| Bra030052 | 1293 | Down | 4.67E-09 |
| Bra005425 | 801 | Down | 0 |
| Bra020009 | 495 | Down | 0 |
| Bra015131 | 2286 | Down | 0 |
| Bra013284 | 402 | Down | 4.48E-12 |
| Bra025349 | 918 | Down | 0.0003324 |
| Bra000390 | 1539 | Down | 2.47E-05 |
| Bra023513 | 321 | Down | 4.76E-12 |
| Bra021189 | 579 | Down | 5.43E-12 |
| Bra024936 | 1005 | Down | 6.39E-12 |
| Bra002021 | 1095 | Down | 2.04E-07 |
| Bra002045 | 489 | Down | 2.28E-12 |
| Bra003991 | 363 | Down | 1.01E-11 |
| Bra025370 | 903 | Down | 0.00052037 |
| Bra035574 | 348 | Down | 2.60E-09 |
| Bra007259 | 1482 | Down | 4.49E-06 |
| Bra008136 | 3015 | Down | 0 |
| Bra000575 | 1557 | Down | 0.00064952 |
| Bra001049 | 786 | Down | 1.01E-11 |
| Bra026417 | 1542 | Down | 9.15E-10 |
| Bra021645 | 1137 | Down | 1.40E-12 |
| Bra009678 | 2226 | Down | 0 |
| Bra025446 | 744 | Down | 0.0001487 |
| Bra024261 | 513 | Down | 0 |
| Bra019012 | 825 | Down | 3.21E-08 |
| Bra010128 | 627 | Down | 2.51E-06 |
| Bra015949 | 2592 | Down | 2.72E-05 |
| Bra024861 | 1389 | Down | 7.68E-14 |
| Bra040178 | 831 | Down | 8.99E-09 |
| Bra025364 | 3741 | Down | 5.02E-12 |
| Bra022536 | 696 | Down | 5.34E-05 |
| Bra013676 | 1935 | Down | 5.34E-05 |
| Bra000131 | 615 | Down | 7.76E-06 |
| Bra027831 | 2241 | Down | 0.00045628 |
| Bra037056 | 453 | Down | 1.76E-06 |
| Bra004147 | 1092 | Down | 4.45E-08 |
| Bra011993 | 2901 | Down | 0 |
| Bra026272 | 798 | Down | 1.52E-05 |
| Bra010018 | 1251 | Down | 1.16E-11 |
| Bra039010 | 1092 | Down | 0.00010491 |
| Bra004225 | 528 | Down | 1.17E-11 |
| Bra030739 | 4662 | Down | 4.88E-07 |
| Bra010807 | 804 | Down | 5.74E-12 |
| Bra007901 | 1020 | Down | 0.00071189 |
| Bra010115 | 1755 | Down | 1.97E-08 |
| Bra000135 | 453 | Down | 0 |
| Bra034860 | 744 | Down | 2.43E-11 |
| Bra036596 | 2124 | Down | 0 |
| Bra039097 | 1170 | Down | 0.00016342 |
| Bra012810 | 1071 | Down | 0.00088961 |
| Bra025022 | 1530 | Down | 6.79E-06 |
| Bra025170 | 957 | Down | 3.74E-05 |
| Bra013616 | 387 | Down | 4.67E-05 |
| Bra022859 | 750 | Down | 3.00E-12 |
| Bra000692 | 459 | Down | 0 |
| Bra019574 | 621 | Down | 1.17E-11 |
| Bra000207 | 954 | Down | 0.00025626 |
| Bra001996 | 1911 | Down | 0.00025621 |
| Bra022514 | 615 | Down | 0.0002563 |
| Bra009908 | 1092 | Down | 1.07E-05 |
| Bra006419 | 849 | Down | 2.41E-06 |
| Bra022336 | 1707 | Down | 3.02E-06 |
| Bra011477 | 171 | Down | 7.31E-05 |
| Bra011682 | 1254 | Down | 1.67E-05 |
| Bra014375 | 1119 | Down | 0 |
| Bra032020 | 339 | Down | 0.0003993 |
| Bra009949 | 459 | Down | 3.23E-13 |
| Bra015448 | 1362 | Down | 2.09E-05 |
| Bra026672 | 663 | Down | 4.75E-06 |
| Bra019122 | 1068 | Down | 1.07E-06 |
| Bra012475 | 1227 | Down | 1.14E-12 |
| Bra006328 | 1707 | Down | 0.00011479 |
| Bra032644 | 861 | Down | 1.34E-06 |
| Bra029537 | 1515 | Down | 3.68E-11 |
| Bra019438 | 1470 | Down | 0.00011477 |
| Bra024909 | 864 | Down | 0.00014284 |
| Bra009769 | 615 | Down | 0.00062465 |
| Bra038784 | 2283 | Down | 0 |
| Bra006315 | 1092 | Down | 0 |
| Bra000069 | 1107 | Down | 1.17E-05 |
| Bra004501 | 894 | Down | 0.00077797 |
| Bra023644 | 321 | Down | 9.31E-07 |
| Bra018797 | 1962 | Down | 0 |
| Bra006057 | 5268 | Down | 0 |
| Bra038573 | 3660 | Down | 0.000969 |
| Bra003155 | 339 | Down | 3.25E-07 |
| Bra006922 | 1644 | Down | 6.68E-12 |
| Bra023324 | 1023 | Down | 5.33E-12 |
| Bra033710 | 2253 | Down | 0.00034913 |
| Bra032494 | 1296 | Down | 0.00034907 |
| Bra031810 | 924 | Down | 0.00010014 |
| Bra039627 | 2049 | Down | 0 |
| Bra014985 | 759 | Down | 3.58E-05 |
| Bra010867 | 3000 | Down | 1.75E-09 |
| Bra020572 | 1407 | Down | 0 |
| Bra037544 | 1029 | Down | 2.83E-07 |
| Bra012507 | 1497 | Down | 3.31E-12 |
| Bra033560 | 1986 | Down | 7.43E-11 |
| Bra022554 | 375 | Down | 2.04E-12 |
| Bra028148 | 507 | Down | 0 |
| Bra038689 | 624 | Down | 0 |
| Bra023771 | 516 | Down | 0.00019497 |
| Bra027185 | 1782 | Down | 1.22E-09 |
| Bra015827 | 1584 | Down | 1.53E-09 |
| Bra020106 | 402 | Down | 6.98E-05 |
| Bra022618 | 1821 | Down | 6.98E-05 |
| Bra000592 | 2481 | Down | 1.88E-11 |
| Bra037191 | 375 | Down | 1.97E-07 |
| Bra010817 | 2277 | Down | 1.97E-07 |
| Bra034271 | 750 | Down | 0.00024411 |
| Bra031353 | 285 | Down | 8.72E-05 |
| Bra007572 | 255 | Down | 6.69E-12 |
| Bra030704 | 312 | Down | 1.11E-05 |
| Bra013045 | 291 | Down | 3.94E-06 |
| Bra030045 | 1416 | Down | 9.35E-14 |
| Bra012069 | 2220 | Down | 1.64E-10 |
| Bra020454 | 363 | Down | 1.71E-08 |
| Bra007556 | 456 | Down | 3.56E-12 |
| Bra035169 | 1221 | Down | 2.35E-12 |
| Bra019179 | 606 | Down | 2.35E-12 |
| Bra002181 | 4101 | Down | 2.14E-07 |
| Bra014865 | 1134 | Down | 7.62E-08 |
| Bra012447 | 1542 | Down | 0 |
| Bra037764 | 354 | Down | 0.00047409 |
| Bra008533 | 510 | Down | 2.21E-11 |
| Bra024164 | 1170 | Down | 4.07E-13 |
| Bra003226 | 735 | Down | 0 |
| Bra011031 | 1059 | Down | 6.07E-05 |
| Bra020722 | 273 | Down | 2.16E-05 |
| Bra025969 | 234 | Down | 2.73E-06 |
| Bra018725 | 1038 | Down | 1.48E-08 |
| Bra004392 | 747 | Down | 5.02E-12 |
| Bra021276 | 1065 | Down | 6.48E-09 |
| Bra020519 | 330 | Down | 0.00059238 |
| Bra019049 | 1197 | Down | 1.03E-11 |
| Bra024093 | 267 | Down | 1.20E-05 |
| Bra013015 | 2409 | Down | 3.38E-05 |
| Bra012370 | 471 | Down | 0 |
| Bra025090 | 3477 | Down | 7.04E-11 |
| Bra019220 | 1389 | Down | 0.00073741 |
| Bra027697 | 930 | Down | 0.00073753 |
| Bra022351 | 339 | Down | 1.58E-09 |
| Bra014910 | 1551 | Down | 3.65E-08 |
| Bra037196 | 765 | Down | 8.29E-11 |
| Bra038499 | 792 | Down | 0.00011868 |
| Bra002664 | 2169 | Down | 0.0001187 |
| Bra015047 | 2001 | Down | 5.72E-12 |
| Bra019193 | 411 | Down | 4.48E-12 |
| Bra012827 | 1506 | Down | 7.04E-09 |
| Bra012558 | 3978 | Down | 6.19E-11 |
| Bra002999 | 798 | Down | 0 |
| Bra025365 | 507 | Down | 8.37E-06 |
| Bra026773 | 897 | Down | 0.00014727 |
| Bra023530 | 1026 | Down | 1.11E-08 |
| Bra035535 | 381 | Down | 1.57E-11 |
| Bra004143 | 684 | Down | 6.60E-05 |
| Bra037884 | 564 | Down | 5.51E-12 |
| Bra017916 | 741 | Down | 3.58E-12 |
| Bra000203 | 1023 | Down | 0.00051504 |
| Bra007816 | 2925 | Down | 5.20E-10 |
| Bra015105 | 1395 | Down | 2.20E-08 |
| Bra001003 | 1146 | Down | 5.83E-06 |
| Bra040801 | 1512 | Down | 0 |
| Bra022459 | 1686 | Down | 5.94E-12 |
| Bra029953 | 2655 | Down | 1.64E-05 |
| Bra005820 | 408 | Down | 1.46E-12 |
| Bra016491 | 1194 | Down | 7.80E-08 |
| Bra013464 | 2577 | Down | 7.25E-06 |
| Bra014334 | 729 | Down | 2.12E-11 |
| Bra030386 | 1581 | Down | 5.01E-12 |
| Bra025730 | 699 | Down | 5.49E-12 |
| Bra030866 | 1269 | Down | 0 |
| Bra011408 | 432 | Down | 0.00028763 |
| Bra033744 | 984 | Down | 0.00012823 |
| Bra020260 | 1602 | Down | 0.0001282 |
| Bra016269 | 360 | Down | 6.96E-12 |
| Bra032139 | 660 | Down | 1.39E-10 |
| Bra017697 | 846 | Down | 6.04E-11 |
| Bra000454 | 1089 | Down | 1.48E-11 |
| Bra034718 | 1014 | Down | 0.00015949 |
| Bra038614 | 1242 | Down | 8.33E-12 |
| Bra013012 | 2295 | Down | 1.74E-10 |
| Bra002290 | 684 | Down | 0 |
| Bra037482 | 1953 | Down | 9.86E-07 |
| Bra029771 | 372 | Down | 0 |
| Bra024059 | 1143 | Down | 1.90E-07 |
| Bra012946 | 1131 | Down | 1.42E-05 |
| Bra028974 | 1203 | Down | 4.23E-11 |
| Bra021294 | 981 | Down | 0 |
| Bra021473 | 1074 | Down | 1.06E-07 |
| Bra002157 | 1359 | Down | 3.97E-05 |
| Bra024021 | 5403 | Down | 1.75E-09 |
| Bra022196 | 630 | Down | 0.00024914 |
| Bra009930 | 3240 | Down | 6.22E-09 |
| Bra036003 | 1710 | Down | 0.00055778 |
| Bra034904 | 717 | Down | 2.20E-05 |
| Bra032062 | 549 | Down | 0.00031015 |
| Bra038729 | 1833 | Down | 4.94E-12 |
| Bra007705 | 1482 | Down | 1.43E-07 |
| Bra024547 | 720 | Down | 6.10E-11 |
| Bra000193 | 390 | Down | 7.72E-05 |
| Bra019846 | 900 | Down | 3.77E-06 |
| Bra035686 | 3144 | Down | 0 |
| Bra039703 | 408 | Down | 1.43E-11 |
| Bra038825 | 1302 | Down | 2.87E-11 |
| Bra015038 | 822 | Down | 9.84E-12 |
| Bra010759 | 2586 | Down | 0.00048124 |
| Bra019329 | 1974 | Down | 8.97E-12 |
| Bra019804 | 1833 | Down | 0.00012021 |
| Bra029167 | 372 | Down | 0 |
| Bra010044 | 2751 | Down | 8.84E-12 |
| Bra009770 | 1014 | Down | 1.81E-06 |
| Bra006067 | 2088 | Down | 1.38E-12 |
| Bra031940 | 1050 | Down | 5.72E-12 |
| Bra020606 | 1659 | Down | 9.18E-06 |
| Bra036728 | 2448 | Down | 1.43E-08 |
| Bra006272 | 549 | Down | 2.89E-12 |
| Bra024736 | 1119 | Up | 0 |
| Bra020470 | 807 | Up | 0 |
| Bra020631 | 930 | Up | 1.32E-218 |
| Bra008531 | 453 | Up | 3.65E-55 |
| Bra021220 | 465 | Up | 2.34E-46 |
| Bra032569 | 1026 | Up | 3.90E-103 |
| Bra026845 | 975 | Up | 6.70E-98 |
| Bra010794 | 393 | Up | 9.71E-37 |
| Bra030162 | 357 | Up | 1.36E-23 |
| Bra014486 | 1245 | Up | 4.56E-81 |
| Bra002528 | 483 | Up | 6.94E-30 |
| Bra011529 | 789 | Up | 7.85E-49 |
| Bra013009 | 942 | Up | 1.95E-55 |
| Bra026843 | 876 | Up | 9.86E-48 |
| Bra022321 | 741 | Up | 9.70E-37 |
| Bra034624 | 510 | Up | 9.01E-23 |
| Bra019035 | 189 | Up | 2.88E-08 |
| Bra031515 | 1671 | Up | 3.57E-74 |
| Bra003336 | 1140 | Up | 6.27E-50 |
| Bra030674 | 480 | Up | 1.36E-20 |
| Bra009466 | 1266 | Up | 1.64E-53 |
| Bra019087 | 450 | Up | 2.04E-18 |
| Bra008532 | 780 | Up | 4.51E-32 |
| Bra030240 | 246 | Up | 7.36E-10 |
| Bra012760 | 366 | Up | 4.58E-14 |
| Bra031046 | 627 | Up | 3.88E-24 |
| Bra005262 | 1149 | Up | 6.94E-44 |
| Bra010096 | 942 | Up | 2.30E-35 |
| Bra005601 | 840 | Up | 5.64E-31 |
| Bra002734 | 507 | Up | 3.83E-18 |
| Bra000299 | 219 | Up | 9.67E-08 |
| Bra011216 | 993 | Up | 1.53E-34 |
| Bra031554 | 375 | Up | 1.86E-12 |
| Bra030920 | 369 | Up | 3.37E-12 |
| Bra024539 | 408 | Up | 2.98E-13 |
| Bra024963 | 288 | Up | 2.51E-09 |
| Bra040318 | 393 | Up | 3.37E-12 |
| Bra031043 | 606 | Up | 3.83E-18 |
| Bra016306 | 804 | Up | 3.88E-24 |
| Bra017656 | 813 | Up | 1.36E-23 |
| Bra039700 | 381 | Up | 6.46E-11 |
| Bra040667 | 960 | Up | 1.07E-27 |
| Bra006401 | 612 | Up | 2.50E-17 |
| Bra008007 | 336 | Up | 4.61E-09 |
| Bra017974 | 261 | Up | 1.08E-06 |
| Bra028352 | 252 | Up | 1.98E-06 |
| Bra014534 | 258 | Up | 1.98E-06 |
| Bra015361 | 2379 | Up | 4.08E-60 |
| Bra027596 | 729 | Up | 4.68E-17 |
| Bra037695 | 747 | Up | 2.51E-17 |
| Bra039434 | 291 | Up | 1.08E-06 |
| Bra030197 | 258 | Up | 6.54E-06 |
| Bra028759 | 918 | Up | 1.36E-20 |
| Bra004175 | 1062 | Up | 4.81E-23 |
| Bra024336 | 273 | Up | 6.53E-06 |
| Bra026323 | 189 | Up | 0.00039786 |
| Bra018233 | 372 | Up | 9.67E-08 |
| Bra032362 | 402 | Up | 2.88E-08 |
| Bra029254 | 912 | Up | 1.10E-18 |
| Bra023225 | 342 | Up | 1.08E-06 |
| Bra009212 | 1362 | Up | 1.33E-26 |
| Bra032911 | 414 | Up | 5.29E-08 |
| Bra009658 | 264 | Up | 3.85E-05 |
| Bra030828 | 879 | Up | 8.76E-17 |
| Bra024653 | 321 | Up | 3.60E-06 |
| Bra025998 | 297 | Up | 1.18E-05 |
| Bra006905 | 330 | Up | 3.60E-06 |
| Bra013177 | 252 | Up | 0.0001247 |
| Bra008440 | 597 | Up | 1.18E-10 |
| Bra002718 | 645 | Up | 3.54E-11 |
| Bra023742 | 846 | Up | 1.30E-14 |
| Bra031021 | 1734 | Up | 6.94E-30 |
| Bra033146 | 1122 | Up | 5.88E-19 |
| Bra014981 | 1431 | Up | 7.29E-24 |
| Bra026911 | 399 | Up | 1.98E-06 |
| Bra031255 | 666 | Up | 1.19E-10 |
| Bra038677 | 585 | Up | 2.51E-09 |
| Bra019920 | 1710 | Up | 2.02E-27 |
| Bra033324 | 990 | Up | 1.08E-15 |
| Bra027501 | 738 | Up | 6.46E-11 |
| Bra033407 | 582 | Up | 1.57E-08 |
| Bra027904 | 1494 | Up | 5.89E-22 |
| Bra007307 | 261 | Up | 0.00070822 |
| Bra016179 | 444 | Up | 3.60E-06 |
| Bra030773 | 1128 | Up | 3.78E-15 |
| Bra010625 | 774 | Up | 2.18E-10 |
| Bra006486 | 918 | Up | 3.37E-12 |
| Bra026170 | 438 | Up | 1.18E-05 |
| Bra007503 | 1572 | Up | 8.95E-20 |
| Bra007491 | 321 | Up | 0.00039827 |
| Bra005739 | 453 | Up | 1.18E-05 |
| Bra040920 | 1638 | Up | 4.77E-20 |
| Bra040999 | 759 | Up | 8.51E-09 |
| Bra035717 | 324 | Up | 0.00070924 |
| Bra003947 | 663 | Up | 1.76E-07 |
| Bra009055 | 621 | Up | 5.89E-07 |
| Bra029548 | 513 | Up | 1.18E-05 |
| Bra037694 | 369 | Up | 0.000398 |
| Bra014316 | 477 | Up | 3.84E-05 |
| Bra008741 | 750 | Up | 9.67E-08 |
| Bra028639 | 519 | Up | 2.14E-05 |
| Bra010881 | 555 | Up | 1.18E-05 |
| Bra009316 | 1119 | Up | 3.54E-11 |
| Bra010304 | 1053 | Up | 2.18E-10 |
| Bra022239 | 1176 | Up | 1.93E-11 |
| Bra013569 | 822 | Up | 5.29E-08 |
| Bra032566 | 2259 | Up | 1.10E-21 |
| Bra016136 | 552 | Up | 3.85E-05 |
| Bra017243 | 2274 | Up | 1.36E-20 |
| Bra030205 | 768 | Up | 5.89E-07 |
| Bra002329 | 564 | Up | 3.85E-05 |
| Bra037251 | 423 | Up | 0.00070935 |
| Bra029385 | 1482 | Up | 5.54E-13 |
| Bra021669 | 582 | Up | 3.85E-05 |
| Bra011077 | 444 | Up | 0.00070901 |
| Bra030477 | 552 | Up | 0.00012449 |
| Bra032232 | 1176 | Up | 1.36E-09 |
| Bra033690 | 660 | Up | 2.14E-05 |
| Bra017778 | 2688 | Up | 2.06E-21 |
| Bra031486 | 1011 | Up | 5.29E-08 |
| Bra036013 | 477 | Up | 0.00070969 |
| Bra014336 | 834 | Up | 1.98E-06 |
| Bra020494 | 1422 | Up | 1.18E-10 |
| Bra009113 | 615 | Up | 0.00012456 |
| Bra003714 | 633 | Up | 0.00012442 |
| Bra001517 | 1275 | Up | 8.50E-09 |
| Bra038174 | 852 | Up | 1.18E-05 |
| Bra002443 | 690 | Up | 0.00012444 |
| Bra017699 | 1101 | Up | 3.21E-07 |
| Bra003682 | 1065 | Up | 5.88E-07 |
| Bra016882 | 909 | Up | 6.53E-06 |
| Bra010839 | 786 | Up | 3.85E-05 |
| Bra013700 | 1002 | Up | 1.98E-06 |
| Bra018051 | 1521 | Up | 1.36E-09 |
| Bra021124 | 768 | Up | 6.94E-05 |
| Bra009053 | 1071 | Up | 1.08E-06 |
| Bra035306 | 1314 | Up | 5.29E-08 |
| Bra017813 | 798 | Up | 6.93E-05 |
| Bra011776 | 1125 | Up | 1.08E-06 |
| Bra026226 | 765 | Up | 0.00012437 |
| Bra012510 | 723 | Up | 0.00022273 |
| Bra001611 | 1086 | Up | 1.98E-06 |
| Bra013922 | 864 | Up | 3.85E-05 |
| Bra032574 | 2283 | Up | 2.97E-13 |
| Bra039804 | 792 | Up | 0.00012468 |
| Bra027546 | 1005 | Up | 1.18E-05 |
| Bra007697 | 936 | Up | 3.85E-05 |
| Bra026606 | 1050 | Up | 1.18E-05 |
| Bra011739 | 1065 | Up | 1.18E-05 |
| Bra016823 | 951 | Up | 6.93E-05 |
| Bra036158 | 900 | Up | 0.00012465 |
| Bra006461 | 1218 | Up | 3.60E-06 |
| Bra002546 | 1275 | Up | 1.98E-06 |
| Bra008806 | 864 | Up | 0.00022261 |
| Bra011123 | 993 | Up | 6.94E-05 |
| Bra014109 | 792 | Up | 0.00070946 |
| Bra005936 | 792 | Up | 0.00070867 |
| Bra009215 | 1134 | Up | 2.14E-05 |
| Bra025164 | 3189 | Up | 1.30E-14 |
| Bra005309 | 1164 | Up | 2.14E-05 |
| Bra010802 | 1002 | Up | 0.0001244 |
| Bra035549 | 1125 | Up | 3.85E-05 |
| Bra026163 | 1191 | Up | 2.13E-05 |
| Bra017253 | 1020 | Up | 0.00012458 |
| Bra035814 | 1509 | Up | 1.08E-06 |
| Bra030525 | 1347 | Up | 6.53E-06 |
| Bra000195 | 1005 | Up | 0.00022265 |
| Bra000568 | 1788 | Up | 1.76E-07 |
| Bra010768 | 900 | Up | 0.00070856 |
| Bra007725 | 1149 | Up | 0.00012463 |
| Bra034443 | 972 | Up | 0.00070912 |
| Bra039508 | 987 | Up | 0.00070811 |
| Bra027079 | 1002 | Up | 0.0007098 |
| Bra023539 | 1194 | Up | 0.00022277 |
| Bra029356 | 1047 | Up | 0.00070957 |
| Bra000870 | 1143 | Up | 0.00039807 |
| Bra006494 | 1236 | Up | 0.00022257 |
| Bra019469 | 2019 | Up | 5.88E-07 |
| Bra017640 | 1476 | Up | 3.85E-05 |
| Bra012042 | 1719 | Up | 6.54E-06 |
| Bra035809 | 1758 | Up | 6.53E-06 |
| Bra022372 | 1377 | Up | 0.00012454 |
| Bra021298 | 1632 | Up | 2.14E-05 |
| Bra035033 | 1809 | Up | 6.53E-06 |
| Bra010316 | 1821 | Up | 6.53E-06 |
| Bra029840 | 1326 | Up | 0.00039813 |
| Bra027791 | 2712 | Up | 5.29E-08 |
| Bra021491 | 1482 | Up | 0.00022281 |
| Bra029717 | 2157 | Up | 3.60E-06 |
| Bra022036 | 4509 | Up | 1.02E-12 |
| Bra038467 | 1770 | Up | 0.00012461 |
| Bra021152 | 1869 | Up | 0.00012447 |
| Bra009863 | 2040 | Up | 6.93E-05 |
| Bra016844 | 2469 | Up | 2.14E-05 |
| Bra031511 | 1857 | Up | 0.0003982 |
| Bra002009 | 1866 | Up | 0.00070878 |
| Bra019754 | 2673 | Up | 3.85E-05 |
| Bra003863 | 3603 | Up | 1.98E-06 |
| Bra019752 | 2583 | Up | 0.00012451 |
| Bra026561 | 2184 | Up | 0.00070833 |
| Bra017617 | 2301 | Up | 0.00070845 |
| Bra001787 | 2793 | Up | 0.00022269 |
| Bra004187 | 2649 | Up | 0.00039793 |
| Bra012689 | 3885 | Up | 0.0007089 |
| Bra036642 | 660 | Up | 8.56E-160 |
| Bra024670 | 9486 | Up | 0.00022285 |
| Bra009294 | 1323 | Up | 1.24E-75 |
| Bra030404 | 423 | Up | 5.70E-71 |
| Bra040582 | 1638 | Up | 2.15E-64 |
| Bra015882 | 564 | Up | 1.78E-62 |
| Bra032570 | 2091 | Up | 1.45E-117 |
| Bra016002 | 645 | Up | 6.47E-56 |
| Bra021222 | 510 | Up | 1.82E-90 |
| Bra009464 | 687 | Up | 7.81E-37 |
| Bra012151 | 1428 | Up | 2.71E-36 |
| Bra003780 | 585 | Up | 7.22E-31 |
| Bra031065 | 822 | Up | 2.31E-239 |
| Bra018344 | 1005 | Up | 1.61E-29 |
| Bra029319 | 672 | Up | 2.98E-29 |
| Bra033077 | 561 | Up | 2.77E-58 |
| Bra021965 | 1320 | Up | 0 |
| Bra025083 | 1413 | Up | 2.77E-26 |
| Bra001453 | 729 | Up | 5.87E-51 |
| Bra016763 | 609 | Up | 3.33E-25 |
| Bra013863 | 639 | Up | 0 |
| Bra006403 | 624 | Up | 7.38E-24 |
| Bra012816 | 1311 | Up | 4.73E-23 |
| Bra004683 | 429 | Up | 5.55E-22 |
| Bra003588 | 894 | Up | 3.77E-40 |
| Bra006843 | 666 | Up | 2.64E-19 |
| Bra037630 | 708 | Up | 4.89E-19 |
| Bra030673 | 396 | Up | 5.64E-18 |
| Bra026326 | 732 | Up | 5.64E-18 |
| Bra037811 | 438 | Up | 9.08E-35 |
| Bra008037 | 2760 | Up | 8.32E-150 |
| Bra029145 | 1524 | Up | 2.24E-16 |
| Bra034717 | 528 | Up | 1.19E-48 |
| Bra008267 | 639 | Up | 2.36E-32 |
| Bra004633 | 1461 | Up | 6.27E-228 |
| Bra010291 | 849 | Up | 7.63E-16 |
| Bra033482 | 321 | Up | 1.75E-30 |
| Bra002719 | 852 | Up | 3.13E-230 |
| Bra005843 | 1527 | Up | 3.24E-30 |
| Bra032607 | 642 | Up | 4.76E-15 |
| Bra014213 | 1143 | Up | 4.76E-15 |
| Bra028290 | 663 | Up | 5.97E-30 |
| Bra024954 | 657 | Up | 8.73E-15 |
| Bra039120 | 1923 | Up | 6.09E-73 |
| Bra023044 | 1230 | Up | 5.37E-101 |
| Bra026963 | 618 | Up | 2.96E-14 |
| Bra007953 | 495 | Up | 1.28E-28 |
| Bra029349 | 843 | Up | 5.45E-14 |
| Bra001907 | 861 | Up | 6.01E-42 |
| Bra031012 | 1116 | Up | 1.00E-13 |
| Bra040159 | 723 | Up | 2.70E-55 |
| Bra032595 | 1623 | Up | 1.84E-13 |
| Bra006925 | 1308 | Up | 5.10E-27 |
| Bra034556 | 2466 | Up | 2.32E-161 |
| Bra014929 | 858 | Up | 5.73E-66 |
| Bra004994 | 1257 | Up | 1.26E-64 |
| Bra027602 | 1401 | Up | 1.29E-111 |
| Bra006089 | 402 | Up | 6.19E-12 |
| Bra041096 | 2016 | Up | 1.09E-11 |
| Bra008435 | 873 | Up | 1.49E-208 |
| Bra018524 | 1494 | Up | 6.22E-11 |
| Bra022981 | 342 | Up | 6.22E-11 |
| Bra040973 | 819 | Up | 6.22E-11 |
| Bra028291 | 714 | Up | 1.02E-21 |
| Bra002283 | 534 | Up | 1.49E-43 |
| Bra017278 | 1239 | Up | 5.38E-98 |
| Bra000845 | 1266 | Up | 1.88E-21 |
| Bra039980 | 678 | Up | 1.12E-10 |
| Bra031195 | 1035 | Up | 2.69E-31 |
| Bra013702 | 1086 | Up | 2.69E-31 |
| Bra019162 | 645 | Up | 2.11E-20 |
| Bra020036 | 906 | Up | 1.67E-30 |
| Bra012006 | 1494 | Up | 3.64E-10 |
| Bra002400 | 846 | Up | 6.35E-81 |
| Bra026540 | 957 | Up | 5.86E-289 |
| Bra000423 | 846 | Up | 6.52E-10 |
| Bra016602 | 2760 | Up | 8.87E-59 |
| Bra006146 | 3369 | Up | 5.31E-78 |
| Bra012149 | 891 | Up | 2.78E-172 |
| Bra000850 | 597 | Up | 1.45E-18 |
| Bra027096 | 1065 | Up | 7.25E-56 |
| Bra027902 | 1389 | Up | 2.13E-09 |
| Bra028186 | 408 | Up | 3.44E-37 |
| Bra035148 | 903 | Up | 4.69E-102 |
| Bra000455 | 819 | Up | 1.72E-73 |
| Bra000903 | 1563 | Up | 3.82E-09 |
| Bra014760 | 693 | Up | 3.82E-09 |
| Bra013630 | 483 | Up | 3.82E-09 |
| Bra005891 | 708 | Up | 2.00E-107 |
| Bra016233 | 2409 | Up | 8.82E-18 |
| Bra008498 | 741 | Up | 1.48E-26 |
| Bra029712 | 1359 | Up | 0 |
| Bra039194 | 2325 | Up | 8.11E-35 |
| Bra034677 | 1107 | Up | 6.86E-157 |
| Bra009626 | 1947 | Up | 1.40E-128 |
| Bra017160 | 2373 | Up | 4.98E-34 |
| Bra001454 | 1416 | Up | 3.06E-25 |
| Bra032293 | 2136 | Up | 1.04E-83 |
| Bra000985 | 426 | Up | 1.78E-48 |
| Bra033350 | 297 | Up | 2.02E-47 |
| Bra021979 | 2580 | Up | 2.99E-110 |
| Bra039937 | 417 | Up | 2.11E-31 |
| Bra009460 | 1179 | Up | 7.13E-08 |
| Bra007293 | 1110 | Up | 7.13E-08 |
| Bra039316 | 618 | Up | 1.98E-15 |
| Bra003114 | 420 | Up | 7.13E-08 |
| Bra005831 | 963 | Up | 3.60E-15 |
| Bra035195 | 633 | Up | 3.60E-15 |
| Bra007240 | 672 | Up | 1.26E-22 |
| Bra016234 | 1953 | Up | 1.27E-07 |
| Bra007195 | 1278 | Up | 2.18E-171 |
| Bra013957 | 1494 | Up | 1.18E-14 |
| Bra012803 | 1272 | Up | 7.54E-22 |
| Bra027357 | 1122 | Up | 2.25E-07 |
| Bra028633 | 348 | Up | 2.13E-14 |
| Bra033436 | 1029 | Up | 6.78E-65 |
| Bra032383 | 1587 | Up | 3.99E-07 |
| Bra029990 | 885 | Up | 1.30E-40 |
| Bra000806 | 429 | Up | 2.72E-20 |
| Bra020433 | 783 | Up | 0 |
| Bra008438 | 996 | Up | 7.14E-07 |
| Bra018448 | 2913 | Up | 7.14E-07 |
| Bra021754 | 2865 | Up | 7.14E-07 |
| Bra038411 | 966 | Up | 2.31E-13 |
| Bra034099 | 456 | Up | 7.14E-07 |
| Bra033773 | 543 | Up | 3.43E-26 |
| Bra022347 | 1275 | Up | 1.63E-19 |
| Bra007964 | 1485 | Up | 1.27E-06 |
| Bra027981 | 555 | Up | 4.32E-32 |
| Bra029355 | 1443 | Up | 5.85E-45 |
| Bra011686 | 375 | Up | 2.96E-19 |
| Bra038796 | 750 | Up | 1.28E-06 |
| Bra009872 | 1047 | Up | 7.81E-32 |
| Bra031263 | 1662 | Up | 5.36E-19 |
| Bra032665 | 480 | Up | 9.68E-19 |
| Bra000544 | 915 | Up | 6.06E-80 |
| Bra032988 | 498 | Up | 4.05E-24 |
| Bra022176 | 729 | Up | 3.97E-06 |
| Bra021824 | 2664 | Up | 3.97E-06 |
| Bra038521 | 399 | Up | 2.41E-23 |
| Bra007039 | 1005 | Up | 1.86E-17 |
| Bra002962 | 1413 | Up | 3.37E-17 |
| Bra040158 | 801 | Up | 9.15E-40 |
| Bra026979 | 2745 | Up | 6.97E-06 |
| Bra010318 | 1656 | Up | 6.97E-06 |
| Bra001067 | 1107 | Up | 2.15E-11 |
| Bra029100 | 291 | Up | 6.97E-06 |
| Bra014297 | 603 | Up | 1.33E-61 |
| Bra040169 | 642 | Up | 2.57E-22 |
| Bra001752 | 804 | Up | 6.28E-27 |
| Bra032790 | 237 | Up | 1.22E-05 |
| Bra017040 | 1266 | Up | 3.34E-102 |
| Bra013449 | 1518 | Up | 7.79E-43 |
| Bra011180 | 849 | Up | 1.03E-47 |
| Bra026562 | 3990 | Up | 2.05E-26 |
| Bra021208 | 2004 | Up | 1.88E-47 |
| Bra018941 | 1302 | Up | 1.22E-199 |
| Bra008316 | 1128 | Up | 3.39E-47 |
| Bra012364 | 480 | Up | 2.14E-05 |
| Bra003164 | 771 | Up | 2.14E-05 |
| Bra012114 | 2184 | Up | 2.14E-05 |
| Bra039037 | 318 | Up | 2.13E-05 |
| Bra024662 | 873 | Up | 1.15E-183 |
| Bra017350 | 762 | Up | 2.20E-25 |
| Bra032309 | 2115 | Up | 1.39E-80 |
| Bra024126 | 366 | Up | 6.72E-15 |
| Bra025713 | 765 | Up | 1.27E-44 |
| Bra031927 | 915 | Up | 3.32E-113 |
| Bra039646 | 1332 | Up | 3.72E-05 |
| Bra004324 | 1194 | Up | 3.72E-05 |
| Bra017893 | 495 | Up | 3.72E-05 |
| Bra008473 | 495 | Up | 3.13E-29 |
| Bra021393 | 972 | Up | 1.20E-14 |
| Bra011135 | 183 | Up | 4.25E-34 |
| Bra031069 | 978 | Up | 3.73E-05 |
| Bra007887 | 828 | Up | 3.72E-05 |
| Bra019777 | 621 | Up | 1.20E-14 |
| Bra018553 | 2004 | Up | 4.24E-34 |
| Bra016400 | 633 | Up | 4.15E-24 |
| Bra011687 | 369 | Up | 1.17E-09 |
| Bra006741 | 870 | Up | 1.17E-09 |
| Bra007334 | 1626 | Up | 3.21E-206 |
| Bra014665 | 702 | Up | 6.97E-14 |
| Bra036138 | 1062 | Up | 6.48E-05 |
| Bra006304 | 1422 | Up | 6.47E-05 |
| Bra026509 | 981 | Up | 2.07E-09 |
| Bra013584 | 1617 | Up | 6.47E-05 |
| Bra026732 | 2265 | Up | 2.07E-09 |
| Bra031755 | 924 | Up | 6.47E-05 |
| Bra010649 | 1089 | Up | 6.47E-05 |
| Bra005572 | 216 | Up | 6.47E-05 |
| Bra000548 | 1320 | Up | 6.47E-05 |
| Bra027763 | 3900 | Up | 4.74E-32 |
| Bra022345 | 651 | Up | 5.55E-18 |
| Bra032936 | 540 | Up | 3.65E-09 |
| Bra038219 | 795 | Up | 1.20E-35 |
| Bra034774 | 981 | Up | 1.11E-26 |
| Bra005104 | 1374 | Up | 8.59E-98 |
| Bra017839 | 1140 | Up | 2.25E-44 |
| Bra014696 | 681 | Up | 9.94E-18 |
| Bra004532 | 1461 | Up | 8.93E-31 |
| Bra032845 | 690 | Up | 1.13E-74 |
| Bra016495 | 1866 | Up | 2.67E-144 |
| Bra025892 | 693 | Up | 0.00011219 |
| Bra013211 | 456 | Up | 0.00011215 |
| Bra025418 | 519 | Up | 0.00011213 |
| Bra020011 | 891 | Up | 0.00011222 |
| Bra028874 | 2613 | Up | 0.00011217 |
| Bra010697 | 687 | Up | 7.03E-13 |
| Bra022770 | 645 | Up | 1.14E-08 |
| Bra006210 | 1416 | Up | 5.69E-17 |
| Bra011181 | 849 | Up | 1.14E-08 |
| Bra029392 | 1557 | Up | 1.31E-33 |
| Bra036570 | 666 | Up | 1.02E-16 |
| Bra018405 | 789 | Up | 3.79E-54 |
| Bra010880 | 519 | Up | 5.95E-75 |
| Bra027740 | 231 | Up | 1.44E-41 |
| Bra001815 | 693 | Up | 2.16E-12 |
| Bra007339 | 555 | Up | 0.0001928 |
| Bra007263 | 1671 | Up | 0.00019284 |
| Bra023294 | 234 | Up | 1.30E-116 |
| Bra007696 | 1371 | Up | 6.29E-64 |
| Bra000064 | 1422 | Up | 0 |
| Bra036295 | 642 | Up | 1.22E-23 |
| Bra001160 | 3135 | Up | 6.34E-12 |
| Bra016564 | 525 | Up | 4.96E-39 |
| Bra031931 | 1110 | Up | 3.09E-27 |
| Bra030216 | 1509 | Up | 8.87E-39 |
| Bra035640 | 1557 | Up | 0.00033129 |
| Bra000170 | 396 | Up | 6.19E-08 |
| Bra025953 | 846 | Up | 0.00033124 |
| Bra021502 | 645 | Up | 0.00033118 |
| Bra031884 | 600 | Up | 0.00033112 |
| Bra005552 | 1752 | Up | 1.08E-11 |
| Bra029740 | 1221 | Up | 6.19E-08 |
| Bra019538 | 1455 | Up | 0.00033141 |
| Bra010397 | 204 | Up | 1.08E-11 |
| Bra027047 | 774 | Up | 0.00033135 |
| Bra009630 | 1023 | Up | 0.00033107 |
| Bra024088 | 849 | Up | 1.04E-72 |
| Bra032795 | 930 | Up | 6.95E-23 |
| Bra015727 | 546 | Up | 4.60E-121 |
| Bra023614 | 552 | Up | 1.08E-07 |
| Bra000391 | 1539 | Up | 3.65E-52 |
| Bra009882 | 2466 | Up | 1.03E-44 |
| Bra025764 | 1146 | Up | 1.57E-188 |
| Bra006556 | 3708 | Up | 9.29E-37 |
| Bra004841 | 1590 | Up | 2.13E-95 |
| Bra009817 | 1491 | Up | 2.28E-76 |
| Bra025816 | 2196 | Up | 4.56E-29 |
| Bra022603 | 867 | Up | 4.20E-58 |
| Bra004522 | 1407 | Up | 1.87E-43 |
| Bra016860 | 735 | Up | 1.74E-97 |
| Bra034711 | 1818 | Up | 0.00056682 |
| Bra031621 | 1014 | Up | 1.24E-21 |
| Bra031820 | 336 | Up | 0.000567 |
| Bra037542 | 183 | Up | 0.00056691 |
| Bra024890 | 651 | Up | 0.00056654 |
| Bra005957 | 573 | Up | 0.00056709 |
| Bra020152 | 2721 | Up | 1.87E-07 |
| Bra034392 | 951 | Up | 0.00056672 |
| Bra004167 | 1491 | Up | 0.00056663 |
| Bra032485 | 2436 | Up | 5.68E-25 |
| Bra039099 | 363 | Up | 8.57E-46 |
| Bra029056 | 1425 | Up | 7.61E-74 |
| Bra016056 | 825 | Up | 2.41E-38 |
| Bra017086 | 1464 | Up | 3.26E-07 |
| Bra003448 | 1275 | Up | 5.81E-14 |
| Bra008033 | 822 | Up | 1.02E-144 |
| Bra011650 | 954 | Up | 2.58E-27 |
| Bra027519 | 294 | Up | 2.18E-20 |
| Bra020313 | 1170 | Up | 1.78E-50 |
| Bra022772 | 1128 | Up | 6.92E-131 |
| Bra022847 | 363 | Up | 0.00096024 |
| Bra027775 | 3078 | Up | 0.00096009 |
| Bra024213 | 591 | Up | 0.00095979 |
| Bra040848 | 588 | Up | 0.00096038 |
| Bra020179 | 813 | Up | 2.54E-70 |
| Bra024795 | 690 | Up | 5.69E-07 |
| Bra015876 | 828 | Up | 0.00095994 |
| Bra005800 | 1482 | Up | 0.00095965 |
| Bra036322 | 681 | Up | 0.0009595 |
| Bra007444 | 1209 | Up | 4.93E-127 |
| Bra037470 | 660 | Up | 6.17E-40 |
| Bra015912 | 831 | Up | 1.48E-16 |
| Bra014762 | 843 | Up | 1.71E-32 |
| Bra027866 | 2790 | Up | 1.40E-165 |
| Bra029107 | 396 | Up | 8.11E-68 |
| Bra020192 | 519 | Up | 9.96E-07 |
| Bra009112 | 993 | Up | 2.17E-19 |
| Bra025917 | 630 | Up | 2.17E-19 |
| Bra010922 | 702 | Up | 1.62E-138 |
| Bra000508 | 1173 | Up | 1.23E-237 |
| Bra013475 | 1608 | Up | 1.17E-28 |
| Bra011299 | 954 | Up | 2.60E-82 |
| Bra000848 | 867 | Up | 9.84E-13 |
| Bra025951 | 5358 | Up | 8.22E-16 |
| Bra022437 | 903 | Up | 8.27E-128 |
| Bra027359 | 1131 | Up | 2.20E-277 |
| Bra009476 | 1635 | Up | 1.19E-18 |
| Bra010396 | 189 | Up | 1.61E-09 |
| Bra031691 | 696 | Up | 1.73E-06 |
| Bra016956 | 690 | Up | 4.26E-34 |
| Bra020564 | 1632 | Up | 1.45E-15 |
| Bra018138 | 261 | Up | 1.73E-06 |
| Bra006426 | 2463 | Up | 2.80E-09 |
| Bra007888 | 1182 | Up | 4.51E-15 |
| Bra028907 | 2097 | Up | 6.85E-42 |
| Bra023297 | 4524 | Up | 2.98E-06 |
| Bra003712 | 540 | Up | 2.67E-38 |
| Bra034592 | 1158 | Up | 0 |
| Bra001639 | 837 | Up | 1.97E-26 |
| Bra005271 | 3459 | Up | 4.86E-09 |
| Bra005027 | 1149 | Up | 8.33E-12 |
| Bra029880 | 294 | Up | 1.39E-14 |
| Bra024638 | 1155 | Up | 2.02E-17 |
| Bra000586 | 1944 | Up | 4.31E-23 |
| Bra013815 | 948 | Up | 1.21E-68 |
| Bra005860 | 1119 | Up | 5.14E-06 |
| Bra003778 | 669 | Up | 5.18E-20 |
| Bra025325 | 1092 | Up | 1.51E-107 |
| Bra040998 | 1887 | Up | 2.34E-22 |
| Bra016561 | 645 | Up | 1.37E-117 |
| Bra027651 | 702 | Up | 1.93E-16 |
| Bra012152 | 1497 | Up | 1.85E-24 |
| Bra031924 | 393 | Up | 1.33E-13 |
| Bra014643 | 852 | Up | 2.55E-08 |
| Bra007947 | 477 | Up | 0 |
| Bra014427 | 207 | Up | 5.96E-16 |
| Bra040633 | 1056 | Up | 2.11E-55 |
| Bra023481 | 1104 | Up | 1.04E-36 |
| Bra016250 | 705 | Up | 1.24E-10 |
| Bra033504 | 291 | Up | 1.50E-05 |
| Bra024380 | 1422 | Up | 4.40E-08 |
| Bra008038 | 1344 | Up | 1.98E-28 |
| Bra024175 | 930 | Up | 4.65E-18 |
| Bra008856 | 1065 | Up | 1.83E-15 |
| Bra017117 | 1392 | Up | 2.09E-145 |
| Bra002773 | 1524 | Up | 7.57E-08 |
| Bra005688 | 861 | Up | 1.21E-12 |
| Bra010099 | 1521 | Up | 1.61E-59 |
| Bra037743 | 834 | Up | 2.54E-05 |
| Bra016319 | 351 | Up | 2.54E-05 |
| Bra022115 | 690 | Up | 1.11E-43 |
| Bra013732 | 951 | Up | 1.15E-203 |
| Bra023998 | 987 | Up | 9.98E-55 |
| Bra019265 | 972 | Up | 5.38E-38 |
| Bra031663 | 1011 | Up | 5.97E-19 |
| Bra023022 | 1317 | Up | 5.98E-19 |
| Bra014850 | 906 | Up | 1.10E-09 |
| Bra006006 | 2940 | Up | 4.32E-05 |
| Bra012026 | 714 | Up | 2.30E-16 |
| Bra014396 | 1380 | Up | 4.49E-32 |
| Bra032951 | 876 | Up | 3.78E-07 |
| Bra038088 | 1497 | Up | 3.78E-07 |
| Bra028302 | 1560 | Up | 2.23E-27 |
| Bra000247 | 2184 | Up | 1.12E-22 |
| Bra030507 | 1941 | Up | 1.10E-69 |
| Bra012479 | 867 | Up | 7.29E-05 |
| Bra015513 | 1092 | Up | 3.24E-09 |
| Bra040642 | 1161 | Up | 2.80E-11 |
| Bra007205 | 1638 | Up | 0 |
| Bra024335 | 327 | Up | 6.48E-07 |
| Bra003044 | 453 | Up | 2.11E-15 |
| Bra007546 | 945 | Up | 1.65E-17 |
| Bra022436 | 804 | Up | 4.54E-39 |
| Bra004390 | 933 | Up | 5.54E-52 |
| Bra007826 | 1800 | Up | 2.26E-19 |
| Bra024611 | 912 | Up | 1.31E-80 |
| Bra029869 | 597 | Up | 9.51E-09 |
| Bra012719 | 624 | Up | 0.00012298 |
| Bra000963 | 2919 | Up | 6.33E-15 |
| Bra000046 | 885 | Up | 0.00012303 |
| Bra032377 | 474 | Up | 3.92E-19 |
| Bra007581 | 564 | Up | 6.32E-15 |
| Bra007813 | 1647 | Up | 0.000123 |
| Bra002559 | 1194 | Up | 2.04E-75 |
| Bra013275 | 1884 | Up | 4.71E-50 |
| Bra015296 | 1590 | Up | 4.41E-150 |
| Bra014080 | 945 | Up | 1.38E-10 |
| Bra010032 | 1200 | Up | 4.71E-31 |
| Bra029235 | 1107 | Up | 2.95E-39 |
| Bra020368 | 1437 | Up | 2.05E-59 |
| Bra038089 | 771 | Up | 2.02E-18 |
| Bra031266 | 1659 | Up | 1.89E-06 |
| Bra014426 | 834 | Up | 2.28E-12 |
| Bra030917 | 1116 | Up | 2.01E-18 |
| Bra031772 | 1032 | Up | 1.89E-06 |
| Bra002984 | 396 | Up | 7.87E-129 |
| Bra025995 | 648 | Up | 0.00020543 |
| Bra031048 | 1149 | Up | 5.79E-73 |
| Bra002206 | 1698 | Up | 6.33E-12 |
| Bra023560 | 873 | Up | 6.33E-12 |
| Bra029456 | 510 | Up | 7.16E-138 |
| Bra008066 | 879 | Up | 1.09E-106 |
| Bra016254 | 1167 | Up | 4.73E-08 |
| Bra032591 | 2154 | Up | 1.45E-119 |
| Bra024318 | 1260 | Up | 1.23E-99 |
| Bra007841 | 735 | Up | 1.54E-93 |
| Bra025450 | 1029 | Up | 0 |
| Bra019961 | 513 | Up | 1.75E-11 |
| Bra032419 | 996 | Up | 0.00034264 |
| Bra001905 | 312 | Up | 8.02E-08 |
| Bra039277 | 198 | Up | 5.40E-06 |
| Bra017272 | 684 | Up | 5.40E-06 |
| Bra035505 | 240 | Up | 5.31E-17 |
| Bra011371 | 846 | Up | 0.00034259 |
| Bra032747 | 2481 | Up | 5.39E-06 |
| Bra020820 | 2034 | Up | 8.01E-08 |
| Bra020828 | 2019 | Up | 1.17E-09 |
| Bra040061 | 2067 | Up | 4.28E-26 |
| Bra006274 | 1506 | Up | 6.23E-61 |
| Bra017301 | 1485 | Up | 2.96E-11 |
| Bra018969 | 1587 | Up | 2.96E-11 |
| Bra027907 | 1167 | Up | 2.14E-18 |
| Bra036269 | 1692 | Up | 8.38E-13 |
| Bra000140 | 909 | Up | 8.39E-13 |
| Bra030246 | 1632 | Up | 9.04E-06 |
| Bra017700 | 1104 | Up | 9.04E-06 |
| Bra027440 | 834 | Up | 9.04E-06 |
| Bra033423 | 384 | Up | 0.00056894 |
| Bra000282 | 819 | Up | 0.00056857 |
| Bra002966 | 579 | Up | 0.00056885 |
| Bra032249 | 468 | Up | 0.00056866 |
| Bra003103 | 876 | Up | 0.00056875 |
| Bra012008 | 1386 | Up | 6.76E-123 |
| Bra037487 | 2436 | Up | 2.27E-07 |
| Bra037076 | 3036 | Up | 0.00056848 |
| Bra003840 | 1521 | Up | 6.29E-86 |
| Bra018346 | 1068 | Up | 1.13E-78 |
| Bra012153 | 1323 | Up | 1.92E-24 |
| Bra021063 | 1593 | Up | 4.85E-95 |
| Bra022030 | 213 | Up | 1.51E-05 |
| Bra034424 | 474 | Up | 3.93E-12 |
| Bra022100 | 672 | Up | 3.93E-12 |
| Bra028983 | 2031 | Up | 3.93E-12 |
| Bra007092 | 756 | Up | 3.83E-07 |
| Bra024284 | 375 | Up | 5.76E-56 |
| Bra016191 | 1287 | Up | 1.38E-79 |
| Bra039217 | 819 | Up | 2.09E-118 |
| Bra023967 | 546 | Up | 1.49E-185 |
| Bra009214 | 999 | Up | 2.87E-30 |
| Bra035002 | 816 | Up | 4.96E-120 |
| Bra007678 | 393 | Up | 2.45E-137 |
| Bra035883 | 765 | Up | 1.06E-11 |
| Bra019036 | 207 | Up | 0.00093673 |
| Bra014848 | 909 | Up | 1.14E-24 |
| Bra017872 | 885 | Up | 1.06E-11 |
| Bra020316 | 726 | Up | 0.00093659 |
| Bra021698 | 228 | Up | 0.00093688 |
| Bra031387 | 1452 | Up | 1.06E-11 |
| Bra038681 | 453 | Up | 0.00093731 |
| Bra037806 | 960 | Up | 0.00093717 |
| Bra011063 | 552 | Up | 0.00093645 |
| Bra003490 | 3879 | Up | 8.81E-20 |
| Bra018579 | 837 | Up | 0.00093702 |
| Bra007236 | 1761 | Up | 2.51E-05 |
| Bra013679 | 2064 | Up | 2.51E-05 |
| Bra026262 | 900 | Up | 1.64E-08 |
| Bra033849 | 2892 | Up | 0.0009363 |
| Bra004026 | 987 | Up | 1.06E-11 |
| Bra036022 | 714 | Up | 9.61E-117 |
| Bra012571 | 1173 | Up | 4.11E-68 |
| Bra000775 | 501 | Up | 0 |
| Bra001774 | 1626 | Up | 1.75E-11 |
| Bra000093 | 585 | Up | 1.42E-22 |
| Bra034596 | 468 | Up | 6.79E-10 |
| Bra031735 | 1875 | Up | 2.76E-08 |
| Bra015986 | 525 | Up | 6.71E-32 |
| Bra031834 | 708 | Up | 2.76E-08 |
| Bra004389 | 933 | Up | 1.40E-86 |
| Bra035384 | 1215 | Up | 2.12E-201 |
| Bra020393 | 564 | Up | 4.11E-22 |
| Bra006498 | 459 | Up | 7.92E-16 |
| Bra031599 | 624 | Up | 4.18E-05 |
| Bra037094 | 948 | Up | 4.18E-05 |
| Bra021049 | 846 | Up | 4.18E-05 |
| Bra040704 | 1104 | Up | 2.62E-101 |
| Bra033200 | 639 | Up | 5.73E-14 |
| Bra036955 | 1248 | Up | 5.34E-17 |
| Bra004996 | 1440 | Up | 5.34E-17 |
| Bra009427 | 534 | Up | 1.83E-06 |
| Bra017146 | 798 | Up | 1.83E-06 |
| Bra000800 | 1950 | Up | 1.91E-24 |
| Bra025493 | 3387 | Up | 1.34E-57 |
| Bra022095 | 1965 | Up | 9.74E-14 |
| Bra003680 | 1137 | Up | 2.85E-167 |
| Bra027920 | 1572 | Up | 0 |
| Bra026188 | 1857 | Up | 1.64E-37 |
| Bra036048 | 801 | Up | 2.04E-28 |
| Bra034611 | 726 | Up | 3.02E-34 |
| Bra012624 | 1872 | Up | 1.10E-38 |
| Bra001612 | 1092 | Up | 3.25E-09 |
| Bra000526 | 771 | Up | 3.24E-09 |
| Bra005127 | 879 | Up | 6.93E-05 |
| Bra003635 | 1320 | Up | 6.93E-05 |
| Bra033070 | 507 | Up | 6.93E-05 |
| Bra009738 | 1551 | Up | 9.91E-21 |
| Bra034321 | 783 | Up | 6.16E-100 |
| Bra023063 | 1872 | Up | 3.04E-06 |
| Bra039426 | 1326 | Up | 3.04E-06 |
| Bra028569 | 1437 | Up | 4.22E-19 |
| Bra003110 | 2934 | Up | 4.22E-19 |
| Bra007828 | 1395 | Up | 1.29E-07 |
| Bra038517 | 948 | Up | 1.86E-143 |
| Bra000261 | 2394 | Up | 5.44E-09 |
| Bra036972 | 1125 | Up | 4.98E-88 |
| Bra023739 | 1812 | Up | 2.85E-20 |
| Bra000158 | 1275 | Up | 2.44E-149 |
| Bra021115 | 1296 | Up | 1.93E-21 |
| Bra027906 | 1368 | Up | 1.93E-21 |
| Bra013450 | 732 | Up | 1.25E-50 |
| Bra009105 | 834 | Up | 5.05E-06 |
| Bra005550 | 1017 | Up | 0.00011417 |
| Bra001988 | 1056 | Up | 1.66E-11 |
| Bra012605 | 402 | Up | 5.05E-06 |
| Bra009184 | 714 | Up | 0 |
| Bra000734 | 1119 | Up | 1.26E-72 |
| Bra000571 | 813 | Up | 1.70E-89 |
| Bra035833 | 708 | Up | 5.79E-18 |
| Bra008940 | 195 | Up | 1.18E-50 |
| Bra018108 | 981 | Up | 3.55E-07 |
| Bra027540 | 1920 | Up | 8.70E-60 |
| Bra017980 | 1392 | Up | 4.69E-111 |
| Bra031074 | 741 | Up | 1.29E-40 |
| Bra018710 | 261 | Up | 8.32E-06 |
| Bra011383 | 690 | Up | 1.07E-09 |
| Bra036360 | 846 | Up | 0.00018688 |
| Bra011928 | 1020 | Up | 0.00018684 |
| Bra004062 | 201 | Up | 0.00018681 |
| Bra025683 | 1143 | Up | 6.97E-48 |
| Bra023619 | 450 | Up | 0.00018691 |
| Bra005486 | 957 | Up | 1.03E-14 |
| Bra026519 | 1155 | Up | 7.54E-11 |
| Bra021053 | 1461 | Up | 2.57E-13 |
| Bra030217 | 1509 | Up | 5.90E-07 |
| Bra029031 | 939 | Up | 5.90E-07 |
| Bra016593 | 786 | Up | 2.06E-22 |
| Bra010960 | 2289 | Up | 5.73E-12 |
| Bra017495 | 738 | Up | 1.17E-15 |
| Bra016193 | 750 | Up | 2.58E-83 |
| Bra024666 | 1005 | Up | 2.95E-09 |
| Bra009869 | 3021 | Up | 1.98E-15 |
| Bra012843 | 1329 | Up | 9.85E-07 |
| Bra040152 | 1074 | Up | 9.85E-07 |
| Bra000811 | 537 | Up | 1.97E-59 |
| Bra009047 | 1893 | Up | 6.98E-08 |
| Bra025883 | 2217 | Up | 1.18E-37 |
| Bra012999 | 1968 | Up | 6.98E-08 |
| Bra032069 | 1248 | Up | 0.00030535 |
| Bra031422 | 516 | Up | 0.00030529 |
| Bra009295 | 903 | Up | 1.53E-28 |
| Bra026520 | 735 | Up | 2.24E-05 |
| Bra001995 | 543 | Up | 3.45E-10 |
| Bra038360 | 1107 | Up | 2.11E-23 |
| Bra027115 | 3480 | Up | 1.69E-18 |
| Bra015384 | 1632 | Up | 1.15E-07 |
| Bra017741 | 1314 | Up | 1.96E-30 |
| Bra030589 | 882 | Up | 6.26E-16 |
| Bra036246 | 1107 | Up | 4.19E-17 |
| Bra037682 | 1170 | Up | 6.13E-135 |
| Bra039279 | 1380 | Up | 4.08E-11 |
| Bra019599 | 1299 | Up | 4.88E-91 |
| Bra036703 | 1179 | Up | 1.21E-27 |
| Bra026860 | 939 | Up | 9.43E-10 |
| Bra028889 | 1896 | Up | 2.67E-06 |
| Bra040324 | 744 | Up | 0.00049394 |
| Bra007390 | 741 | Up | 3.67E-05 |
| Bra011500 | 777 | Up | 1.06E-77 |
| Bra014771 | 1899 | Up | 0.00049386 |
| Bra008497 | 633 | Up | 3.83E-13 |
| Bra000382 | 1533 | Up | 2.67E-06 |
| Bra039555 | 1317 | Up | 3.67E-05 |
| Bra027779 | 3177 | Up | 0.00049403 |
| Bra021925 | 1320 | Up | 1.66E-22 |
| Bra039058 | 1059 | Up | 2.59E-108 |
| Bra009238 | 999 | Up | 2.93E-15 |
| Bra022577 | 1053 | Up | 4.30E-29 |
| Bra020827 | 2046 | Up | 8.23E-12 |
| Bra017460 | 474 | Up | 1.78E-27 |
| Bra039945 | 777 | Up | 3.09E-07 |
| Bra016604 | 813 | Up | 2.65E-26 |
| Bra021539 | 672 | Up | 1.41E-45 |
| Bra021861 | 1323 | Up | 9.18E-31 |
| Bra000880 | 2040 | Up | 3.68E-17 |
| Bra019731 | 2307 | Up | 1.81E-10 |
| Bra007230 | 321 | Up | 4.14E-18 |
| Bra015952 | 1404 | Up | 1.11E-52 |
| Bra031698 | 3174 | Up | 2.13E-21 |
| Bra020088 | 798 | Up | 1.10E-24 |
| Bra031209 | 312 | Up | 9.12E-16 |
| Bra003959 | 1122 | Up | 9.29E-28 |
| Bra028523 | 1002 | Up | 0 |
| Bra037486 | 2310 | Up | 0.00079617 |
| Bra003613 | 1113 | Up | 0.00079605 |
| Bra029472 | 663 | Up | 0.0007958 |
| Bra009036 | 1059 | Up | 1.99E-13 |
| Bra038763 | 1752 | Up | 0.0007963 |
| Bra001507 | 1773 | Up | 1.99E-13 |
| Bra034278 | 2601 | Up | 0.00079592 |
| Bra000660 | 945 | Up | 1.42E-256 |
| Bra011656 | 480 | Up | 2.22E-14 |
| Bra040374 | 1356 | Up | 5.36E-49 |
| Bra030957 | 570 | Up | 7.11E-06 |
| Bra032393 | 1074 | Up | 7.11E-06 |
| Bra022723 | 396 | Up | 2.88E-28 |
| Bra016077 | 411 | Up | 5.99E-08 |
| Bra004065 | 1014 | Up | 1.77E-45 |
| Bra037368 | 897 | Up | 1.90E-17 |
| Bra000056 | 669 | Up | 4.89E-10 |
| Bra029679 | 1449 | Up | 4.89E-10 |
| Bra035732 | 654 | Up | 1.25E-81 |
| Bra012784 | 1473 | Up | 3.71E-14 |
| Bra025207 | 1284 | Up | 9.66E-05 |
| Bra028918 | 873 | Up | 9.65E-05 |
| Bra010050 | 201 | Up | 6.92E-09 |
| Bra012551 | 1404 | Up | 3.16E-17 |
| Bra014598 | 405 | Up | 3.47E-51 |
| Bra031470 | 1218 | Up | 8.99E-29 |
| Bra033474 | 2313 | Up | 6.16E-14 |
| Bra012938 | 777 | Up | 5.98E-56 |
| Bra006949 | 531 | Up | 1.07E-51 |
| Bra016591 | 837 | Up | 1.13E-30 |
| Bra009221 | 1077 | Up | 1.27E-92 |
| Bra019123 | 585 | Up | 9.78E-08 |
| Bra026610 | 2832 | Up | 1.16E-05 |
| Bra025715 | 699 | Up | 1.16E-05 |
| Bra011179 | 849 | Up | 2.77E-29 |
| Bra032246 | 1341 | Up | 1.14E-08 |
| Bra024280 | 915 | Up | 1.47E-82 |
| Bra031738 | 915 | Up | 1.37E-06 |
| Bra008333 | 2721 | Up | 1.34E-24 |
| Bra000925 | 1032 | Up | 1.59E-07 |
| Bra035198 | 771 | Up | 1.59E-07 |
| Bra030916 | 1092 | Up | 1.59E-07 |
| Bra008690 | 1089 | Up | 0.00015506 |
| Bra031540 | 300 | Up | 2.35E-12 |
| Bra028752 | 1119 | Up | 0.00015508 |
| Bra036531 | 666 | Up | 0.00015511 |
| Bra012780 | 1746 | Up | 2.15E-09 |
| Bra012540 | 3831 | Up | 2.16E-09 |
| Bra005818 | 639 | Up | 3.70E-33 |
| Bra006467 | 309 | Up | 1.87E-05 |
| Bra010143 | 1059 | Up | 1.87E-05 |
| Bra015346 | 894 | Up | 5.13E-36 |
| Bra018808 | 1146 | Up | 2.22E-06 |
| Bra006090 | 3120 | Up | 2.22E-06 |
| Bra014674 | 465 | Up | 2.22E-06 |
| Bra029551 | 246 | Up | 2.22E-06 |
| Bra005241 | 1491 | Up | 1.28E-48 |
| Bra025253 | 2352 | Up | 2.58E-07 |
| Bra000430 | 357 | Up | 3.04E-08 |
| Bra013144 | 1344 | Up | 3.51E-09 |
| Bra027664 | 495 | Up | 4.81E-11 |
| Bra008915 | 684 | Up | 5.97E-12 |
| Bra016562 | 498 | Up | 3.01E-05 |
| Bra023127 | 1191 | Up | 7.77E-11 |
| Bra031900 | 594 | Up | 3.01E-05 |
| Bra022605 | 399 | Up | 1.76E-15 |
| Bra004534 | 393 | Up | 2.20E-17 |
| Bra017493 | 885 | Up | 3.98E-22 |
| Bra001123 | 645 | Up | 3.01E-05 |
| Bra013476 | 1620 | Up | 6.81E-192 |
| Bra025882 | 2214 | Up | 1.62E-29 |
| Bra006833 | 1182 | Up | 6.95E-33 |
| Bra026795 | 648 | Up | 1.26E-10 |
| Bra007885 | 858 | Up | 1.37E-49 |
| Bra001701 | 291 | Up | 9.32E-09 |
| Bra024477 | 1512 | Up | 9.32E-09 |
| Bra022001 | 666 | Up | 8.02E-08 |
| Bra003774 | 327 | Up | 8.02E-08 |
| Bra018065 | 1137 | Up | 6.00E-136 |
| Bra000108 | 2217 | Up | 6.82E-07 |
| Bra004982 | 1671 | Up | 1.40E-19 |
| Bra034041 | 9525 | Up | 4.10E-24 |
| Bra001635 | 1218 | Up | 1.54E-41 |
| Bra037181 | 1617 | Up | 6.23E-169 |
| Bra019413 | 2616 | Up | 4.83E-05 |
| Bra012270 | 1131 | Up | 4.83E-05 |
| Bra033158 | 996 | Up | 5.34E-22 |
| Bra000796 | 1827 | Up | 1.80E-17 |
| Bra040264 | 1122 | Up | 1.80E-17 |
| Bra008032 | 354 | Up | 1.52E-08 |
| Bra030157 | 918 | Up | 4.97E-12 |
| Bra022935 | 1566 | Up | 1.44E-15 |
| Bra016430 | 3108 | Up | 0.00039811 |
| Bra007213 | 1713 | Up | 5.26E-28 |
| Bra013634 | 1575 | Up | 2.47E-84 |
| Bra026156 | 4335 | Up | 1.43E-229 |
| Bra036701 | 954 | Up | 1.11E-06 |
| Bra008544 | 2538 | Up | 3.75E-25 |
| Bra021628 | 1152 | Up | 9.32E-06 |
| Bra003071 | 192 | Up | 2.46E-08 |
| Bra019354 | 879 | Up | 7.33E-39 |
| Bra003306 | 408 | Up | 5.38E-30 |
| Bra040157 | 867 | Up | 1.59E-12 |
| Bra030705 | 1254 | Up | 4.32E-22 |
| Bra018368 | 1404 | Up | 9.60E-37 |
| Bra021802 | 2283 | Up | 1.23E-11 |
| Bra005670 | 1527 | Up | 6.33E-46 |
| Bra011665 | 4326 | Up | 1.79E-06 |
| Bra028645 | 837 | Up | 5.05E-44 |
| Bra030501 | 846 | Up | 3.97E-08 |
| Bra030785 | 663 | Up | 1.88E-21 |
| Bra017065 | 537 | Up | 1.49E-05 |
| Bra021641 | 309 | Up | 0.00063233 |
| Bra002747 | 933 | Up | 0.00063223 |
| Bra021503 | 627 | Up | 0.00063213 |
| Bra039012 | 2754 | Up | 5.42E-55 |
| Bra026364 | 879 | Up | 9.96E-56 |
| Bra027659 | 1068 | Up | 1.32E-24 |
| Bra003236 | 525 | Up | 1.65E-14 |
| Bra003575 | 1638 | Up | 1.40E-09 |
| Bra037874 | 1005 | Up | 6.38E-08 |
| Bra019187 | 765 | Up | 6.38E-08 |
| Bra031811 | 1068 | Up | 1.47E-13 |
| Bra027550 | 618 | Up | 6.29E-12 |
| Bra036942 | 1050 | Up | 2.86E-06 |
| Bra011755 | 1542 | Up | 9.18E-22 |
| Bra015791 | 798 | Up | 2.65E-10 |
| Bra008101 | 567 | Up | 3.91E-33 |
| Bra032869 | 435 | Up | 0.00012301 |
| Bra022003 | 1140 | Up | 3.29E-93 |
| Bra008668 | 1014 | Up | 5.38E-07 |
| Bra040586 | 1344 | Up | 2.26E-09 |
| Bra018439 | 756 | Up | 2.26E-09 |
| Bra024900 | 876 | Up | 2.37E-05 |
| Bra028853 | 408 | Up | 2.37E-05 |
| Bra030494 | 456 | Up | 2.43E-29 |
| Bra005193 | 1143 | Up | 3.88E-13 |
| Bra016261 | 861 | Up | 5.69E-26 |
| Bra031912 | 2337 | Up | 1.46E-39 |
| Bra018762 | 1938 | Up | 1.04E-18 |
| Bra029328 | 885 | Up | 2.77E-24 |
| Bra009293 | 1398 | Up | 2.71E-16 |
| Bra020829 | 765 | Up | 3.62E-09 |
| Bra006709 | 1182 | Up | 6.40E-21 |
| Bra012614 | 939 | Up | 3.22E-12 |
| Bra002459 | 483 | Up | 1.68E-18 |
| Bra016230 | 1137 | Up | 6.80E-10 |
| Bra030417 | 2556 | Up | 0.00099287 |
| Bra014623 | 294 | Up | 0.00099272 |
| Bra000670 | 381 | Up | 8.04E-17 |
| Bra026237 | 1608 | Up | 3.52E-30 |
| Bra026902 | 1143 | Up | 1.63E-07 |
| Bra003970 | 705 | Up | 2.11E-14 |
| Bra006624 | 708 | Up | 6.48E-23 |
| Bra001651 | 747 | Up | 0.00019421 |
| Bra031435 | 543 | Up | 0.00019418 |
| Bra015329 | 1617 | Up | 0.00019411 |
| Bra006451 | 1404 | Up | 0.00019425 |
| Bra038486 | 1482 | Up | 0.00019414 |
| Bra038546 | 963 | Up | 9.20E-20 |
| Bra003363 | 1650 | Up | 2.74E-28 |
| Bra027531 | 750 | Up | 1.33E-26 |
| Bra022000 | 1017 | Up | 7.43E-234 |
| Bra011720 | 978 | Up | 2.04E-44 |
| Bra015514 | 756 | Up | 3.76E-05 |
| Bra023790 | 3054 | Up | 3.76E-05 |
| Bra027437 | 897 | Up | 1.10E-09 |
| Bra033441 | 3399 | Up | 3.76E-05 |
| Bra005390 | 1620 | Up | 2.07E-10 |
| Bra029946 | 1032 | Up | 1.62E-42 |
| Bra040819 | 1494 | Up | 3.87E-17 |
| Bra028533 | 564 | Up | 1.32E-28 |
| Bra027692 | 747 | Up | 7.83E-12 |
| Bra028419 | 1146 | Up | 4.79E-43 |
| Bra038031 | 891 | Up | 1.39E-06 |
| Bra026571 | 1824 | Up | 6.24E-17 |
| Bra017450 | 1461 | Up | 2.10E-18 |
| Bra021077 | 1029 | Up | 2.40E-21 |
| Bra016375 | 1695 | Up | 3.73E-43 |
| Bra000352 | 729 | Up | 2.20E-06 |
| Bra032415 | 1083 | Up | 5.58E-32 |
| Bra034823 | 693 | Up | 0.00030644 |
| Bra040924 | 1176 | Up | 1.01E-18 |
| Bra026536 | 567 | Up | 0.00030649 |
| Bra027508 | 957 | Up | 7.91E-08 |
| Bra000720 | 1071 | Up | 1.15E-05 |
| Bra035221 | 1725 | Up | 5.95E-05 |
| Bra019382 | 1122 | Up | 2.79E-49 |
| Bra034088 | 1413 | Up | 1.81E-37 |
| Bra010908 | 1257 | Up | 7.81E-15 |
| Bra011455 | 2298 | Up | 4.48E-09 |
| Bra037877 | 1059 | Up | 1.85E-25 |
| Bra031521 | 2034 | Up | 2.39E-08 |
| Bra004819 | 1197 | Up | 1.26E-07 |
| Bra002388 | 1377 | Up | 3.73E-13 |
| Bra005378 | 2715 | Up | 2.33E-67 |
| Bra004477 | 789 | Up | 3.50E-06 |
| Bra022069 | 1878 | Up | 3.50E-06 |
| Bra039327 | 2952 | Up | 5.24E-55 |
| Bra011821 | 1554 | Up | 7.74E-21 |
| Bra031537 | 1806 | Up | 1.82E-05 |
| Bra024381 | 1455 | Up | 1.77E-33 |
| Bra012665 | 1017 | Up | 1.35E-09 |
| Bra023316 | 1869 | Up | 1.35E-09 |
| Bra019932 | 762 | Up | 5.97E-15 |
| Bra037727 | 1851 | Up | 0.00047871 |
| Bra001533 | 3090 | Up | 7.66E-11 |
| Bra023383 | 1044 | Up | 4.03E-10 |
| Bra038271 | 1044 | Up | 1.05E-06 |
| Bra030367 | 297 | Up | 1.05E-06 |
| Bra028214 | 267 | Up | 1.99E-24 |
| Bra009384 | 894 | Up | 5.21E-14 |
| Bra004321 | 765 | Up | 5.52E-06 |
| Bra034161 | 2568 | Up | 1.21E-10 |
| Bra026797 | 540 | Up | 5.15E-20 |
| Bra037520 | 2769 | Up | 2.16E-68 |
| Bra029099 | 1140 | Up | 8.21E-18 |
| Bra035230 | 411 | Up | 3.39E-09 |
| Bra012720 | 1371 | Up | 1.80E-08 |
| Bra012907 | 1596 | Up | 0.00014597 |
| Bra021530 | 1902 | Up | 3.88E-20 |
| Bra036140 | 483 | Up | 8.66E-06 |
| Bra014189 | 1404 | Up | 3.16E-35 |
| Bra016733 | 2244 | Up | 0.00074462 |
| Bra005607 | 417 | Up | 4.94E-07 |
| Bra020016 | 999 | Up | 4.94E-07 |
| Bra030159 | 891 | Up | 4.94E-07 |
| Bra002584 | 741 | Up | 0.0007445 |
| Bra009268 | 852 | Up | 6.16E-18 |
| Bra011002 | 1080 | Up | 9.88E-24 |
| Bra001051 | 492 | Up | 2.85E-08 |
| Bra023983 | 1038 | Up | 1.13E-12 |
| Bra025406 | 1311 | Up | 4.46E-05 |
| Bra003944 | 1299 | Up | 1.57E-21 |
| Bra013205 | 678 | Up | 2.50E-25 |
| Bra029231 | 2148 | Up | 2.78E-11 |
| Bra027476 | 882 | Up | 8.56E-19 |
| Bra031929 | 1134 | Up | 8.62E-12 |
| Bra009377 | 768 | Up | 0.00022788 |
| Bra016708 | 1752 | Up | 4.49E-08 |
| Bra000551 | 768 | Up | 7.83E-07 |
| Bra022254 | 1008 | Up | 1.18E-23 |
| Bra017526 | 552 | Up | 1.34E-08 |
| Bra001769 | 1320 | Up | 1.35E-16 |
| Bra002110 | 1626 | Up | 2.34E-07 |
| Bra023863 | 1608 | Up | 6.95E-05 |
| Bra031656 | 969 | Up | 2.32E-69 |
| Bra010658 | 1038 | Up | 1.24E-06 |
| Bra038962 | 348 | Up | 6.97E-45 |
| Bra006853 | 639 | Up | 2.11E-05 |
| Bra004330 | 1080 | Up | 3.57E-10 |
| Bra030095 | 507 | Up | 3.49E-25 |
| Bra005020 | 1026 | Up | 3.66E-07 |
| Bra036862 | 447 | Up | 3.66E-07 |
| Bra011666 | 3405 | Up | 3.66E-07 |
| Bra007198 | 636 | Up | 6.28E-09 |
| Bra016463 | 1662 | Up | 5.96E-70 |
| Bra000799 | 1971 | Up | 2.07E-12 |
| Bra029884 | 1587 | Up | 5.47E-16 |
| Bra029819 | 1155 | Up | 0.00035319 |
| Bra030026 | 483 | Up | 0.00035325 |
| Bra014344 | 249 | Up | 6.42E-06 |
| Bra005124 | 1014 | Up | 0.00035331 |
| Bra015298 | 675 | Up | 9.87E-09 |
| Bra040001 | 1482 | Up | 0.0001082 |
| Bra027510 | 3924 | Up | 5.74E-07 |
| Bra007669 | 1125 | Up | 5.74E-07 |
| Bra018573 | 972 | Up | 1.86E-18 |
| Bra009108 | 303 | Up | 1.72E-07 |
| Bra015917 | 3255 | Up | 5.49E-19 |
| Bra023116 | 1005 | Up | 1.72E-07 |
| Bra017022 | 1080 | Up | 3.28E-05 |
| Bra034164 | 912 | Up | 1.72E-07 |
| Bra003975 | 858 | Up | 2.20E-22 |
| Bra011316 | 744 | Up | 2.16E-15 |
| Bra038205 | 1254 | Up | 2.17E-15 |
| Bra028379 | 1506 | Up | 3.24E-71 |
| Bra003699 | 816 | Up | 7.49E-62 |
| Bra016052 | 456 | Up | 9.02E-07 |
| Bra006206 | 1719 | Up | 5.45E-27 |
| Bra006873 | 2628 | Up | 5.46E-27 |
| Bra040624 | 3285 | Up | 5.13E-44 |
| Bra036327 | 1278 | Up | 5.14E-44 |
| Bra004907 | 939 | Up | 0.00054571 |
| Bra008348 | 1026 | Up | 4.04E-24 |
| Bra009767 | 879 | Up | 0.0005458 |
| Bra039968 | 1200 | Up | 8.40E-39 |
| Bra010819 | 1701 | Up | 7.16E-40 |
| Bra022585 | 1746 | Up | 0 |
| Bra001588 | 636 | Up | 1.58E-15 |
| Bra010178 | 1803 | Up | 1.12E-106 |
| Bra021803 | 2229 | Up | 5.07E-05 |
| Bra025371 | 1356 | Up | 9.96E-19 |
| Bra025935 | 3303 | Up | 6.38E-10 |
| Bra015820 | 240 | Up | 1.54E-05 |
| Bra020915 | 1596 | Up | 4.67E-06 |
| Bra030986 | 900 | Up | 8.48E-79 |
| Bra010332 | 792 | Up | 0 |
| Bra036846 | 1617 | Up | 3.92E-15 |
| Bra010122 | 858 | Up | 3.77E-08 |
| Bra016197 | 501 | Up | 9.99E-10 |
| Bra019366 | 1578 | Up | 1.14E-28 |
| Bra030285 | 795 | Up | 2.99E-10 |
| Bra029259 | 876 | Up | 8.44E-31 |
| Bra007279 | 687 | Up | 2.69E-12 |
| Bra035415 | 1473 | Up | 2.35E-39 |
| Bra031851 | 417 | Up | 2.08E-14 |
| Bra012058 | 1596 | Up | 9.17E-81 |
| Bra009218 | 1935 | Up | 9.93E-108 |
| Bra017516 | 2328 | Up | 9.57E-15 |
| Bra013199 | 2022 | Up | 0.00083314 |
| Bra012655 | 642 | Up | 0.00025618 |
| Bra016930 | 981 | Up | 0.00083301 |
| Bra015404 | 981 | Up | 0.00083288 |
| Bra009072 | 2025 | Up | 0.00083327 |
| Bra019357 | 1353 | Up | 2.08E-25 |
| Bra003770 | 861 | Up | 0.00083275 |
| Bra005463 | 906 | Up | 0.00025623 |
| Bra034735 | 1029 | Up | 7.81E-05 |
| Bra009037 | 876 | Up | 3.82E-24 |
| Bra017113 | 354 | Up | 1.20E-93 |
| Bra023339 | 195 | Up | 4.44E-15 |
| Bra007333 | 2295 | Up | 6.92E-52 |
| Bra013797 | 693 | Up | 2.04E-23 |
| Bra028942 | 2880 | Up | 6.20E-12 |
| Bra016785 | 2679 | Up | 2.15E-10 |
| Bra022266 | 1521 | Up | 4.20E-100 |
| Bra032741 | 2568 | Up | 9.08E-08 |
| Bra011337 | 4785 | Up | 9.08E-08 |
| Bra006077 | 912 | Up | 7.92E-20 |
| Bra028836 | 1146 | Up | 1.36E-67 |
| Bra016746 | 1179 | Up | 4.06E-33 |
| Bra038966 | 1353 | Up | 3.65E-05 |
| Bra022168 | 384 | Up | 3.65E-05 |
| Bra039460 | 1191 | Up | 3.74E-09 |
| Bra006453 | 2175 | Up | 0.00011994 |
| Bra010879 | 1830 | Up | 1.24E-16 |
| Bra034845 | 1200 | Up | 7.43E-32 |
| Bra038567 | 1419 | Up | 4.25E-16 |
| Bra011128 | 1503 | Up | 3.01E-27 |
| Bra022174 | 867 | Up | 4.21E-08 |
| Bra014328 | 804 | Up | 8.26E-37 |
| Bra011087 | 1086 | Up | 1.43E-18 |
| Bra000943 | 1803 | Up | 1.40E-07 |
| Bra035526 | 2091 | Up | 1.40E-07 |
| Bra021603 | 2079 | Up | 1.73E-09 |
| Bra015911 | 837 | Up | 1.57E-06 |
| Bra028487 | 1683 | Up | 5.79E-09 |
| Bra023940 | 639 | Up | 2.98E-22 |
| Bra004540 | 858 | Up | 1.11E-32 |
| Bra017743 | 1956 | Up | 3.02E-16 |
| Bra035469 | 1179 | Up | 3.48E-18 |
| Bra012588 | 1644 | Up | 1.70E-05 |
| Bra036362 | 351 | Up | 1.70E-05 |
| Bra029495 | 1950 | Up | 1.70E-05 |
| Bra004630 | 915 | Up | 4.64E-22 |
| Bra006015 | 843 | Up | 1.78E-26 |
| Bra027044 | 2214 | Up | 3.33E-12 |
| Bra022389 | 1281 | Up | 1.37E-16 |
| Bra010220 | 927 | Up | 5.40E-21 |
| Bra026157 | 4389 | Up | 2.76E-55 |
| Bra000165 | 345 | Up | 1.61E-15 |
| Bra006452 | 4986 | Up | 7.17E-07 |
| Bra025728 | 741 | Up | 8.44E-62 |
| Bra011749 | 987 | Up | 1.02E-57 |
| Bra028623 | 1242 | Up | 0.00018262 |
| Bra013715 | 720 | Up | 0.00018259 |
| Bra021558 | 1797 | Up | 3.00E-08 |
| Bra034163 | 1761 | Up | 2.87E-17 |
| Bra023033 | 1275 | Up | 1.50E-49 |
| Bra013176 | 387 | Up | 5.00E-165 |
| Bra031910 | 1746 | Up | 7.90E-06 |
| Bra006543 | 771 | Up | 1.21E-27 |
| Bra023782 | 306 | Up | 0.00059436 |
| Bra009953 | 963 | Up | 1.71E-10 |
| Bra002736 | 528 | Up | 3.29E-07 |
| Bra008583 | 3432 | Up | 3.18E-47 |
| Bra026535 | 1050 | Up | 0.00059427 |
| Bra001368 | 1932 | Up | 4.61E-118 |
| Bra016422 | 1062 | Up | 2.59E-05 |
| Bra015963 | 1053 | Up | 2.59E-05 |
| Bra004165 | 462 | Up | 5.67E-10 |
| Bra034466 | 1440 | Up | 2.80E-173 |
| Bra017464 | 897 | Up | 1.76E-31 |
| Bra009370 | 837 | Up | 6.86E-33 |
| Bra020203 | 1299 | Up | 1.13E-11 |
| Bra027547 | 1623 | Up | 3.66E-06 |
| Bra030430 | 1014 | Up | 8.50E-05 |
| Bra012504 | 1257 | Up | 1.53E-07 |
| Bra000910 | 417 | Up | 3.67E-06 |
| Bra006511 | 1911 | Up | 6.33E-09 |
| Bra027319 | 1539 | Up | 1.07E-99 |
| Bra016284 | 2247 | Up | 1.11E-38 |
| Bra033813 | 1749 | Up | 6.41E-77 |
| Bra000793 | 1560 | Up | 8.71E-10 |
| Bra024649 | 1725 | Up | 9.31E-15 |
| Bra013579 | 1407 | Up | 4.91E-30 |
| Bra032129 | 228 | Up | 5.45E-12 |
| Bra037751 | 1680 | Up | 1.21E-05 |
| Bra040651 | 336 | Up | 3.43E-33 |
| Bra016649 | 1569 | Up | 3.26E-24 |
| Bra003115 | 420 | Up | 3.68E-13 |
| Bra039847 | 909 | Up | 3.95E-05 |
| Bra028520 | 1986 | Up | 3.44E-17 |
| Bra017628 | 789 | Up | 6.22E-29 |
| Bra018130 | 798 | Up | 5.60E-06 |
| Bra003988 | 1443 | Up | 0.00089678 |
| Bra007959 | 1521 | Up | 1.08E-07 |
| Bra011815 | 1044 | Up | 0.00012881 |
| Bra032691 | 1062 | Up | 2.58E-13 |
| Bra018128 | 3123 | Up | 3.06E-22 |
| Bra006179 | 660 | Up | 1.84E-05 |
| Bra033546 | 618 | Up | 2.58E-06 |
| Bra036599 | 1770 | Up | 5.85E-12 |
| Bra009830 | 3144 | Up | 3.56E-07 |
| Bra009379 | 1365 | Up | 6.36E-23 |
| Bra034095 | 231 | Up | 6.84E-09 |
| Bra020758 | 1332 | Up | 1.26E-16 |
| Bra019406 | 1407 | Up | 8.87E-41 |
| Bra034623 | 2505 | Up | 1.74E-24 |
| Bra030189 | 822 | Up | 1.19E-06 |
| Bra000412 | 852 | Up | 8.73E-12 |
| Bra025439 | 483 | Up | 1.52E-76 |
| Bra007708 | 873 | Up | 0.00041804 |
| Bra013227 | 1347 | Up | 8.49E-06 |
| Bra017927 | 426 | Up | 4.33E-10 |
| Bra036864 | 1023 | Up | 1.51E-22 |
| Bra035641 | 1134 | Up | 1.27E-41 |
| Bra004571 | 1854 | Up | 7.59E-08 |
| Bra002767 | 588 | Up | 3.71E-14 |
| Bra005984 | 705 | Up | 3.57E-29 |
| Bra023161 | 966 | Up | 7.82E-22 |
| Bra008695 | 1443 | Up | 1.91E-100 |
| Bra017125 | 615 | Up | 6.58E-10 |
| Bra018402 | 660 | Up | 6.59E-10 |
| Bra005312 | 1485 | Up | 2.77E-05 |
| Bra022663 | 387 | Up | 1.26E-13 |
| Bra025934 | 2514 | Up | 4.79E-09 |
| Bra000214 | 450 | Up | 3.48E-08 |
| Bra039752 | 1269 | Up | 0.0001942 |
| Bra016126 | 1041 | Up | 1.11E-18 |
| Bra010283 | 753 | Up | 1.20E-21 |
| Bra008947 | 1173 | Up | 2.49E-07 |
| Bra014308 | 2202 | Up | 4.64E-16 |
| Bra015915 | 1371 | Up | 5.92E-25 |
| Bra024269 | 987 | Up | 3.75E-18 |
| Bra004029 | 1863 | Up | 5.02E-41 |
| Bra032605 | 1431 | Up | 1.97E-11 |
| Bra017826 | 819 | Up | 1.29E-05 |
| Bra001422 | 1002 | Up | 4.23E-50 |
| Bra037283 | 840 | Up | 1.97E-57 |
| Bra035175 | 1455 | Up | 5.31E-08 |
| Bra013985 | 555 | Up | 8.42E-30 |
| Bra012688 | 4206 | Up | 0.00062875 |
| Bra001610 | 1062 | Up | 0.00062865 |
| Bra031690 | 1584 | Up | 1.12E-34 |
| Bra009057 | 1119 | Up | 4.18E-05 |
| Bra031402 | 999 | Up | 4.18E-05 |
| Bra028586 | 828 | Up | 2.09E-58 |
| Bra006616 | 858 | Up | 2.74E-06 |
| Bra038364 | 444 | Up | 1.74E-07 |
| Bra005543 | 1437 | Up | 1.84E-22 |
| Bra011511 | 1671 | Up | 2.77E-41 |
| Bra027838 | 1023 | Up | 1.24E-14 |
| Bra023588 | 3423 | Up | 8.03E-08 |
| Bra029268 | 726 | Up | 0.00029245 |
| Bra029702 | 198 | Up | 0.0002925 |
| Bra020566 | 636 | Up | 1.94E-05 |
| Bra013904 | 1332 | Up | 4.97E-36 |
| Bra021745 | 1467 | Up | 1.16E-15 |
| Bra016520 | 780 | Up | 3.16E-17 |
| Bra034589 | 732 | Up | 8.95E-06 |
| Bra036902 | 1230 | Up | 1.07E-09 |
| Bra033563 | 795 | Up | 8.88E-23 |
| Bra006022 | 1008 | Up | 0.0001353 |
| Bra029001 | 339 | Up | 4.02E-23 |
| Bra035778 | 558 | Up | 6.46E-14 |
| Bra028126 | 1308 | Up | 4.57E-30 |
| Bra035723 | 1101 | Up | 1.21E-07 |
| Bra029842 | 1248 | Up | 5.97E-15 |
| Bra036495 | 762 | Up | 2.54E-28 |
| Bra030755 | 951 | Up | 2.07E-46 |
| Bra018998 | 1152 | Up | 6.07E-67 |
| Bra018675 | 621 | Up | 0.00093737 |
| Bra020322 | 339 | Up | 0.00093723 |
| Bra009756 | 2001 | Up | 0.00093766 |
| Bra002944 | 699 | Up | 4.18E-19 |
| Bra036211 | 396 | Up | 0.00093752 |
| Bra002002 | 1074 | Up | 2.20E-11 |
| Bra025541 | 1119 | Up | 3.31E-13 |
| Bra023966 | 711 | Up | 3.96E-07 |
| Bra009632 | 1245 | Up | 1.35E-05 |
| Bra015499 | 2079 | Up | 9.93E-223 |
| Bra011828 | 1383 | Up | 2.36E-89 |
| Bra014635 | 1041 | Up | 7.57E-70 |
| Bra007863 | 1017 | Up | 0.00043649 |
| Bra020996 | 1467 | Up | 0.00043678 |
| Bra007792 | 1545 | Up | 0.00043656 |
| Bra033776 | 1578 | Up | 0.00043671 |
| Bra015631 | 1137 | Up | 0.00043664 |
| Bra022398 | 1908 | Up | 1.71E-33 |
| Bra006598 | 723 | Up | 8.41E-08 |
| Bra024952 | 1092 | Up | 5.43E-21 |
| Bra036774 | 1773 | Up | 2.40E-34 |
| Bra022229 | 885 | Up | 2.86E-06 |
| Bra025457 | 1428 | Up | 5.10E-10 |
| Bra010532 | 582 | Up | 2.86E-06 |
| Bra002084 | 720 | Up | 0.00020257 |
| Bra029258 | 1032 | Up | 3.49E-12 |
| Bra010231 | 999 | Up | 3.48E-12 |
| Bra039533 | 891 | Up | 2.34E-10 |
| Bra027376 | 2331 | Up | 7.45E-104 |
| Bra016771 | 891 | Up | 9.39E-05 |
| Bra029505 | 1374 | Up | 4.92E-18 |
| Bra004736 | 843 | Up | 4.34E-05 |
| Bra009292 | 1332 | Up | 2.74E-07 |
| Bra003670 | 2733 | Up | 1.16E-21 |
| Bra002171 | 309 | Up | 2.01E-05 |
| Bra030988 | 2760 | Up | 4.22E-20 |
| Bra010551 | 5961 | Up | 5.82E-08 |
| Bra026368 | 2934 | Up | 2.67E-08 |
| Bra021686 | 1608 | Up | 2.67E-08 |
| Bra013660 | 1881 | Up | 3.15E-19 |
| Bra012850 | 2616 | Up | 2.89E-46 |
| Bra003287 | 1617 | Up | 2.99E-33 |
| Bra013363 | 1380 | Up | 3.35E-36 |
| Bra034925 | 702 | Up | 5.55E-09 |
| Bra032019 | 1011 | Up | 1.48E-45 |
| Bra023398 | 828 | Up | 9.16E-16 |
| Bra025921 | 753 | Up | 0.00064731 |
| Bra027336 | 1794 | Up | 3.80E-17 |
| Bra018462 | 2286 | Up | 4.05E-21 |
| Bra013748 | 1809 | Up | 0.00013934 |
| Bra014747 | 1356 | Up | 3.00E-05 |
| Bra019875 | 891 | Up | 9.99E-103 |
| Bra018609 | 1170 | Up | 1.39E-05 |
| Bra015656 | 2673 | Up | 2.41E-237 |
| Bra001729 | 1734 | Up | 7.70E-37 |
| Bra030231 | 954 | Up | 3.65E-32 |
| Bra027374 | 387 | Up | 2.95E-06 |
| Bra007091 | 2118 | Up | 2.95E-06 |
| Bra029642 | 1389 | Up | 1.62E-23 |
| Bra005125 | 1470 | Up | 3.05E-25 |
| Bra035236 | 1467 | Up | 1.39E-134 |
| Bra013292 | 1173 | Up | 1.30E-07 |
| Bra008991 | 660 | Up | 5.99E-08 |
| Bra001653 | 480 | Up | 3.12E-117 |
| Bra036850 | 1248 | Up | 1.19E-09 |
| Bra005539 | 1941 | Up | 2.31E-19 |
| Bra001825 | 1569 | Up | 2.45E-11 |
| Bra002564 | 954 | Up | 1.67E-23 |
| Bra007945 | 690 | Up | 5.84E-27 |
| Bra034648 | 1998 | Up | 3.23E-61 |
| Bra006086 | 390 | Up | 4.05E-17 |
| Bra026471 | 1674 | Up | 9.61E-05 |
| Bra005163 | 906 | Up | 0.00044605 |
| Bra040260 | 1095 | Up | 1.93E-07 |
| Bra023846 | 1254 | Up | 9.61E-05 |
| Bra027605 | 1152 | Up | 4.21E-07 |
| Bra006824 | 1467 | Up | 9.61E-05 |
| Bra035952 | 726 | Up | 9.52E-06 |
| Bra029252 | 453 | Up | 7.19E-15 |
| Bra004298 | 783 | Up | 0.00095485 |
| Bra014914 | 1485 | Up | 9.62E-05 |
| Bra003108 | 2631 | Up | 0.00044612 |
| Bra029650 | 483 | Up | 0.00020676 |
| Bra010644 | 1557 | Up | 0.0009547 |
| Bra017523 | 1524 | Up | 4.45E-05 |
| Bra011241 | 1638 | Up | 0.00020672 |
| Bra034295 | 1575 | Up | 4.65E-35 |
| Bra010280 | 552 | Up | 4.86E-25 |
| Bra028996 | 1134 | Up | 2.64E-91 |
| Bra010209 | 1437 | Up | 1.18E-13 |
| Bra008489 | 378 | Up | 5.59E-12 |
| Bra015398 | 2856 | Up | 5.59E-12 |
| Bra017390 | 2046 | Up | 2.60E-18 |
| Bra025035 | 534 | Up | 1.28E-08 |
| Bra016512 | 1056 | Up | 3.06E-16 |
| Bra022430 | 1410 | Up | 1.51E-15 |
| Bra009491 | 4278 | Up | 6.11E-08 |
| Bra009338 | 711 | Up | 1.33E-07 |
| Bra008756 | 1569 | Up | 2.08E-45 |
| Bra010834 | 2160 | Up | 6.88E-26 |
| Bra035023 | 1977 | Up | 6.33E-32 |
| Bra028910 | 675 | Up | 1.84E-12 |
| Bra018850 | 1497 | Up | 6.53E-06 |
| Bra032083 | 1242 | Up | 6.53E-06 |
| Bra001919 | 2157 | Up | 6.54E-06 |
| Bra035707 | 2055 | Up | 4.67E-21 |
| Bra002992 | 1125 | Up | 1.41E-05 |
| Bra019645 | 492 | Up | 1.10E-14 |
| Bra017111 | 591 | Up | 0.00014143 |
| Bra015999 | 684 | Up | 1.91E-08 |
| Bra040056 | 1224 | Up | 0.0001414 |
| Bra015543 | 1794 | Up | 4.17E-08 |
| Bra022337 | 1797 | Up | 0.00030578 |
| Bra015660 | 711 | Up | 0.00030573 |
| Bra013668 | 2103 | Up | 1.97E-07 |
| Bra033148 | 1116 | Up | 0.0006548 |
| Bra020967 | 558 | Up | 5.19E-165 |
| Bra015964 | 1374 | Up | 2.05E-06 |
| Bra038432 | 1671 | Up | 2.05E-06 |
| Bra017508 | 1233 | Up | 1.57E-20 |
| Bra022531 | 1632 | Up | 8.28E-12 |
| Bra036343 | 897 | Up | 5.92E-09 |
| Bra020376 | 795 | Up | 3.56E-30 |
| Bra008391 | 1341 | Up | 9.64E-06 |
| Bra033991 | 2742 | Up | 3.87E-10 |
| Bra007161 | 1362 | Up | 7.60E-15 |
| Bra026455 | 360 | Up | 8.43E-10 |
| Bra004066 | 756 | Up | 3.26E-79 |
| Bra008095 | 1299 | Up | 2.91E-07 |
| Bra008132 | 1020 | Up | 1.35E-94 |
| Bra019233 | 2409 | Up | 4.48E-05 |
| Bra036402 | 672 | Up | 4.48E-05 |
| Bra017148 | 918 | Up | 1.29E-33 |
| Bra013657 | 1671 | Up | 4.02E-09 |
| Bra035133 | 285 | Up | 9.68E-05 |
| Bra036516 | 894 | Up | 9.69E-05 |
| Bra011249 | 1452 | Up | 9.68E-05 |
| Bra018731 | 3258 | Up | 1.40E-06 |
| Bra003503 | 438 | Up | 1.40E-06 |
| Bra022923 | 921 | Up | 3.92E-24 |
| Bra026009 | 2019 | Up | 4.83E-122 |
| Bra033093 | 729 | Up | 0.00020791 |
| Bra003858 | 2649 | Up | 3.03E-06 |
| Bra007924 | 1146 | Up | 1.23E-13 |
| Bra009633 | 1635 | Up | 3.20E-61 |
| Bra031556 | 2856 | Up | 9.17E-08 |
| Bra030637 | 1146 | Up | 6.56E-06 |
| Bra020705 | 1659 | Up | 7.93E-20 |
| Bra024459 | 966 | Up | 1.03E-41 |
| Bra021052 | 2379 | Up | 0.00044818 |
| Bra010153 | 960 | Up | 0.00044825 |
| Bra026701 | 453 | Up | 1.07E-15 |
| Bra014511 | 2799 | Up | 1.42E-05 |
| Bra024089 | 840 | Up | 6.83E-22 |
| Bra031333 | 1338 | Up | 4.06E-13 |
| Bra036342 | 1284 | Up | 1.31E-23 |
| Bra005743 | 261 | Up | 0.000957 |
| Bra029496 | 1914 | Up | 9.47E-07 |
| Bra019785 | 1008 | Up | 9.47E-07 |
| Bra003234 | 384 | Up | 6.43E-23 |
| Bra008343 | 936 | Up | 8.19E-45 |
| Bra035334 | 1350 | Up | 2.05E-06 |
| Bra031932 | 315 | Up | 2.05E-06 |
| Bra010320 | 1350 | Up | 2.92E-23 |
| Bra007940 | 1245 | Up | 1.94E-08 |
| Bra001341 | 1377 | Up | 0.00014155 |
| Bra003822 | 2100 | Up | 1.23E-24 |
| Bra022447 | 1383 | Up | 4.22E-08 |
| Bra004316 | 966 | Up | 1.80E-10 |
| Bra030771 | 1308 | Up | 1.35E-77 |
| Bra025522 | 1128 | Up | 4.48E-05 |
| Bra016553 | 1002 | Up | 2.86E-08 |
| Bra035931 | 1944 | Up | 0.00065263 |
| Bra028662 | 690 | Up | 0.00065274 |
| Bra036546 | 1164 | Up | 5.25E-41 |
| Bra009876 | 1380 | Up | 3.02E-38 |
| Bra011985 | 1758 | Up | 0 |
| Bra007985 | 1356 | Up | 1.28E-18 |
| Bra039495 | 1506 | Up | 1.35E-07 |
| Bra007743 | 1350 | Up | 1.35E-07 |
| Bra008255 | 1278 | Up | 2.04E-17 |
| Bra028320 | 2898 | Up | 2.05E-06 |
| Bra023471 | 894 | Up | 2.05E-06 |
| Bra021743 | 1371 | Up | 6.01E-13 |
| Bra026986 | 1395 | Up | 0.0002068 |
| Bra025405 | 348 | Up | 4.44E-06 |
| Bra020715 | 1494 | Up | 3.83E-35 |
| Bra025150 | 3612 | Up | 5.55E-25 |
| Bra032432 | 1143 | Up | 1.77E-19 |
| Bra021101 | 831 | Up | 9.14E-08 |
| Bra003208 | 2124 | Up | 2.69E-59 |
| Bra019829 | 489 | Up | 0.00044465 |
| Bra031582 | 1053 | Up | 6.55E-05 |
| Bra018131 | 1173 | Up | 0.00044457 |
| Bra011088 | 1923 | Up | 6.55E-05 |
| Bra011437 | 1119 | Up | 0.0004445 |
| Bra016382 | 1443 | Up | 8.80E-09 |
| Bra039737 | 747 | Up | 3.01E-06 |
| Bra033201 | 564 | Up | 0.00094597 |
| Bra035721 | 564 | Up | 5.91E-09 |
| Bra022905 | 2559 | Up | 2.70E-13 |
| Bra024735 | 1119 | Up | 9.06E-18 |
| Bra002280 | 855 | Up | 3.86E-19 |
| Bra027257 | 831 | Up | 4.43E-05 |
| Bra034147 | 1890 | Up | 1.83E-09 |
| Bra002416 | 1482 | Up | 1.50E-21 |
| Bra009629 | 1074 | Up | 1.06E-15 |
| Bra031248 | 1572 | Up | 1.81E-13 |
| Bra008581 | 1035 | Up | 1.85E-18 |
| Bra005974 | 1158 | Up | 1.40E-05 |
| Bra011786 | 849 | Up | 1.40E-05 |
| Bra037942 | 417 | Up | 7.25E-21 |
| Bra035893 | 1008 | Up | 1.23E-09 |
| Bra017076 | 954 | Up | 2.74E-12 |
| Bra029430 | 918 | Up | 4.38E-06 |
| Bra032645 | 696 | Up | 1.50E-26 |
| Bra025961 | 774 | Up | 2.56E-19 |
| Bra008836 | 1713 | Up | 6.86E-32 |
| Bra027662 | 759 | Up | 5.62E-19 |
| Bra040598 | 1377 | Up | 5.86E-13 |
| Bra013523 | 2067 | Up | 4.21E-07 |
| Bra028835 | 1512 | Up | 3.93E-12 |
| Bra027990 | 2010 | Up | 9.44E-06 |
| Bra034817 | 1371 | Up | 4.55E-37 |
| Bra033046 | 1725 | Up | 1.14E-19 |
| Bra011554 | 1947 | Up | 1.32E-07 |
| Bra002655 | 264 | Up | 0.00020372 |
| Bra038227 | 1410 | Up | 1.81E-18 |
| Bra005227 | 1455 | Up | 1.24E-38 |
| Bra032613 | 1281 | Up | 6.45E-05 |
| Bra008130 | 963 | Up | 1.60E-30 |
| Bra030584 | 987 | Up | 8.03E-14 |
| Bra012088 | 972 | Up | 2.43E-14 |
| Bra032940 | 1332 | Up | 8.91E-08 |
| Bra029939 | 1518 | Up | 2.03E-05 |
| Bra011049 | 1470 | Up | 1.99E-27 |
| Bra036575 | 1599 | Up | 2.77E-08 |
| Bra034851 | 936 | Up | 2.77E-08 |
| Bra024643 | 1164 | Up | 6.40E-06 |
| Bra001371 | 705 | Up | 1.99E-06 |
| Bra022338 | 786 | Up | 1.99E-06 |
| Bra027170 | 1119 | Up | 0.00043561 |
| Bra002626 | 1743 | Up | 0.00043568 |
| Bra013823 | 1338 | Up | 5.99E-08 |
| Bra011735 | 858 | Up | 0.00013745 |
| Bra021940 | 1884 | Up | 0.00013743 |
| Bra015784 | 1242 | Up | 8.41E-13 |
| Bra034103 | 960 | Up | 1.04E-30 |
| Bra018863 | 2559 | Up | 1.37E-05 |
| Bra020294 | 1866 | Up | 4.30E-06 |
| Bra034404 | 2739 | Up | 7.45E-100 |
| Bra032084 | 978 | Up | 4.03E-08 |
| Bra025654 | 567 | Up | 4.14E-17 |
| Bra011148 | 1497 | Up | 1.06E-14 |
| Bra016392 | 1737 | Up | 8.71E-08 |
| Bra031393 | 1179 | Up | 1.60E-19 |
| Bra040475 | 3864 | Up | 7.87E-12 |
| Bra028068 | 786 | Up | 8.26E-13 |
| Bra006547 | 822 | Up | 4.06E-17 |
| Bra010111 | 1839 | Up | 1.05E-29 |
| Bra011590 | 1443 | Up | 5.43E-12 |
| Bra016237 | 1152 | Up | 1.12E-33 |
| Bra021569 | 2142 | Up | 6.01E-07 |
| Bra026635 | 1134 | Up | 5.50E-18 |
| Bra037954 | 1152 | Up | 1.12E-11 |
| Bra013019 | 1173 | Up | 1.98E-05 |
| Bra039130 | 861 | Up | 6.97E-15 |
| Bra009235 | 1005 | Up | 6.28E-05 |
| Bra020826 | 441 | Up | 0.00019835 |
| Bra016265 | 1185 | Up | 0.00019839 |
| Bra029407 | 1530 | Up | 1.65E-13 |
| Bra003304 | 819 | Up | 0.00062596 |
| Bra028402 | 1779 | Up | 4.02E-07 |
| Bra027477 | 582 | Up | 0.00062606 |
| Bra024345 | 1698 | Up | 3.23E-70 |
| Bra034676 | 1149 | Up | 1.31E-06 |
| Bra032150 | 1074 | Up | 3.08E-57 |
| Bra008857 | 759 | Up | 4.18E-06 |
| Bra027377 | 2064 | Up | 6.25E-21 |
| Bra024237 | 1506 | Up | 3.60E-23 |
| Bra001253 | 510 | Up | 4.23E-05 |
| Bra009599 | 1170 | Up | 7.86E-13 |
| Bra027073 | 1506 | Up | 3.00E-20 |
| Bra005720 | 243 | Up | 4.49E-43 |
| Bra029583 | 1959 | Up | 3.68E-27 |
| Bra010075 | 615 | Up | 1.33E-95 |
| Bra003969 | 723 | Up | 5.85E-07 |
| Bra008530 | 1392 | Up | 2.45E-09 |
| Bra015174 | 1374 | Up | 1.23E-30 |
| Bra002189 | 1458 | Up | 3.90E-07 |
| Bra032560 | 801 | Up | 3.91E-07 |
| Bra037860 | 1800 | Up | 1.56E-13 |
| Bra009774 | 1368 | Up | 1.63E-22 |
| Bra029765 | 243 | Up | 6.44E-64 |
| Bra015570 | 1545 | Up | 3.42E-134 |
| Bra002224 | 1008 | Up | 1.09E-22 |
| Bra009315 | 1581 | Up | 1.27E-06 |
| Bra016182 | 627 | Up | 1.27E-06 |
| Bra016669 | 4314 | Up | 3.42E-10 |
| Bra001447 | 903 | Up | 1.52E-11 |
| Bra028651 | 2055 | Up | 3.72E-202 |
| Bra026864 | 2433 | Up | 8.49E-07 |
| Bra024716 | 1218 | Up | 0.00089341 |
| Bra031650 | 549 | Up | 0.00089355 |
| Bra017960 | 876 | Up | 3.17E-49 |
| Bra026125 | 2595 | Up | 1.53E-10 |
| Bra007733 | 795 | Up | 2.30E-22 |
| Bra031935 | 540 | Up | 0.00019191 |
| Bra018864 | 612 | Up | 0.00019188 |
| Bra037725 | 1419 | Up | 3.69E-08 |
| Bra011605 | 1638 | Up | 1.40E-98 |
| Bra038361 | 474 | Up | 8.39E-16 |
| Bra006966 | 906 | Up | 4.09E-05 |
| Bra002423 | 1299 | Up | 1.35E-14 |
| Bra018122 | 687 | Up | 1.47E-11 |
| Bra035841 | 1635 | Up | 3.33E-12 |
| Bra029105 | 864 | Up | 1.83E-06 |
| Bra030692 | 1293 | Up | 0.00060339 |
| Bra015531 | 1539 | Up | 6.01E-15 |
| Bra029248 | 1839 | Up | 2.26E-12 |
| Bra026621 | 1482 | Up | 0.0001291 |
| Bra011164 | 2619 | Up | 2.75E-05 |
| Bra000536 | 1137 | Up | 8.69E-15 |
| Bra018468 | 2901 | Up | 3.48E-25 |
| Bra024689 | 579 | Up | 0.00040622 |
| Bra003881 | 357 | Up | 1.85E-05 |
| Bra014489 | 843 | Up | 3.19E-10 |
| Bra033345 | 627 | Up | 4.93E-09 |
| Bra006235 | 1614 | Up | 2.29E-45 |
| Bra011765 | 1263 | Up | 6.02E-27 |
| Bra030459 | 678 | Up | 1.24E-05 |
| Bra010076 | 597 | Up | 5.85E-05 |
| Bra034436 | 1641 | Up | 5.85E-05 |
| Bra011407 | 1434 | Up | 2.63E-59 |
| Bra024075 | 1293 | Up | 7.27E-45 |
| Bra029769 | 2271 | Up | 0.00027416 |
| Bra011043 | 2064 | Up | 4.58E-10 |
| Bra004743 | 759 | Up | 1.76E-06 |
| Bra027736 | 918 | Up | 3.07E-10 |
| Bra015646 | 1020 | Up | 2.44E-07 |
| Bra005694 | 1578 | Up | 3.16E-90 |
| Bra033840 | 1362 | Up | 4.98E-32 |
| Bra027458 | 1332 | Up | 2.63E-05 |
| Bra038762 | 1467 | Up | 7.84E-07 |
| Bra000884 | 285 | Up | 6.41E-17 |
| Bra018834 | 2556 | Up | 3.75E-06 |
| Bra015053 | 1935 | Up | 4.33E-74 |
| Bra032174 | 960 | Up | 0.00012403 |
| Bra009608 | 1644 | Up | 6.18E-11 |
| Bra035213 | 1080 | Up | 1.37E-12 |
| Bra027837 | 387 | Up | 1.78E-05 |
| Bra025601 | 1182 | Up | 6.13E-12 |
| Bra017462 | 1929 | Up | 1.69E-18 |
| Bra016255 | 495 | Up | 0.000577 |
| Bra025003 | 681 | Up | 3.48E-07 |
| Bra022872 | 1122 | Up | 1.19E-05 |
| Bra005918 | 618 | Up | 1.69E-06 |
| Bra000647 | 1626 | Up | 2.87E-12 |
| Bra026506 | 651 | Up | 2.33E-07 |
| Bra017889 | 2373 | Up | 4.53E-09 |
| Bra012387 | 1389 | Up | 7.98E-06 |
| Bra011514 | 2391 | Up | 2.47E-62 |
| Bra023327 | 2907 | Up | 0.00038871 |
| Bra017005 | 1335 | Up | 2.60E-17 |
| Bra020823 | 1665 | Up | 1.06E-14 |
| Bra037055 | 747 | Up | 3.76E-05 |
| Bra008739 | 1950 | Up | 0.00026172 |
| Bra028604 | 1089 | Up | 2.51E-05 |
| Bra026876 | 1290 | Up | 0.00017541 |
| Bra036882 | 1398 | Up | 4.50E-15 |
| Bra024187 | 1197 | Up | 2.09E-29 |
| Bra002870 | 1962 | Up | 0.00011841 |
| Bra025209 | 546 | Up | 1.14E-05 |
| Bra007757 | 1101 | Up | 0.00081401 |
| Bra024634 | 1404 | Up | 3.18E-14 |
| Bra011201 | 753 | Up | 3.44E-41 |
| Bra018804 | 1500 | Up | 4.47E-08 |
| Bra031968 | 777 | Up | 5.32E-05 |
| Bra022867 | 1404 | Up | 6.57E-18 |
| Bra004680 | 342 | Up | 0.00055035 |
| Bra006181 | 1791 | Up | 3.17E-07 |
| Bra019672 | 600 | Up | 0.00055044 |
| Bra012613 | 2385 | Up | 4.57E-14 |
| Bra039214 | 1011 | Up | 2.28E-06 |
| Bra000477 | 789 | Up | 0.0003692 |
| Bra024656 | 333 | Up | 0.00036913 |
| Bra017134 | 1398 | Up | 1.31E-73 |
| Bra034418 | 1518 | Up | 2.24E-13 |
| Bra027827 | 1065 | Up | 2.39E-05 |
| Bra029230 | 987 | Up | 3.45E-11 |
| Bra036935 | 831 | Up | 1.55E-11 |
| Bra034386 | 1443 | Up | 6.55E-14 |
| Bra024084 | 2370 | Up | 1.21E-28 |
| Bra037294 | 1488 | Up | 1.09E-12 |
| Bra004075 | 606 | Up | 1.08E-186 |
| Bra010155 | 1008 | Up | 1.08E-05 |
| Bra024687 | 1458 | Up | 3.36E-12 |
| Bra027504 | 576 | Up | 3.44E-32 |
| Bra008667 | 2115 | Up | 5.56E-15 |
| Bra032090 | 1059 | Up | 0.00011219 |
| Bra033743 | 1614 | Up | 0.00011221 |
| Bra008261 | 1746 | Up | 1.53E-12 |
| Bra017077 | 936 | Up | 1.26E-08 |
| Bra034609 | 1368 | Up | 6.52E-20 |
| Bra024449 | 3573 | Up | 5.59E-09 |
| Bra019409 | 4017 | Up | 7.51E-05 |
| Bra020666 | 3264 | Up | 2.19E-53 |
| Bra018293 | 3963 | Up | 2.16E-06 |
| Bra016862 | 1242 | Up | 8.01E-43 |
| Bra010954 | 525 | Up | 5.05E-05 |
| Bra019416 | 879 | Up | 3.66E-21 |
| Bra001618 | 2172 | Up | 1.32E-89 |
| Bra036278 | 1719 | Up | 7.48E-82 |
| Bra022754 | 1662 | Up | 4.03E-08 |
| Bra023847 | 2022 | Up | 3.38E-05 |
| Bra021062 | 1179 | Up | 3.38E-05 |
| Bra037270 | 1779 | Up | 3.11E-11 |
| Bra021636 | 1518 | Up | 2.26E-05 |
| Bra021653 | 2400 | Up | 1.38E-40 |
| Bra008993 | 450 | Up | 2.54E-59 |
| Bra005007 | 957 | Up | 1.52E-05 |
| Bra032985 | 2352 | Up | 0.00052003 |
| Bra024328 | 621 | Up | 0.00034914 |
| Bra006416 | 528 | Up | 0.0003492 |
| Bra032681 | 1518 | Up | 2.09E-36 |
| Bra010775 | 2172 | Up | 1.11E-40 |
| Bra001149 | 357 | Up | 0.00015699 |
| Bra017502 | 744 | Up | 5.46E-37 |
| Bra000342 | 1206 | Up | 0.00010603 |
| Bra040854 | 2121 | Up | 7.49E-09 |
| Bra001930 | 1074 | Up | 2.96E-15 |
| Bra041010 | 1437 | Up | 4.76E-05 |
| Bra011701 | 942 | Up | 2.69E-07 |
| Bra006197 | 1314 | Up | 2.14E-05 |
| Bra011929 | 312 | Up | 2.14E-05 |
| Bra001227 | 969 | Up | 2.14E-05 |
| Bra017296 | 555 | Up | 5.38E-08 |
| Bra027553 | 1308 | Up | 5.38E-08 |
| Bra009007 | 1308 | Up | 2.16E-47 |
| Bra027509 | 2286 | Up | 3.09E-14 |
| Bra015227 | 1770 | Up | 2.18E-49 |
| Bra035326 | 2016 | Up | 9.29E-10 |
| Bra007507 | 1542 | Up | 8.58E-07 |
| Bra038170 | 465 | Up | 2.21E-16 |
| Bra008246 | 1113 | Up | 1.05E-18 |
| Bra040145 | 441 | Up | 0.00022076 |
| Bra018538 | 1551 | Up | 0.00014773 |
| Bra015560 | 1530 | Up | 9.96E-05 |
| Bra005175 | 969 | Up | 1.82E-71 |
| Bra038700 | 978 | Up | 6.68E-05 |
| Bra013358 | 1554 | Up | 4.47E-05 |
| Bra027867 | 2766 | Up | 4.47E-05 |
| Bra026565 | 2445 | Up | 3.89E-10 |
| Bra037199 | 1410 | Up | 4.05E-06 |
| Bra011869 | 489 | Up | 4.10E-67 |
| Bra022063 | 693 | Up | 1.82E-06 |
| Bra038612 | 1092 | Up | 1.21E-06 |
| Bra009925 | 1704 | Up | 5.34E-07 |
| Bra020770 | 1851 | Up | 2.38E-07 |
| Bra033582 | 1341 | Up | 2.38E-07 |
| Bra004750 | 2796 | Up | 4.33E-58 |
| Bra024703 | 2349 | Up | 2.77E-09 |
| Bra000124 | 1803 | Up | 1.23E-09 |
| Bra027057 | 1659 | Up | 8.19E-10 |
| Bra033438 | 930 | Up | 1.41E-20 |
| Bra007099 | 975 | Up | 3.78E-53 |
| Bra031329 | 1023 | Up | 0 |
| Bra037157 | 1215 | Up | 1.09E-26 |
| Bra002038 | 1143 | Up | 2.54E-06 |
| Bra024254 | 1101 | Up | 1.27E-05 |
| Bra002712 | 1215 | Up | 1.49E-07 |
| Bra022302 | 525 | Up | 9.34E-05 |
| Bra034595 | 723 | Up | 1.89E-05 |
| Bra026550 | 1929 | Up | 2.81E-05 |
| Bra023711 | 435 | Up | 5.12E-10 |
| Bra019821 | 1026 | Up | 1.65E-28 |
| Bra011409 | 1419 | Up | 0.00045811 |
| Bra013614 | 1080 | Up | 8.48E-06 |
| Bra013777 | 1173 | Up | 1.53E-10 |
| Bra017180 | 966 | Up | 3.41E-14 |
| Bra030359 | 1398 | Up | 0.0001386 |
| Bra031080 | 912 | Up | 0.00020671 |
| Bra015281 | 807 | Up | 0.00030827 |
| Bra018591 | 1500 | Up | 1.49E-14 |
| Bra004417 | 615 | Up | 4.32E-22 |
| Bra008998 | 1167 | Up | 1.01E-18 |
| Bra028837 | 675 | Up | 1.78E-28 |
| Bra020237 | 501 | Up | 8.90E-11 |
| Bra018168 | 1377 | Up | 2.00E-10 |
| Bra040531 | 3144 | Up | 1.01E-09 |
| Bra016908 | 1509 | Up | 1.52E-09 |
| Bra026809 | 987 | Up | 1.52E-09 |
| Bra002367 | 2517 | Up | 2.88E-36 |
| Bra036491 | 1671 | Up | 8.72E-05 |
| Bra018425 | 1272 | Up | 1.74E-08 |
| Bra003064 | 1002 | Up | 1.74E-08 |
| Bra010182 | 978 | Up | 0.00012979 |
| Bra040932 | 726 | Up | 4.62E-20 |
| Bra016914 | 762 | Up | 0.00019325 |
| Bra016765 | 1575 | Up | 1.31E-07 |
| Bra035183 | 1080 | Up | 1.31E-07 |
| Bra029481 | 957 | Up | 6.87E-35 |
| Bra031382 | 846 | Up | 4.64E-13 |
| Bra015880 | 738 | Up | 9.90E-07 |
| Bra034439 | 1737 | Up | 0.00094401 |
| Bra013678 | 2007 | Up | 5.00E-56 |
| Bra003842 | 927 | Up | 8.16E-08 |
| Bra021187 | 507 | Up | 7.30E-29 |
| Bra028665 | 1029 | Up | 5.71E-28 |
| Bra038662 | 1026 | Up | 6.83E-12 |
| Bra001249 | 768 | Up | 7.64E-100 |
| Bra032396 | 1257 | Up | 3.28E-11 |
| Bra009366 | 1578 | Up | 5.32E-28 |
| Bra025334 | 1785 | Up | 8.13E-05 |
| Bra026194 | 1863 | Up | 1.71E-23 |
| Bra030759 | 1284 | Up | 3.85E-19 |
| Bra039500 | 903 | Up | 0.00026822 |
| Bra024588 | 1404 | Up | 0.00026831 |
| Bra031390 | 1170 | Up | 0.00026827 |
| Bra039995 | 1461 | Up | 5.71E-15 |
| Bra012530 | 1071 | Up | 6.89E-06 |
| Bra009578 | 990 | Up | 6.88E-06 |
| Bra008855 | 1797 | Up | 1.70E-07 |
| Bra008841 | 2256 | Up | 1.03E-05 |
| Bra034681 | 3267 | Up | 1.03E-05 |
| Bra024848 | 972 | Up | 5.37E-48 |
| Bra022264 | 1032 | Up | 4.09E-12 |
| Bra024996 | 687 | Up | 4.14E-14 |
| Bra022870 | 1938 | Up | 8.84E-63 |
| Bra010996 | 1767 | Up | 2.57E-09 |
| Bra022148 | 1149 | Up | 1.92E-06 |
| Bra001646 | 645 | Up | 5.07E-05 |
| Bra033938 | 1281 | Up | 7.01E-19 |
| Bra039471 | 405 | Up | 2.35E-07 |
| Bra001244 | 1344 | Up | 0.00011253 |
| Bra031649 | 1476 | Up | 2.40E-12 |
| Bra001046 | 1335 | Up | 2.31E-111 |
| Bra011254 | 1173 | Up | 0.00016686 |
| Bra002527 | 651 | Up | 4.38E-08 |
| Bra030653 | 999 | Up | 3.52E-25 |
| Bra034608 | 1452 | Up | 0.00024883 |
| Bra017310 | 1923 | Up | 0.00024887 |
| Bra014764 | 2136 | Up | 9.79E-08 |
| Bra030214 | 1584 | Up | 0.00036923 |
| Bra002619 | 918 | Up | 3.16E-05 |
| Bra010084 | 906 | Up | 4.99E-14 |
| Bra016934 | 474 | Up | 3.08E-92 |
| Bra005812 | 2181 | Up | 4.69E-05 |
| Bra038589 | 1170 | Up | 1.66E-20 |
| Bra025258 | 606 | Up | 0.00081252 |
| Bra037277 | 1656 | Up | 8.85E-06 |
| Bra005687 | 930 | Up | 1.65E-57 |
| Bra011844 | 1719 | Up | 1.16E-16 |
| Bra026665 | 1785 | Up | 1.24E-14 |
| Bra030320 | 1005 | Up | 1.32E-05 |
| Bra002703 | 2940 | Up | 5.27E-37 |
| Bra034306 | 1407 | Up | 1.66E-06 |
| Bra030558 | 2211 | Up | 2.46E-06 |
| Bra025668 | 1014 | Up | 3.02E-07 |
| Bra024174 | 462 | Up | 3.01E-07 |
| Bra000122 | 1653 | Up | 5.63E-08 |
| Bra027089 | 4809 | Up | 1.26E-09 |
| Bra023224 | 1782 | Up | 6.79E-07 |
| Bra037798 | 426 | Up | 2.53E-19 |
| Bra002177 | 1233 | Up | 4.99E-49 |
| Bra019654 | 1263 | Up | 4.35E-05 |
| Bra009131 | 1026 | Up | 1.55E-08 |
| Bra018161 | 216 | Up | 0.00034174 |
| Bra000115 | 981 | Up | 8.19E-06 |
| Bra010401 | 2244 | Up | 1.09E-17 |
| Bra003615 | 1530 | Up | 6.48E-05 |
| Bra018694 | 999 | Up | 2.79E-07 |
| Bra026474 | 780 | Up | 2.28E-12 |
| Bra007825 | 1728 | Up | 0.00075182 |
| Bra002627 | 1602 | Up | 9.50E-32 |
| Bra016103 | 1527 | Up | 0.00014275 |
| Bra040029 | 1494 | Up | 1.74E-13 |
| Bra011690 | 2148 | Up | 1.73E-07 |
| Bra007494 | 1263 | Up | 5.92E-38 |
| Bra025082 | 462 | Up | 1.09E-27 |
| Bra020445 | 1680 | Up | 2.10E-06 |
| Bra015272 | 468 | Up | 0.00031546 |
| Bra029587 | 1665 | Up | 2.41E-22 |
| Bra009595 | 243 | Up | 2.22E-40 |
| Bra039971 | 789 | Up | 1.60E-07 |
| Bra011899 | 810 | Up | 8.89E-05 |
| Bra040416 | 1278 | Up | 8.12E-09 |
| Bra006872 | 285 | Up | 8.02E-19 |
| Bra030528 | 2211 | Up | 4.69E-06 |
| Bra007202 | 1023 | Up | 0.00046847 |
| Bra035426 | 1863 | Up | 0.00046832 |
| Bra011835 | 1038 | Up | 0.00046839 |
| Bra007615 | 1344 | Up | 1.24E-34 |
| Bra019765 | 1326 | Up | 3.17E-11 |
| Bra039271 | 402 | Up | 1.12E-08 |
| Bra018150 | 1584 | Up | 2.46E-15 |
| Bra004721 | 1029 | Up | 3.00E-30 |
| Bra016892 | 435 | Up | 5.51E-05 |
| Bra002315 | 3555 | Up | 7.67E-14 |
| Bra014915 | 324 | Up | 0.00029141 |
| Bra002982 | 615 | Up | 2.52E-08 |
| Bra003419 | 738 | Up | 6.87E-09 |
| Bra014221 | 666 | Up | 2.29E-05 |
| Bra039452 | 1821 | Up | 2.81E-35 |
| Bra027967 | 2097 | Up | 8.21E-37 |
| Bra033057 | 1164 | Up | 2.14E-10 |
| Bra018426 | 1215 | Up | 5.62E-08 |
| Bra026631 | 2751 | Up | 4.34E-26 |
| Bra022034 | 3264 | Up | 3.96E-06 |
| Bra039329 | 1410 | Up | 5.79E-33 |
| Bra015679 | 1029 | Up | 1.42E-05 |
| Bra007914 | 1833 | Up | 5.07E-05 |
| Bra005207 | 4272 | Up | 0.00018025 |
| Bra008792 | 1188 | Up | 7.51E-45 |
| Bra012324 | 1473 | Up | 0.000639 |
| Bra025792 | 819 | Up | 2.86E-12 |
| Bra037280 | 1233 | Up | 5.92E-06 |
| Bra021759 | 1914 | Up | 4.55E-47 |
| Bra035917 | 417 | Up | 1.81E-10 |
| Bra030337 | 1092 | Up | 6.10E-12 |
| Bra007212 | 1731 | Up | 3.23E-35 |
| Bra005236 | 1122 | Up | 4.60E-62 |
| Bra030826 | 975 | Up | 8.78E-06 |
| Bra015306 | 1041 | Up | 8.78E-06 |
| Bra039440 | 546 | Up | 8.71E-09 |
| Bra024740 | 693 | Up | 0.000944 |
| Bra010997 | 1470 | Up | 0.00094385 |
| Bra036465 | 1440 | Up | 1.12E-10 |
| Bra039748 | 657 | Up | 1.30E-08 |
| Bra001573 | 1821 | Up | 4.12E-07 |
| Bra004267 | 597 | Up | 2.20E-24 |
| Bra038674 | 1338 | Up | 8.57E-21 |
| Bra029558 | 1257 | Up | 0.00016527 |
| Bra010102 | 1467 | Up | 1.53E-10 |
| Bra032902 | 1830 | Up | 1.04E-13 |
| Bra037741 | 1458 | Up | 4.89E-09 |
| Bra003527 | 4323 | Up | 8.29E-196 |
| Bra037254 | 2949 | Up | 3.35E-06 |
| Bra034519 | 414 | Up | 2.18E-28 |
| Bra014125 | 1071 | Up | 1.48E-35 |
| Bra028006 | 1440 | Up | 2.33E-24 |
| Bra035664 | 1356 | Up | 2.05E-12 |
| Bra035897 | 600 | Up | 1.14E-34 |
| Bra002216 | 711 | Up | 4.18E-56 |
| Bra036647 | 1218 | Up | 6.67E-23 |
| Bra029656 | 1188 | Up | 0.00036305 |
| Bra008242 | 585 | Up | 2.26E-22 |
| Bra040870 | 1365 | Up | 9.44E-31 |
| Bra034941 | 1500 | Up | 1.20E-75 |
| Bra012474 | 1761 | Up | 7.94E-15 |
| Bra012230 | 663 | Up | 8.40E-23 |
| Bra028861 | 1917 | Up | 2.27E-49 |
| Bra027394 | 1518 | Up | 3.17E-07 |
| Bra014283 | 504 | Up | 3.76E-09 |
| Bra039280 | 963 | Up | 6.35E-10 |
| Bra002955 | 1245 | Up | 1.63E-05 |
| Bra010751 | 1674 | Up | 1.63E-30 |
| Bra030676 | 1518 | Up | 3.92E-49 |
| Bra036724 | 1575 | Up | 1.09E-16 |
| Bra009149 | 1467 | Up | 1.13E-38 |
| Bra030166 | 2178 | Up | 0.00033249 |
| Bra030696 | 1005 | Up | 2.41E-05 |
| Bra000587 | 1632 | Up | 1.34E-12 |
| Bra017207 | 708 | Up | 7.09E-07 |
| Bra008265 | 975 | Up | 4.11E-11 |
| Bra014820 | 2145 | Up | 0.00013864 |
| Bra009420 | 1776 | Up | 9.94E-18 |
| Bra018622 | 417 | Up | 7.42E-08 |
| Bra009090 | 1287 | Up | 5.07E-13 |
| Bra030644 | 1152 | Up | 1.30E-09 |
| Bra010740 | 3498 | Up | 3.09E-13 |
| Bra037739 | 1551 | Up | 8.59E-05 |
| Bra024708 | 1446 | Up | 1.14E-08 |
| Bra005847 | 1998 | Up | 9.19E-06 |
| Bra031210 | 507 | Up | 9.70E-71 |
| Bra008080 | 2220 | Up | 2.20E-05 |
| Bra013843 | 1854 | Up | 3.95E-07 |
| Bra006943 | 417 | Up | 0.00012708 |
| Bra004126 | 552 | Up | 3.39E-25 |
| Bra003194 | 942 | Up | 6.30E-13 |
| Bra013077 | 4380 | Up | 9.38E-13 |
| Bra029372 | 468 | Up | 2.24E-12 |
| Bra002018 | 960 | Up | 1.42E-14 |
| Bra020801 | 1551 | Up | 0.00018802 |
| Bra013426 | 810 | Up | 1.24E-05 |
| Bra015501 | 783 | Up | 6.51E-141 |
| Bra041073 | 966 | Up | 3.18E-06 |
| Bra001789 | 1629 | Up | 2.59E-33 |
| Bra010967 | 1269 | Up | 0.00027818 |
| Bra010937 | 726 | Up | 8.04E-07 |
| Bra016719 | 471 | Up | 1.07E-48 |
| Bra032649 | 2283 | Up | 5.16E-08 |
| Bra008966 | 1971 | Up | 0.00066212 |
| Bra018940 | 3540 | Up | 2.60E-103 |
| Bra011085 | 639 | Up | 4.72E-06 |
| Bra027764 | 2847 | Up | 5.93E-17 |
| Bra020541 | 1056 | Up | 3.00E-07 |
| Bra002839 | 453 | Up | 1.14E-05 |
| Bra027206 | 660 | Up | 5.42E-18 |
| Bra002264 | 1128 | Up | 1.34E-18 |
| Bra008984 | 1281 | Up | 0.00040971 |
| Bra016060 | 531 | Up | 6.97E-06 |
| Bra028595 | 2034 | Up | 2.20E-13 |
| Bra033035 | 1104 | Up | 6.98E-08 |
| Bra013909 | 573 | Up | 0.00025353 |
| Bra002096 | 1350 | Up | 4.30E-06 |
| Bra013983 | 1725 | Up | 6.76E-19 |
| Bra002223 | 1716 | Up | 2.64E-06 |
| Bra008107 | 1896 | Up | 6.14E-19 |
| Bra008341 | 492 | Up | 4.03E-05 |
| Bra035332 | 816 | Up | 0.00015588 |
| Bra025924 | 504 | Up | 6.48E-11 |
| Bra016937 | 807 | Up | 0.00060442 |
| Bra016503 | 633 | Up | 7.33E-13 |
| Bra010581 | 939 | Up | 1.63E-06 |
| Bra015511 | 1332 | Up | 6.55E-12 |
| Bra031371 | 783 | Up | 2.46E-11 |
| Bra034128 | 1917 | Up | 1.44E-69 |
| Bra007906 | 4380 | Up | 1.72E-22 |
| Bra002582 | 492 | Up | 1.61E-12 |
| Bra026869 | 1443 | Up | 9.42E-06 |
| Bra040761 | 2796 | Up | 5.45E-09 |
| Bra003659 | 957 | Up | 1.37E-13 |
| Bra029773 | 996 | Up | 2.18E-08 |
| Bra024020 | 1230 | Up | 7.94E-11 |
| Bra033239 | 858 | Up | 7.55E-15 |
| Bra018873 | 372 | Up | 0.00055122 |
| Bra011041 | 2214 | Up | 1.40E-05 |
| Bra022267 | 1056 | Up | 8.79E-05 |
| Bra017981 | 2301 | Up | 0.00055113 |
| Bra012437 | 1845 | Up | 4.66E-10 |
| Bra034697 | 1524 | Up | 1.13E-09 |
| Bra026630 | 1002 | Up | 4.50E-09 |
| Bra011988 | 3012 | Up | 2.06E-05 |
| Bra023434 | 1842 | Up | 4.24E-10 |
| Bra005919 | 996 | Up | 8.93E-30 |
| Bra038661 | 2145 | Up | 0.00080713 |
| Bra010757 | 1149 | Up | 6.28E-10 |
| Bra038369 | 1056 | Up | 4.67E-16 |
| Bra033225 | 3213 | Up | 3.54E-19 |
| Bra014208 | 1428 | Up | 5.80E-183 |
| Bra026409 | 2274 | Up | 1.78E-44 |
| Bra016636 | 1899 | Up | 4.80E-06 |
| Bra027940 | 765 | Up | 5.83E-54 |
| Bra023965 | 1152 | Up | 2.95E-06 |
| Bra011358 | 510 | Up | 1.11E-06 |
| Bra009751 | 2223 | Up | 0.00019093 |
| Bra000698 | 1155 | Up | 0.00011812 |
| Bra008430 | 456 | Up | 5.18E-10 |
| Bra004880 | 1188 | Up | 7.25E-05 |
| Bra040375 | 699 | Up | 9.55E-08 |
| Bra006585 | 1206 | Up | 4.47E-05 |
| Bra000227 | 375 | Up | 9.99E-26 |
| Bra030423 | 558 | Up | 0.00073492 |
| Bra004710 | 2928 | Up | 5.74E-101 |
| Bra025375 | 906 | Up | 1.70E-05 |
| Bra019659 | 591 | Up | 9.14E-29 |
| Bra015961 | 1194 | Up | 2.29E-07 |
| Bra030560 | 690 | Up | 0.00045425 |
| Bra003009 | 1053 | Up | 9.55E-70 |
| Bra035756 | 1563 | Up | 8.67E-08 |
| Bra010936 | 2112 | Up | 0.00028126 |
| Bra030747 | 1413 | Up | 0.00028121 |
| Bra009543 | 531 | Up | 6.12E-12 |
| Bra027649 | 1263 | Up | 1.54E-05 |
| Bra003288 | 1635 | Up | 9.49E-06 |
| Bra009422 | 723 | Up | 5.86E-06 |
| Bra008657 | 825 | Up | 2.09E-11 |
| Bra011819 | 1557 | Up | 9.34E-37 |
| Bra001845 | 1467 | Up | 1.54E-09 |
| Bra008240 | 2187 | Up | 5.53E-27 |
| Bra007336 | 1158 | Up | 8.07E-11 |
| Bra036751 | 3306 | Up | 0.00025523 |
| Bra013161 | 1170 | Up | 9.53E-17 |
| Bra027737 | 453 | Up | 5.64E-46 |
| Bra003835 | 702 | Up | 5.10E-49 |
| Bra025494 | 2997 | Up | 1.71E-07 |
| Bra028219 | 2658 | Up | 5.14E-33 |
| Bra009181 | 810 | Up | 1.99E-96 |
| Bra006812 | 3189 | Up | 8.37E-13 |
| Bra024139 | 723 | Up | 2.62E-10 |
| Bra033355 | 693 | Up | 1.62E-19 |
| Bra007051 | 933 | Up | 2.86E-16 |
| Bra009969 | 1824 | Up | 5.42E-05 |
| Bra028999 | 567 | Up | 4.80E-06 |
| Bra016185 | 498 | Up | 7.79E-06 |
| Bra011268 | 1803 | Up | 2.54E-41 |
| Bra005581 | 1746 | Up | 2.77E-09 |
| Bra036512 | 1743 | Up | 2.67E-06 |
| Bra033604 | 1572 | Up | 1.89E-11 |
| Bra020508 | 1164 | Up | 8.04E-126 |
| Bra005229 | 1440 | Up | 3.51E-10 |
| Bra032558 | 4656 | Up | 1.80E-08 |
| Bra018933 | 2622 | Up | 2.78E-11 |
| Bra011877 | 1104 | Up | 5.96E-22 |
| Bra016945 | 864 | Up | 0.00088659 |
| Bra009263 | 1236 | Up | 0.00088672 |
| Bra035731 | 1029 | Up | 0.00088686 |
| Bra027521 | 819 | Up | 6.42E-06 |
| Bra023900 | 1092 | Up | 1.69E-05 |
| Bra030449 | 2085 | Up | 4.45E-05 |
| Bra008919 | 1293 | Up | 7.21E-05 |
| Bra025081 | 1170 | Up | 7.21E-05 |
| Bra016732 | 546 | Up | 7.21E-05 |
| Bra024379 | 1707 | Up | 0.00018974 |
| Bra019852 | 1107 | Up | 3.57E-06 |
| Bra001598 | 585 | Up | 0.00030746 |
| Bra003057 | 873 | Up | 0.00049665 |
| Bra004424 | 309 | Up | 0.00049657 |
| Bra010116 | 690 | Up | 1.69E-14 |
| Bra015706 | 195 | Up | 0.00080178 |
| Bra033644 | 537 | Up | 0.00080165 |
| Bra019448 | 3900 | Up | 1.68E-09 |
| Bra029837 | 2022 | Up | 6.01E-30 |
| Bra010015 | 1623 | Up | 1.98E-06 |
| Bra035194 | 921 | Up | 1.20E-08 |
| Bra010498 | 2154 | Up | 1.09E-28 |
| Bra040762 | 1449 | Up | 2.81E-15 |
| Bra039676 | 1767 | Up | 3.65E-05 |
| Bra011282 | 969 | Up | 2.91E-06 |
| Bra018250 | 1125 | Up | 1.74E-10 |
| Bra033775 | 1803 | Up | 1.01E-14 |
| Bra033411 | 1272 | Up | 9.78E-09 |
| Bra003007 | 1095 | Up | 2.63E-08 |
| Bra014105 | 525 | Up | 0.00015484 |
| Bra038804 | 585 | Up | 2.03E-05 |
| Bra034254 | 1869 | Up | 1.14E-11 |
| Bra039061 | 2031 | Up | 1.17E-23 |
| Bra013931 | 972 | Up | 6.90E-19 |
| Bra037405 | 408 | Up | 3.29E-05 |
| Bra027317 | 1779 | Up | 5.43E-07 |
| Bra010055 | 1218 | Up | 5.48E-13 |
| Bra001122 | 423 | Up | 1.82E-09 |
| Bra026581 | 1737 | Up | 1.14E-07 |
| Bra036394 | 351 | Up | 0.00040639 |
| Bra022980 | 852 | Up | 0.00040632 |
| Bra003069 | 696 | Up | 0.00065665 |
| Bra006850 | 2169 | Up | 0.00065675 |
| Bra023872 | 1494 | Up | 4.10E-18 |
| Bra039238 | 2193 | Up | 4.93E-12 |
| Bra016276 | 1506 | Up | 0.00036728 |
| Bra037201 | 1599 | Up | 1.84E-29 |
| Bra040669 | 1668 | Up | 6.60E-14 |
| Bra015336 | 432 | Up | 4.36E-05 |
| Bra023704 | 1170 | Up | 0.0002052 |
| Bra037814 | 1452 | Up | 1.57E-08 |
| Bra019468 | 1161 | Up | 0.00095713 |
| Bra027685 | 1464 | Up | 3.00E-25 |
| Bra007795 | 1050 | Up | 3.69E-10 |
| Bra039513 | 789 | Up | 5.14E-06 |
| Bra017823 | 609 | Up | 8.79E-18 |
| Bra002220 | 450 | Up | 3.02E-46 |
| Bra031081 | 1536 | Up | 1.23E-07 |
| Bra028478 | 2313 | Up | 2.31E-08 |
| Bra022917 | 1752 | Up | 0.00053724 |
| Bra017596 | 624 | Up | 5.51E-70 |
| Bra012127 | 2007 | Up | 7.70E-165 |
| Bra029788 | 1146 | Up | 2.19E-05 |
| Bra027332 | 3129 | Up | 7.50E-06 |
| Bra015697 | 1023 | Up | 6.38E-15 |
| Bra024567 | 1818 | Up | 0.00086551 |
| Bra039261 | 480 | Up | 0.00010345 |
| Bra004835 | 1521 | Up | 8.23E-35 |
| Bra005598 | 1323 | Up | 3.75E-28 |
| Bra033224 | 1212 | Up | 4.66E-20 |
| Bra004520 | 819 | Up | 1.10E-05 |
| Bra010339 | 1902 | Up | 2.72E-29 |
| Bra009651 | 1086 | Up | 3.87E-07 |
| Bra008353 | 2055 | Up | 6.10E-11 |
| Bra009529 | 1500 | Up | 1.67E-14 |
| Bra026758 | 1977 | Up | 2.14E-07 |
| Bra040749 | 1374 | Up | 3.76E-142 |
| Bra016549 | 2460 | Up | 4.05E-08 |
| Bra023412 | 825 | Up | 2.54E-09 |
| Bra014632 | 4545 | Up | 8.40E-05 |
| Bra016866 | 2895 | Up | 1.32E-100 |
| Bra001891 | 1389 | Up | 1.44E-49 |
| Bra021902 | 615 | Up | 4.67E-05 |
| Bra024640 | 2235 | Up | 8.93E-06 |
| Bra006068 | 1650 | Up | 1.33E-16 |
| Bra011069 | 1281 | Up | 0.00013576 |
| Bra028445 | 402 | Up | 2.17E-17 |
| Bra023866 | 1464 | Up | 4.99E-12 |
| Bra009182 | 648 | Up | 7.57E-05 |
| Bra018744 | 1218 | Up | 1.79E-14 |
| Bra032034 | 750 | Up | 9.67E-08 |
| Bra002692 | 1893 | Up | 1.10E-58 |
| Bra018019 | 1545 | Up | 1.15E-44 |
| Bra032010 | 633 | Up | 4.21E-05 |
| Bra012807 | 942 | Up | 3.71E-47 |
| Bra004109 | 1671 | Up | 1.91E-21 |
| Bra013382 | 534 | Up | 1.91E-10 |
| Bra035451 | 849 | Up | 2.61E-16 |
| Bra018357 | 2220 | Up | 1.63E-08 |
| Bra014638 | 882 | Up | 0.00063649 |
| Bra011916 | 936 | Up | 4.37E-15 |
| Bra032998 | 3714 | Up | 1.73E-10 |
| Bra017217 | 678 | Up | 6.83E-05 |
| Bra000005 | 1434 | Up | 1.55E-10 |
| Bra011333 | 1884 | Up | 1.88E-12 |
| Bra014923 | 873 | Up | 1.24E-06 |
| Bra000913 | 1953 | Up | 6.15E-05 |
| Bra021392 | 1983 | Up | 6.63E-61 |
| Bra003531 | 765 | Up | 3.73E-07 |
| Bra029688 | 1221 | Up | 1.11E-06 |
| Bra016494 | 2715 | Up | 6.35E-08 |
| Bra010858 | 1173 | Up | 5.89E-06 |
| Bra013856 | 1134 | Up | 1.86E-07 |
| Bra035926 | 975 | Up | 1.03E-07 |
| Bra013066 | 495 | Up | 1.78E-09 |
| Bra033399 | 861 | Up | 9.98E-07 |
| Bra017019 | 1425 | Up | 1.11E-25 |
| Bra007162 | 1353 | Up | 1.66E-10 |
| Bra026991 | 1770 | Up | 3.91E-19 |
| Bra013123 | 486 | Up | 6.29E-102 |
| Bra025529 | 2076 | Up | 2.77E-05 |
| Bra001488 | 4482 | Up | 4.38E-10 |
| Bra022326 | 1386 | Up | 1.47E-06 |
| Bra014655 | 1146 | Up | 0.00083058 |
| Bra010593 | 918 | Up | 0.00046492 |
| Bra005077 | 1578 | Up | 0.00025958 |
| Bra017355 | 486 | Up | 4.48E-05 |
| Bra003231 | 870 | Up | 4.48E-05 |
| Bra003814 | 1176 | Up | 6.39E-10 |
| Bra026192 | 2184 | Up | 2.67E-20 |
| Bra002512 | 1455 | Up | 7.84E-14 |
| Bra008680 | 1341 | Up | 4.03E-05 |
| Bra023984 | 1599 | Up | 2.54E-28 |
| Bra038942 | 846 | Up | 2.64E-32 |
| Bra004158 | 867 | Up | 8.61E-27 |
| Bra037296 | 516 | Up | 1.98E-191 |
| Bra001345 | 342 | Up | 3.97E-18 |
| Bra019867 | 2409 | Up | 5.20E-20 |
| Bra020529 | 2166 | Up | 1.27E-10 |
| Bra019959 | 567 | Up | 3.64E-05 |
| Bra003543 | 1725 | Up | 3.63E-05 |
| Bra017940 | 2118 | Up | 3.44E-57 |
| Bra010728 | 519 | Up | 2.43E-08 |
| Bra006372 | 831 | Up | 3.26E-05 |
| Bra002106 | 2151 | Up | 5.87E-05 |
| Bra003177 | 726 | Up | 2.32E-07 |
| Bra036883 | 576 | Up | 4.18E-22 |
| Bra033251 | 1668 | Up | 6.13E-22 |
| Bra011185 | 2259 | Up | 1.46E-09 |
| Bra002031 | 1233 | Up | 0.00016948 |
| Bra016686 | 822 | Up | 3.18E-08 |
| Bra016252 | 693 | Up | 1.47E-05 |
| Bra034277 | 1188 | Up | 1.47E-05 |
| Bra015711 | 1398 | Up | 0.00030505 |
| Bra009854 | 1164 | Up | 1.62E-66 |
| Bra036069 | 1353 | Up | 4.73E-05 |
| Bra018529 | 981 | Up | 0.00054597 |
| Bra009006 | 3075 | Up | 9.26E-40 |
| Bra009352 | 1533 | Up | 7.78E-09 |
| Bra014298 | 1452 | Up | 9.36E-08 |
| Bra038281 | 1002 | Up | 3.39E-46 |
| Bra033006 | 846 | Up | 1.32E-05 |
| Bra024851 | 1710 | Up | 0.00027417 |
| Bra033142 | 1668 | Up | 1.82E-06 |
| Bra024559 | 1830 | Up | 4.93E-07 |
| Bra001159 | 1437 | Up | 2.13E-05 |
| Bra020836 | 1488 | Up | 1.52E-63 |
| Bra031205 | 567 | Up | 1.97E-07 |
| Bra034523 | 2139 | Up | 7.74E-28 |
| Bra036205 | 957 | Up | 8.58E-06 |
| Bra006124 | 1482 | Up | 1.26E-17 |
| Bra016935 | 285 | Up | 0.00039581 |
| Bra032089 | 1200 | Up | 0.00011114 |
| Bra015881 | 648 | Up | 0.00039588 |
| Bra003889 | 2901 | Up | 3.45E-06 |
| Bra021736 | 924 | Up | 6.74E-22 |
| Bra024068 | 753 | Up | 1.10E-11 |
| Bra039967 | 789 | Up | 1.46E-22 |
| Bra006700 | 2484 | Up | 0.00015951 |
| Bra036004 | 1878 | Up | 0.00015948 |
| Bra009768 | 1692 | Up | 2.83E-11 |
| Bra017724 | 1308 | Up | 5.00E-06 |
| Bra026988 | 1380 | Up | 1.24E-06 |
| Bra017672 | 2067 | Up | 1.71E-12 |
| Bra021408 | 1830 | Up | 1.24E-06 |
| Bra005059 | 1701 | Up | 0.0005131 |
| Bra009028 | 1983 | Up | 1.17E-17 |
| Bra007731 | 270 | Up | 1.03E-30 |
| Bra036406 | 1188 | Up | 1.83E-08 |
| Bra020504 | 1464 | Up | 6.47E-05 |
| Bra029670 | 2103 | Up | 1.96E-31 |
| Bra000377 | 396 | Up | 4.81E-56 |
| Bra024255 | 1068 | Up | 8.40E-12 |
| Bra039844 | 1524 | Up | 1.94E-57 |
| Bra003055 | 2001 | Up | 1.48E-11 |
| Bra034284 | 1809 | Up | 1.45E-05 |
| Bra000635 | 2079 | Up | 1.08E-07 |
| Bra037384 | 690 | Up | 0.00020678 |
| Bra009968 | 1056 | Up | 0.00010397 |
